# Asymmetric Hydroamination Using Oxidative Radical Initiation in Flavin Enzymes

**DOI:** 10.1021/jacs.6c04368

**Published:** 2026-06-12

**Authors:** Alexandra C. Brown, Carlos E. Del Angel Aguilar, Felix C. Raps, Paul S. Riehl, Todd K. Hyster

**Affiliations:** Department of Chemistry, 6740 Princeton University, Princeton, New Jersey 08544, United States

## Abstract

Asymmetric radical alkene hydroamination offers a potentially powerful strategy to form enantioenriched, saturated *N-*heterocycles, but these reactions have proved challenging to render stereoselective. Here, we report an engineered flavin enzyme that catalyzes the formation of chiral pyrrolidines with high yield and enantioselectivity. Development of this transformation addresses a long-standing limitation in flavin photobiocatalysis: the short lifetime of the photoexcited flavin quinone has previously made it challenging to leverage this species as a photooxidant. We report that the photophysical properties of a flavin enzyme can be tuned via rational mutagenesis to obtain a variant with a long-lived excited state, and that this enzyme can be further engineered to create an efficient catalyst for asymmetric radical hydroaminations. Moreover, this work introduces two mechanistic discoveries that can be applied broadly in photobiocatalysis: (1) an exogeneous cophotocatalyst can serve as a photoprotectant for the enzymatic cofactor and (2) enantiospecific termination of chiral radical intermediates provides a mechanism to achieve stereocontrol over previously challenging classes of transformations.

## Graphical abstract

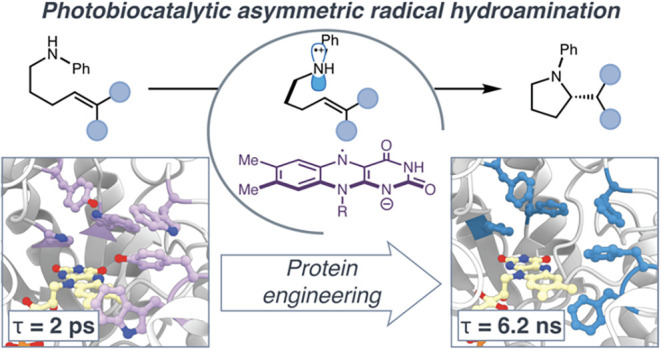

## Introduction

Photobiocatalysis offers an appealing strategy to discover new enzymatic reactivity by using light to drive formation of reactive intermediates within protein active sites.^1,2^ Subsequent optimization of the protein scaffold using directed evolution can direct these high-energy intermediates toward divergent and highly selective reaction pathways.^3–5^ This feature enables engineered enzymes to address reactivity and selectivity challenges that plague small-molecule catalytic methods. Our group and others have demonstrated that common flavoenzymes can catalyze a wide range of transformations in which flavin hydroquinone (FMN_HQ_) serves as a photoreductant to generate substrate-centered radicals.^1,2,6^ Controlling the subsequent reactivity of these intermediates by tuning the protein scaffold has enabled the synthesis of myriad enantioenriched products via non-native reaction pathways (Figure 1A).

**Figure 1:**
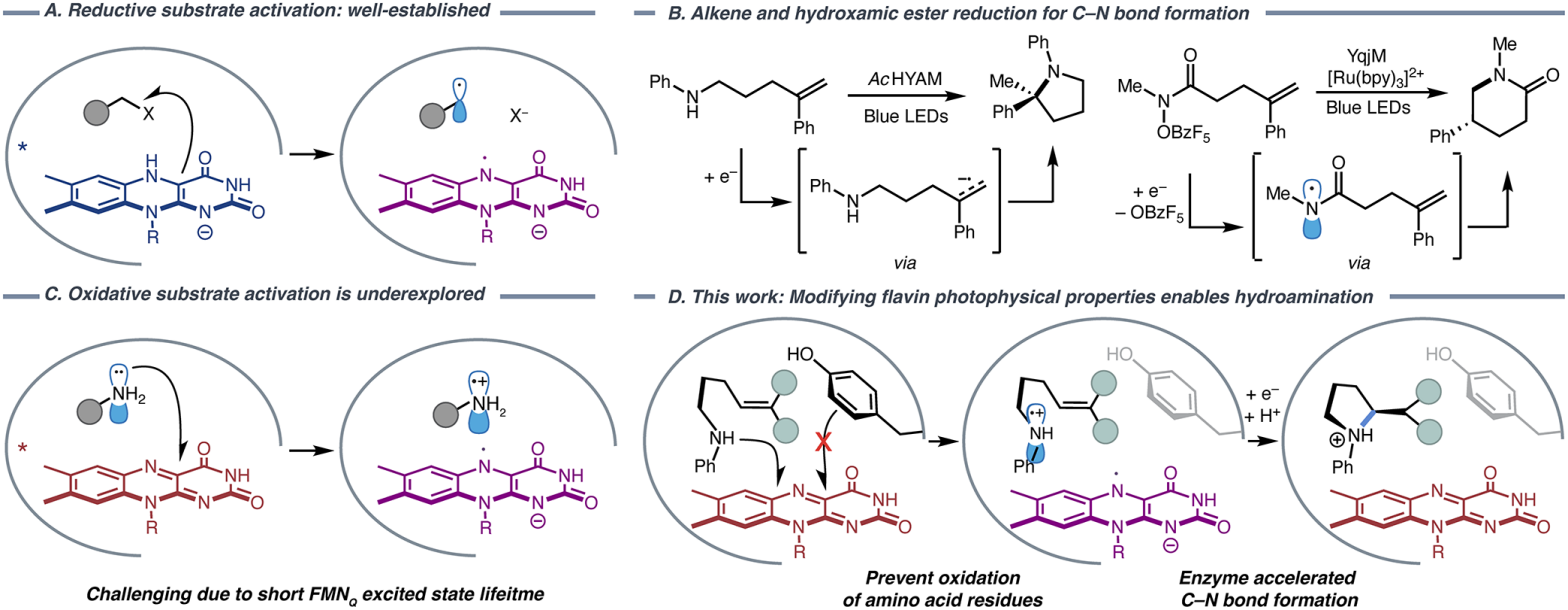
Electron transfer in photocatalytic flavin enzymes. (A). Reductive substrate activation from the flavin hydroquinone (FMN_HQ_) is a well-established strategy. (B). Methods to form C–N bonds by reduction of alkenes or reductive cleavage of hydroxamic esters. (C). Oxidative substrate activation is uncommon due to the short excited state lifetime for FMN_Q_. (D). This work introduces a strategy for oxidative substrate activation by tuning flavin photophysical properties to enable alkene hydroaminations.

Chiral *N*-heterocycles are critical motifs in many pharmaceutical and agrochemical molecules^7^ and are most commonly synthesized using enantioselective imine or heteroaromatic reduction methods.^8–10^ We are interested in developing flavin photobiocatalytic methodologies for preparing enantioenriched *N*-heterocycles via C–N bond formation, thereby enabling greater variation in the position and functionality of stereocenters in these molecules. Our group previously reported photobiocatalytic flavin-dependent enantioselective *N*-heterocycle synthesis via reduction of either styrenyl alkenes^11^ or hydroxamic esters^12^ (Figure 1B); we and others later reported variations on both methods.^13–15^ However, these methods require either alkenes with relatively high reduction potentials or prefunctionalized amides, respectively, which limits their synthetic utility.

Toward the goal of developing a photobiocatalytic C–N bond-forming strategy that is compatible with unactivated alkenes (which are thermodynamically challenging to reduce)^16^ and unfunctionalized amines, we questioned whether flavoenzymes could be used to oxidize amines to aminium radical cations. Aryl aminium radical cations react rapidly with styrenyl alkenes to afford hydroaminated products,^17–19^ however these reactions have yet to be rendered asymmetric. Moreover, small molecule photoredox systems struggle to match the kinetics of electron transfer and C–N bond formation, thereby limiting the substrate classes compatible with these transformations. For example, although hydroamination of unactivated alkenes with *alkyl amines* proceeds efficiently (*k* = 10^8^ M^–1^ s^–1^),^18,20^ the functionalization of unactivated alkenes with *anilines* does not proceed.^21^ This is attributed to the slower rates of C–N bond formation between alkenes and anilines (*k* < 10^6^ M^–1^ s^–1^),^20^ which results in back electron transfer (BET) to the aminium radical cation from the reduced photocatalyst outcompeting productive hydroamination.^21^ We hypothesized that an enzyme scaffold could simultaneously overcome the kinetic challenge of this reaction and render the transformation stereoselective by preorganizing the substrate for rapid, stereocontrolled cyclization within the enzyme active site.

To develop oxidatively initiated hydroamination reactions, we must first address a long-standing limitation of flavin photobiocatalysis: the excited-state lifetime of the oxidized flavin cofactor, FMN_Q_, is exceedingly short, making it challenging to direct toward productive substrate oxidation.^22^ Instead, photoexcitation of FMN_Q_ leads to rapid, picosecond time scale oxidation of protein residues (e.g., tryptophan, tyrosine, histidine) within the active site and quenching of the FMN_Q_ excited state (FMN_Q_*, Figure 1C).^22–25^ As a result, previous examples of oxidatively initiated photobiocatalytic transformations in ene-reductase-containing catalytic systems have typically employed a cophotocatalyst; the presence of two potential photooxidants in these reactions makes it challenging to determine whether FMN_Q_ is responsible for substrate oxidation, and in some cases the cophotocatalyst is established as the active photooxidant.^26–33^ Among flavin-dependent enzymes more broadly, fatty acid photodecarboxylases^34–37^ and engineered lactate monooxygenases,^23,38^ are important exceptions to this paradigm. In these enzymes, the absence of oxidizable amino acid residues in the vicinity of the flavin cofactor leads to a long-lived excited state that can oxidize carboxylic acids.

Here, we demonstrate that the photophysical properties of FMN_Q_ in a stable and soluble ERED protein scaffold can be tuned using rational mutagenesis to achieve long-lived FMN_Q_ excited states and overcome limitations on oxidative radical initiation. Using engineered protein variants, we catalyze the stereoselective hydroamination of unactivated alkenes with anilines, enabled by enzymatic acceleration of the kinetically challenging C–N bond formation. Moreover, mechanistic analysis reveals an unexpected mechanism of stereodetermination, in which enantiospecific transfer of a hydrogen atom determines the configuration of an adjacent stereocenter in the product via a dynamic kinetic resolution of enantiomeric radical intermediates. This unusual stereodetermination mechanism highlights the strength of enzyme catalysts in controlling highly reactive species.

## Results

We began our exploration of oxidative hydroaminations by screening our in-house libraries of flavin-dependent ‘ene’-reductases (EREDs) for the hydroamination of compound **1** (Figure 2A).^39^ Compound **1** was selected because small molecule catalysts have proved inefficient for hydroamination of similar substrates.^21^ Moreover, asymmetric radical hydroaminations of alkenes with amines via aminium radical cations have not been reported.^21,40^ Upon screening 14 enzymes from our existing ERED libraries, (Table S1 and Figure 2B) we could not identify any that catalyzed the hydroamination of **1** in more than trace yield. That none of the EREDs in our library were natively capable of catalyzing the hydroamination of **1** is consistent with our hypothesis that rapid quenching of FMN_Q_* by tryptophan and tyrosine residues lining the active site limits the efficiency of electron transfer from **1** to FMN_Q_* (Figure 2C). Previous work demonstrated that the fluorescence of FMN_Q_ in many EREDs is efficiently quenched by electron transfer to tryptophan and tyrosine residues in the active site on the picosecond time scale.^22,41^ Indeed, transient absorption measurements revealed the lifetime of the GluER T36A FMN_Q_ excited state to be 2 ps, too short to efficiently catalyze substrate oxidation.

**Figure 2:**
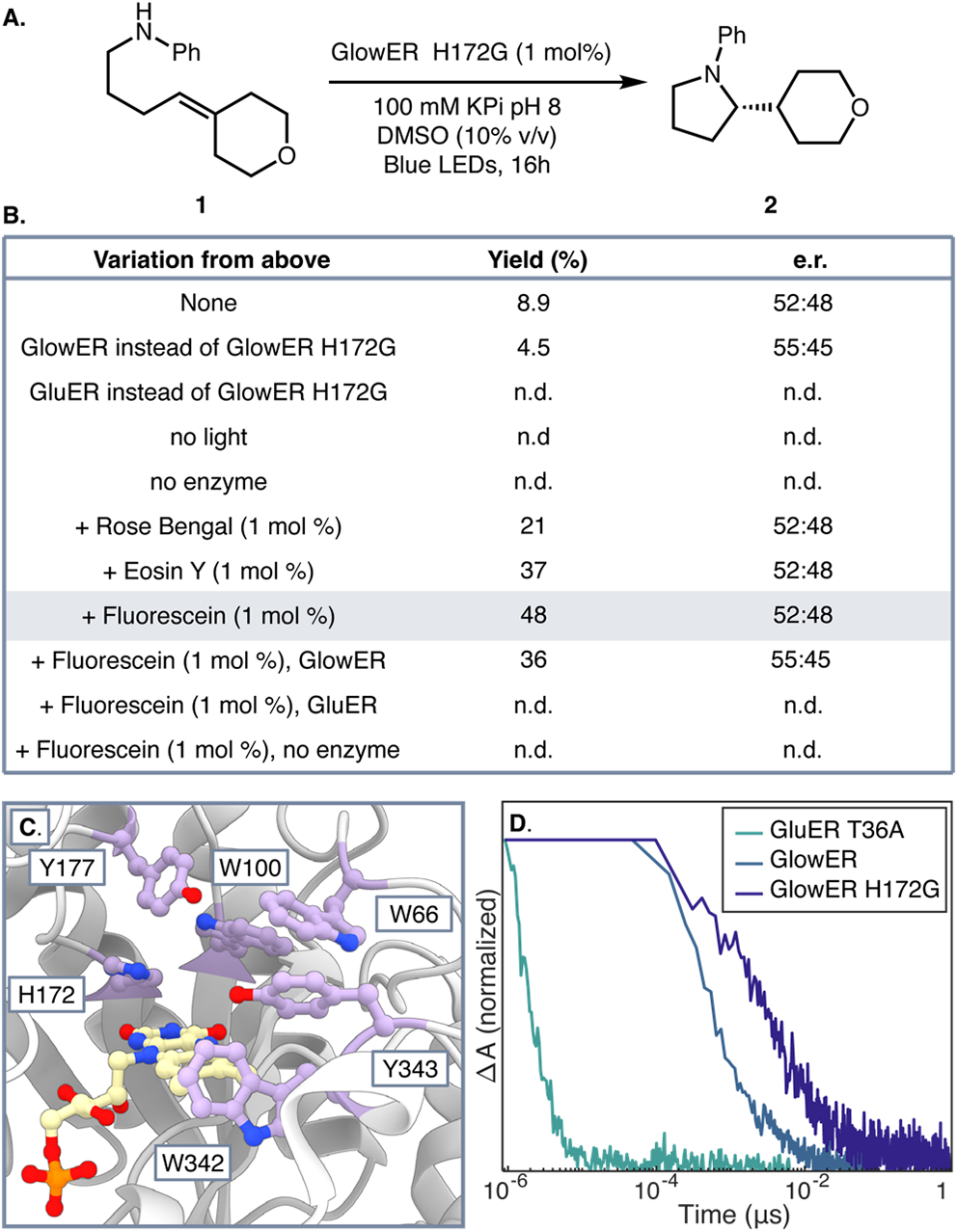
Development of hydroamination reaction. (A). Hydroamination reaction. (B). Optimization table for hydroamination with GlowER and Fluorescein. (C). Active site of GluER highlighting tyrosine, tryptophan and histidine residues in the active site. (D). Transient absorption traces at 350 nm showing the excited state lifetimes of GlowER (1.1 ns) and GlowER H172G (6.2 ns) are much longer than for GluER (2 ps).

We therefore constructed a variant of the ERED from *Gluconobacter oxidans* (GluER, previously demonstrated to be overproduced in high yield in *Escherichia coli* and to have high stability and solubility)^6^ in which all five of the tyrosine and tryptophan residues within 6 Å of the flavin cofactor were replaced with phenylalanine (GluER W66F/W100F/Y177F/W342F/Y343F, Figure 2C). We found that, unlike the native enzyme, FMN_Q_ for this 5xF variant demonstrated weak but detectable fluorescence (Figure S2). The increased fluorescence intensity for the 5xF variant—termed GlowER for its emission properties—is indicative of a longer-lived FMN_Q_*, such that the fluorescence lifetime is competitive with other excited state decay pathways. Consistent with this interpretation, the lifetime of FMN_Q_* in GlowER was measured to be 1.1 ns, over 500-fold longer than that of FMN_Q_* in GluER T36A (2 ps) and comparable to that of both free FMN_Q_* and FAD_Q_* in fatty acid photodecarboxylases.^34,42,43^

Having obtained an ERED variant with an active site that exhibits a long-lived FMN_Q_ excited state, we tested GlowER for the oxidative hydroamination of **1** (Figure 2A). Compared to GluER, which could not catalyze the transformation of **1** to **2** (<1% yield), GlowER catalyzed hydroamination of **1** in 4.5% yield with detectable enantioselectivity (55:45 e.r. favoring the (*S*) enantiomer; absolute stereochemistry established by derivatization of enantioenriched Boc-pyrrolidines).^44^ Control experiments demonstrated that both light and enzyme are essential to the reaction (Figure 2B).

We hypothesized the yield of the reaction could still be limited by electron transfer from H172, which sits only 3.1 Å away from the flavin cofactor and could also serve to quench FMN_Q_* via electron transfer. We therefore carried out site-saturation mutagenesis on H172 to determine if any other amino acids could provide an improved variant. We identified the variant GlowER H172G, which increased the yield of **2** to 8.9%, albeit with no enantioselectivity (52:48 e.r.). Consistent with the hypothesis that H172 could quench FMN_Q_*, we found that GlowER H172G has increased fluorescence intensity and a longer excited state lifetime compared to GlowER (6.2 ns, Figures 2D and S2). GlowER H172G was found to have comparable thermostability to GluER^6^ (*T*_m_ = 62.8 °C, Figure S39), however the introduction of these six mutations relative to the native enzyme, GluER, significantly ablated the native NADPH oxidation and alkene reduction activity of this “ene”-reductase derived enzyme (SI p. 100).

### Role of Fluorescein in Hydroamination Reaction

During development of the hydroamination reaction using GlowER H172G, we observed that the enzyme fluorescence decayed following 1 h of irradiation; we attributed this decay to photoreduction of FMN_Q_ as has been observed in other enzymes.^45^ As a result, we questioned if adding cophotocatalyst could increase the yield of **2**; cophotocatalysts have previously proved beneficial in flavin-catalyzed photobiocatalytic transformations.^1^ We carried out the reaction of **1** with GlowER H172G in the presence of a small panel of photocatalysts (Table S2) and identified that the xanthene-family of photocatalysts increased the yield of **2** by approximately 10-fold (Figure 2B), achieving a maximal yield of 48% with 1 mol % GlowER H172G and 1 mol % fluorescein as a cophotocatalyst. Only xanthene-family photocatalysts (including fluorescein, eosin Y, and rose bengal) were found to furnish significantly increased product yield. Rhodamine dyes (rhodamine B and 6G) provided minor increases in yield, while [Ru­(bpy)_3_]^2+^ and [Ir­(dtbbpy)­(ppy)_2_]^+^ derivatives provided no benefit (Table S2).

The observation that xanthene-family cophotocatalysts increased the yield of the hydroamination reaction raised the question of which potential photocatalyst—FMN_Q_ or fluorescein—was responsible for oxidation of **1**. We initially considered the possibility that fluorescein could serve as an oxidant upon photoexcitation, such that the enzyme—with flavin in the reduced FMN_HQ_ state—serves as a hydrogen atom donor to quench the radical following C–N bond formation and not as the source of holes to initiate the reaction. This synergistic mechanism has been previously proposed for oxidative transformations using exogenous photocatalysts in combination with flavin enzymes.^1,26^

However, in the hydroamination of **1** several observations suggested that fluorescein was not responsible for oxidation of **1**. First, Stern–Volmer fluorescence quenching of the water-soluble 4-aminopyridine substrate **11** (*vide infra*) showed that the fluorescence of GlowER H172G was more effectively quenched by **11** (*K*_SV_ = 29 M^–1^) than was the fluorescence of fluorescein (*K*_SV_ = 6 M^–1^, Figure S34). Second, we observed that fluorescein was the most efficient photocatalyst regardless of excitation wavelength, followed by eosin Y and then rose bengal (Table S3). The trend in photocatalyst efficiency is not consistent with the exogenous photocatalyst being responsible for oxidation of **1** for two reasons: (1) the shorter excited state lifetime of fluorescein compared to eosin Y and rose bengal leads to fluorescein being a generally poorer photocatalyst than heavy-atom substituted xanthene-derived photocatalysts^46,47^ and (2) fluorescein absorbs light much less efficiently at 525 nm than eosin Y or rose bengal,^46^ yet remains an effective cophotocatalyst at all wavelengths, suggesting that the efficiency of photoexcitation is not the primary determinant of cophotocatalyst efficiency (Table S3). Third, cophotocatalyst binding to the protein is essential to reactions in which the cophotocatalyst is responsible for radical generation,^26,48^ but in the hydroamination of **1**, cophotocatalyst binding efficiency is inversely correlated with reaction yield (*K*_D_ (fluorescein) = 63 μM; *K*_D_ (eosin Y) = 8.9 μM; *K*_D_ (rose bengal) = 2.5 μM; Figure S19). Lastly, a hallmark of an exogenous photocatalyst being responsible for substrate oxidation is significant racemic background reactivity in the absence of enzyme,^26^ since the photocatalyst is independently capable of substrate oxidation without enzyme. However, no cyclized product **2** is formed when amine **1** is subjected to 1 mol % fluorescein, eosin Y, or rose bengal in the absence of GlowER, nor using any of these photocatalysts in combination with 1 mol % GluER T36A. This indicates that the unique active site of GlowER with a long-lived FMN_Q_* excited state is essential to catalysis.

As described above, a combination of Stern–Volmer quenching, unusual trends in photocatalyst efficiency, and specificity for GlowER variants as catalysts indicated that fluorescein was unlikely to be the photooxidant for **1** under the reaction conditions. We therefore set out to determine alternative mechanisms by which fluorescein could improve the yield of the hydroamination with GlowER H172G using steady-state and transient ultraviolet-Visible (UV–vis) spectroscopy.

The fluorescence of GlowER H172G is much weaker than fluorescein, making it difficult to observe steady-state fluorescence for FMN_Q_* in the presence of fluorescein (Figure S4). Therefore, we turned to picosecond transient absorption UV–vis spectroscopy to directly observe the FMN_Q_ excited-state of GlowER H172G. Transient absorption spectra of GlowER H172G (460 nm excitation) revealed that photoexcitation of FMN_Q_ leads to formation of FMN_Q_* with Δ*A*_max_ at 360 nm and a ground state bleach feature centered at 460 nm, consistent with previous reports for flavin enzymes (Figure 3A).^22,23^ Independent transient absorption spectra of fluorescein were also consistent with literature (excited state lifetime of 4.1 ns)^33,47^ and showed that substrate **1** was an inefficient quencher of the fluorescein excited state, even taking into account the high concentration of substrate in the reaction (Figure S34).

**Figure 3:**
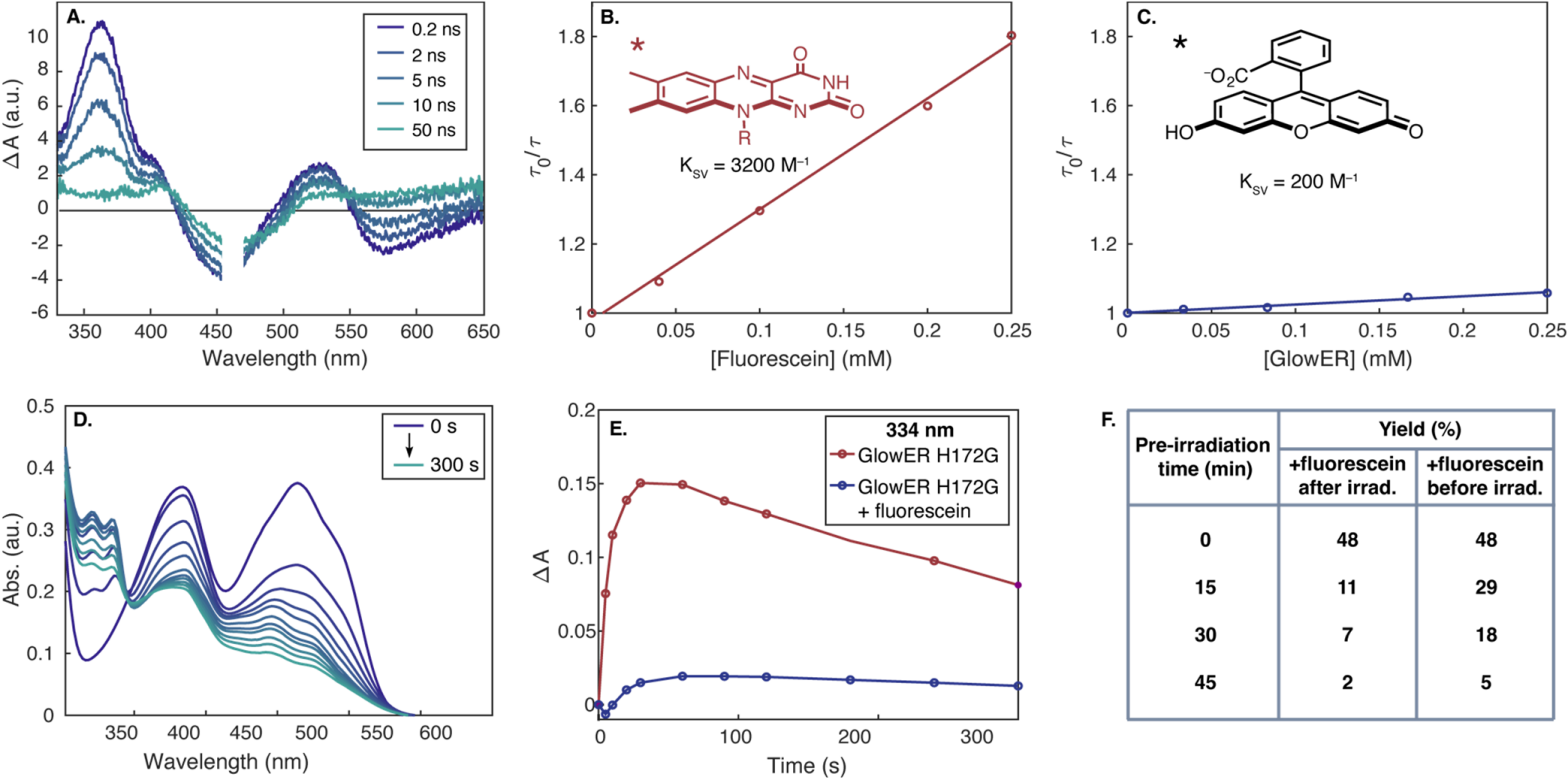
Spectroscopic analysis of interactions between fluorescein and GlowER H172G. (A). Picosecond transient absorption spectrum of GlowER H172G highlighting FMN_Q_* absorbance at 360 nm and the ground state bleaching at 450 nm. (B). Stern–Volmer quenching of photoexcited GlowER H172G FMN_Q_ (0.17 mM) with increasing concentrations of fluorescein. (C). Stern–Volmer quenching of photoexcited fluorescein (0.17 mM) with increasing concentrations of GlowER H172G. (D). Photoreduction kinetics of GlowER H172G (456 nm light). (E). Extracted traces at 334 nm for the photoreduction of FMN_Q_ in GlowER H172G in the absence (red) or presence of fluorescein (blue). (F). Comparative yields for GlowER H172G following 0–45 min of irradiation prior to adding substrate, with and without fluorescein added during the preirradiation. Following preirradiation, the fluorescein concentration was adjusted to 1 mol %, **1** (10 μmol) was added, and irradiation was continued for 16 h.

Next, we analyzed the transient absorption spectra of GlowER H172G with varying amounts (0.2, 0.5, 1, and 1.5 equiv) of fluorescein with excitation at 460 nm. In these spectra, the same excited state absorption features (360 nm for GlowER H172G and 405 nm for fluorescein) are observed, although overlap between the spectra of GlowER and fluorescein make interpretation of the spectral region between 420 and 650 nm challenging (Figures S20–S32). We fit the lifetime of the excited state absorption for GlowER H172G to a single exponential decay extracted from ΔA_350_ and found that in the presence of increasing amounts of fluorescein, the lifetime of the flavin quinone excited state became consistently shorter (Figures 3B and S20–S32), from 6.2 ns alone to 3.5 ns in the presence of 1.5 equiv. fluorescein. The reversed experiment (addition of increasing amounts of GlowER H172G to a fluorescein solution and extraction of the fluorescein excited state lifetime from the single exponential decay of ΔA_405_, corresponding to photoexcited fluorescein, Fl*) revealed that GlowER H172G only minorly affects the lifetime of Fl* (decreasing from 4.1 to 4.0 ns in the presence of 1.5 equiv. GlowER H172G, Figure 3C). We furthermore analyzed the transient absorption spectra of GlowER H172G in the presence of eosin Y and rose bengal (Figure S33) and determined that these photocatalysts are less efficient at quenching FMN_Q_*, consistent with their lower efficiency as cophotocatalysts.

The decreased lifetime of FMN_Q_* in the presence of fluorescein is indicative of a dynamic quenching mechanism that returns the FMN_Q_ excited state to the ground state.^49^ The mechanism of excited state quenching is not clear, as no evidence for energy transfer or electron transfer was observed spectroscopically. An inability to detect products of electron transfer or energy transfer has been encountered in other photocatalytic systems, and results from unfavorable kinetics that prevent accumulation of electron/energy transfer products.^50^ Taken together, however, the changes in the transient absorption spectra of FMN_Q_ in the presence of fluorescein indicate that it serves a photoprotective role, returning FMN_Q_* to the ground state more rapidly than FMN_Q_*’s intrinsic lifetime.

We hypothesized that the decreased lifetime of FMN_Q_* in the presence of fluorescein might prevent reactions of FMN_Q_* that result in degradation of the protein or FMN. To identify potential species arising from this oxidative degradation, we monitored the photoreduction of GlowER H172G by steady-state UV–visible spectroscopy after different irradiation times (Figure 3D). Like all flavin enzymes, GlowER H172G undergoes reduction after extended irradiation with 456 nm light,^45^ producing the flavin hydroquinone after 1 h of irradiation. The photoreduction time course was monitored by acquiring UV–vis spectra at 10 time points between 5 and 300 s of irradiation. Over the course of the reaction, multiple photoreduction intermediates can be identified. The flavin anionic semiquinone forms at short irradiation times as determined by the increased A_380_/A_464_ ratio (Figure S6)^51,52^ and the anionic semiquinone subsequently decays to flavin hydroquinone with prolonged irradiation. At early time points, two peaks with λ_max_ = 322 and 334 nm appear and then slowly decay after 60 s of irradiation (Figure 3D). While we cannot conclusively identify these peaks, they resemble the reported λ_max_ for dialkylated (C4a, N5) flavin derivatives^53^ and the time scale of their appearance appears to match the time scale of decrease in the intensity of FMN_Q_ absorbance. When fluorescein is added to GlowER H172G, both the intensity of these features and the rate of disappearance of FMN_Q_ change. Irradiating GlowER H172G in the presence of 1 equiv. fluorescein results in a decreased rate FMN_Q_ photoreduction (464 nm, Figure S13) and suppression of the formation of the species with λ_max_ = 322 and 334 nm (Figure 4E). Together, these results indicate that fluorescein can reduce the proportion of FMN_Q_ that is converted to the putative dialkylated flavin species; the change in the photoreduction outcomes arises due to the shorter lifetime of FMN_Q_* in the presence of fluorescein (*vide supra*). Though we were unable to definitively determine the species responsible for cofactor alkylation, several protein modifications between GlowER residues 224 and 248 that were only present in samples irradiated with light were detected using protein mass spectrometry (SI p. S106). Regardless of the precise mechanism, suppression of the formation of degraded flavin species provides a mechanism by which fluorescein can protect FMN_Q_ from oxidative deactivation.

**Figure 4:**
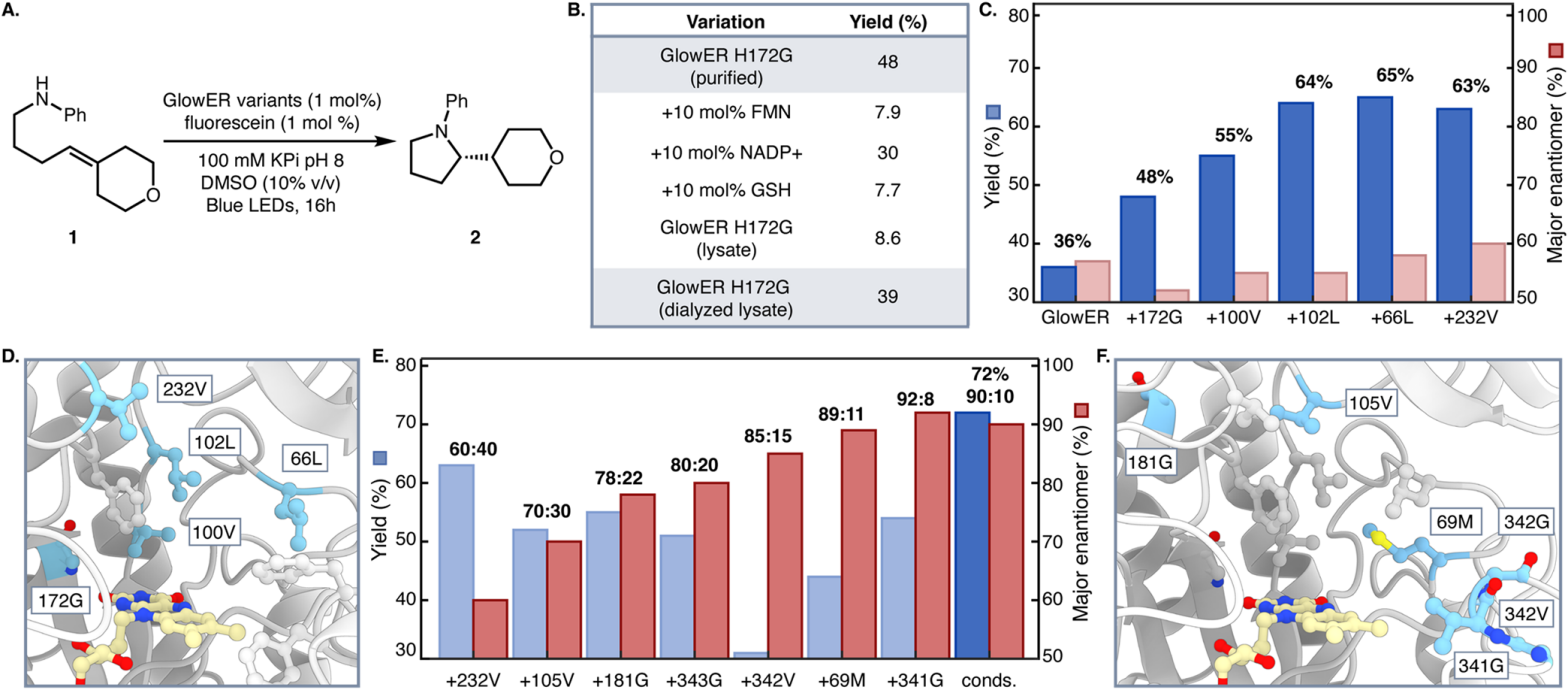
Directed evolution for GlowER-catalyzed hydroamination. (A). Reaction conditions for protein engineering campaign. (B). Identification of inhibitory components of cell lysate and strategies for removal to enable high-throughput analysis. (C). Introduction of five mutations increased hydroamination yield to 63% (blue bars) with 60:40 e.r. (red bars). (D). Mutations introduced through screening variants for increased yield; structure predicted using AlphaFold 3. (E). Introduction of five additional mutations and optimization of reaction conditions with the final variant furnished hydroaminated products in 72% yield (blue bars) with 90:10 e.r. (red bars). (F). Residues introduced through screening variants for increased enantioselectivity; structure predicted using AlphaFold 3.

We last returned to analysis of the hydroamination of **1** to demonstrate that the shorter lifetime of FMN_Q_* in the presence of fluorescein translates to changes in the catalytic efficiency of GlowER H172G (Figure 3F). If GlowER H172G is irradiated with 456 nm light prior to the addition of 1 mol % fluorescein and **1**, the overall yield of **2** is decreased. The extent to which the yield is decreased is dependent on the preirradiation time (Figure 3F), indicating that light is indeed responsible for irreversible deactivation of the enzyme, potentially due to the protein damage detected by mass spectrometry. If 1 mol % fluorescein is added to GlowER H172G prior to irradiation, the reaction yield is partially restored to that observed under standard conditions (Figure 3F). Therefore, fluorescein decreases the extent to which GlowER H172G is deactivated during a given preirradiation time period, consistent with the proposed mechanism in which fluorescein acts as a photoprotectant for GlowER H172G and suggesting that oxidative damage to GlowER H172G is important to limiting the yield of the transformation.

Overall, transient and steady state absorption spectroscopic experiments probing the photochemistry of GlowER H172G in the presence and absence of fluorescein indicate that fluorescein serves as a photoprotectant for FMN_Q_ in GlowER, slowing down deactivation pathways that render GlowER inactive for the hydroamination of **1**. Fluorescein introduces a pathway to return FMN_Q_ to the ground state that competes with deleterious, irreversible, light-driven reactions of FMN_Q_*. This mechanism for photoprotection of FMN_Q_ by fluorescein is distinct from other mechanisms involving cophotocatalysts with flavin enzymes—in our system, fluorescein is not directly involved in conversion of substrate to product but rather modulates the photophysical properties of FMN_Q_. Such photoprotective strategies may prove applicable to other systems, since light-dependent oxidative flavin enzymes such as CvFAP are similarly subject to irradiation-induced deactivation. The half-life for deactivation of GlowER H172G under these irradiation conditions (*t*_1/2_ ∼ 10 min, 456 nm, 2 mmol/(m^2^·s) photons) is comparable to that of CvFAP.^54^ Addition of photoprotective reagents such as fluorescein may provide a general strategy to limit photoinactivation, and further development of cophotocatalyst-driven photoprotective mechanisms for FMN_Q_ will expand the utility of FMN_Q_ in photobiocatalysis.

### Directed Evolution and Reaction Scope

With a mechanism by which fluorescein could lead to increased yields of **2** in hand, we turned to directed evolution to further increase the yield and enantioselectivity of the transformation (Figure 4A).^3,55,56^ However, in our initial mutagenesis studies to identify GlowER H172G, we found that all variants exhibited low activity in clarified cell lysate (<0.1% yield, c.f. 36% for purified GlowER), which could be attributed to small molecules present in the lysate (particularly free FMN and glutathione). Dialysis of the clarified cell lysate restored the reaction yield, consistent with small molecules being the primary inhibitors of catalysis (Figure 4B). To separate these small molecules from the GlowER variants in a manner suitable for high-throughput engineering, we subjected the clarified cell lysate to buffer exchange using size exclusion resin. Addition of this purification step during the evolutionary screening led to an approximately 100-fold increase in enzymatic activity in cell lysate and enabled screening variants in 96-well plate format.

In the reaction catalyzed by GlowER H172G and fluorescein (48% yield), 48% of the starting material **1** was recovered (96% overall mass balance) indicating that the overall rate of catalysis by GlowER H172G was limiting the yield, rather than side reaction pathways for **1**. Therefore, we began our engineering campaign targeting higher yields of **1**. Two rounds of site-saturation mutagenesis^55,56^ introduced the mutations F100V and M102L, which increased the yield of **2** to 55% and 64% with enantioselectivities of 53:47 and 55:45, respectively. Further directed evolution introduced the mutations F66L and Q232V, which did not improve the reaction yields significantly (65% and 63%, respectively) but provided (*S*)-**2** with an increased enantiomeric ratio (58:42 and 62:38, respectively, Figure 4C,D).^57^ We expect the introduction of the highly hydrophobic residues leucine and valine would increase the size of the active site and its affinity for the hydrophobic substrate **1**.

After four rounds of site-saturation mutagenesis, we had obtained a GlowER variant capable of the hydroamination of **1** in good yields (∼65%) yet no variants had been identified with high enantioselectivity. The minor increases in enantioselectivity across the evolution campaign were surprising, since enzymatic yield and enantioselectivity are often correlated in directed evolution campaigns. Nevertheless, to improve the enantioselectivity of the reaction, we switched our engineering approach to screen variants for the enantiomeric ratio of **2** rather than the yield of **2** using reverse-phase chiral high-pressure liquid chromatography, selecting hits for each round based primarily on the enantiomeric ratio of **2**. Returning to our site-saturation mutagenesis libraries using the GlowER H172G/F100V/M102L/F66L/Q232V variant (GlowER R5), we identified the mutation M105V, which increased the enantiomeric ratio of **2** from 60:40 to 70:30, albeit with decreased yields (52%). Continued site-saturation mutagenesis screening for the enantiomeric ratio of **2** introduced the mutations E181G, F343G, F342V, and A69M, which provided (*S*)-**2** with an e.r. of 78:22, 80:20, 85:15, and 89:11, respectively. However, introduction of these mutations, particularly F342 V, lead to a decreased yield of **2** (44% after round 9). Therefore, we carried out a final round of mutagenesis, returning to our initial engineering strategy screening for increased yield of **2**. We were able to identify the mutation T341G, which increased the yield to 54% with 92:8 e.r. Following reoptimization of the reaction conditions using this final variant (GlowER H172G/F100V/M102L/F66L/Q232V/M105V/E181G/F343G/F342V/A69M/T341G; termed GlowHA hereafter, Figure 4F)^57^ we were able to prepare (*S*)-**2** in 72% yield with an enantiomeric ratio of 90:10 (19% recovered **1**, 91% overall mass balance). Ultrafast and steady state UV–vis spectroscopic studies of GlowHA were similar to those for GlowER H172G (see SI), indicating that evolution had not significantly changed the mechanism of interactions with fluorescein. Moreover, the melting temperature of GlowHA (*T*_m_ = 63.7 °C) was very similar to that of GlowER H172G (Figure S40), indicating mutagenesis had not significantly perturbed the enzyme’s thermostability.

We next turned to exploring the scope of the GlowHA catalyzed hydroamination reaction (Figure 5). Evaluating the tolerance of the reaction to substituents on the aniline moiety showed the enantioselectivity generally remained high (ca. 90:10) with a range of electron donating and withdrawing substituents on the aniline. Substitution at the 4-position generally decreased the yield (**3**, 9%, 93:7 e.r.; **4**, 20%, 91:9 e.r.; **5**, 10%, 89:11 e.r.; **6**, 6%, 79:21 e.r.), while substitution at the 3-position was better tolerated (**7**, 25% 90:10; **8**, 25%, 86:14; **9**, 35%, 81:19, **10**, 34%, 90:10). Additionally, electron withdrawing substituents (**4**, **5**, **6**, **8**, and **9**) were better tolerated than electron donating substrates; this can be attributed to the less-stabilized electron-deficient aminium radical cations undergoing more rapid C–N bond formation than their electron-rich counterparts. Conversely, the enantioselectivity is generally lower for electron-deficient anilines, suggesting that the more rapid C–N bond formation for these substrates leads to less efficient enantioinduction due to less efficient equilibration between the two pro-chiral conformers of the aminium radical cation (*vide infra)*. Nitrogen-containing heterocycles on the aniline were tolerated in modest yields and enantioselectivities (**11**, 22%, 76:24 e.r. and **12**, 13%, 91:9).

**Figure 5:**
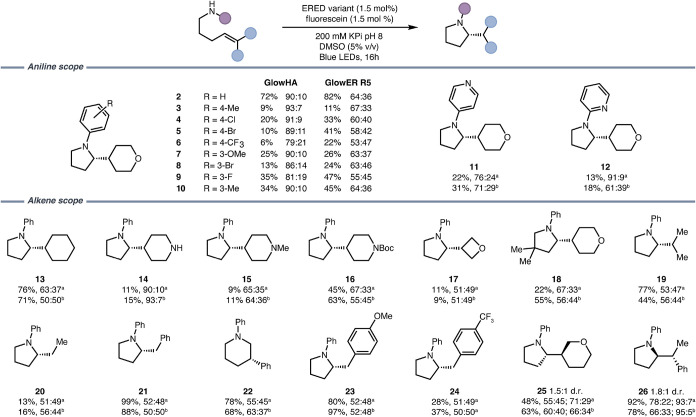
Scope of GlowER-variant catalyzed hydroamination reaction, evaluated with both GlowHA (a) and GlowER R5 (b).

Alkenes that were suitable for hydroamination included trisubstituted alkenes with different substituents in the six-membered ring including a cyclohexyl ring (**13**, 76% yield, 63:37 e.r.), and piperidines with varied N-substituents (**14**, 11%, 90:10 e.r.; **15**, 9%, 65:35; **16**, 45% yield, 67:33). Substrates containing strained cyclic substituents such as oxetanes afforded the hydroamination product in low yields (**17**, 11%, 51:49). Strikingly, replacement of the pyran with other cyclic substituents except for unprotected piperidine (**13**, **14**, **15**, **16**) led to a general erosion of the enantioselectivity, particularly using GlowHA as the catalyst. We hypothesize that specific interactions with the oxygen atom of the pyran are important for positioning the prochiral face of the olefin for enantioselective C–N bond formation. Consistent with this hypothesis, acyclic trisubstituted alkenes furnish hydroaminated products in good yield but with ablated enantioselectivity (**19**, 77% yield, 53:47 e.r.). Disubstituted alkenes, which form less stabilized alkyl radicals following cyclization, underwent hydroamination in modest yields (**20**, 13%, 51:49 e.r.) that would provide a suitable starting point for further engineering, demonstrating that this methodology does not require formation of a stabilized tertiary or benzylic radical following cyclization. Styrenyl alkenes (**21, 23**, **24**), which form a highly stabilized benzylic radical following cyclization, afforded the hydroaminated product in high yields (99%, 80%, and 28%, respectively) albeit with no enantioselectivity (52:48, 52:48, and 51:49, respectively). Modifications to the pyrrolidine ring were also tolerated (**18**, 22% yield 67:33 e.r.). Substrates primed for 6-endo instead of 5-exo cyclization could be hydroaminated in good yields (**22**, 78%, 55:45 e.r), enabling formation of piperidines in addition to pyrrolidines. Lastly, substrates containing an asymmetric trisubstituted alkene (**25** and **26**) could be hydroaminated in good yield (48% and 92%, respectively), albeit with modest stereocontrol (1.5:1 d.r. and 1.8:1 d.r., respectively). Futher directed evolution using these substrates could be used to improve the diastereomeric ratio of these products.

Since hydroamination of most substrates proceeded in lower yields than for the model substrate, we hypothesized that protein engineering for enantioselectivity could have increased the enzyme’s substrate specificity. Therefore, we additionally evaluated the scope of the transformation using GlowER H172G/F100V/M102L/F66L/Q232V (GlowER R5), which was the final variant constructed before beginning screening protein libraries for enantioselectivity. Indeed, the yields for the hydroaminated products **3**–**11** are generally higher (33% on average for GlowER R5 19% for GlowHA), although the products are formed with lower enantioselectivity (ca. 60:40 on average). We expect that enantioselectivities could be readily improved for particular substrates of interest by further screening protein variant libraries for improved enantiomeric ratios, using either GlowHA or GlowER R5 as a high-yielding starting point.

### Mechanism of Enantioselective Hydroamination

At the outset of our mechanistic investigation, we wanted to understand how GlowHA was able to overcome the challenge faced by small molecule catalysts for hydroamination of unactivated alkenes by anilines,^21^ that C–N bond formation is slow compared to back-electron transfer to the photocatalyst (BET). Additionally, we wanted to understand if the hydroamination mechanism was related to the difficulty in obtaining a simultaneously highly enantioselective and high-yielding enzyme variant for this reaction. We first carried out kinetic analysis of the hydroamination of **2**. Michaelis–Menten kinetics showed *V*_max_ = 1.11 μM/s, *K*_m_ = 780 μM, and *k*_cat_ = 0.44 min^–1^ (Figure S36). The value of *k*_cat_ for GlowER is very similar to that previously reported for a reductive photocatalytic reaction using GluER, while *K*_m_ is approximately 10-fold lower.^6^ In addition, initial rate analysis for GlowER H172G, GlowER R5, and GlowHA revealed that engineering the enzyme for improved enantioselectivity has led to an overall lower initial rate: the initial rate for GlowER H172G and GlowER R5 were 0.84(7) and 0.88(6) nmol/min, respectively, while the initial rate for GlowHA was 0.56(7) nmol/min (Figure S37). That engineering for enantioselectivity would decrease the overall rate of catalysis is unusual for engineered enzymes, as proteins are typically thought to stabilize a single enantiomer of the transition state, leading to simultaneous improvements in rate and enantioselectivity through engineering. Therefore, we further investigated the mechanism of enantiodetermination in this reaction through deuterium labeling studies.

Our final proposed mechanism of enantioselective hydroamination by GlowHA (Figure 6A) is closely related to proposed mechanisms for radical hydroamination with small molecule catalysts: following substrate binding to GlowHA, FMN_Q_ could undergo photoexcitation prompting to electron transfer from **1** to FMN_Q_ to generate an aminium radical cation (**1-rad**) and FMN_SQ_^–^. **1-rad** could then undergo addition of the aminium radical cation to the alkene to form a cyclized radical intermediate ((*S*)-**2-rad**). The enzyme accelerates C–N bond formation compared to **1-rad** in solution by stabilizing substrate conformations that can undergo rapid C–N bond formation, overcoming kinetic competition from BET. Lastly, (*S*)-**2-rad** could be terminated by proton and electron transfer, likely via coupled electron transfer from FMN_SQ_^–^ and proton transfer from the solvent, but potentially via protonation of FMN_SQ_^–^ to FMN_SQ_H followed by hydrogen atom transfer, to form (*S*)-**2**.

**Figure 6:**
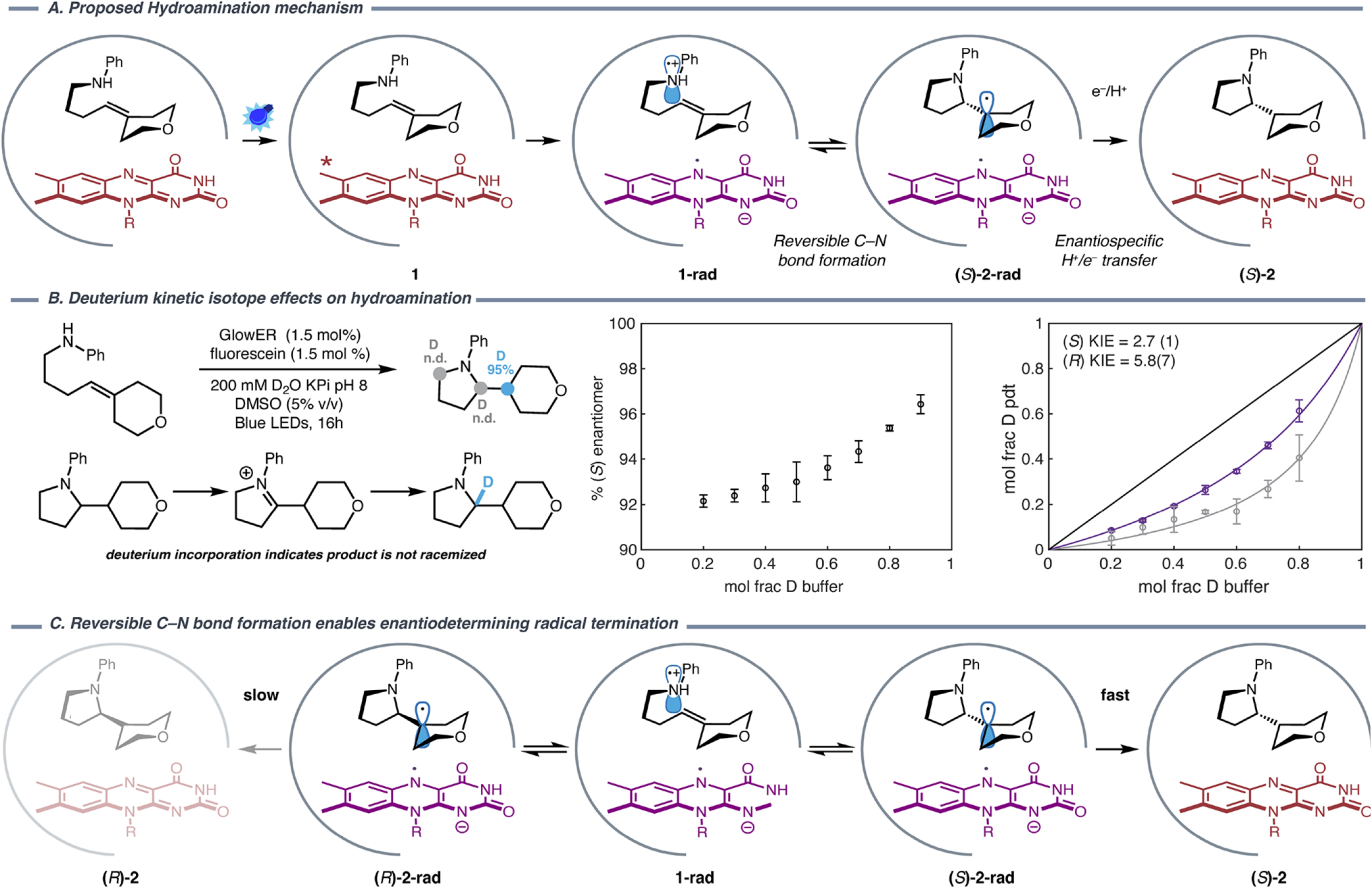
(A). Proposed mechanism for oxidative hydroamination. (B). Deuterium from the buffer is exclusively incorporated β to the aniline, consistent with the mechanism in 6A and ruling out racemization of **2** via ablation of the stereocenter. Isotope incorporation experiments reveal deuterated buffer increases the enantioselectivity of the hydroamination; increased e.r. arises due to different KIEs for quenching of each enantiomer of **2-rad**. (C). Reversible C–N bond formation is required to interconvert the two enantiomers of the cyclized radical. Fast hydrogen transfer to the major enantiomer is the origin of enantioselectivity.

At the outset of our analysis, we hypothesized that stereoselective C–N bond formation could be achieved by pro-(*S*) conformations of **1** in the active site having either a higher binding affinity or higher quantum yield for electron transfer compared to pro-(*R*) conformations. Both higher binding affinity for a specific substrate conformer and higher quantum yield for electron transfer have been previously proposed as sources of enantioinduction in photobiocatalysis.^38,58–61^ However, we discovered that stereoinduction in the formation of **2** arises from a distinct mechanism, whereby rapid and enantiospecific hydrogen transfer to a single enantiomer of **2-rad** controls the overall stereochemical outcome, rather than being dictated by the initial binding conformation.

Before elucidating this mechanism, we considered the possibility that low enantioselectivity could arise from a competing reaction pathway that would racemize the product: oxidation of **2** to an aminium radical cation would acidify the α-C–H bonds, enabling deprotonation to ablate the stereocenter in **2** (Figure 6B).^62^ Nonstereoselective hydrogen transfer would then racemize **2**. To determine racemization of **2** was occurring, we carried out a deuterium labeling study by conducting the hydroamination reaction in D_2_O buffer. The hydroamination of **1** in D_2_O buffer furnishes **2** with a deuterium atom at the β-position relative to the aniline, consistent with the proposed hydrogen transfer step to quench the alkyl radical after hydroamination (Figure 6A). In contrast, no deuterium incorporation was observed at the α-positions of **2**, indicating racemization of **2** is not a significant contributor to the overall enantioselectivity of the transformation.

To our surprise, during these studies we found a significant KIE (*k*_H_/*k*_D_ = 2.2(4)) for the formation of **2**. A primary KIE for this reaction is inconsistent with the rate-limiting C–N bond formation that has been observed with small molecule catalysts,^21^ which would be expected to have no more than secondary KIE (*k*_H_/*k*_D_ < 1.5). The observation of a primary KIE suggested that transfer of a proton—likely in termination of the cyclized radical **2-rad**—was the rate-determining step in this transformation. Radical termination as the rate-determining step is different from the rate-limiting C–N bond formation proposed for the hydroamination of unactivated alkenes with amines by small molecule catalysts^18,21^ and suggests that the enzyme indeed accelerates C–N bond formation compared to free **1-rad** in solution. This acceleration enables productive catalysis by favoring C–N bond formation over BET.

More importantly, the enantiomeric ratio of **2** for the reaction carried out in D_2_O buffer was significantly higher (96:4) than for the reaction carried out in H_2_O buffer (90:10). The higher enantioselectivity in deuterated solvent was unexpected, since the stereocenter in **2** is established in the previous, C–N bond forming step before partitioning of the product to incorporate deuterium or hydrogen from the buffer. The increased enantioselectivity in D_2_O was found to be general to variants across the engineering campaign (Table S4).

Two mechanisms can be proposed that lead to a change in enantioselectivity upon conducting the hydroamination in deuterated buffer.^63^ The first is an equilibrium isotope effect that perturbs the binding of pro-(*R*) and pro-(*S*) conformers within the active site, favoring pro-(*S*) conformers. The second possible mechanism involved a significant difference in the KIEs for hydrogen vs deuterium transfer to the enantiomers of the cyclized radical intermediates, *(R)-* **2**-**rad** and *(S)-* **2**-**rad**. Coupled with a racemization mechanism to interconvert *(R)-* **2**-**rad** and *(S)-* **2**-**rad**, the different KIEs for each enantiomer would result in a change to the overall enantioselectivity.

These two mechanisms can be differentiated in two ways using the individual, H/D competition isotope effects for each enantiomer of **2**: (1) An equilibrium isotope effect would be expected to have a magnitude between 0.8–1.2 for hydrogen bonds between typical neutral or cationic O/N atoms, while a primary KIE would be between 2 and 7, and (2) For a kinetic isotope effect, the KIEs for hydrogen transfer to *(R)-* **2**-**rad** (KIE_R_) and *(S)-* **2**-**rad** (KIE_S_) must be sufficiently different to explain the magnitude change in e.r. We can derive the following relationship (eq 1, see SI p. 76)^64,65^ for the ratio KIE_R_/KIE_S_ required to explain the observed change in e.r. (90:10 in H_2_O and 96:4 in D_2_O) and find that KIE_R_/KIE_S_ = 2.67.

1 $$\frac{{e.r.}_{D}}{{e.r}_{H}}=\frac{{\mathrm{KIE}}_{R}}{{\mathrm{KIE}}_{S}}$$

The above equation requires that the rate constants that determine the KIE (for hydrogen transfer to **2**-**rad**) are the same as the ones that determine the enantiomeric preference (*k*_R_ and *k*_*S*_). Therefore, if the measured competitive KIEs for *(R)-* **2** and *(S)-* **2** are sufficient to explain the change in enantioselectivity, this relationship provides support for hydrogen transfer as the overall rate-determining step in this transformation.

To measure the individual KIEs for each enantiomer, we carried out competitive H/D KIE experiments in seven solvent compositions ranging from 10% D_2_O to 80% D_2_O. Looking first at the overall enantioselectivity, we find that the e.r. increases nonlinearly with solvent composition, favoring enantioselectivities close to 90:10 until the reaction buffer approaches 80% D_2_O (Figure 6B). Using HPLC-MS to detect the ratio of isotopes incorporated into the product, we additionally see a clear bias toward incorporation of a proton into the product over a deuterium (Figure S1). Both observations are consistent with a primary competitive kinetic isotope effect, favoring H transfer over D transfer to **2-rad**.

To measure the KIEs for each enantiomer independently, we turned to reverse-phase chiral HPLC-MS to detect the isotopic ratios in each enantiomer (Figure 4B). The extent of deuterium incorporation in the major enantiomer (*S*)**-2** is higher than in the minor enantiomer (*R*)**-2**, consistent with a smaller isotope effect for (*S*)**-2** and the observation that the enantioselectivity is higher in D_2_O than in H_2_O. The KIEs can be determined quantitatively by fitting the isotope distribution of the products.^64,65^ From this analysis, we obtain KIEs of 2.7(1) and 5.8(7) for (*S*)**-2** and *(R*)**-2**, respectively. Isotope effects >2 are large enough to be unlikely to arise from an equilibrium isotope effect, suggesting that the change in enantioselectivity in D_2_O is the result of different kinetic isotope effects for enantiospecific hydrogen transfer to radical intermediates (*S*)**-2-rad** and *(R*)**-2-rad**. From eq 1, we obtain a ratio of KIE_R_/KIE_S_ = 2.2(4), which is in good agreement with the ratio required to explain the overall change in enantioselectivity (2.67).

From the above analysis, we can conclude that the stereoselectivity of GlowHA arises from differences in the rate of enantiospecific hydrogen transfer to each enantiomer of **2**-**rad**, rather than to stereoselective C–N bond formation. Since the stereocenter has already been formed in **2-rad**, enantiodetermining hydrogen transfer requires a mechanism to racemize the stereocenter set by formation of the C–N bond. We propose that C–N bond formation occurs reversibly to enable the racemization of **2-rad** (Figure 6C). In sum, enantioselectivity in GlowHA is achieved through Curtin-Hammett control of the cyclized radical intermediate **2-rad**, which can undergo rapid equilibration between (*S*)**-2-rad** and *(R*)**-2-rad**. Enantiospecific hydrogen transfer to the cyclized radical is the selectivity determining step for the overall reaction.

The mechanism of enantiodetermination described above—Curtin-Hammett control over enantiospecific hydrogen transfer to a radical intermediate—is distinct from previously reported mechanisms of enantioinduction in photobiocatalysis. Established mechanisms for enantiodetermination in photoenzymes rely on selective positioning of the substrate(s) in a prochiral conformation within the active site; in these examples stereodetermination occurs prior to photoexcitation and generation of high-energy intermediates.^38,58–61^ Here, the enantiodetermining occurs well after photoexcitation and instead relies on the ability of the enzyme to control the differential rates of hydrogen transfer to each enantiomer of a chiral intermediate, rather than by establishing direct selectivity over stereocenter formation. The ability of the enzyme to mediate enantiospecific hydrogen transfer to a single enantiomer of a transient radical intermediate highlights the utility of enzymes in precisely controlling reactive intermediates in catalysis.

## Conclusions

Here, we have demonstrated the use of rational mutagenesis to tune the photophysical properties of flavin-containing proteins. Enzymes that have increased FMN_Q_ excited state lifetimes can catalyze new, non-native oxidative radical chemistry in low yields; the activity of these catalysts can be further improved using established directed evolution strategies. This strategy enables oxidatively initiated hydroaminations to be catalyzed by flavin-dependent “ene” reductases, enabling us to take advantage of the favorable practical (solubility, stability, high expression, etc.) properties of these enzymes while overcoming long-standing limitations on the types of mechanisms accessible to this enzyme class.

Access to oxidative transformations enabled elucidation of unique mechanistic features which may be applied to further development of oxidative transformations: (1) Co-photocatalysts can act as photoprotectants for the FMN_Q_ cofactor, limiting deleterious deactivation mechanisms to extend catalyst lifetime and (2) The enantiodetermining step in the photobiocatalytic hydroamination reaction is enantiospecific transfer of a hydrogen atom to a formally nonstereogenic carbon center, following reversible C–N bond formation that establishes the stereocenter. Enantiospecific hydrogen transfer takes place under Curtin-Hammett control, highlighting the ability of enzyme active sites to achieve kinetic control over high-energy intermediates. Related dynamic kinetic resolutions of radical intermediates may be useful for enantioselective formation of other congested stereocenters in engineered enzymes. These mechanisms are complementary to other modes of photobiocatalysis and may prove to be generalizable to other oxidative transformations in flavin enzymes. The enzyme variants and mechanistic strategies introduced in this work will enable future oxidative photobiocatalysis in flavin containing enzymes with diverse structures and native functionalities.

## Supplementary Material



## Notes

Experimental procedures; supporting data tables; supporting spectroscopic data; NMR data; and HPLC data (PDF)
