# Supplementary material for: Asymmetric Hydroamination Using Oxidative Radical Initiation in Flavin Enzymes
Source: J Am Chem Soc. 2026 Jun 12;148(25):25945–56. doi: 10.1021/jacs.6c04368 (PMC13339135; doi:10.1021/jacs.6c04368)
Supplement: Supplementary file 1 [file ja6c04368_si_001.pdf]

Asymmetric Hydroamination Using Oxidative Radical Initiation in Flavin Enzymes

*Alexandra C. Brown, Carlos E. Del Angel Aguilar, Felix C. Raps, Paul S. Riehl, and Todd K. Hyster**

Department of Chemistry, Princeton University, Princeton, New Jersey, 08544, United States

Supporting Information

A. General details and Instrumentation S2-S3
B. Enzymatic reaction methods S4
C. Protein engineering methods........ S5
D. Reaction optimization S6–S7
E. Substrate synthesis and characterization........ S8–S41
F. Product standard synthesis and characterization........ S42–S71
G. Protein and DNA sequences S72–S80
H. Deuterium incorporation........ S80–S82
I. UV-vis spectroscopic details........ S83–S91
J. Ultrafast spectroscopic details S92–S99
K. Biochemical characterization of GlowER H172G and Glow HA S100–S105
L. Protein digest mass spectrometry........ S106–S109
M. Chiral HPLC traces S110–S134
N. NMR spectra S135–S184
O. References........ S185

A. General Details and Instrumentation

Reagents and solvents were purchased from commercial vendors and used as received. Silica gel purifications were performed with AMD Silica Gel 60. NMR spectra were collected on either a Bruker NanoBay 300 MHz, NanoBay 400MHz, or Avance III 500 MHz. ^{1}H and ^{13}C resonances are reported in reference to the residual solvent peak and the observed multiplicity (s= singlet, d= doublet, t= triplet, q = quartet, quin.= quintet, m= multiplet). Coupling constants are given in Hz. Infrared spectra were recorded on an Agilent Cary 630 FTIR and reported according to relative intensity (w= weak, m= medium, s= strong, br= broad) and wavenumber (cm^{-1}).

High pressure liquid chromatography (HPLC) and low-resolution Electron Spray Ionization (ESI) mass spectrometry were carried out using an Agilent 1260 LCMS System. Yields were determined using a Waters XSelect Premier HSST3 column (4.6 x 100 mm, 2.5 µm) against an internal standard 1,3,5-tribromobenzene (TBB) at 210 nm with water (0.1 % formic acid) and acetonitrile (0.1% formic acid) as the mobile phase. Normal-phase chiral HPLC was carried out using an Agilent 1260 Infinity instrument equipped with ChiralPak® AS-H, AD, OD, IA, IB, IC, ID, IE, IG, IF, IH, and ChiralCel® OJ-H, (4.6 x 250 mm, 5 µm) columns with isopropanol and hexanes as the mobile phase. Reverse phase chiral HPLC. Site-saturation libraries were screened using a Shimadzu LC-2060D LC equipped with a Waters XSelect Premier HSST3 column (4.6 x 100 mm, 2.5 µm, yield analysis) or ChiralPak® IC column (4.6 x 250 mm, 5 µm, enantioselectivity analysis)

GlowER variants were produced and purified according to protocols reported for GluER,[1] except the purification buffers were as follows:
Buffer A, low-imidazole: (300 mM NaCl, 100 mM KP_i, 50 mM imidazole, pH 8)
Buffer B, high-imidazole: (300 mM NaCl, 100 mM KP_i, 250 mM imidazole, pH 8)
Buffer C, imidazole free, concentration and storage: (200 mM KP_i, pH 8)
Media was supplemented with kanamycin rather than ampicillin for all GlowER variants (in pET-28(a)+ vectors).

UV-visible spectra and fluorescence spectra were collected on samples in quartz cuvettes with a 1 cm path length. Spectra were recorded using a Duetta Fluorescence and Absorbance Spectrometer from Horiba Scientific.

Transient absorption spectra were collected on a commercial Ultrafast Systems Transient Absorption Helios Spectrometer. For femtosecond timescale experiments samples were excited with a 300 nJ pulse centered at 460nm, generated using a Coherent Astrella Titanium : Sapphire Amplifier (45 fs, 800nm,1 kHz, 2.5W x 2). The internally compressed pulse is steered into a Coherent OPerA Solo computer controlled optical parametric amplifier which generated 460 nm excitation pulses. For picosecond timescale experiments, samples were excited with a 300 nJ pulse centered at 460nm generated with Ekspla PT403 tunable picosecond laser. The electronic delay between the two pulses is calculated using a commercial setup design by Ultrafast Systems. In both cases samples were probed using a white-light supercontinuum spectrum generated by focusing 800 nm light from a Coherent Astrella (45fs, two 2.5mJ outputs) in a continuously vertically translating calcium fluoride crystal. Samples were run in sealed 2mm quartz cuvettes. To minimize sample degradation the solution is continually stirred and translated during the experiment.

Melting temperatures were determined via Differential Scanning Calorimetry (DSC) using a MicroCal PEAQ-DSC (Malvern Instruments Ltd., Malvern, UK). Measurements were taken in 200 mM KPi pH 8.0 buffer, from 25°C to 100°C at a rate of 3°C per minute. Protein samples were measured at a concentration of 0.1 mM (approximately 4 mg/mL).

Photocatalytic reactions were carried out in 1 mL vials in a 96-Well Photoredox Block Assembly by Lumidox on a Lumidox II 470 nm 96-well plate with a lens mat (library screening, top) or in 4 mL shell vials using a 456 nm Kessil PR160L light at 25% intensity (10 µmol scale reactions, bottom).

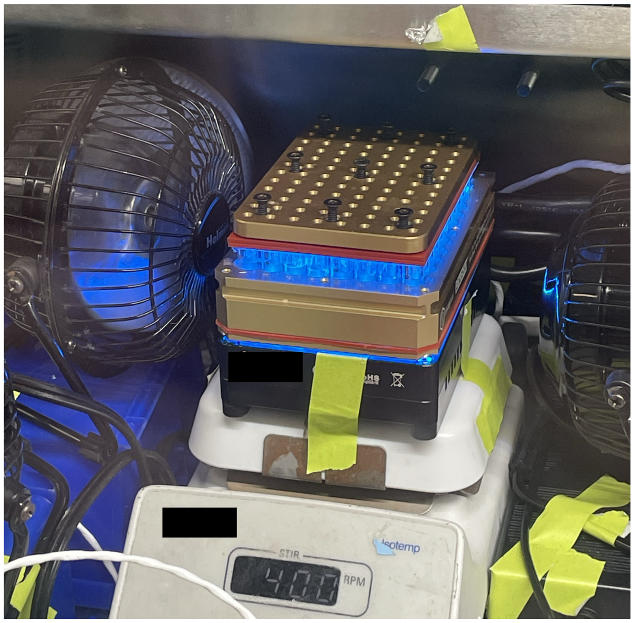

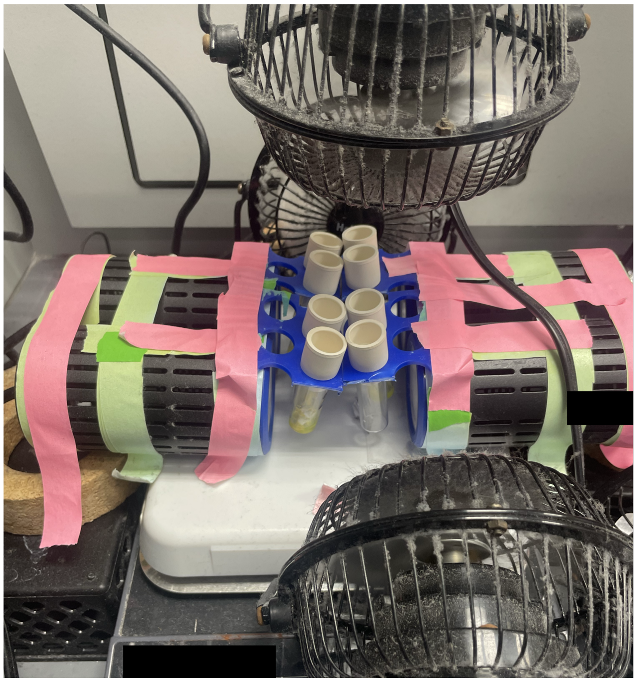

B. General Procedure for evaluation of enzymatic oxidative hydroamination.

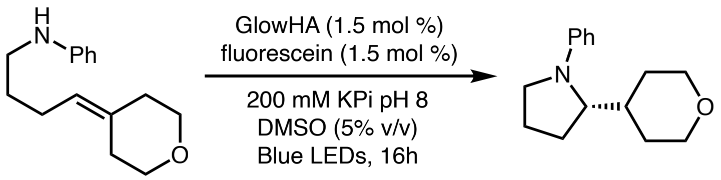

All reactions were prepared inside of a dinitrogen filled glovebox (<0.1 ppm O_2). To a 4 mL dram vial charged with a stir bar were added KP_i buffer (200 mM, pH 8, 1800 uL), substrate (10 µmol, dissolved in 100 µL DMSO), and GlowHA (150 nmol, in 100 µL of 200 mM KP_i pH 8). The vial was sealed with a septum and irradiated (456 nm) for 16 h. Following irradiation, the vial was opened to the atmosphere and 3 mL of MeCN were added. 1,3,5-tribromobenzene (2.5 µmol) was added to the solution as an internal standard and the reaction yield was assayed by LC-MS using a product standard curve. The remaining solution was evaporated under vacuum and the solids extracted with hexanes for chiral HPLC analysis.

C. Protein engineering methods

DNA libraries for site-saturation mutagenesis were prepared according to primers designed using the 22-codon trick.[2] Following PCR amplification, the DNA was subjected to DPN1 digestion, purified using NEB Monarch purification kits and circularized using NEB HIFI Assembly. Plasmids were transformed into electrocompetent *E. coli* BL21(DE3) cells. Sterile toothpicks were used to inoculate single wells of a 96 well-plate containing terrific broth (500 µL/well) and kanamycin (50 mg/mL) in 96 deep-well plates. The parent variant was inoculated into wells A1, B2, C4, D6, E8, F10, G11 as a control. Well H12 was left blank as a contamination control. The plate was incubated at 37 °C and 250 rpm for 16 h. A glycerol stock of the plate was prepared by mixing overnight culture (50 µL) with 50% glycerol (50 µL/well) in a separate 96 clear-well plate. The glycerol plate was sealed and stored at –80 °C. Separately, overnight culture (50 µL/well) was added to expression medium consisting of terrific broth (950 µL/well) with auto-inducing mix (glucose (1 w/w%), lactose (4 w/w%) and glycerol (15 w/w%) in sterile water; 40 mL/L) and kanamycin (50 mg/L) in a 96 deep-well plate. The plate was incubated at 30 °C and shaken at 250 rpm for 24 h. Cells were harvested by centrifugation (5000 g, 10 min) and stored at –20 °C prior to use.

Before reaction setup, the site-saturation plate was thawed for 30 min in room temperature water. To each well was added lysis buffer (200 mM KP_i pH 8, 100 µL/well) containing FMN (0.1 mg/mL), lysozyme (1.0 mg/mL), DNAse (0.05 mg/mL) and PMSF (35 µg/mL). The lowered FMN concentration compared to prior reports[3] (from 1mg/mL to 0.1 mg/mL) was necessary to enable removal of unbound FMN by size exclusion chromatography. The plate was shaken at 37 °C for 2 h. After 2 h, the plate was centrifuged (2500 g, 10 min) to precipitate cell debris. To a Zeba 96-well plate containing 7k MWCO size exclusion resin (Thermo Scientific) equilibrated with 200 mM KP_i pH 8 was added clarified cell lysate (95 µL /well) from the site saturation plate. The desalted lysate was eluted by centrifugation (1000 g, 30 sec) and transferred to a Lumidox 96-well photoredox assembly. The plate was brought into the glovebox and a stock solution containing substrate (100 mM) and fluorescein (1 mM) in DMSO was added (10 µL/well). The plate was sealed and stirred with blue light irradiation (470 nm) for 16 h inside of an MBraun glovebox. After the reaction the plate was removed from the glovebox, the reactions were quenched by addition of MeCN (600 µL/well), and the mixtures were stirred for 20 min at room temperature. Precipitates were removed by centrifugation (2500 g, 10 min) and aliquots from each well (200 µL) were transferred to a clean 96-well plate for HPLC analysis.

D. Reaction optimization

Table S1: Screen of EREDs from in-house library for hydroamination of **1** (10 µmol **1**, 1 mol% enzyme, 100 mM KP_i pH 8, 10% v/v DMSO, 1 mL. Irradiated with 456 nm light for 16 h).

Enzyme	Yield of 2 (%)
GluER T36A	n.d.
GkOYE	n.d
OYE1	0.7
OYE3	n.d.
PpER	n.d.
CsER	n.d.
MorB	1.2
YqjM	n.d.
XenA	0.1
FlOYE	0.3
LacER	0.2
BpER	n.d.
YersER	0.4
OPR1	0.7
GlowER	4.5
GlowER H172G	8.9

Table S2: Photocatalyst screen for hydroamination of **1** with GlowER H172G (10 µmol **1**, 1 mol% enzyme, 100 mM KP_i pH 8, 10% v/v DMSO, 1 mL) 1 mol% photocatalyst added. Irradiated with 456 nm light for 16 h.

Photocatalyst	Yield of 2 (%)
None	8.9
Fluorescein	48
Eosin Y	37
Rose Bengal	21
Rhodamine 6G	9.4
Rhodamine B	7.1
$Ru(bpy)_3Cl_2$	1.8
$[Ir(dtbbpy)_2(ppy)][PF_6]$	2.3

Table S3: Wavelength dependence of photocatalyst efficiency. Analysis of hydroamination of **1** with GlowER H172G (10 µmol **1**, 1 mol% enzyme, 100 mM KP_i pH 8, 10% v/v DMSO, 1 mL) 1 mol% co-photocatalyst added. Irradiated for 16 h.

Wavelength (nm)	Photocatalyst	Yield of 2 (%)
456	Fluorescein	52
	Eosin Y	35
	Rose Bengal	25
505	Fluorescein	34
	Eosin Y	33
	Rose Bengal	25
525	Fluorescein	29
	Eosin Y	26
	Rose Bengal	21
390	Fluorescein	20
	Eosin Y	7
	Rose Bengal	4

Table S4: Effect of adding free FMN to the hydroamination of **1** by Glow HA. (10 µmol **1**, 1 mol% GlowHA, 100 mM KP_i pH 8, 10% v/v DMSO, 1 mL) 1 mol% fluorescein added. Irradiated for 16 h.

GlowHA	Added free FMN	Yield of 2 (%)
1 mol%	0 nmol	62
	20 nmol	60
	40 nmol	61
	60 nmol	62
	80 nmol	63
	100 nmol	60

E. Substrate synthesis

General Procedures. Characterization of intermediates is reported following the respective procedure. The reaction sequence used for each substrate and characterization data for each substrate is reported beginning on page S16.

Procedure A: Grignard addition to carbonyl
Ketone (1 equiv, 90 mmol) was dissolved in THF (200 mL) and the solution was cooled to 0 °C. A solution of vinylmagnesium bromide (100 mL, 1 M in THF, 1.1 equiv.) was added and the mixture was stirred overnight at room temperature. After 16h, 200 mL of saturated NH_4Cl in water were added and the mixture was extracted with EtOAc (3 x 100 mL). The combined organic layers were dried over Na_2SO_4 and evaporated to dryness. The crude alcohol was purified by silica column chromatography.

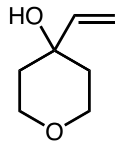

S1a: From 4-oxotetrahydropyran. Purified by silica gel column chromatography (0-100% EtOAc/hexanes). ^1H NMR (300 MHz, $CDCl_3$) δ 5.99 (dd, *J* = 17.4, 10.7 Hz, 1H), 5.29 (dd, *J* = 17.4, 0.8 Hz, 1H), 5.13 (dd, *J* = 10.7, 0.8 Hz, 1H), 3.96 – 3.68 (m, 5H), 1.94 – 1.76 (m, 2H), 1.70 (s, 1H), 1.63 – 1.40 (m, 3H).

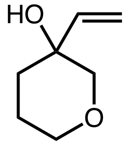

S25a: From 3-oxotetrahydropyran. Purified by silica gel column chromatography (0-100% EtOAc/hexanes). ^1H NMR (500 MHz, $CDCl_3$) δ 5.88 (dd, *J* = 17.4, 10.9 Hz, 1H), 5.40 (d, *J* = 17.4 Hz, 1H), 5.20 (d, *J* = 10.9 Hz, 1H), 3.91 (d, *J* = 11.3 Hz, 2H), 3.60 – 3.22 (m, 2H), 1.96 (m, 2H), 1.74 (m, 2H).

Procedure B: Johnson-Claisen rearrangement
Allyl alcohol (1 equiv, 50 mmol) was dissolved in triethylorthoacetate (50 mL). Propanoic acid (1 mL) was added and the mixture was heated to reflux for 16 h or until TLC analysis indicated complete conversion of the alcohol. The solvent was removed *in vacuo* and the crude ester was directly subjected to hydrolysis (procedure C) without further purification.

Procedure C: Ester hydrolysis
Crude ester (1 mmol, 1 equiv.) was dissolved in a 1:1:1 mixture of THF/MeOH/H_2O (3 mL). LiOH·H_2O (3 equiv.) was added and the suspension was stirred until TLC analysis indicated complete consumption of the ester (approx. 3h). The solution was diluted with water (100 mL) and the aqueous layer was washed with DCM (3 x 10 mL). The pH of the aqueous layer was adjusted to 1 with 1 M HCl and the aqueous layer was extracted with EtOAc (3 x 50 mL). The organic layer was dried over Na_2SO_4 and evaporated to dryness. The carboxylic acid was typically sufficiently pure to be employed directly in subsequent steps following this acid/base purification protocol.

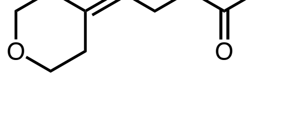

S1c: From **S1a** by procedure B and C. Yield 2.3 g (74%) ^1H NMR (500 MHz, $CDCl_3$) δ 5.20 (t, *J* = 7.0 Hz, 1H), 3.68 (dt, *J* = 10.7, 5.5 Hz, 4H), 2.48 – 2.34 (m, 5H), 2.31 (t, *J* = 5.1 Hz, 2H), 2.22 (t, *J* = 5.2 Hz, 2H).

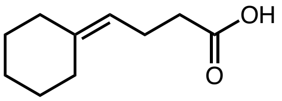

S13c: From cyclohexanone by procedure J and C. Yield 136 mg (68%) ^{1}H NMR (300 MHz, $CDCl_3$) δ 5.08 (t, *J* = 6.3 Hz, 1H), 2.53 – 2.27 (m, 4H), 2.25 – 2.12 (m, 1H), 2.12 – 2.00 (m, 2H), 1.54 (s, 4H).

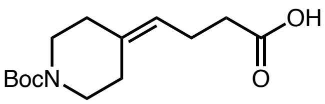

S16c: From 1-boc-4-piperidone by procedure J and C. Yield: 763 mg (43%). ^{1}H NMR (300 MHz, $CDCl_3$) δ 5.20 (t, *J* = 6.5 Hz, 1H), 3.79 – 3.58 (m, 4H), 2.44 – 2.18 (m, 8H), 1.28 (s, 9H).

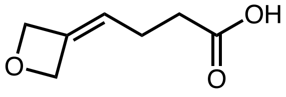

S17c: From 3-oxetanone by procedure J and C. Yield: 173 mg (32%). ^{1}H NMR (300 MHz, $CDCl_3$) δ 5.32 – 5.09 (m, 3H), 2.29 – 2.08 (m, 2H), 1.96 (dt, *J* = 13.6, 6.8 Hz, 3H).

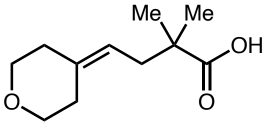

S18c: From methyl isobutyrate and 4-(2-bromethylidene)tetrahydro-2*H*-pyran by procedure L and C. Yield: 215 mg (72%). ^{1}H NMR (300 MHz, $CDCl_3$) δ 5.17 (t, *J* = 7.6 Hz, 1H), 3.67 (dt, *J* = 7.9, 5.5 Hz, 4H), 2.39 – 2.16 (m, 6H), 1.19 (s, 6H).

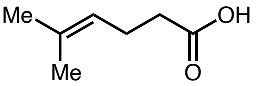

S19c: From tert-butylacetate and 1-bromo-3-methylbut-2-ene by procedure L and C. Yield: 183 mg (75%) ^{1}H NMR (500 MHz, $CDCl_3$) δ 5.17 (t, *J* = 6.7 Hz, 1H), 3.14 (t, *J* = 7.1 Hz, 2H), 2.12 (q, *J* = 7.3 Hz, 2H), 1.73 (s, 3H), 1.64 (s, 4H).

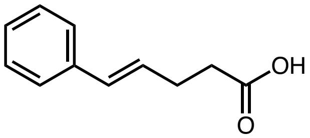

S21c: From benzaldehyde by procedure J and C. Yield: 110 mg (61%) H NMR (300 MHz, $CDCl_3$) δ 7.45 – 7.16 (m, 6H), 6.52 (d, *J* = 11.6 Hz, 1H), 5.66 (dt, *J* = 11.6, 7.1 Hz, 1H), 2.70 (q, *J* = 7.3 Hz, 2H), 2.61 – 2.45 (m, 2H).

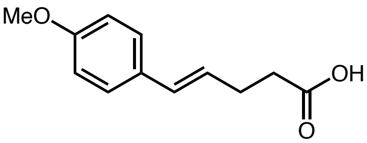

S23c: From 4-methoxybenzaldehyde by procedure J and C. Yield: 234 mg (57%) ^{1}H NMR (300 MHz, $CDCl_3$) δ 7.25 (d, *J* = 8.7 Hz, 2H), 6.90 (d, *J* = 8.7 Hz, 2H), 6.42 (d, *J* = 11.5 Hz, 1H), 5.55 (dt, *J* = 11.6, 7.1 Hz, 1H), 2.68 (q, *J* = 7.0 Hz, 2H), 2.45 (t, *J* = 7.6 Hz, 2H).

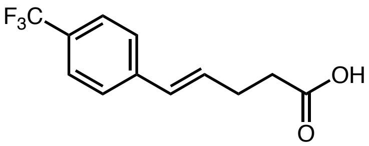

S24c: From 4-(trifluoromethyl)benzaldehyde by procedure J and C. Yield: 132 mg (21%). ^{1}H NMR (300 MHz, $CDCl_3$) δ 7.62 (d, *J* = 8.2 Hz, 2H), 7.40 (s, 2H), 6.54 (d, *J* = 11.7 Hz, 1H), 5.77 (dt, *J*= 11.7, 7.2 Hz, 1H), 2.68 (q, *J* = 7.1 Hz, 2H), 2.60 – 2.48 (m, 2H).

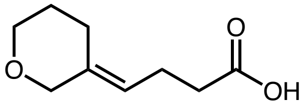

S25c: From **S25a** by procedure B and C. Mixture of E:Z isomers (1:1.7). Yield 143 mg (51%) ^{1}H NMR (300 MHz, $CDCl_3$) δ 5.21 (m, 1H, E:Z isomers), 4.17 (s, 2H, Z isomer), 3.99 (s, 2H, E isomer), 3.85 – 3.63 (m, 2H), 2.57 – 2.32 (m, 6H), 1.62 (m, 2H)

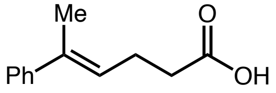

S25c From acetophenone by procedure J and C as a 90:10 mixture of E:Z isomers. Yield: 654 mg (82%). NMR peaks are for the major E isomer. ^{1}H NMR (500 MHz, $CDCl_3$) δ 7.36 (t, *J* = 7.5 Hz, 2H), 7.28 (t, *J* = 8.5 Hz, 1H), 7.18 (d, *J* = 7.4 Hz, 2H), 5.47 (t, *J* = 6.7 Hz, 1H), 2.51 – 2.25 (m, 4H), 2.05 (s, 3H).

Procedure D: Amide coupling (EDC)

Carboxylic acid (1 mmol, 1 equiv.) was dissolved in DCM (5 mL). To this solution was added EDC·HCl (1.5 equiv), DMAP (1.5 equiv), and aniline (2 equiv). The solution was stirred overnight, then diluted with 1 M HCl (100 mL). The aqueous layer was extracted with DCM (3 x 50 mL), the organic layer was dried over Na_2SO_4 and evaporated to dryness. The crude amide was purified by silica column chromatography.

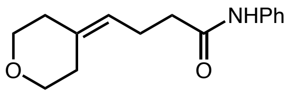

S1d: From **S1c** by procedure D. Purified by column chromatography (0–40% EtOAc/hexanes). Yield: 2.6 g (90%). ^{1}H NMR (500 MHz, $CDCl_3$) δ 7.52 (d, *J* = 7.7 Hz, 2H), 7.32 (d, *J* = 7.5 Hz, 2H), 7.11 (t, *J* = 7.4 Hz, 1H), 5.24 (t, *J* = 7.1 Hz, 1H), 3.71 – 3.59 (m, 4H), 2.44 – 2.38 (m, 4H), 2.30 (t, *J* = 5.5 Hz, 2H), 2.21 (t, *J* = 5.3 Hz, 2H).

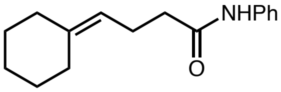

S13d: From **S13c** by procedure D. Purified by column chromatography (0–50% EtOAc/hexanes). Yield 129 mg (56%). ^{1}H NMR (300 MHz, $CDCl_3$) δ 7.52 (d, *J* = 7.9 Hz, 2H), 7.34 (t, *J* = 7.9 Hz, 2H), 7.21 (s, 1H), 7.12 (t, *J* = 7.4 Hz, 1H), 5.14 (d, *J* = 6.8 Hz, 1H), 2.42 (td, *J* = 11.0, 5.1 Hz, 5H), 2.19 (d, *J* = 6.3 Hz, 2H), 2.09 (d, *J* = 11.2 Hz, 3H), 1.54 (s, 7H).

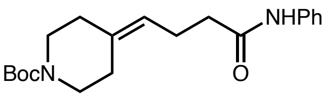

S16d: From **S16c** by procedure D. Purified by column chromatography (0–50% EtOAc/hexanes). Yield: 245 mg (86%) ^{1}H NMR (300 MHz, $CDCl_3$) δ 7.52 (d, *J* = 7.9 Hz, 2H), 7.34 (t, *J* = 7.9 Hz, 2H), 7.13 (s, 2H), 5.28 (s, 1H), 3.49 – 3.31 (m, 4H), 2.59 – 2.37 (m, 4H), 2.26 (s, 2H), 2.16 (s, 2H), 1.48 (s, 9H).

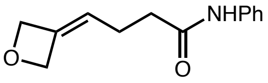

S17d: From **S17c** by procedure D. Purified by column chromatography (0–50% EtOAc/hexanes). Yield 136 mg (54%). ^{1}H NMR (300 MHz, $CDCl_3$) δ 7.53 (d, *J* = 8.0 Hz, 2H), 7.35 (t, *J* = 7.9 Hz, 2H), 7.14 (t, *J* = 7.2 Hz, 1H), 5.32 – 5.16 (m, 5H), 2.43 (t, *J* = 6.8 Hz, 2H), 2.31 (t, *J* = 6.8 Hz, 2H).

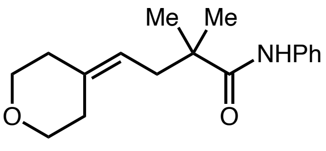

S18d: From **S18c** by procedure D. Purified by column chromatography (0–50% EtOAc/hexanes). Yield 96 mg (62%). ^{1}H NMR (500 MHz, $CDCl_3$) δ 7.53 (d, *J* = 7.6 Hz, 2H), 7.35 (t, *J* = 7.9 Hz, 2H), 7.13 (t, *J* = 7.4 Hz, 1H), 5.27 (t, *J* = 7.7 Hz, 1H), 3.65 (dt, *J* = 15.2, 5.5 Hz, 4H), 2.37 (d, *J* = 7.7 Hz, 2H), 2.31 (t, *J* = 5.3 Hz, 2H), 2.24 (t, *J* = 5.3 Hz, 2H), 1.32 (s, 6H).

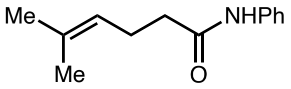

S19d: From S19c by procedure D. Purified by column chromatography (0–50% EtOAc/hexanes). Yield 290 mg (81%). ^{1}H NMR (300 MHz, $CDCl_3$) δ 7.56 (d, *J* = 7.6 Hz, 2H), 7.30 (t, *J* = 7.9 Hz, 2H), 7.07 (t, *J* = 7.4 Hz, 1H), 5.17 (t, *J* = 6.7 Hz, 1H), 2.43 (t, *J* = 6.8 Hz, 2H), 2.31 (t, *J* = 6.8 Hz, 2H), 1.72 (s, 3H), 1.64 (s, 3H).

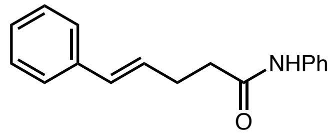

S21d: From S21c by procedure D. Purified by column chromatography (0–50% EtOAc/hexanes). Yield 432 mg (66%). ^{1}H NMR (300 MHz, $CDCl_3$) δ 7.51 (d, J = 7.9 Hz, 2H), 7.37 – 7.27 (m, 6H), 7.21 (t, J = 7.1 Hz, 1H), 7.10 (t, J = 7.4 Hz, 1H), 6.48 (d, J = 15.9 Hz, 1H), 6.26 (dt, J = 15.9, 6.9 Hz, 1H), 2.65 (m, 2H), 2.53 (t, J = 7.3 Hz, 2H).

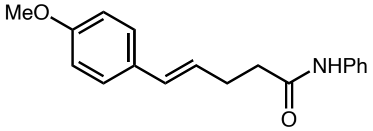

S23d: From S23c by procedure D. Purified by column chromatography (0–50% EtOAc/hexanes). Yield 211 mg (67%). ^{1}H NMR (300 MHz, $CDCl_3$) δ 7.50 (d, *J* = 7.9 Hz, 2H), 7.40 (t, *J* = 8.1 Hz, 2H), 7.13 (t, *J* = 7.2 Hz, 2H), 7.25 (d, *J* = 8.7 Hz, 2H), 6.90 (d, *J* = 8.7 Hz, 2H), 6.42 (d, *J* = 11.5 Hz, 1H), 5.55 (dt, *J* = 11.6, 7.1 Hz, 1H), 2.68 (q, *J* = 7.0 Hz, 2H), 2.45 (t, *J* = 7.6 Hz, 2H).

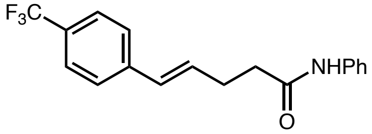

S24d: From S24c by procedure D. Purified by column chromatography (0–50% EtOAc/hexanes). Yield 145 mg (44%). ^{1}H NMR (300 MHz, $CDCl_3$) δ 7.61 (d, *J* = 8.1 Hz, 2H), 7.50 (d, *J* = 7.9 Hz, 2H), 7.42 (d, *J* = 8.4 Hz 1H), 7.40 (t, *J* = 8.1 Hz, 2H), 7.13 (t, *J* = 7.2 Hz, 2H), 6.55 (d, *J* = 11.7 Hz, 1H), 5.83 (dt, *J* = 11.6, 7.3 Hz, 1H), 2.78 (q, *J* = 7.1 Hz, 2H), 2.52 (t, *J* = 7.4 Hz, 2H).

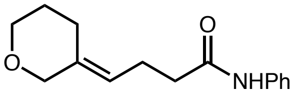

S25d: From **S25c** by procedure D. Purified by column chromatography (0–50% EtOAc/hexanes). Yield 112 mg (73%). Mixture of E:Z isomers (1:1.7). ^{1}H NMR (300 MHz, $CDCl_3$) δ 7.54 (d, *J* = 7.8 Hz, 3H), 7.34 (t, *J* = 7.9 Hz, 2H), 7.12 (t, *J* = 7.3 Hz, 2H), 5.28 (t, *J* = 7.0 Hz, 1H), 4.19 (s, 1H, E isomer), 4.01 (s, 1H, Z isomer), 3.86 – 3.63 (m, 4H), 2.57 – 2.15 (m, 4H), 1.88 – 1.44 (m, 4H).

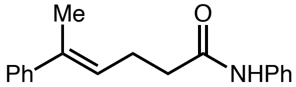

S26d: From **S26c** by procedure D. Purified by column chromatography (0–50% EtOAc/hexanes) as a 90:10 mixture of E:Z isomers. Yield: 438 mg (67%). NMR peaks are for the major E isomer. ^{1}H NMR (500 MHz, $CDCl_3$) δ 7.48 (d, *J* = 7.9 Hz, 2H), 7.33 (m, 3H), 7.26 (t, *J* = 7.4 Hz, 1H), 7.20 (d, *J* = 7.0 Hz, 3H), 7.11 (t, *J* = 7.4 Hz, 1H), 5.53 (t, *J* = 6.6 Hz, 1H), 2.52 – 2.28 (m, 4H), 2.06 (s, 3H)

Procedure E: Amide reduction to aniline

Amide (1 mmol, 1 equiv) was dissolved in dry THF (50 mL). The THF solution was cooled to 0 °C and $LiAlH_4$ (3 equiv.) was added as a solid. The solution was stirred overnight or until TLC analysis demonstrated complete consumption of the amide. The mixture was then diluted with Et_2O (100 mL) and slowly quenched with H_2O and 1 M NaOH at 0 °C. $MgSO_4$ was added to remove the water and the suspension was filtered through Celite. The organic solvent was evaporated to dryness and the crude aniline was purified by silica column chromatography.

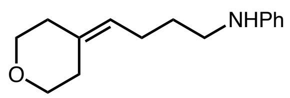

1: From **S1d** by procedure E. Product **1** was isolated by column chromatography (0-10 % EtOAc/hexanes). Yield: 512 mg (70%)

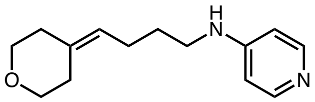

S11: From **S11I** by procedure E. Product **S11** was isolated by column chromatography (0-20 % DCM/Methanol). Yield: 136 mg (51%)

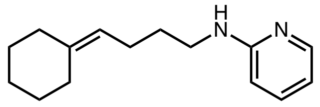

S12: From **S12I** by procedure E. Product **S12** was isolated by column chromatography (0-20 % DCM/Methanol). Yield: 97 mg (36%)

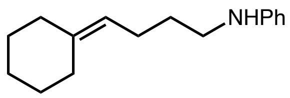

S13: From **S13d** by procedure E. Product **S13** was isolated by column chromatography (0-10% EtOAc/hexanes). Yield: 116 mg (72 %)

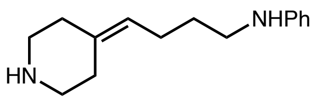

S14: From **S14k** by procedure E. Product **S14** was isolated by column chromatography (0-20% Methanol/DCM + 1% TEA). Yield: 214 mg (64%)

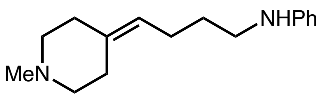

S15: From **S16d** by procedure E. Product **S15** was isolated by column chromatography (0-20% Methanol/DCM + 1% TEA). Yield: 145 mg (66%)

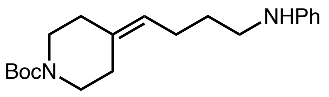

S16: From **S16d** by procedure E, monitored by TLC to limit reduction of the Boc protecting group. Product **S16** was isolated by column chromatography (0-20% EtOAc/Hexanes). Yield: 62 mg (52%)

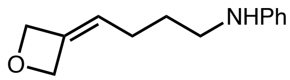

S17: From **S17d** by procedure E, monitored by TLC to limit reductive ring opening of the oxetane. Product **S17** was isolated by column chromatography (0-40% EtOAc/Hexanes). Yield: 57 mg (64%)

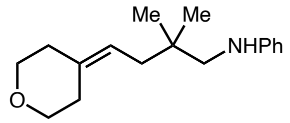

S18: From **S18d** by procedure E. Product **S18** was isolated by column chromatography (0-10% EtOAc/Hexanes). Yield: 125 mg (63%)

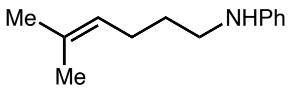

S19: From **S19d** by procedure E. Product **S19** was isolated by column chromatography (0-10% EtOAc/Hexanes). Yield: 348 mg (58%)

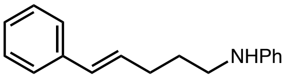

S21: From **S21d** by procedure E. Product **S21** was isolated by column chromatography (0-20% EtOAc/Hexanes). Yield: 367 mg (72%)

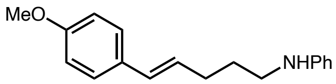

S23: From **S23d** by procedure E. Product **S23** was isolated by column chromatography (0-20% EtOAc/Hexanes). Yield: 147 mg (61%) as an 89:11 mixture of E:Z olefins. NMR peaks are reported for the major isomer.

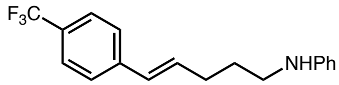

S24: From **S24d** by procedure E. Product **S24** was isolated by column chromatography (0-20% EtOAc/Hexanes). Yield: 241 mg (45%) as an 80:20 mixture of E:Z olefins. NMR peaks are reported for the major isomer.

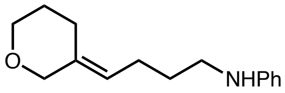

S25: From **S25d** by procedure E. Product **S25** was isolated by column chromatography (0-10% EtOAc/Hexanes). Yield: 68 mg (32%) as a 1:1.7 mixture of E:Z olefins. NMR peaks are reported for the major isomer.

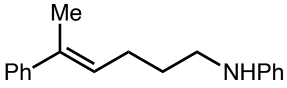

S24: From **S24d** by procedure E. Product **S24** was isolated by column chromatography (0-20% EtOAc/Hexanes). Yield: 256 mg (74%) as a 98:2 mixture of E:Z olefins. NMR peaks are reported for the major isomer.

Procedure F: Carboxylic acid to alcohol
The same protocol as **E** was followed except carboxylic acid was used as the starting material and 5 equiv. $LiAlH_4$ were added.

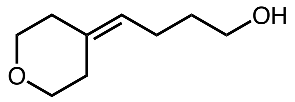

S1F: From **S1C** by procedure F. Purified by silica gel column chromatography (0–50% EtOAc/hexanes) Yield: 1.6 g (76%). ^{1}H NMR (500 MHz, $CDCl_3$) δ 5.23 (t, J = 7.4 Hz, 1H), 3.75 – 3.63 (m, 8H), 2.30 (t, J = 5.2 Hz, 2H), 2.23 (d, J = 5.2 Hz, 2H), 2.12 (q, J = 7.4 Hz, 3H), 1.76 – 1.59 (m, 4H).

Procedure G: Alcohol to aldehyde
Alcohol (1 equiv, 1 mmol) was dissolved in MeCN (10 mL). Solid IBX (1.5 equiv) was added and the suspension was heated to 60 °C for 3 h. The solution was cooled to room temperature, filtered, and subjected to silica column chromatography to obtain the aldehyde.

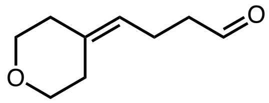

S1G: From **S1F** by procedure G. Purified by silica gel column chromatography (0–20% EtOAc/hexanes) Yield: 1.2 g (83%). ^{1}H NMR (300MHz, $CDCl_3$) δ 9.79 (s, 1H), 5.18 (t, J = 7.2 Hz, 1H), 3.67 (td, J = 5.5, 2.6 Hz, 4H), 2.58 – 2.46 (m, 2H), 2.38 (t, J = 7.1 Hz, 2H), 2.30 (t, J = 5.4 Hz, 2H), 2.21 (t, J = 5.2 Hz, 2H).

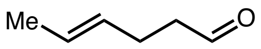

S20G: From (*E*)-hex-4-en-1-ol by procedure G. Yield: 950 mg (79%). ^{1}H NMR (300MHz, $CDCl_3$) δ 9.72 (s, 1H), 5.42 (m, 2H), 3.14 (m, 2H), 2.10 (m, 2H), 1.68 (d, J = 6.4 Hz, 3H)

Procedure H: Reductive amination
Aldehyde (1 equiv, 0.5 mmol) was dissolved in DCM (2 mL). $NaHB(OAc)_3$ (1.5 equiv.), aniline (1.2 equiv.), and HOAc (50 μL) were added. The solution was stirred for 3 h then diluted with water (20 mL) and DCM (20 mL). The aqueous layer was extracted with DCM (3 x 20 mL) and the combined organic layers were dried over Na_2SO_4 and evaporated to dryness. The crude aniline was purified by silica column chromatography.

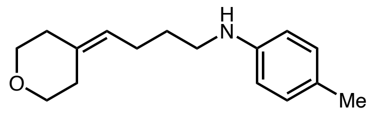

S3: From **S1G** and 4-methylaniline by procedure H. Product **S3** was isolated by column chromatography (0-10% EtOAc/hexanes). Yield: 50. mg (41%)

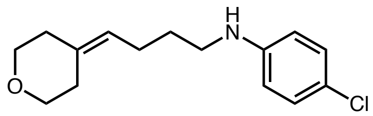

S4: From **S1G** and 4-chloroaniline by procedure H. Product **S4** was isolated by column chromatography (0-10% EtOAc/hexanes). Yield: 81 mg (60%)

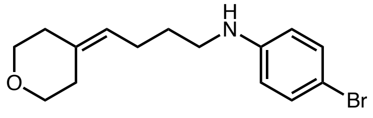

S5: From **S1G** and 4-bromoaniline by procedure H. Product **S5** was isolated by column chromatography (0-10% EtOAc/hexanes). Yield: 97 mg (62%)

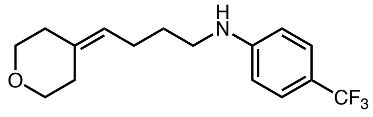

S6: From **S1G** and 4-(trifluoromethyl)aniline by procedure H. Product **S6** was isolated by column chromatography (0-100% hexanes/DCM). Yield: 102 mg (68%)

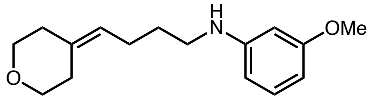

S7: From **S1G** and 3-methoxyaniline by procedure H. Product **S7** was isolated by column chromatography (0-10% EtOAc/hexanes). Yield: 72 mg (55%)

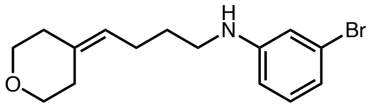

S8: From **S1G** and 3-bromoaniline by procedure H. Product **S8** was isolated by column chromatography (0-10% EtOAc/hexanes). Yield: 122 mg (94%)

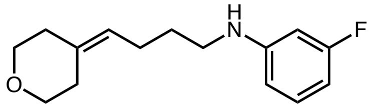

S9: From **S1G** and 3-fluoroaniline by procedure H. Product **S9** was isolated by column chromatography (0-10 % EtOAc/hexanes). Yield: 38 mg (36%)

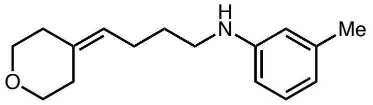

S10: From **S1G** and 3-methylaniline by procedure H. Product **S10** was isolated by column chromatography (0-10% EtOAc/hexanes). Yield: 51 mg (41%)

Procedure I: Amide coupling (HATU)

Carboxylic acid (1 mmol, 1 equiv.) was dissolved in DMF (5 mL). To this solution was added HATU (1.5 equiv), DIPEA (1.5 equiv), and aniline (2 equiv). The solution was stirred overnight, then diluted with water (100 mL). The aqueous layer was extracted with DCM (3 x 50 mL), the organic layer was dried over Na_2SO_4 and evaporated to dryness. The crude amide was purified by silica column chromatography.

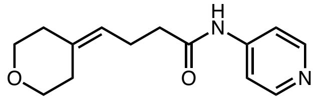

S11I: From **S1C** and 4-aminopyridine by procedure I. Purified by column chromatography (0–20% MeOH/DCM). Yield: 136 mg (68%). ^{1}H NMR (300 MHz, $CDCl_3$) δ 8.52 (d, *J* = 6.1 Hz, 2H), 7.51 (d, *J* = 6.1 Hz, 2H), 5.24 (t, 1H), 3.78 – 3.61 (m, 4H), 2.47 (m, 4H), 2.33 (s, 2H), 2.24 (d, *J* = 5.3 Hz, 2H).

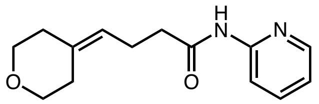

S12I: From **S1C** and 2-aminopyridine by procedure I. Purified by column chromatography (0–20% MeOH/DCM). Yield: 97 mg (45%). ^{1}H NMR (300 MHz, $CDCl_3$) δ 8.28 (d, *J* = 4.0 Hz, 1H), 8.22 (d, *J* = 8.3 Hz, 1H), 7.78 – 7.66 (m, 1H), 7.05 (dd, *J* = 6.8, 5.5 Hz, 1H), 5.25 (s, 1H), 3.74 – 3.58 (m, 4H), 2.54 – 2.42 (m, 4H), 2.35 – 2.29 (t, *J* = 5.1 Hz, 2H), 2.22 (t, *J* = 5.1 Hz, 2H).

Procedure J: Wittig Olefination

[3-(Ethoxycarbonyl)propyl]triphenylphosphonium bromide (1.5 equiv) and KO^tBu (1.5 equiv.) were dissolved in THF (40 mL) and stirred at room temperature. After 1 h, ketone (1 equiv, 1 mmol) was added and the solution was stirred overnight. After 16 h, 1 M HCl (20 mL) was added to quench the reaction and the mixture was diluted with water (20 mL) and DCM (20 mL). The aqueous layer was extracted with DCM (3 x 20 mL) and the combined organic extracts were dried over Na_2SO_4 and evaporated to dryness. The crude esters were subjected to column chromatography to remove $OPPh_3$ and directly hydrolyzed (procedure C, above).

Procedure K: Boc Deprotection

Boc-protected amine (1 mmol, 1 equiv) was dissolved in DCM (8 mL). Trifluoroacetic acid (2 mL) was added dropwise and the mixture was stirred for 1 h. After 1 h, 2M NaOH (100 mL) was added and the solution was extracted with EtOAc (3 x 100 mL). The organic extracts were dried with Na_2SO_4 and evaporated to obtain the deprotected amine which was directly subjected to further reactions (procedure E, above).

Procedure L: Addition of alkyl halides α to esters

A solution of ester (1 equiv, 2 mmol) in THF (20 mL) was cooled to – 78 °C. Freshly generated LDA (1.2 equiv.) was added and the solution was stirred for 1 h. After 1 h, allyl bromide (1.4 equiv) was added, the solution was warmed to room temperature, and stirred for an additional 2 h. After 2 h, water (10 mL) was added to quench the reaction. The aqueous layer was extracted with DCM (3 x 20 mL) and the combined organic extracts were dried over Na_2SO_4 and evaporated to dryness. The crude ester was subjected directly to hydrolysis (procedure C, above).

N-(4-(tetrahydro-4*H*-pyran-4-ylidene)butyl)aniline (**1**)

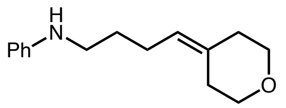

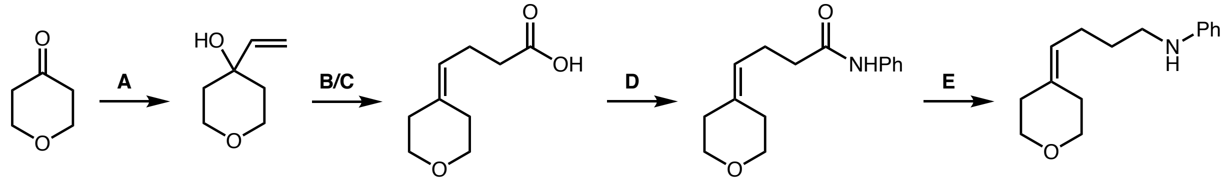

Synthesized according to Procedures A, B, C, D, and E.

^{1}H NMR (500 MHz, $CDCl_3$) δ 7.17 (t, *J* = 7.6 Hz, 2H), 6.69 (t, *J* = 7.6 Hz, 1H), 6.60 (d, *J* = 7.8 Hz, 2H), 5.22 (t, *J* = 7.0 Hz, 1H), 3.67 (t, *J* = 5.3 Hz, 2H), 3.64 (t, *J* = 5.3 Hz, 2H), 3.59 (s, 1H), 3.12 (t, *J* = 6.9 Hz, 2H), 2.27 (t, *J* = 5.0 Hz, 2H), 2.21 (t, *J* = 4.8 Hz, 2H), 2.13 (q, *J* = 7.1 Hz, 2H), 1.68 (p, *J* = 7.1 Hz, 2H).

^{13}C NMR (126 MHz, $CDCl_3$) δ 148.55, 135.26, 129.39, 122.53, 117.36, 112.85, 77.16, 69.82, 68.90, 43.60, 37.09, 29.89, 24.60.

IR (neat, cm^{-1}): 3371 (m), 2954 (m), 2844 (m), 1608 (s), 1506 (m), 1097 (m), 747 (m), 691 (w)

HRMS (ESI-MS): m/z calcd. for $C_{15}H_{21}NO$ [M+H$^+$]: 232.1701; found 232.1698

4-methyl-*N*-(4-(tetrahydro-4*H*-pyran-4-ylidene)butyl)aniline (**S3**)

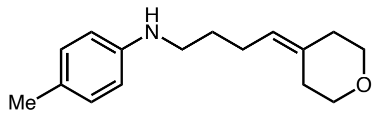

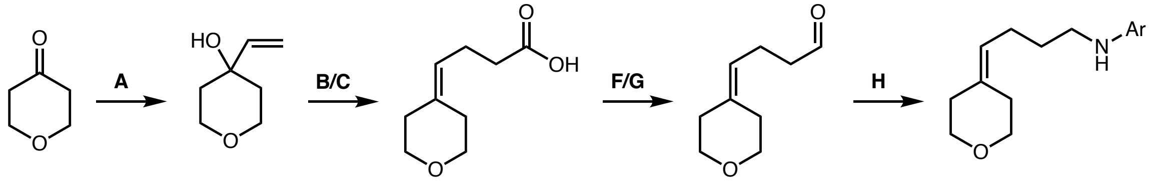

Synthesized according to Procedures A, B, C, F, G, and H.

^{1}H NMR (500 MHz, $CDCl_3$) δ 6.98 (d, *J* = 7.9 Hz, 2H), 6.53 (d, *J* = 8.1 Hz, 2H), 5.22 (t, *J* = 7.0 Hz, 1H), 3.67 (t, *J* = 5.3 Hz, 2H), 3.64 (t, *J* = 5.3 Hz, 2H), 3.50 (m, 1H), 3.10 (t, *J* = 7.0 Hz, 2H), 2.30 – 2.25 (m, 2H), 2.24 (s, 3H), 2.23 – 2.19 (m, 2H), 2.12 (q, *J* = 7.1 Hz, 2H), 1.66 (p, *J* = 7.1 Hz, 2H).

^{13}C NMR (126 MHz, $CDCl_3$) δ 146.31, 135.17, 129.86, 126.54, 122.60, 113.06, 69.82, 68.90, 43.98, 37.09, 29.88, 24.60, 20.51.

IR (neat, cm^{-1}): 3382 (m), 2956 (m), 2849 (m), 1619 (s), 1553 (s), 1256, 1099 (m), 807 (w)

HRMS (ESI-MS): m/z calcd. for $C_{16}H_{23}NO$ [M+H$^+$]: 246.1858; found 246.1860

4-chloro-*N*-(4-(tetrahydro-4*H*-pyran-4-ylidene)butyl)aniline (**S4**)

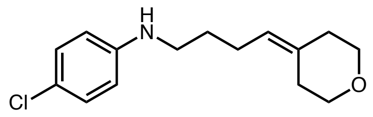

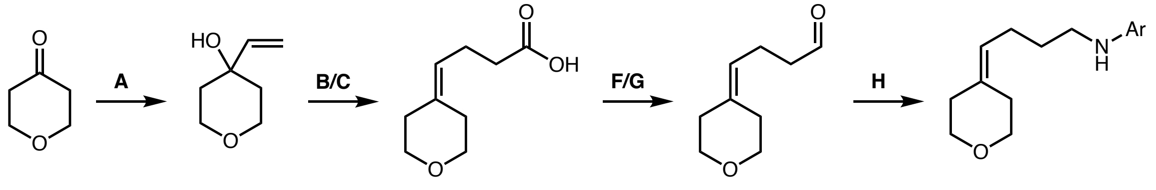

Synthesized according to Procedures A, B, C, F, G, and H.

^{1}H NMR (500 MHz, $CDCl_3$) δ 7.11 (d, *J* = 8.5 Hz, 2H), 6.50 (d, *J* = 8.5 Hz, 2H), 5.21 (t, *J* = 7.0 Hz, 1H), 3.67 (t, *J* = 5.3 Hz, 2H), 3.65 – 3.57 (m, 4H), 3.08 (t, *J* = 6.9 Hz, 2H), 2.26 (t, *J* = 5.0 Hz, 2H), 2.21 (t, *J* = 4.8 Hz, 2H), 2.12 (q, *J* = 7.1 Hz, 2H), 1.66 (p, *J* = 7.0 Hz, 2H).

^{13}C NMR (126 MHz, $CDCl_3$) δ 147.33, 135.65, 129.43, 122.60, 114.10, 70.05, 69.12, 43.92, 37.33, 30.13, 29.96, 24.78.

IR (neat, cm^{-1}): 3362 (m), 2955 (m), 2847 (m), 1600 (s), 1500 (m), 1319 (m), 1095 (w), 814 (w)

HRMS (ESI-MS): m/z calcd. for $C_{15}H_{20}NOCl$ [$M+H^+$]: 266.1312; found 266.1321

4-bromo-*N*-(4-(tetrahydro-4*H*-pyran-4-ylidene)butyl)aniline (**S5**)

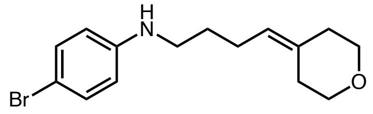

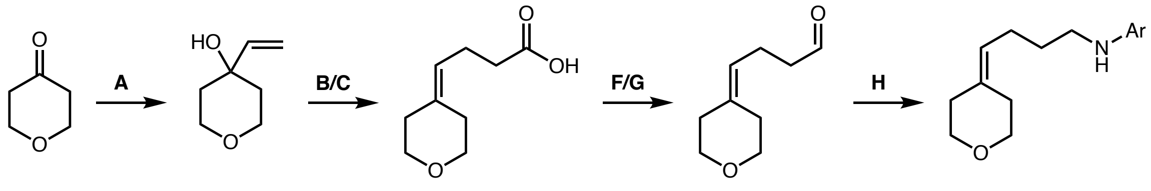

Synthesized according to Procedures A, B, C, F, G, and E.

^{1}H NMR (500 MHz, $CDCl_3$) δ 7.24 (d, *J* = 8.5 Hz, 2H), 6.46 (d, *J* = 8.5 Hz, 2H), 5.20 (t, *J* = 7.0 Hz, 1H), 3.67 (t, *J* = 5.3 Hz, 2H), 3.65 – 3.60 (m, 4H), 3.08 (s, 2H), 2.26 (t, *J* = 5.1 Hz, 2H), 2.21 (t, *J* = 4.9 Hz, 2H), 2.12 (q, *J* = 7.1 Hz, 2H), 1.66 (p, *J* = 7.1 Hz, 2H).

^{13}C NMR (126 MHz, $CDCl_3$) δ 147.35, 135.29, 131.92, 122.20, 114.22, 108.70, 69.67, 68.74, 43.44, 36.95, 29.76, 29.55, 24.39.

IR (neat, cm^{-1}): 3367 (m), 2955 (m), 2851 (m), 1597 (s), 1500 (m), 1321 (m), 1097 (w), 812 (w)

HRMS (ESI-MS): m/z calcd. for $C_{15}H_{20}NOBr$ [$M+H^{+}$]: 310.0807, 312.0786; found 310.0813, 312.0784

4-trifluoromethyl-*N*-(4-(tetrahydro-4*H*-pyran-4-ylidene)butyl)aniline (**S6**)

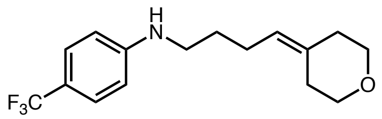

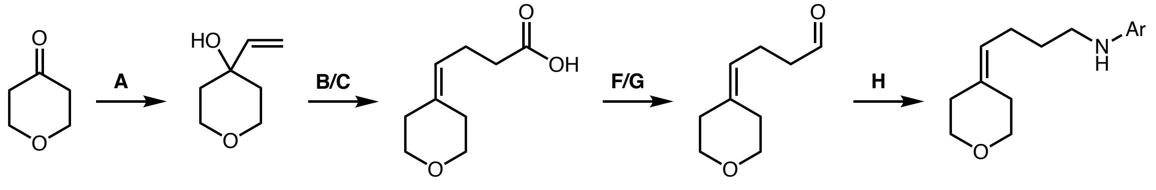

Synthesized according to Procedures A, B, C, F, G, and H.

^{1}H NMR (500 MHz, $CDCl_3$) δ 7.39 (d, *J* = 8.4 Hz, 2H), 6.58 (d, *J* = 8.5 Hz, 2H), 5.21 (t, *J* = 7.3 Hz, 1H), 3.95 (s, 1H), 3.72 – 3.65 (m, 2H), 3.65 – 3.61 (m, 2H), 3.15 (q, *J* = 6.9 Hz, 2H), 2.26 (t, *J* = 5.1 Hz, 2H), 2.24 – 2.19 (m, 2H), 2.13 (q, *J* = 7.3 Hz, 2H), 1.69 (p, *J* = 7.2 Hz, 2H).

^{13}C NMR (126 MHz, $CDCl_3$) δ 150.87, 135.57, 126.77, 126.74, 122.19, 118.86, 118.61, 111.82, 69.80, 68.86, 43.11, 37.08, 29.90, 29.59, 24.48, 14.91.

IR (neat, cm^{-1}): 3356 (m), 2957 (m), 2853 (m), 1619 (s), 1537 (m) 1326 (m), 1101 (m), 825 (w)

HRMS (ESI-MS): m/z calcd. for $C_{16}H_{21}NOF_3$ [M+H$^+$]: 300.1575; found 300.1598

3-methoxy-*N*-(4-(tetrahydro-4*H*-pyran-4-ylidene)butyl)aniline (**S7**)

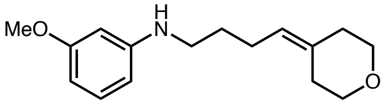

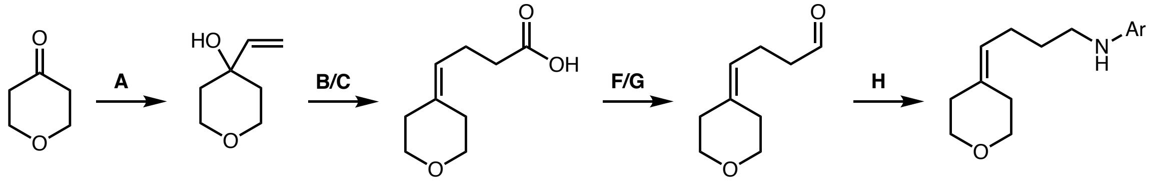

Synthesized according to Procedures A, B, C, F, G, and H.

^{1}H NMR (500 MHz, $CDCl_3$) δ 7.07 (t, *J* = 8.0 Hz, 1H), 6.26 (d, *J* = 7.8 Hz, 1H), 6.21 (d, *J* = 7.8 Hz, 1H), 6.15 (s, 1H), 5.21 (t, *J* = 7.0 Hz, 1H), 3.77 (s, 3H), 3.67 (t, *J* = 5.2 Hz, 2H), 3.65 – 3.59 (m, 3H), 3.11 (t, *J* = 6.8 Hz, 2H), 2.27 (t, *J* = 4.9 Hz, 2H), 2.21 (t, *J* = 4.6 Hz, 2H), 2.12 (q, *J* = 7.1 Hz, 2H), 1.67 (p, *J* = 7.0 Hz, 2H).

^{13}C NMR (126 MHz, $CDCl_3$) δ 161.01, 149.95, 135.27, 130.10, 122.50, 106.11, 102.34, 98.84, 69.81, 68.89, 55.22, 43.58, 37.09, 29.89, 29.83, 24.58.

IR (neat, cm^{-1}): 3390 (m), 2955 (m), 2845 (m), 1615 (s), 1518 (m), 1213 (m), 1162 (m), 833 (w)

HRMS (ESI-MS): m/z calcd. for $C_{16}H_{23}NO_2$ [M+H$^+$]: 262.1807; found 262.1791

3-bromo-*N*-(4-(tetrahydro-4*H*-pyran-4-ylidene)butyl)aniline (**S8**)

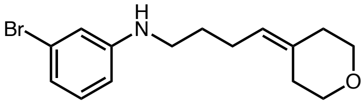

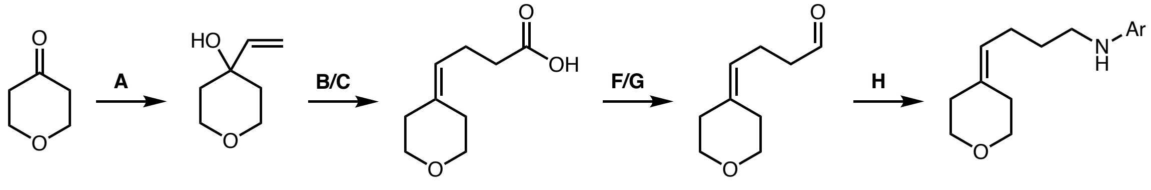

Synthesized according to Procedures A, B, C, F, G, and H.

^{1}H NMR (500 MHz, $CDCl_3$) δ 7.00 (t, *J* = 8.0 Hz, 1H), 6.79 (m, 1H), 6.72 (t, *J* = 2.1 Hz, 1H), 6.49 (m, 1H), 5.21 (t, *J* = 7.3 Hz, 1H), 3.71 – 3.65 (m, 3H), 3.66 – 3.60 (m, 2H), 3.09 (q, *J* = 7.0 Hz, 2H), 2.30 – 2.25 (m, 2H), 2.22 (t, *J* = 5.1 Hz, 2H), 2.12 (q, *J* = 7.3 Hz, 2H), 1.66 (p, *J* = 7.2 Hz, 2H).

^{13}C NMR (126 MHz, $CDCl_3$) δ 150.02, 135.73, 130.85, 122.55, 120.28, 115.48, 111.86, 70.05, 69.12, 43.60, 37.33, 30.14, 29.92, 24.55.

IR (neat, cm^{-1}): 3414 (m), 2957 (m), 2849 (m), 1597 (s), 1502 (m), 1326 (m), 1097 (m), 985 (w)

HRMS (ESI-MS): m/z calcd. for $C_{15}H_{20}NOBr$ [M+H$^+$]: 310.0807, 312.0786; found 310.0810, 312.0787

3-fluoro-*N*-(4-(tetrahydro-4*H*-pyran-4-ylidene)butyl)aniline (**S9**)

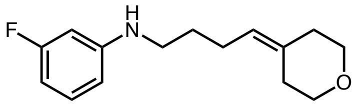

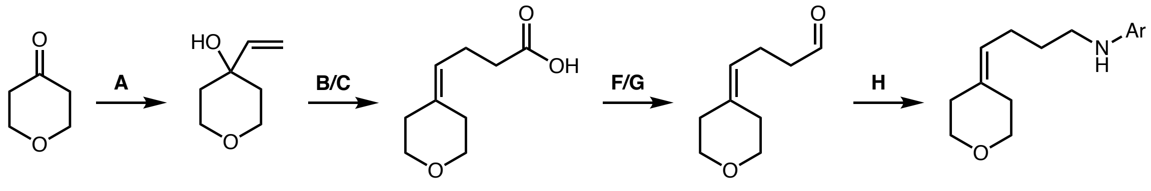

Synthesized according to Procedures A, B, C, F, G, and H.

^{1}H NMR (500 MHz, $CDCl_3$) δ 7.10 (q, *J* = 7.8 Hz, 1H), 6.43 – 6.34 (m, 2H), 6.30 (d, *J* = 11.7 Hz, 1H), 5.23 (t, *J* = 7.0 Hz, 1H), 3.77 (s, 1H), 3.70 (t, *J* = 5.3 Hz, 2H), 3.66 (t, *J* = 5.3 Hz, 2H), 3.12 (t, *J* = 7.0 Hz, 2H), 2.29 (t, *J* = 5.0 Hz, 2H), 2.24 (t, *J* = 4.8 Hz, 2H), 2.15 (q, *J* = 7.1 Hz, 2H), 1.70 (p, *J* = 7.0 Hz, 2H).

^{13}C NMR (126 MHz, $CDCl_3$) δ 165.16, 163.23, 150.20, 150.11, 135.32, 130.29, 130.20, 122.19, 108.61, 103.63, 103.46, 99.29, 99.09, 69.67, 68.73, 43.33, 36.95, 29.76, 29.54, 24.38.

IR (neat, cm^{-1}): 3365 (m), 2957 (m), 2851 (m), 1623 (s), 1513 (m), 1340 (m), 1149 (m), 1099(m)

HRMS (ESI-MS): m/z calcd. for $C_{15}H_{20}NOF$ [M+H$^+$]: 250.1607; found 250.1604

3-methyl-*N*-(4-(tetrahydro-4*H*-pyran-4-ylidene)butyl)aniline (**S10**)

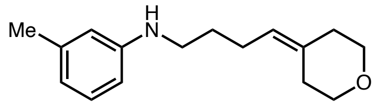

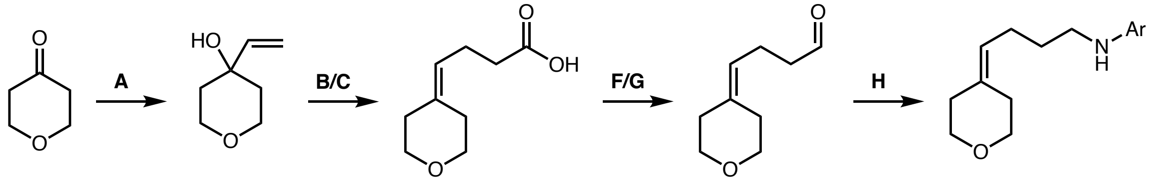

Synthesized according to Procedures A, B, C, F, G, and H.

^{1}H NMR (500 MHz, $CDCl_3$) δ 7.09 (t, *J* = 7.5 Hz, 1H), 6.55 (d, *J* = 7.1 Hz, 1H), 6.44 (m, 2H), 5.25 (t, *J* = 7.0 Hz, 1H), 3.70 (t, *J* = 5.3 Hz, 2H), 3.67 (t, *J* = 5.3 Hz, 2H), 3.57 (s, 1H), 3.14 (t, *J* = 7.0 Hz, 2H), 2.30 (m, 5H), 2.24 (t, *J* = 4.8 Hz, 2H), 2.15 (q, *J* = 7.1 Hz, 2H), 1.69 (p, *J* = 7.0 Hz, 2H).

^{13}C NMR (126 MHz, $CDCl_3$) δ 148.48, 139.01, 135.08, 129.05, 122.45, 118.18, 113.52, 109.90, 69.69, 68.77, 43.50, 36.96, 29.80, 29.30, 24.47, 21.66.

IR (neat, cm^{-1}): 3382 (m), 2957 (m), 2847 (m), 1606 (s), 1492(m), 1328 (m), 1099 (m), 768 (w)

HRMS (ESI-MS): m/z calcd. for $C_{15}H_{21}NO$ [M+H$^+$]: 246.1858; found 246.1856

N-(4-(tetrahydro-4*H*-pyran-4-ylidene)butyl)pyridin-4-amine (**S11**)

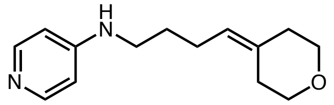

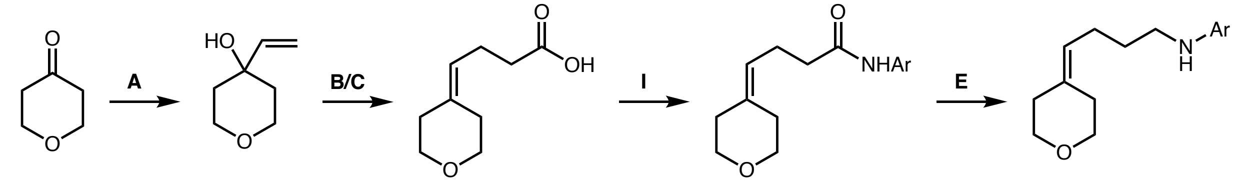

Synthesized according to Procedures A, B, C, I, and E.

^{1}H NMR (500 MHz, $CDCl_3$) δ 8.17 (s, 2H), 6.42 (d, *J* = 5.8 Hz, 2H), 5.20 (t, *J* = 7.0 Hz, 1H), 4.25 (s, 1H), 3.72 – 3.65 (m, 2H), 3.63 (t, *J* = 5.2 Hz, 2H), 3.15 (q, *J* = 6.4 Hz, 2H), 2.25 (t, *J* = 5.0 Hz, 2H), 2.23 – 2.17 (m, 2H), 2.12 (q, *J* = 7.1 Hz, 2H), 1.69 (q, *J* = 7.1 Hz, 2H).

^{13}C NMR (126 MHz, $CDCl_3$) δ 149.75, 135.59, 121.87, 107.46, 69.64, 68.70, 42.15, 36.93, 29.76, 29.28, 24.27.

IR (neat, cm^{-1}): 3240 (m), 2953 (m), 2845 (m), 1604 (s), 1526 (m), 1217 (m), 1099 (m), 987 (w)

HRMS (ESI-MS): m/z calcd. for $C_{14}H_{20}N_2O$ [$M+H^+$]: 233.1654; found 233.1668

N-(4-(tetrahydro-4*H*-pyran-4-ylidene)butyl)pyridin-2-amine (**S12**)

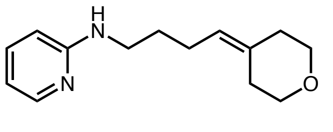

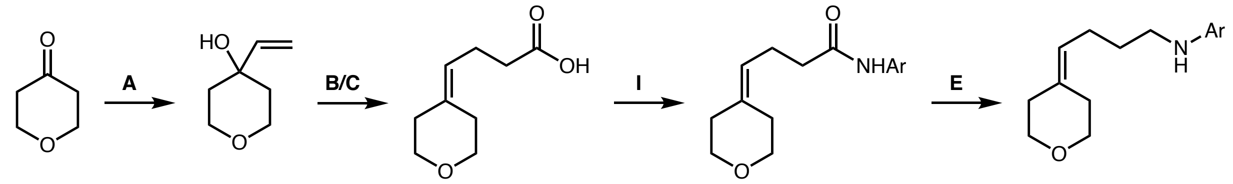

Synthesized according to Procedures A, B, C, I, and E.

^{1}H NMR (500 MHz, $CDCl_3$) δ 8.09 (d, *J* = 4.0 Hz, 1H), 7.43 (t, *J* = 7.4 Hz, 1H), 6.61 – 6.53 (m, 1H), 6.38 (d, *J* = 8.3 Hz, 1H), 5.23 (t, *J* = 6.9 Hz, 1H), 4.49 (s, 1H), 3.73 – 3.66 (m, 2H), 3.64 (t, *J* = 5.3 Hz, 2H), 3.26 (m, 2H), 2.28 (t, *J* = 5.0 Hz, 2H), 2.22 (t, *J* = 4.8 Hz, 2H), 2.15 (q, *J* = 7.1 Hz, 2H), 1.71 (p, *J* = 7.1 Hz, 2H).

^{13}C NMR (126 MHz, $CDCl_3$) δ 158.88, 148.25, 137.40, 135.16, 122.29, 112.62, 106.32, 69.66, 68.63, 41.72, 36.78, 29.70, 24.34.

IR (neat, cm^{-1}): 3367 (m), 2957 (m), 2849 (m), 1603 (s), 1515 (m), 1287 (m), 1099 (m), 771 (w)

HRMS (ESI-MS): m/z calcd. for $C_{14}H_{20}N_2O$ [$M+H^+$]: 233.1654; found 233.1646

N-(4-cyclohexylidenebutyl)aniline (**S13**)

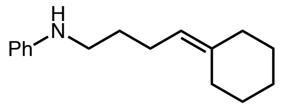

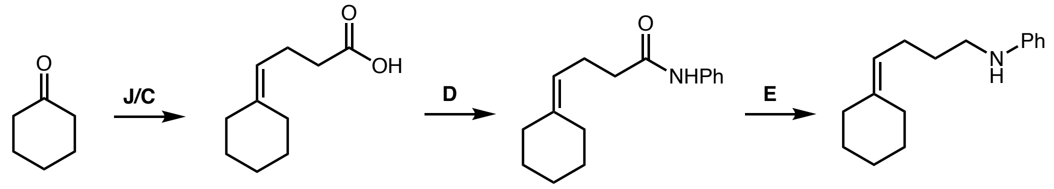

Synthesized according to Procedures J, C, D, and E.

^{1}H NMR (500 MHz, $CDCl_3$) δ 7.19 (t, *J* = 7.6 Hz, 2H), 6.71 (t, *J* = 7.2 Hz, 1H), 6.62 (d, *J* = 7.8 Hz, 2H), 5.12 (t, *J* = 6.9 Hz, 1H), 3.64 (s, 1H), 3.14 (t, *J* = 6.7 Hz, 2H), 2.14 (m, 6H), 1.68 (p, *J* = 7.0 Hz, 2H), 1.61 – 1.46 (m, 7H).

^{13}C NMR (126 MHz, $CDCl_3$) δ 148.52, 140.55, 129.22, 120.36, 117.08, 112.72, 43.56, 37.19, 29.86, 28.69, 27.84, 26.92, 24.65.

IR (neat, cm^{-1}): 3410 (m), 2926 (m), 2853 (m), 1604 (s), 1507 (m), 1321 (m), 747 (w), 691 (w)

HRMS (ESI-MS): m/z calcd. for $C_{16}H_{23}N$ [M+H$^+$]: 230.1909; found 230.1900

N-(4-(piperidin-4-ylidene)butyl)aniline (**S14**)

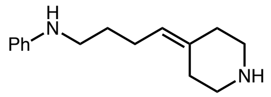

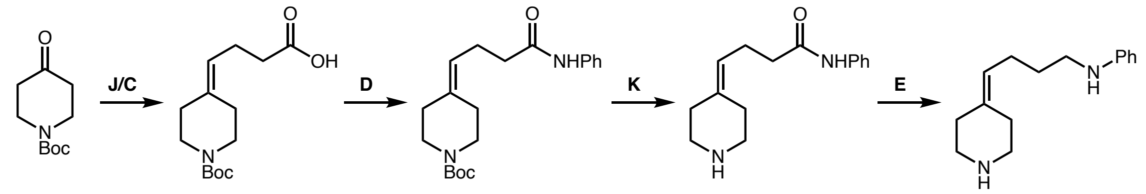

Synthesized according to Procedures J, C, D, K, and E.

^{1}H NMR (500 MHz, $CDCl_3$) δ 7.17 (t, *J* = 7.5 Hz, 2H), 6.69 (t, *J* = 7.1 Hz, 1H), 6.60 (d, *J* = 7.8 Hz, 2H), 5.17 (t, *J* = 6.9 Hz, 1H), 3.60 (s, 1H), 3.12 (t, *J* = 6.8 Hz, 2H), 2.84 (m, 4H), 2.28 – 2.05 (m, 6H), 1.67 (p, *J* = 6.9 Hz, 2H).

^{13}C NMR (126 MHz, $CDCl_3$) δ 148.44, 136.49, 129.25, 122.28, 117.21, 112.73, 62.77, 48.29, 47.46, 46.15, 43.49, 37.13, 29.94, 29.76, 29.49, 24.49.

IR (neat, cm^{-1}): 3311 (m), 2935 (m), 2860 (m), 1604 (s), 1507 (m), 1323 (m), 1261 (m), 749 (w)

HRMS (ESI-MS): m/z calcd. for $C_{16}H_{22}N_2$ [M+H$^+$]: 231.1861; found 231.1843

N-(4-(1-methylpiperidin-4-ylidene)butyl)aniline (**S15**)

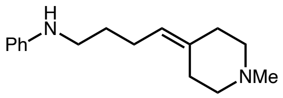

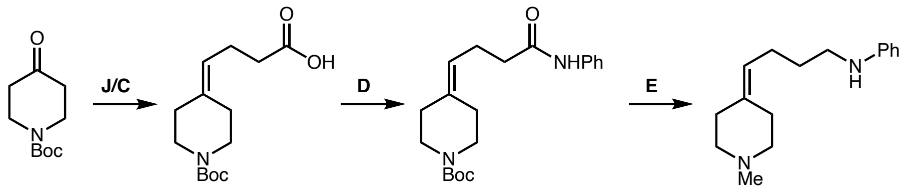

Synthesized according to Procedures J, C, D, and E.

^{1}H NMR (500 MHz, $CDCl_3$) δ 7.21 – 7.12 (m, 2H), 6.68 (t, *J* = 7.3 Hz, 1H), 6.60 (d, *J* = 7.9 Hz, 2H), 5.18 (t, *J* = 7.3 Hz, 1H), 3.60 (s, 1H), 3.12 (q, *J* = 6.7 Hz, 2H), 2.41 – 2.37 (m, 2H), 2.37 – 2.33 (m, 2H), 2.29 (m, 5H), 2.23 (q, *J* = 5.6 Hz, 2H), 2.16 – 2.10 (m, 2H), 1.67 (p, *J* = 7.2 Hz, 2H).

^{13}C NMR (126 MHz, $CDCl_3$) δ 148.46, 136.27, 129.24, 121.95, 117.17, 112.74, 57.54, 56.69, 46.25, 43.51, 36.08, 29.78, 28.31, 24.70.

IR (neat, cm^{-1}): 3410 (m), 2935 (m), 2782 (m), 1603 (s), 1506 (s), 1278 (m), 1131 (m), 745 (w)

HRMS (ESI-MS): m/z calcd. for $C_{16}H_{24}N_2$ [M+H$^+$]: 245.2018; found 245.2002

tert-butyl 4-(4-(phenylamino)butylidene)piperidine-1-carboxylate (**S16**)

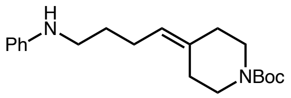

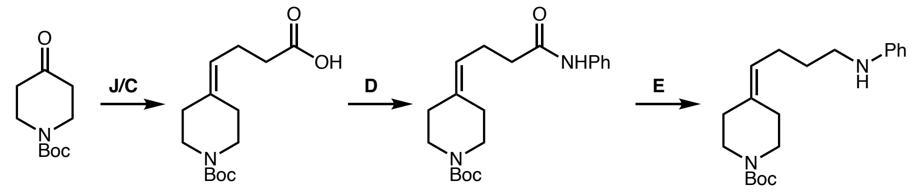

Synthesized according to Procedures J, C, D, and E.

^{1}H NMR (500 MHz, $CDCl_3$) δ 7.17 (t, *J* = 7.6 Hz, 2H), 6.69 (t, *J* = 7.2 Hz, 1H), 6.59 (d, *J* = 8.1 Hz, 2H), 5.24 (t, *J* = 7.1 Hz, 1H), 3.59 (s, 1H), 3.39 (dd, *J* = 11.9, 5.7 Hz, 4H), 3.12 (t, *J* = 7.0 Hz, 2H), 2.20 (s, 2H), 2.13 (q, *J* = 7.0 Hz, 4H), 1.67 (p, *J* = 7.1 Hz, 2H), 1.47 (s, 9H).

^{13}C NMR (126 MHz, $CDCl_3$) δ 154.82, 148.41, 135.91, 129.26, 123.11, 117.25, 112.73, 79.47, 43.48, 35.90, 29.75, 28.48, 24.66.

IR (neat, cm^{-1}): 3370 (m), 2974 (m), 2862 (m), 1682 (s), 1604 (s), 1422 (m), 1166 (m), 747 (w)

HRMS (ESI-MS): m/z calcd. for $C_{20}H_{30}N_2O_2$ [M+H^{+}]: 331.2386; found 331.2346

N-(4-(oxetan-3-ylidene)butyl)aniline (**S17**)

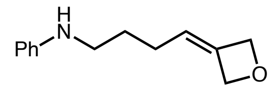

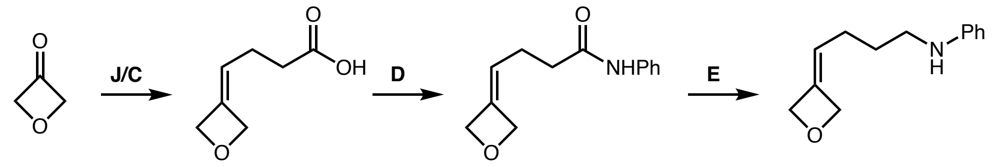

Synthesized according to Procedures J, C, D, and E.

^{1}H NMR (500 MHz, $CDCl_3$) δ 7.18 (tt, *J* = 7.4, 2.2 Hz, 2H), 6.70 (t, *J* = 7.3 Hz, 1H), 6.63 – 6.57 (m, 2H), 5.24 – 5.17 (m, 4H), 5.15 (t *J* = 7.0 Hz, 1H), 3.59 (s, 1H), 3.13 (t, *J* = 7.1 Hz, 2H), 2.04 – 1.90 (m, 2H), 1.69 (p, *J* = 7.2 Hz, 2H).

^{13}C NMR (126 MHz, $CDCl_3$) δ 148.28, 134.79, 129.29, 118.73, 117.37, 112.75, 79.44, 78.82, 43.40, 28.94, 25.81.

IR (neat, cm^{-1}): 3372 (m), 2927 (m), 2857 (m), 1602 (s), 1507 (s), 956 (w), 855 (w), 749 (w)

HRMS (ESI-MS): m/z calcd. for $C_{13}H_{17}NO$ [M+H^{+}]: 204.1388; found 204.1402

N-(2,2-dimethyl-4-(tetrahydro-4*H*-pyran-4-ylidene)butyl)aniline (**S18**)

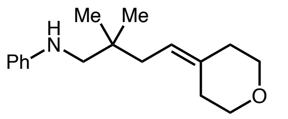

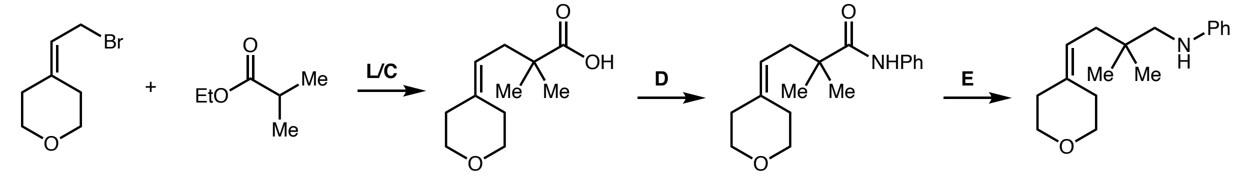

Synthesized according to Procedures L, C, D, and E.

^{1}H NMR (500 MHz, $CDCl_3$) δ 7.24 – 7.14 (m, 2H), 6.70 (t, *J* = 7.3 Hz, 1H), 6.64 (d, *J* = 7.7 Hz, 2H), 5.29 (t, *J* = 7.9 Hz, 1H), 3.73 – 3.67 (m, 2H), 3.64 (t, *J* = 5.5 Hz, 3H), 2.94 (s, 2H), 2.30 (t, *J* = 5.4 Hz, 2H), 2.26 (t, *J* = 5.3 Hz, 2H), 2.06 (d, *J* = 7.8 Hz, 2H), 1.00 (s, 6H).

^{13}C NMR (126 MHz, $CDCl_3$) δ 148.98, 136.48, 129.24, 119.16, 117.06, 112.68, 69.76, 68.70, 53.96, 37.40, 37.10, 35.25, 29.91, 25.47.

IR (neat, cm^{-1}): 3395 (m), 2959 (m), 2845 (m), 1604 (s), 1507 (m), 1321 (m), 1099 (m), 747 (s)

HRMS (ESI-MS): m/z calcd. for $C_{17}H_{25}NO$ [M+H$^+$]: 260.2014; found 260.1980

N-(5-methylhex-4-en-1-yl)aniline (**S19**)

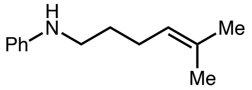

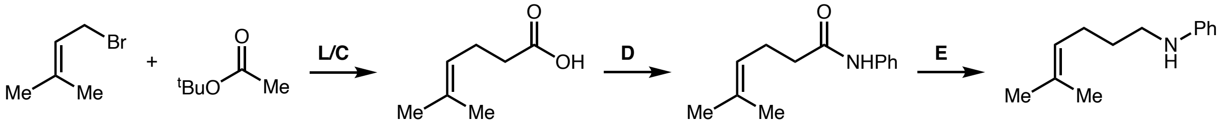

Synthesized according to Procedures L, C, D, and E.

^{1}H NMR (500 MHz, $CDCl_3$) δ 7.17 (t, *J* = 7.4, 2H), 6.72 – 6.65 (m, 1H), 6.60 (d, *J* = 8.5 2H), 5.15 (t, *J* = 7.2, 1H), 3.61 (s, 1H), 3.11 (t, *J* = 7.1 Hz, 2H), 2.10 (q, *J* = 7.3 Hz, 2H), 1.71 (s, 3H), 1.66 (p, *J* = 7.3 Hz, 2H), 1.62 (s, 3H).

^{13}C NMR (126 MHz, $CDCl_3$) δ 148.54, 132.29, 129.22, 123.79, 117.10, 112.73, 43.61, 29.61, 25.73, 17.73.

IR (neat, cm^{-1}): 3412 (m), 2927 (m), 2857 (m), 1604 (s), 1507 (m), 1321 (m), 1259 (m), 747 (w)

HRMS (ESI-MS): m/z calcd. for $C_{13}H_{19}N$ [M+H$^+$]: 190.1596; found 190.1587

(*E*)-*N*-(hex-4-en-1-yl)aniline (**S20**)

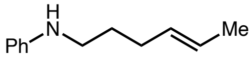

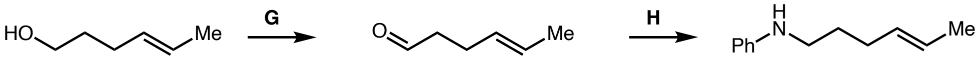

Synthesized according to Procedures G and H. Product **S20** was isolated by column chromatography (0-10% EtOAc/Hexanes). Yield: 423 mg (77%)

^{1}H NMR (500 MHz, $CDCl_3$) δ 7.21 – 7.14 (m, 2H), 6.69 (t, *J* = 7.3 Hz, 1H), 6.65 – 6.57 (m, 2H), 5.55 – 5.38 (m, 2H), 3.61 (s, 1H), 3.12 (t, *J* = 7.1 Hz, 2H), 2.11 (td, *J* = 7.3, 4.0 Hz, 2H), 1.73 – 1.61 (m, 5H).

^{13}C NMR (126 MHz, $CDCl_3$) δ 148.48, 130.53, 129.23, 125.6, 117.1, 112.7, 43.5, 30.2, 29.3, 17.9

IR (neat, cm^{-1}): 3418 (m), 3022 (m), 2857 (m), 1602 (s), 1505 (m), 1319 (m), 967 (m), 747 (w)

HRMS (ESI-MS): m/z calcd. for $C_{12}H_{17}N$ [M+H^{+}]: 176.1439; found 176.1432

(E)-N-(5-phenylpent-4-en-1-yl)aniline (**S21**)

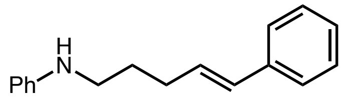

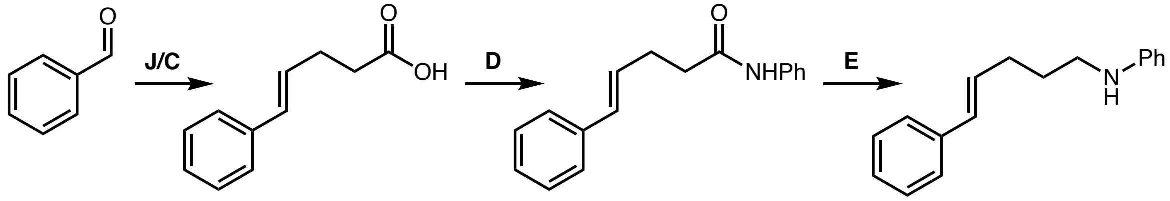

Synthesized according to Procedures J, C, D, and E.

^{1}H NMR (500 MHz, $CDCl_3$) δ 7.36 (t, J = 7.7 Hz, 2H), 7.30 (d, J = 7.9 Hz, 2H), 7.26 (d, J = 7.1 Hz, 1H), 7.19 (t, J = 7.8 Hz, 2H), 6.72 (t, J = 7.3 Hz, 1H), 6.59 (d, J = 8.4 Hz, 2H), 6.51 (d, J = 11.6 Hz, 1H), 5.71 (m, 1H), 3.76 (s, 1H), 3.18 (t, J = 7.0 Hz, 2H), 2.47 (q, J = 7.4 Hz, 2H), 1.80 (p, J = 7.2 Hz, 2H).

^{13}C NMR (126 MHz, $CDCl_3$) δ 148.12, 137.49, 131.85, 129.71, 129.24, 128.75, 128.24, 126.67, 117.31, 112.85, 43.40, 29.45, 26.01.

IR (neat, cm^{-1}): 3408 (m), 2926 (m), 2860 (m), 1600 (s), 1505 (s), 1317 (m), 1258 (m), 747 (w)

HRMS (ESI-MS): m/z calcd. for $C_{17}H_{19}N$ [M+H^{+}]: 238.1596; found 238.1582

N-(4-phenylpent-4-en-1-yl)aniline (**S22**)

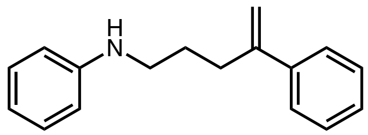

Synthesized according to literature procedure.[4]

^{1}H NMR (500 MHz, $CDCl_3$) δ 7.47 – 7.42 (m, 2H), 7.40 – 7.33 (m, 2H), 7.34 – 7.29 (m, 1H), 7.22 – 7.15 (m, 2H), 6.71 (t, J = 7.3 Hz, 1H), 6.60 (d, J = 7.7 Hz, 2H), 5.34 (s, 1H), 5.14 (s, 1H), 3.62 (s, 1H), 3.17 (t, J = 7.1 Hz, 2H), 2.67 (t, J = 7.3 Hz, 2H), 1.81 (p, J = 7.3 Hz, 2H).

^{13}C NMR (126 MHz, $CDCl_3$) δ 148.35, 147.83, 140.96, 129.24, 128.38, 127.50, 126.17, 117.19, 112.77, 112.72, 43.46, 32.88, 27.91.

IR (neat, cm^{-1}): 3410 (m), 2939 (m), 2864 (m), 1602 (s), 1505 (m), 1319 (m), 1258 (m), 896 (w)

HRMS (ESI-MS): m/z calcd. for $C_{17}H_{19}N$ [M+H^{+}]: 238.1596; found 238.1578

(*E*)-*N*-(5-(4-methoxyphenyl)pent-4-en-1-yl)aniline (**S23**)

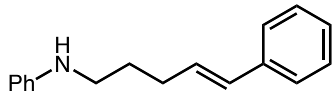

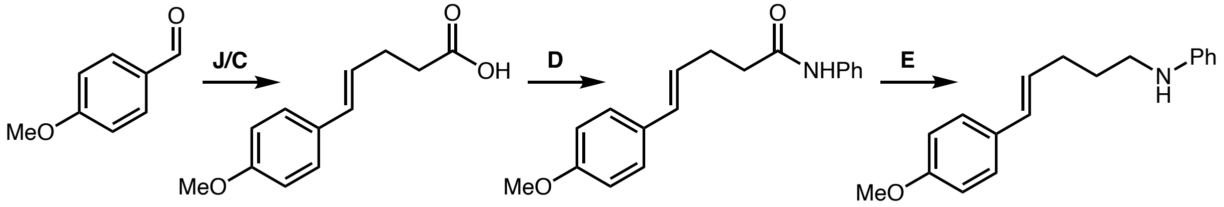

Synthesized according to Procedures J, C, D, and E.

^{1}H NMR (500 MHz, $CDCl_3$) δ 7.24 – 7.12 (m, 4H), 6.87 (d, *J* = 8.6 Hz, 2H), 6.69 (t, *J* = 7.3 Hz, 1H), 6.57 (d, *J* = 8.4 Hz, 2H), 6.41 (d, *J* = 11.6 Hz, 1H), 5.59 (dt, *J* = 11.6, 7.2 Hz, 1H), 3.82 (s, 3H), 3.63 (s, 1H), 3.16 (t, *J* = 7.0 Hz, 2H), 2.44 (qd, *J* = 7.4, 1.6 Hz, 2H), 1.78 (p, *J* = 7.2 Hz, 2H).

^{13}C NMR (126 MHz, $CDCl_3$) δ 158.80, 158.32, 148.28, 130.34, 130.16, 129.95, 129.89, 129.26, 129.24, 129.10, 127.73, 127.07, 117.18, 113.97, 113.66, 112.75, 55.29, 43.42, 43.37, 30.58, 29.57, 29.29, 26.05.

IR (neat, cm^{-1}): 3406 (m), 2935 (m), 2838 (m), 1604 (s), 1511 (m), 1246 (m), 1034 (m), 748 (w)

HRMS (ESI-MS): m/z calcd. for $C_{18}H_{21}NO$ [M+H$^+$]: 268.1701; found 268.1678

(*E*)-*N*-(5-(4-(trifluoromethyl)phenyl)pent-4-en-1-yl)aniline (**S24**)

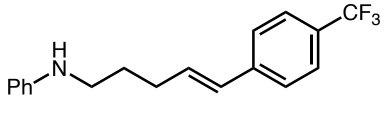

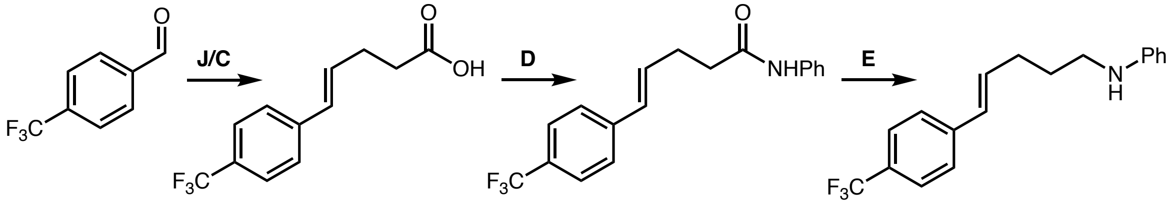

Synthesized according to Procedures J, C, D, and E.

^{1}H NMR (300 MHz, $CDCl_3$) δ 7.60 (d, J = 7.9 Hz, 2H), 7.4 (d, J = 7.9 Hz, 2H), 7.20 (m, 2H), 6.73 (m, 1H), 6.59 (m, 2H), 6.52 (d, J = 11.6 Hz, 1H), 5.82 (m, 1H), 3.60 (s, 1H), 3.18 (t, J = 7.1 Hz, 2H), 2.46 (t, J = 7.1 Hz, 2H), 1.81 (q, J = 7.1 Hz, 2H).

^{13}C NMR (126 MHz, $CDCl_3$) δ 148.31, 133.40, 129.49, 128.97, 125.12, 117.55, 112.74, 43.28, 30.62, 29.74, 28.99, 26.08.

IR (neat, cm^{-1}): 3419 (m), 2935 (m), 2864 (m), 1604 (s), 1507 (m), 1326 (m), 1121 (m), 1067(m)

HRMS (ESI-MS): m/z calcd. for $C_{18}H_{18}NF_3$ [M+H$^+$]: 306.1470; found 306.1481

(*E*)-*N*-(4-(dihydro-2*H*-pyran-3(4*H*)-ylidene)butyl)aniline (**S25**)

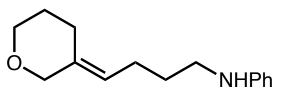

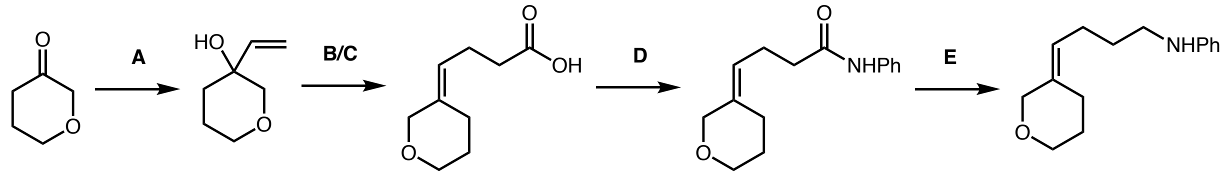

Synthesized according to Procedures A, B, C, D, and E. Isolated as a 1.7:1 mixture of E:Z olefins

^{1}H NMR (500 MHz, $CDCl_3$) δ 7.20 (d, *J* = 6.5 Hz, 2H), 6.74 – 6.68 (m, 1H), 6.62 (d, *J* = 8.6 Hz, 2H), 5.33 (d, *J* = 7.5 Hz, 1H, Z isomer), 5.25 (t, *J* = 8.0 Hz, 1H, E isomer), 4.17 (s, 2H, E isomer), 4.02 (s, 2H, Z isomer), 3.82 – 3.74 (m, 2H), 3.65 (s, 1H), 3.22 – 3.11 (m, 2H), 2.35 (t, *J* = 5.5 Hz, 2H), 2.34 – 2.27 (m, 2H), 2.16 (p, *J* = 7.2 Hz, 2H), 1.80 – 1.66 (m, 4H).

^{13}C NMR (126 MHz, $CDCl_3$) δ 148.41, 148.37, 135.27, 134.92, 129.24, 129.00, 123.60, 123.53, 117.21, 117.14, 112.74, 112.71, 74.48, 68.59, 68.51, 66.95, 43.46, 43.25, 33.23, 29.53, 29.42, 28.57, 27.72, 25.71, 24.41, 24.34.

IR (neat, cm^{-1}): 3369 (m), 2937 (m), 1603 (s), 15078 (m), 1080 (m)

HRMS (ESI-MS): m/z calcd. for $C_{15}H_{122}NO$ [M+H^{+}]: 232.1701; found 232.1716

(*E*)-*N*-(5-phenylhex-4-en-1-yl)aniline (**S26**)

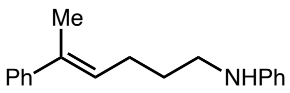

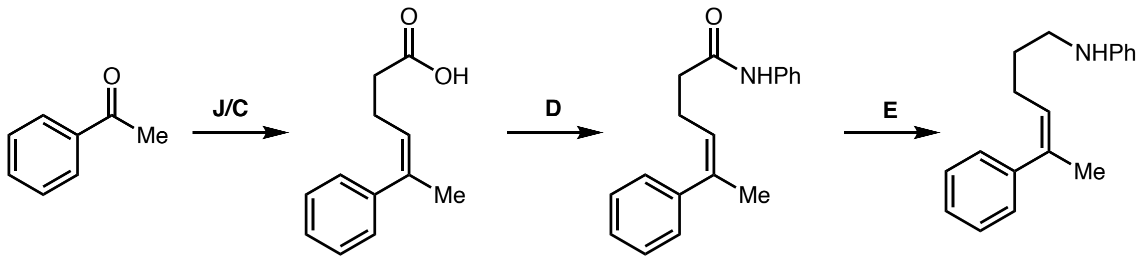

Synthesized according to Procedures J, C, D, and E. Isolated as a 98:2 mixture of E:Z isomers, NMR peaks are reported for the major isomer

^{1}H NMR (500 MHz, CDCl3) δ 7.36 (d, *J* = 7.6 Hz, 1H), 7.29 (d, *J* = 7.0 Hz, 2H), 7.23 – 7.11 (m, 4H), 6.69 (t, *J* = 7.3 Hz, 1H), 6.53 (d, *J* = 7.8 Hz, 2H), 5.50 (t, *J* = 7.1 Hz, 1H), 3.07 (t, *J* = 7.0 Hz, 2H), 2.11 (q, *J* = 7.2 Hz, 2H), 2.07 (s, 3H), 1.67 (p, *J* = 7.1 Hz, 2H).

^{13}C NMR (126 MHz, $CDCl_3$) δ 148.28, 142.02, 137.27, 129.18, 128.18, 127.94, 126.62, 126.59, 117.22, 117.07, 112.75, 112.73, 77.28, 77.03, 76.78, 43.11, 29.45, 26.37, 25.65.

IR (neat, cm^{-1}): 3412 (m), 2916 (m), 1600 (s), 1506 (s), 1258 (m)

HRMS (ESI-MS): m/z calcd. for $C_{18}H_{22}N$ [M+H^{+}]: 252.1752; found 252.1798

F. Racemic standard synthesis

General Procedures. Characterization of intermediates is reported following the respective procedure. The reaction sequence used for each standard and characterization data for each standard is reported beginning on page S43.

Procedure M: Metallophotoredox cross coupling
Carried out according to literature procedure.[5]

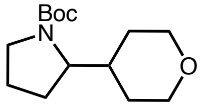

S2m: From N-Boc-L-proline and 4-bromotetrahydropyran. Purified by column chromatography (0-50% EtOAc/Hexanes) ^{1}H-NMR (500 MHz, $CDCl_3$): δ 4.06-3.93 (m, 2H), 3.82-3.63 (m, 1H), 3.59-3.27 (m, 3H), 3.25-3.18 (m, 1H), 1.88-1.72 (m, 5H), 1.52-1.47 (m, 1H), 1.47-1.45 (s, 9H), 1.44-1.28 (m, 3H)

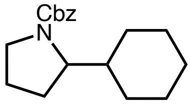

S13m: From N-Cbz-L-proline and bromocyclohexane. Purified by column chromatography (0-20% EtOAc/Hexanes). ^{1}H-NMR (500 MHz, CDCl3): δ 7.39 (m, 5H), 5.20 (m, 2H), 3.67 – 3.48 (m, 1H), 3.47 – 3.36 (m, 1H), 3.36–3.27 (m, 1H), 1.90 – 0.83 (m, 15H).

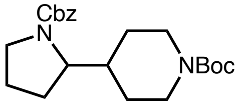

S16m: From N-Cbz-L-proline and 4-bromo-N-Boc-piperidine. Purified by column chromatography (0-40% EtOAc/Hexanes). ^{1}H NMR (300 MHz, $CDCl_3$) δ 7.54 – 7.32 (m, 5H), 5.30 – 5.03 (m, 2H), 4.14 (m, 2H), 3.86 (m, 1H), 3.54 (m, 1H), 3.38 (m, 1H), 2.64 (s, 2H), 1.83 (m, 5H), 1.47 (s, 9H), 1.20 (s, 2H).

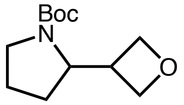

S17m: From N-Boc-L-proline and 3-bromooxetane. Purified by column chromatography (0-40% EtOAc/Hexanes). ^{1}H NMR (400 MHz, $CDCl_3$) δ 4.82 (s, 1H), 4.67 (m, 2H), 4.50 (t, J = 6.7 Hz, 1H), 3.61 – 3.37 (m, 2H), 3.37 – 3.15 (m, 2H), 2.00 (m, 2H), 1.95 – 1.77 (m, 2H), 1.55 (s, 9H).

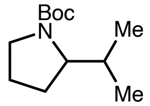

S19m: From N-Boc-L-proline and 2-bromopropane. Purified by column chromatography (0-40% EtOAc/Hexanes). ^{1}H-NMR (300 MHz, $CDCl_3$): δ 5.19 – 5.06 (m, 2H), 3.84 – 3.72 (m, 1H), 3.68 – 3.51 (m, 1H,), 3.35 – 3.26 (m, 1H), 2.29 – 2.04 (m, 1H), 1.91 – 1.68 (m, 4H), 1.55 (s, 9H) 0.97 – 0.74 (m, 6H).

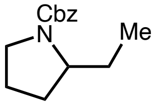

S20m: From N-Cbz-L-proline and bromoethane. Purified by column chromatography (0-40% EtOAc/Hexanes). ^{1}H NMR (500 MHz, $CDCl_3$) δ 7.45 – 7.30 (m, 5H), 5.15 (m, 2H), 3.81 (s, 1H), 3.57 – 3.33 (m, 2H), 1.99 – 1.79 (m, 2H), 1.81 – 1.63 (m, 2H), 1.45 – 1.24 (m, 2H), 0.96 –0.82 (m, 3H).

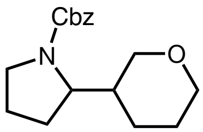

S25m: From N-Boc-L-proline and 3-bromotetrahydropyran. Purified by column chromatography (0-40% EtOAc/Hexanes). Mixture of diasteromers and rotamers. ^{1}H NMR (500 MHz, $CDCl_3$) δ 7.35 (m, *5* H), 5.15 (m, 2H), 4.03 – 2.86 (m, 7H), 1.84 (m, 6H), 1.62 (s, 2H).

Procedure N: Boc Deprotection

Protected amine (1 mmol, 1 equiv.) was dissolved in DCM (8 mL) and the solution was cooled to 0 °C. Trifluoroacetic acid (2 mL) was added dropwise and the mixture was warmed to room temperature and stirred for 1 h. After 1 h, 2 M NaOH (100 mL) was added and the solution was extracted with EtOAc (3 x 100 mL). The organic extracts were dried with Na_2SO_4 and concentrated in vacuo to obtain the deprotected amine which was directed subjected to arylation (procedure P).

Procedure O: Cbz deprotection:

Cbz protected amine (0.5 mmol, 1 equiv) was dissolved in MeOH (1 mL) and Pd/C (10 wt%) was added. The suspension was stirred for 5 min, filtered through Celite, and evaporated to dryness to obtain the deprotected amine which was directed subjected to arylation (procedure P).

Procedure P: Buchwald-Hartwig cross coupling.

To a flame-dried flask equipped with a stir bar were added $Pd_2(dba)_3$ (0.05 equiv), rac-BINAP (0.1 equiv), KO^{t}Bu (2 equiv.), and dry, degassed toluene (5 mL). The solution was stirred for 5 min and then amine (0.3 mmol, 1 equiv.) and aryliodide (1.5 equiv) were added. The flask was sealed and heated to 110 °C for 16 h or until TLC analysis indicated full conversion of the amine. The solution was cooled to room temperature, diluted with THF (10 mL), filtered through Celite, and evaporated to dryness. The crude reaction mixture was purified by silica column chromatography and/or reverse-phase preparatory HPLC.

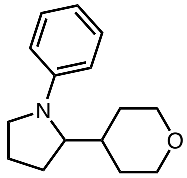

2: From **S2m** and iodobenzene. Purified by silica gel column chromatography (0-10% EtOAc/hexanes). Yield: 122 mg (71%).

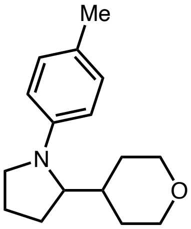

3: From **S2m** and 4-iodotoluene. Purified by silica gel column chromatography (0-10% EtOAc/hexanes). Yield: 74 mg (63%).

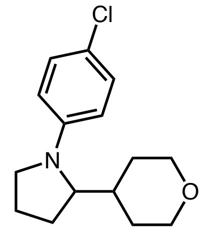

4: From **S2m** and 1-iodo-4-chlorobenzene. Purified by silica gel column chromatography (0-10% EtOAc/hexanes) and preparative HLPC. Yield: 16 mg (20%).

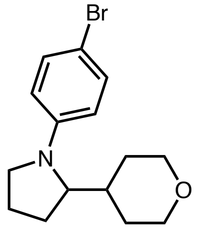

5: From **S2m** and 1-iodo-4-bromobenzene. Purified by silica gel column chromatography (0-100% DCM/hexanes) and preparative HLPC. Yield: 21 mg (20%).

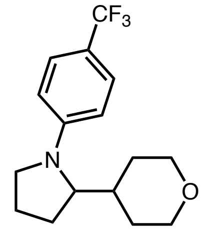

6: From **S2m** and 1-iodo-4-(trifluoromethyl)benzene. Purified by silica gel column chromatography (0-100% DCM/hexanes) and preparative HLPC. Yield: 14 mg (12%).

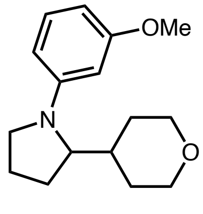

7: From **S2m** and 1-iodo-3-methoxybenzene. Purified by silica gel column chromatography (0-20% EtOAc/hexanes) and preparative HLPC. Yield: 24 mg (24%).

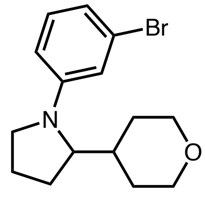

8: From **S2m** and 1-iodo-3-bromobenzene. Purified by silica gel column chromatography (0-100% DCM/hexanes) and preparative HLPC. Yield: 15 mg (14%).

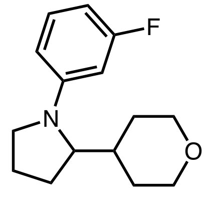

9: From **S2m** and 1-iodo-3-fluorobenzene. Purified by silica gel column chromatography (0-10% EtOAc/hexanes) and preparative HLPC. Yield: 31 mg (19%).

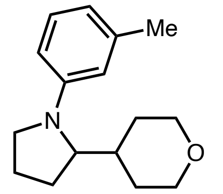

10: From **S2m** and 1-iodo-3-methylbenzene. Purified by silica gel column chromatography (0-12% EtOAc/hexanes). Yield: 16 mg (20%).

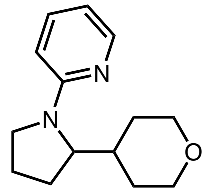

11: From **S2m** and 2-iodopyridine. Purified by silica gel column chromatography (0-8% MeOH/DCM) and preparative HLPC. Yield: 7 mg (8%).

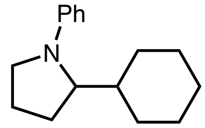

13: From deprotected **S13m** and iodobenzene. Purified by silica gel column chromatography (0-100% DCM/hexanes). Yield: 35 mg (62%).

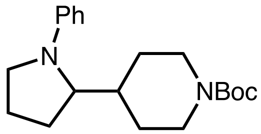

16: From deprotected **S16m** and iodobenzene. Purified by silica gel column chromatography (0-20% EtOAc/hexanes). Yield: 21 mg (48%).

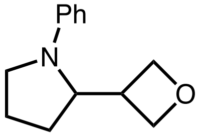

17: From deprotected **S17m** and iodobenzene. Purified by silica gel column chromatography (0-100% DCM/hexanes). Yield: 100 mg (49%).

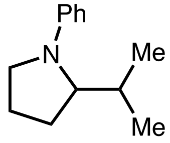

19: From deprotected **S19m** and iodobenzene. Purified by silica gel column chromatography (0-100% DCM/hexanes). Yield: 73 mg (52%).

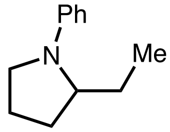

20: From deprotected **S20m** and iodobenzene. Purified by silica gel column chromatography (0-100% DCM/hexanes). Yield: 32 mg (83%).

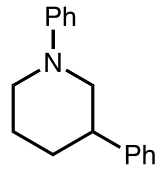

22: From commercial 3-phenylpiperidine and iodobenzene. Yield 129 mg (73%).

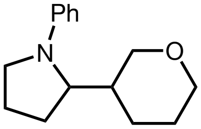

25: From deprotected **S25m** and iodobenzene. Purified by silica gel column chromatography (0-100% DCM/hexanes). Yield: 12 mg (83%).

1-phenyl-2-(tetrahydro-2*H*-pyran-4-yl)pyrrolidine (**2**)

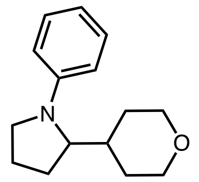

Synthesized according to Procedures M, N, and P.

^{1}H NMR (300 MHz, $CDCl_3$) δ 7.25 (d, J = 8.3 Hz, 1H), 6.76 – 6.63 (m, 2H), 4.09 – 3.95 (m, 2H), 3.75 – 3.66 (m, 1H), 3.53 (m, 1H), 3.47 – 3.12 (m, 3H), 2.14 – 1.83 (m, 5H), 1.50 (m, 4H).

^{13}C NMR (126 MHz, $CDCl_3$) δ 147.93, 129.65, 115.82, 112.51, 68.45, 62.19, 49.46, 37.91, 30.25, 27.88, 26.74, 24.41.

IR (neat, cm^{-1}): 2953 (m), 2931 (m), 2851 (m), 1507 (m), 1325 (m), 1099 (m), 749 (w), 693 (w)

HRMS (ESI-MS): m/z calcd. for $C_{15}H_{21}NO$ [M+H$^+$]: 232.1701; found 232.1709

Enantioenriched (*R*)-**2** was synthesized according to literature procedure[6] followed by procedures N and O for determination of the absolute stereochemistry of the enzymatic product. The enzymatic product is (*S)*-configured.

2-(tetrahydro-2*H*-pyran-4-yl)-1-(*p*-tolyl)pyrrolidine (**3**)

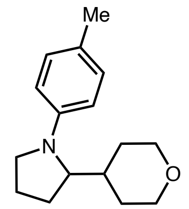

Synthesized according to Procedures M, N, and P.

^{1}H NMR (500 MHz, $CDCl_3$) δ 7.04 (d, *J* = 8.3 2H), 6.54 (d, *J* = 8.3, 2H), 4.05 – 3.93 (m, 2H), 3.67 – 3.60 (m, 1H), 3.54 – 3.46 (m, 1H), 3.36 (td, *J* = 11.5, 2.4 Hz, 1H), 3.25 (td, *J* = 11.6, 2.4 Hz, 1H), 3.17 – 3.08 (m, 1H), 2.25 (s, 3H), 2.04 – 1.77 (m, 5H), 1.61 – 1.33 (m, 5H).

^{13}C NMR (126 MHz, $CDCl_3$) δ 146.10, 129.80, 124.81, 112.45, 68.47, 68.42, 62.42, 49.89, 38.17, 30.40, 27.88, 26.89, 24.49, 20.34.

IR (neat, cm^{-1}): 1620 (m), 1519 (s), 1333 (m), 1232 (m), 798 (s)

HRMS (ESI-MS): m/z calcd. for $C_{16}H_{23}NO$ [$M+H^+$]: 246.1858; found 246.1857

1-(4-chlorophenyl)-2-(tetrahydro-2*H*-pyran-4-yl)pyrrolidine (**4**)

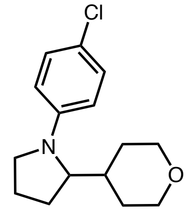

Synthesized according to Procedures M, N, and P.

^{1}H NMR (500 MHz, $CDCl_3$) δ 7.15 (d, *J* = 9.0 Hz, 2H), 6.52 (d, *J* = 9.0 Hz, 2H), 4.03 – 3.94 (m, 2H), 3.67 – 3.61 (m, 1H), 3.49 – 3.42 (m, 1H), 3.40 – 3.32 (m, 1H), 3.30 – 3.21 (m, 1H), 3.17 – 3.10 (m, 1H), 2.04 – 1.83 (m, 5H), 1.55 – 1.36 (m, 4H).

^{13}C NMR (126 MHz, $CDCl_3$) δ 146.57, 129.01, 120.49, 113.48, 68.38, 68.31, 62.47, 49.74, 37.96, 30.33, 27.86, 26.93, 24.40.

IR (neat, cm^{-1}): 1597 (m), 1497 (s), 1364 (m), 1234 (m), 805 (s)

HRMS (ESI-MS): m/z calcd. for $C_{15}H_{20}NOCl$ [M+H^{+}]: 266.1306; found 266.1304.

1-(4-bromophenyl)-2-(tetrahydro-2*H*-pyran-4-yl)pyrrolidine (**5**)

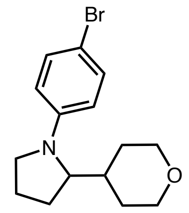

Synthesized according to Procedures M, N, and P.

^{1}H NMR (500 MHz, $CDCl_3$) δ 7.30 – 7.27 (m, 2H), 6.50 – 6.45 (m, 2H), 4.03 – 3.94 (m, 2H), 3.66 – 3.61 (m, 1H), 3.48 – 3.42 (m, 1H), 3.39 – 3.32 (m, 1H), 3.30 – 3.21 (m, 1H), 3.16 – 3.09 (m, 1H), 2.06 – 1.83 (m, 5H), 1.53 – 1.35 (m, 4H).

^{13}C NMR (126 MHz, $CDCl_3$) δ 146.92, 131.87, 114.06, 107.52, 68.37, 68.31, 62.43, 49.66, 37.91, 30.33, 27.87, 26.93, 24.38.

IR (neat, cm^{-1}): 1590 (m), 1493 (m), 1349 (m), 803 (s).

HRMS (ESI-MS): m/z calcd. for $C_{15}H_{20}NOBr$ [M+H^{+}]: 310.0801; found, 310.0794.

2-(tetrahydro-2*H*-pyran-4-yl)-1-(4-(trifluoromethyl)phenyl)pyrrolidine (**6**)

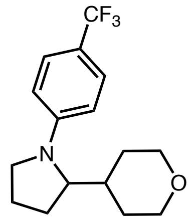

Synthesized according to Procedures M, N, and P.

^{1}H NMR (500 MHz, $CDCl_3$) δ 7.44 (d, *J* = 8.6 Hz, 2H), 6.51 (d, *J* = 8.6 Hz, 2H), 4.04 – 3.95 (m, 2H), 3.77 – 3.72 (m, 1H), 3.53 – 3.46 (m, 1H), 3.40 – 3.33 (m, 1H), 3.30 – 3.18 (m, 2H), 2.08 – 1.86 (m, 5H), 1.53 – 1.38 (m, 4H).

^{13}C NMR (126 MHz, $CDCl_3$) δ 149.93, 126.53 (q, *J* = 3.8 Hz), 117.35, 117.09, 111.78, 68.33, 68.25, 62.36, 49.38, 37.83, 30.30, 27.91, 26.89, 24.22

^{19}F NMR: (300 MHz, $CDCl_3$): δ -60.75.

IR (neat, cm^{-1}): 1612 (s), 1528 (m), 1321 (s), 1098 (s), 1068 (s), 816 (s).

HRMS (ESI-MS): m/z calcd. for $C_{16}H_{21}NOF_3$ [M+H$^+$]: 300.1570; found, 300.1573.

1-(3-methoxyphenyl)-2-(tetrahydro-2*H*-pyran-4-yl)pyrrolidine (**7**)

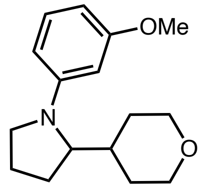

Synthesized according to Procedures M, N, and P.

^{1}H NMR (500 MHz, $CDCl_3$) δ 7.14 (t, *J* = 8.2 Hz, 1H), 6.26 (dt, *J* = 8.2, 2.3 Hz, 2H), 6.17 (t, *J* = 2.3 Hz, 1H), 4.05 – 3.93 (m, 2H), 3.81 – 3.79 (s, 3H), 3.70 – 3.64 (m, 1H), 3.52 – 3.44 (m, 1H), 3.41 – 3.32 (m, 1H), 3.30 – 3.22 (m, 1H), 3.22 – 3.12 (m, 1H), 2.08 – 1.81 (m, 5H), 1.54 – 1.36 (m, 4H).

^{13}C NMR (126 MHz, $CDCl_3$) δ 160.85, 149.40, 129.93, 105.86, 100.43, 99.14, 68.43, 68.38, 62.45, 55.27, 49.65, 38.03, 30.37, 27.90, 26.85, 24.40.

IR (neat, cm^{-1}): 1608 (m), 1497 (m), 1364 (m), 1223 (s), 1169 (s), 824 (m), 755 (s), 691 (m)

HRMS (ESI-MS): m/z calcd. for $C_{16}H_{23}NO_2$ [M+H^{+}]: 262.1802; found, 262.1806.

1-(3-bromophenyl)-2-(tetrahydro-2*H*-pyran-4-yl)pyrrolidine (**8**)

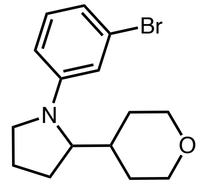

Synthesized according to Procedures M, N, and P.

^{1}H NMR (500 MHz, $CDCl_3$) δ 7.05 (t, *J* = 8.1 Hz, 1H), 6.79 – 6.75 (m, 1H), 6.72 (t, *J* = 2.3 Hz), 6.52 (dd, *J* = 8.1, 2.3 Hz, 1H), 4.03 – 3.95 (m, 2H), 3.68 – 3.63 (m, 1H), 3.48-3.41 (m, 1H), 3.41 – 3.33 (m, 1H), 3.31 – 3.22 (m, 1H), 3.20 – 3.11 (m, 1H), 2.03 – 1.83 (m, 5H), 1.53 – 1.36 (m, 4H).

^{13}C NMR (126 MHz, $CDCl_3$) δ 149.17, 130.41, 123.54, 118.51, 115.19, 111.12, 68.36, 68.28, 62.35, 49.57, 37.93, 30.29, 27.91, 26.90, 24.27.

IR (neat, cm^{-1}): 1588 (s), 1480 (s) 1364 (m), 1090 (s), 982 (s), 755 (s), 682 (m).

HRMS (ESI-MS): m/z calcd. for $C_{15}H_{20}NOBr$ [$M+H^{+}$]: 310.0801; found, 310.0804

1-(3-fluorophenyl)-2-(tetrahydro-2*H*-pyran-4-yl)pyrrolidine (**9**)

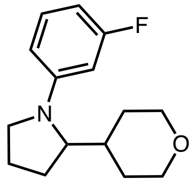

Synthesized according to Procedures M, N, and P.

^{1}H NMR (500 MHz, $CDCl_3$) δ 7.14 (td, *J* = 8.2, 7.0 Hz, 1H), 6.39 – 6.33 (m, 2H), 6.29 (dt, *J* = 12.7, 2.4 Hz, 1H), 4.04 – 3.94 (m, 2H), 3.68 – 3.61 (m, 1H), 3.49 – 3.42 (m, 1H), 3.41 – 3.43 (m, 1H), 3.31 – 3.22 (m, 1H), 3.20 – 3.12 (m, 1H), 2.06 – 1.84 (m, 5H), 1.54 – 1.37 (m, 4H).

^{13}C NMR (126 MHz, $CDCl_3$) δ 164.23 (d, *J* = 242 Hz), 149.66 (d, *J* = 10.8 Hz), 130.20 (d, *J* = 10.5 Hz), 108.21 (d, *J* = 2.1 Hz), 102.22 (d, *J* = 21.7 Hz), 99.38 (d, *J* = 25.8 Hz), 68.37, 68.32, 62.59, 49.66, 37.91, 30.31, 27.87, 26.87, 24.32.

^{19}F NMR: (400 MHz, $CDCl_3$): δ -112.77.

IR (neat, cm^{-1}): 1614 (s), 1497 (s), 1364 (m), 1157 (m), 1012 (s), 818 (m), 749 (s), 682 (m)

HRMS (ESI-MS): m/z calcd. for $C_{15}H_{20}NOF$ [M+H^{+}]: 250.1602; found, 250.1597

2-(tetrahydro-2*H*-pyran-4-yl)-1-(*m*-tolyl)pyrrolidine (**10**)

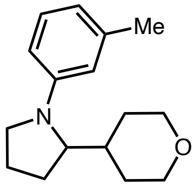

Synthesized according to Procedures M, N, and P.

^{1}H NMR (500 MHz, $CDCl_3$) δ 7.18 – 7.09 (m, 2H), 7.03 (d, J = 7.4 Hz, 1H), 6.90 (t, J = 7.4 Hz, 1H), 3.94 – 3.88 (m, 2H), 3.70 – 3.64 (m, 1H), 3.51 – 3.45 (m, 1H), 3.31 – 3.24 (m, 1H), 3.23 – 3.16 (m, 1H), 2.76 – 2.69 (m, 1H), 2.26 (s, 3H), 2.00 – 1.91 (m, 1H), 1.90-1.69 (m, 4H), 1.49-1.31 (m, 4H).

^{13}C NMR (126 MHz, $CDCl_3$) δ 149.32, 132.56, 131.42, 126.27, 121.72, 118.84, 68.45, 68.38, 63.13, 54.87, 37.25, 30.76, 26.95, 24.59, 19.49.

IR (neat, cm^{-1}): 1599 (m), 1491 (s), 1232 (m), 1092 (s), 982 (m), 757 (s), 721 (m)

HRMS (ESI-MS): m/z calcd. for $C_{15}H_{21}NO$ [M+H^{+}]: 246.1852; found, 246.1857

4-(2-(tetrahydro-2*H*-pyran-4-yl)pyrrolidin-1-yl)pyridine (**11**)

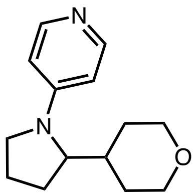

Synthesized following a modification of a literature procedure beginning from deprotected **S2m**.[7] The product was isolated using reverse phase preparative HPLC. Yield: 1.5 mg (3%).

^{1}H NMR (500 MHz, $CDCl_3$) δ 8.23 (d, J = 6.4 Hz, 2H), 6.45 (d, J = 6.4 Hz, 2H), 4.07 – 3.98 (m, 2H), 3.80 – 3.74 (m, 1H), 3.47 (m, 1H), 3.39 (td, J = 11.3, 3.2 Hz, 1H), 3.35 – 3.22 (m, 2H), 2.13 – 2.04 (m, 2H), 2.04 – 1.88 (m, 4H), 1.56 – 1.41 (m, 4H).

^{13}C NMR (126 MHz, $CDCl_3$) δ 158.10, 147.01, 131.56, 130.28, 129.33, 115.42, 113.84, 111.80, 59.88, 55.27, 48.40, 37.64, 29.47, 23.04.

IR (neat, cm^{-1}): 2922, 2842, 1595, 1439, 1374, 1094, 995

HRMS (ESI-MS): m/z calcd. for $C_{14}H_{20}N_2O$ [M+H$^+$]: 233.1648, found: 233.1648

2-(2-(tetrahydro-2*H*-pyran-4-yl)pyrrolidin-1-yl)pyridine (**12**)

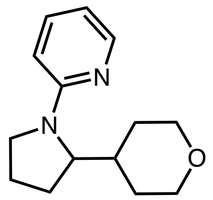

Synthesized according to Procedures M, N, and P.

^{1}H NMR (500 MHz, $CDCl_3$) δ 8.15 (ddd, *J* = 5.0, 2.0, 0.9 Hz, 1H), 7.42 (ddd, *J* = 8.5, 7.0, 2.0 Hz, 1H), 6.52 (ddd, *J* = 7.0, 5.0, 0.9 Hz, 1H), 6.38 (d, *J* = 8.5 Hz, 1H), 4.10 – 4.03 (m, 1H), 4.02 – 3.94 (m, 2H), 3.53 – 3.46 (m,1H), 3.44 – 3.33 (m, 2H), 3.33 – 3.23 (m, 1H), 2.22 – 2.12 (m, 1H), 2.06 – 1.84 (m, 4H), 1.75 – 1.62 (br s, <1H), 1.54 – 1.37 (m, 4H).

^{13}C NMR (126 MHz, $CDCl_3$) δ 157.92, 148.26, 136.95, 111.56, 107.11, 68.48, 68.38, 61.35, 48.40, 38.02, 30.31, 28.05, 26.82, 24.37.

IR (neat, cm^{-1}): 2932, 2842, 1703, 1597, 1478, 1439, 1375, 1092

HRMS (ESI-MS): m/z calcd. for $C_{14}H_{20}N_2O$ [M+H$^+$]: 233.1648; found, 233.1650

2-cyclohexyl-1-phenylpyrrolidine (**13**)

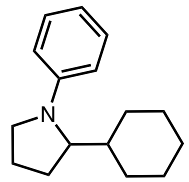

Synthesized according to Procedures M, O, and P.

^{1}H NMR (500 MHz, $CDCl_3$) δ 7.23 (dd, *J* = 8.6, 7.3 Hz, 2H), 6.65 (t, *J* = 7.2 Hz, 1H), 6.61 (d, *J* = 8.0 Hz, 2H), 3.67 – 3.59 (m, 1H), 3.49 (td, *J* = 8.6, 2.5 Hz, 1H), 3.15 (q, *J* = 8.7 Hz, 1H), 2.06–1.48 (m, 10H), 1.33 – 0.91 (m, 5H).

^{13}C NMR (126 MHz, $CDCl_3$) δ 148.46, 129.43, 115.51, 112.56, 63.39, 49.74, 40.83, 30.87, 27.98, 27.18, 26.79, 24.81.

IR (neat, cm^{-1}): 2924 (m), 2853 (m), 1599 (m), 1505 (m), 1364 (m), 1241 (m), 993 (w), 745 (w)

HRMS (ESI-MS): m/z calcd. for $C_{16}H_{23}N$ [$M+H^+$]: 230.1909; found 230.1996

4-(1-phenylpyrrolidin-2-yl)piperidine (**14**)

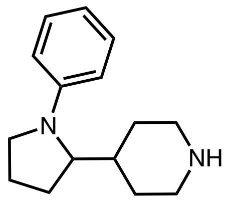

Prepared by deprotection of **16** according to Procedure N. Yield: 68 mg (95%).

^{1}H NMR (500 MHz, $CDCl_3$) δ 7.22 (t *J* = 7.3, 7.9 Hz, 2H), 6.67 (t, *J* = 7.3 Hz, 1H), 6.61 (d, *J* = 7.9 Hz, 2H), 3.70 – 3.61 (m, 1H), 3.53 – 3.45 (m, 1H), 3.19 – 3.06 (m, 3H), 2.88 (s, 1H), 2.60 (td, *J* = 12.1, 2.7 Hz, 1H), 2.48 (td, *J* = 12.1, 2.6 Hz, 1H), 2.04 – 1.79 (m, 5H), 1.60 (m, 2H), 1.30 (m, 2H).

^{13}C NMR (126 MHz, $CDCl_3$) δ 147.94, 128.90, 115.24, 112.00, 62.30, 49.37, 46.84, 46.64, 38.96, 30.25, 27.49, 26.77, 24.35.

IR (neat, cm^{-1}): 2924 (m), 2849 (m), 1597 (m), 1504 (m), 1362 (m), 1159 (m), 993 (w), 747 (w)

HRMS (ESI-MS): m/z calcd. for $C_{16}H_{22}N_2$ [$M+H^+$]: 231.1861; found 231.1861

1-methyl-4-(1-phenylpyrrolidin-2-yl)piperidine (**15**)

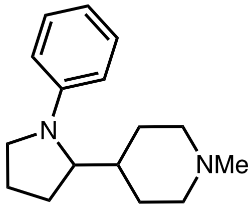

Prepared by reduction of **16** according to Procedure E. Purified by silica gel column chromatography (0-100% MeOH/DCM + 1% TEA). Yield: 34 mg (82%).

^{1}H NMR (500 MHz, $CDCl_3$) δ 7.24 (tt, *J* = 7.3, 2.2 Hz, 2H), 6.69 (t, *J* = 7.3 Hz, 1H), 6.63 (d, *J* = 8.0 Hz, 2H), 3.75 – 3.69 (m, 1H), 3.52 (m, 1H), 3.22 – 3.13 (m, 1H), 3.01 – 2.89 (m, 2H), 2.30 (s, 3H), 2.08 – 1.73 (m, 8H), 1.70 – 1.39 (m, 4H).

^{13}C NMR (126 MHz, $CDCl_3$) δ 147.93, 128.89, 115.47, 112.00, 61.92, 56.00, 55.85, 49.28, 46.22, 37.99, 29.45, 26.79, 26.69, 24.37.

IR (neat, cm^{-1}): 2953 (m), 2844 (m), 2780 (m), 1599 (m), 1505 (m), 1364 (m), 1159 (w), 747 (w)

HRMS (ESI-MS): m/z calcd. for $C_{16}H_{24}N_2$ [$M+H^+$]: 245.2018; found 245.2047

tert-butyl 4-(1-phenylpyrrolidin-2-yl)piperidine-1-carboxylate (**16**)

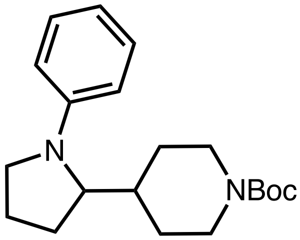

Synthesized according to Procedures M, O, and P.

^{1}H NMR (500 MHz, $CDCl_3$) δ 7.23 (t, *J* = 7.4, 2H), 6.68 (t, *J* = 7.4 Hz, 1H), 6.61 (d, *J* = 8.0 Hz, 2H), 4.16 (s, 2H), 3.74 – 3.65 (m, 1H), 3.50 (m, 1H), 3.16 (q, *J* = 8.5 Hz, 1H), 2.64 (t, *J* = 10.8 Hz, 1H), 2.52 (t, *J* = 10.9 Hz, 1H), 2.06 – 1.79 (m, 5H), 1.67 – 1.49 (m, 2H), 1.46 (s, 9H), 1.26 (m, 2H).

^{13}C NMR (126 MHz, $CDCl_3$) δ 154.83, 147.90, 129.17, 115.64, 112.51, 79.33, 62.07, 49.33, 44.08, 39.07, 29.42, 28.34, 26.82, 24.32.

IR (neat, cm^{-1}): 2972 (m), 2931 (m), 2853 (m), 1690 (m), 1597 (m), 1504 (m), 1159 (m), 747 (w)

HRMS (ESI-MS): m/z calcd. for $C_{20}H_{30}N_2O_2$ [$M+H^+$]: 331.2386; found 331.2336

2-(oxetan-3-yl)-1-phenylpyrrolidine (**17**)

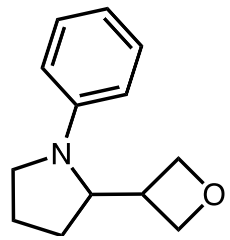

Synthesized according to Procedures M, N, and P.

^{1}H NMR (500 MHz, $CDCl_3$) δ 7.25 (t, *J* = 7.9 Hz, 2H), 6.72 (t, *J* = 7.3 Hz, 1H), 6.67 (d, *J* = 8.2 Hz, 2H), 4.80 – 4.61 (m, 3H), 4.55 (t, *J* = 6.4 Hz, 1H), 4.10 (t, *J* = 5.8 Hz, 1H), 3.55 (dt, *J* = 10.3, 5.5 Hz, 1H), 3.42 (h, *J* = 6.8 Hz, 1H), 3.25 – 3.15 (m, 1H), 2.12 – 1.97 (m, 4H).

^{13}C NMR (126 MHz, $CDCl_3$) δ 148.15, 129.18, 116.30, 112.52, 74.96, 74.14, 58.78, 49.71, 39.05, 28.03, 23.90.

IR (neat, cm^{-1}): 2959 (m), 2873 (m), 1597 (m), 1504 (m), 1359 (m), 1159 (m), 976 (w), 747 (w)

HRMS (ESI-MS): m/z calcd. for $C_{13}H_{17}NO$ [M+H$^+$]: 204.1388; found 204.1364

4,4-dimethyl-1-phenyl-2-(tetrahydro-2*H*-pyran-4-yl)pyrrolidine (**18**)

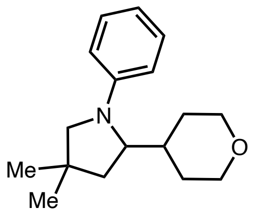

Synthesized according to modification of literature procedure[8] (added 10 mol% PhSH) from **S18** (100 mg, 0.38 mmol) and isolated by preparative HPLC. Yield: 18 mg, (18%)

^{1}H NMR (500 MHz, $CDCl_3$) δ 7.23 (dd, *J* = 8.6, 7.3 Hz, 2H), 6.67 (t, *J* = 7.3 Hz, 1H), 6.61 (d, *J* = 8.0 Hz, 2H), 4.06 – 3.88 (m, 3H), 3.38 (td, *J* = 11.7, 2.4 Hz, 1H), 3.25 (td, *J* = 11.7, 2.2 Hz, 1H), 3.17 (s, 2H), 2.35 (tq, *J* = 12.0, 3.9 Hz, 1H), 1.73 (dt, *J* = 8.4, 4.7 Hz, 2H), 1.54 – 1.23 (m, 4H), 1.18 (s, 3H), 0.96 (s, 3H).

^{13}C NMR (126 MHz, $CDCl_3$) δ 147.97, 129.00, 115.42, 112.72, 68.21, 68.15, 63.90, 60.13, 40.76, 37.45, 35.46, 29.51, 27.09, 26.17, 25.30.

IR (neat, cm^{-1}): 2955 (m), 2842 (m), 1597 (m), 1504 (m), 1364 (m), 1142 (m), 1097 (m), 747 (w)

HRMS (ESI-MS): m/z calcd. for $C_{17}H_{25}NO$ [M+H^{+}]: 260.2014; found 260.2021

2-isopropyl-1-phenylpyrrolidine (**19**)

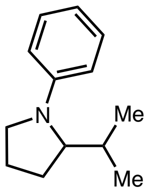

Synthesized according to Procedures M, N, and P.

^{1}H NMR (500 MHz, $CDCl_3$) δ 7.26 (tt, J = 7.2, 2.2 Hz, 2H), 6.69 (t, J = 7.3 Hz, 1H), 6.65 (d, J = 8.0 Hz, 2H), 3.70 (dt, J = 7.5, 3.6 Hz, 1H), 3.58 – 3.51 (m, 1H), 3.23 (q, J = 8.5 Hz, 1H), 2.25 (m, 1H), 2.10 – 1.82 (m, 4H), 0.98 (d, J = 7.0 Hz, 3H), 0.84 (d, J = 6.8 Hz, 3H).

^{13}C NMR (126 MHz, $CDCl_3$) δ 147.98, 129.05, 115.28, 112.32, 63.24, 49.61, 29.43, 25.67, 24.42, 19.72, 16.57.

IR (neat, cm^{-1}): 2959 (m), 2873 (m), 2840 (m), 1597 (m), 1504 (m), 1366 (m), 1172 (m), 745 (w)

HRMS (ESI-MS): m/z calcd. for $C_{13}H_{19}N$ [$M+H^+$]: 190.1596; found 190.1574

2-ethyl-1-phenylpyrrolidine (**20**)

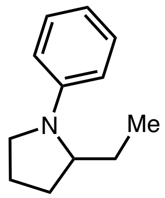

Synthesized according to Procedures M, O, and P.

^{1}H NMR (500 MHz, $CDCl_3$) δ 7.27 – 7.22 (m, 2H), 6.67 (t, *J* = 7.2 Hz, 1H), 6.59 (d, *J* = 8.0 Hz, 2H), 3.59 (m, 1H), 3.45 (t, *J* = 7.5 Hz, 1H), 3.23 – 3.12 (m, 1H), 2.02 (m, 3H), 1.90 – 1.73 (m, 2H), 1.32 (m, 1H), 0.96 (t, *J* = 7.5 Hz, 3H).

^{13}C NMR (126 MHz, $CDCl_3$) δ 147.35, 129.16, 115.18, 111.81, 60.12, 48.39, 29.74, 25.76, 23.49, 10.76.

IR (neat, cm^{-1}): 2963 (m), 2875 (m), 2834 (m), 1597 (m), 1505 (m), 1364 (m), 1161 (m), 746 (w)

HRMS (ESI-MS): m/z calcd. for $C_{12}H_{17}N$ [M+H^{+}]: 176.1439; found 176.1427

2-benzyl-1-phenylpyrrolidine (**21**)

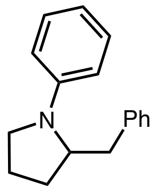

Synthesized according to literature procedure from **S21** (50 mg, 0.21 mmol).[8] Yield: 38 mg (76%).

^{1}H NMR (500 MHz, $CDCl_3$) δ 7.33 (m, 5H), 7.26 (d, *J* = 7.8 Hz, 2H), 6.74 (d, *J* = 8.0 Hz, 3H), 4.01 (tt, *J* = 7.0, 2.8 Hz, 1H), 3.46 (t, *J* = 7.6 Hz, 1H), 3.21 (t, *J* = 8.1 Hz, 1H), 3.10 (dd, *J* = 13.6, 3.0 Hz, 1H), 2.60 (dd, *J* = 13.5, 9.7 Hz, 1H), 2.04 – 1.80 (m, 4H).

^{13}C NMR (126 MHz, $CDCl_3$) δ 146.98, 139.55, 129.39, 128.44, 126.20, 115.49, 111.84, 59.78, 48.37, 38.59, 29.50, 23.03.

IR (neat, cm^{-1}): 3028 (m), 2968 (m), 2842 (m), 1597 (m), 1506 (m), 1366 (m), 1157 (m), 745 (w)

HRMS (ESI-MS): m/z calcd. for $C_{17}H_{19}N$ [M+H^{+}]: 238.1596; found 238.1562

1,3-diphenylpiperidine (**22**)

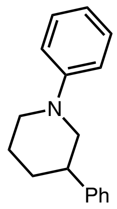

Synthesized according to Procedure O.

^{1}H NMR (500 MHz, $CDCl_3$) δ 7.41 – 7.29 (m, 6H), 7.27 (d, J = 4.3 Hz, 1H), 7.04 – 6.98 (m, 2H), 6.91 – 6.84 (m, 1H), 3.81 (m, 2H), 2.98 (tt, J = 11.7, 3.6 Hz, 1H), 2.90 – 2.76 (m, 2H), 2.15 – 2.03 (m, 1H), 1.97 – 1.80 (m, 2H), 1.70 (m, 1H).

^{13}C NMR (126 MHz, $CDCl_3$) δ 151.63, 144.36, 129.14, 128.54, 127.30, 126.57, 119.33, 116.56, 57.09, 50.16, 42.60, 31.59, 25.48.

IR (neat, cm^{-1}): 3028 (m), 2931 (m), 2804 (m), 1599 (m), 1496 (m), 1239 (w), 754 (w), 697 (w)

HRMS (ESI-MS): m/z calcd. for $C_{17}H_{19}N$ [$M+H^+$]: 238.1596; found 238.1568

2-(4-methoxybenzyl)-1-phenylpyrrolidine (**23**)

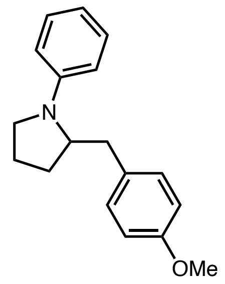

Synthesized according to literature procedure from **S23** (50 mg, 0.19 mmol).[9] Yield: 25 mg (50%).

^{1}H NMR (500 MHz, $CDCl_3$) δ 7.34 – 7.26 (m, 2H), 7.17 (d, *J* = 8.5 Hz, 2H), 6.91 – 6.85 (m, 2H), 6.71 (m, 3H), 3.95 (td, *J* = 6.3, 3.0 Hz, 1H), 3.83 (s, 3H), 3.43 (td, *J* = 7.0, 2.2 Hz, 1H), 3.20 (q, *J* = 7.8 Hz, 1H), 3.01 (dd, *J* = 13.8, 3.0 Hz, 1H), 2.55 (dd, *J* = 13.8, 9.4 Hz, 1H), 1.98 – 1.88 (m, 2H), 1.90 – 1.83 (m, 2H).

^{13}C NMR (126 MHz, $CDCl_3$) δ 158.10, 147.01, 131.56, 130.28, 129.33, 115.42, 113.84, 111.80, 77.28, 77.02, 76.77, 59.88, 55.27, 48.40, 37.64, 29.47, 23.04.

IR (neat, cm^{-1}): 2953 (m), 2836 (m), 1597 (m), 1505 (m), 1366 (m), 1246 (m), 1036 (m), 747 (w)

HRMS (ESI-MS): m/z calcd. for $C_{18}H_{21}NO$ [M+H$^+$]: 268.1701; found 268.1674

1-phenyl-2-(4-(trifluoromethyl)benzyl)pyrrolidine (**24**)

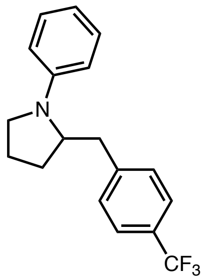

Synthesized according to literature procedure from **S24** (50 mg, 0.16 mmol).[9] Yield: 31 mg (62%).

^{1}H NMR (500 MHz, $CDCl_3$) δ 7.58 (d, *J* = 8.0 Hz, 2H), 7.36 (d, *J* = 8.0 Hz, 2H), 7.31 (t, *J* = 7.8 Hz, 2H), 6.73 (dd, *J* = 16.2, 7.9 Hz, 3H), 4.07 – 4.00 (m, 1H), 3.44 (t, *J* = 7.1 Hz, 1H), 3.22 (q, *J* = 8.4 Hz, 1H), 3.11 (dd, *J* = 13.6, 2.8 Hz, 1H), 2.71 (dd, *J* = 13.6, 9.1 Hz, 1H), 2.06 – 1.78 (m, 4H).

^{13}C NMR (126 MHz, $CDCl_3$) δ 146.81, 143.60, 129.70, 129.41, 128.73, 128.47, 125.31, 125.28, 115.81, 59.35, 48.44, 38.46, 29.57, 23.04.

IR (neat, cm^{-1}): 2970 (m), 2881 (m), 1597 (m), 1506 (m), 1323 (m), 1161 (m), 1145 (m), 747 (w)

HRMS (ESI-MS): m/z calcd. for $C_{18}H_{18}NF_3$ [M+H$^+$]: 306.1470; found 306.1451

1-phenyl-2-(tetrahydro-2*H*-pyran-3-yl)pyrrolidine (**25**, 1:1.5 mixture of diastereomers)

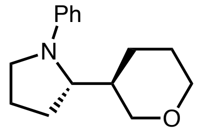

Synthesized according to Procedures M, N, and P. Yield: 12.3 mg (31%).

^{1}H NMR (500 MHz, $CDCl_3$) δ 7.24 (t, *J* = 7.6 Hz, 2H), 6.69 (t, *J* = 7.2 Hz, 1H), 6.64 (d, *J* = 6.9 Hz, 2H), 3.92 (m, 2H), 3.69 – 3.63 (m, 1H), 3.54 – 3.47 (m, 1H), 3.40 – 3.22 (m, 2H), 3.16 (m, 1H), 2.14 – 1.98 (m, 2H), 2.01 – 1.93 (m, 1H), 1.95 – 1.90 (m, 1H), 1.83 (m, 2H), 1.62 (m, 1H), 1.33 (m, 2H).

^{13}C NMR (126 MHz, CDCl3) δ 129.11, 129.08, 115.69, 112.35, 112.25, 77.28, 77.02, 76.77, 71.12, 70.27, 68.56, 68.47, 60.33, 59.16, 49.20, 49.11, 39.62, 39.59, 27.49, 27.44, 27.38, 26.49, 26.04, 25.38, 24.26, 24.24.

IR (neat, cm^{-1}): 2953 (m), 2931 (m), 1507 (s), 1325 (s), 1099 (m),

HRMS (ESI-MS): m/z calcd. for $C_{15}H_{22}NO$ [M+H$^+$]: 232.1701; found 232.1782

1-phenyl-2-(1-phenylethyl)pyrrolidine (**26**, 1:3.5 cis:trans mixture of diastereomers)

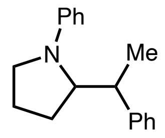

Synthesized according to literature procedure from **S26** (68 mg, 0.26 mmol).[9] Yield: 65 mg (95%).

NMR spectra were in accordance with the literature.[9]

IR (neat, cm^{-1}): 2965 (m), 2875 (m), 1597 (s), 1504 (s), 1362 (m)

HRMS (ESI-MS): m/z calcd. for $C_{18}H_{22}N$ $[M+H^+]$: 252.1752; found 252.1756

G. Protein and DNA sequences

All plasmids were constructed in pET-28a(+) vectors with an N-terminal His-tag and a thrombin cleavage site between the NdeI and XhoI restriction sites. Whole plasmids were purchased from Twist Biosciences and transformed into electrocompetent *E. coli* BL21(DE3) cells.

GlowER Protein sequence
MPTLFDPIDFGPIHAKNRIVMSPLTRGRADKEAVPTPIMAEYYAQRASAGLIITEATGISR
EGLGFPFAPGIWSDAQVEAWKPIVAGVHAKGGKIVCQLFHMGRMVHSSVTGTQPVSSS
ATTAPGEVHTYEGKKPFEQARAIDAADISRILNDYENAARNAIRAGFDGVQIHAANGFLI
DEFLRNGTNHRTDEYGGVPENRIRFLKEVTERVIAAIGADRTGVRLSPNGDTQGCIDSAP
ETVFVPAAKLLQDLGVAWLELREPGPNGTFGKTDQPKLSPQIRKVFLRPLVLNQDYTFE
AAQTALAEGKADAIAFGRKFISNPDLPERFARGIALQPDDMKTFFSQGPEGYTDYPSATS
GPN*
GlowER DNA sequence
ATGCCGACCCTGTTTGATCCTATTGATTTTGGCCCCATCCACGCCAAAAATCGTATCG
TTATGAGCCCGCTGACTCGTGGCCGTGCCGACAAAGAGGCCGTCCCGACCCCGATC
ATGGCGGAATATTATGCCCAACGGGCAAGCGCCGGACTGATCATTACGGAAGCAAC
GGGAATCTCACGCGAAGGCCTGGGTTTTCCGTTCGCGCCAGGCATTTGGAGCGACGC
GCAAGTTGAGGCCTGGAAACCCATTGTTGCAGGCGTCCACGCCAAAGGTGGTAAAA
TTGTTTGCCAATTGTTCCACATGGGCCGTATGGTGCATTCGAGCGTGACGGGTACGC
AACCAGTTAGCAGCAGCGCCACAACCGCTCCGGGGGAAGTGCATACCTACGAAGGG
AAAAAGCCGTTCGAACAAGCCCGCGCTATTGATGCCGCCGATATTAGTCGGATTTTA
AACGATTATGAAAACGCGGCCCGCAATGCCATCCGTGCGGGTTTCGATGGTGTTCAG
ATTCATGCGGCAAACGGATTTTTAATTGATGAATTCCTGCGTAATGGGACTAATCAT
CGCACGGACGAATATGGGGGCGTGCCGGAAAACCGCATTCGTTTTTTGAAGGAAGT
GACGGAGCGCGTCATTGCGGCGATTGGGGCGGATCGCACTGGCGTGCGTCTGAGCC
CTAACGGGGACACCCAGGGCTGCATCGACAGTGCCCCGGAGACAGTGTTCGTGCCG
GCAGCGAAATTGCTGCAAGATCTGGGGGTAGCGTGGCTGGAACTTCGTGAACCGGG
CCCGAACGGCACCTTCGGGAAAACCGATCAGCCGAAATTATCTCCGCAGATCCGTA
AAGTGTTCCTGCGTCCACTGGTGCTCAATCAGGATTACACATTTGAAGCGGCGCAGA
CCGCCCTCGCGGAAGGTAAAGCGGACGCGATCGCATTTGGCCGGAAATTCATCTCT
AATCCCGACCTCCCTGAGCGTTTTGCGCGGGGCATTGCGCTGCAACCCGATGACATG
AAAACGTTTTTTTCTCAAGGCCCGGAAGGTTACACCGATTATCCGTCCGCCACGTCA
GGCCCGAACTAA

GlowER H172G Protein Sequence
MPTLFDPIDFGPIHAKNRIVMSPLTRGRADKEAVPTPIMAEYYAQRASAGLIITEATGISR
EGLGFPFAPGIWSDAQVEAWKPIVAGVHAKGGKIVCQLFHMGRMVHSSVTGTQPVSSS
ATTAPGEVHTYEGKKPFEQARAIDAADISRILNDYENAARNAIRAGFDGVQIGAANGFLI
DEFLRNGTNHRTDEYGGVPENRIRFLKEVTERVIAAIGADRTGVRLSPNGDTQGCIDSAP
ETVFVPAAKLLQDLGVAWLELREPGPNGTFGKTDQPKLSPQIRKVFLRPLVLNQDYTFE
AAQTALAEGKADAIAFGRKFISNPDLPERFARGIALQPDDMKTFFSQGPEGYTDYPSATS
GPN*
GlowER H172G DNA Sequence
ATGCCGACCCTGTTTGATCCTATTGATTTTGGCCCCATCCACGCCAAAAATCGTATCG
TTATGAGCCCGCTGACTCGTGGCCGTGCCGACAAAGAGGCCGTCCCGACCCCGATC

ATGGCGGAATATTATGCCCAACGGGCAAGCGCCGGACTGATCATTACGGAAGCAAC
GGGAATCTCACGCGAAGGCCTGGGTTTTCCGTTCGCGCCAGGCATTTGGAGCGACGC
GCAAGTTGAGGCCTGGAAACCCATTGTTGCAGGCGTCCACGCCAAAGGTGGTAAAA
TTGTTTGCCAATTGTTCCACATGGGCCGTATGGTGCATTCGAGCGTGACGGGTACGC
AACCAGTTAGCAGCAGCGCCACAACCGCTCCGGGGGAAGTGCATACCTACGAAGGG
AAAAAGCCGTTCGAACAAGCCCGCGCTATTGATGCCGCCGATATTAGTCGGATTTTA
AACGATTATGAAAACGCGGCCCGCAATGCCATCCGTGCGGGTTTCGATGGTGTTCAG
ATTGGTGCGGCAAACGGATTTTTAATTGATGAATTCCTGCGTAATGGGACTAATCAT
CGCACGGACGAATATGGGGGCGTGCCGGAAAACCGCATTCGTTTTTTGAAGGAAGT
GACGGAGCGCGTCATTGCGGCGATTGGGGCGGATCGCACTGGCGTGCGTCTGAGCC
CTAACGGGGACACCCAGGGCTGCATCGACAGTGCCCCGGAGACAGTGTTCGTGCCG
GCAGCGAAATTGCTGCAAGATCTGGGGGTAGCGTGGCTGGAACTTCGTGAACCGGG
CCCGAACGGCACCTTCGGGAAAACCGATCAGCCGAAATTATCTCCGCAGATCCGTA
AAGTGTTCCTGCGTCCACTGGTGCTCAATCAGGATTACACATTTGAAGCGGCGCAGA
CCGCCCTCGCGGAAGGTAAAGCGGACGCGATCGCATTTGGCCGGAAATTCATCTCT
AATCCCGACCTCCCTGAGCGTTTTGCGCGGGGCATTGCGCTGCAACCCGATGACATG
AAAACGTTTTTTCTCAAGGCCCGGAAGGTTACACCGATTATCCGTCCGCCACGTCA
GGCCCGAACTAA

GlowER H172G F100V Protein Sequence
MPTLFDPIDFGPIHAKNRIVMSPLTRGRADKEAVPTPIMAEYYAQRASAGLIITEATGISR
EGLGFPFAPGIWSDAQVEAWKPIVAGVHAKGGKIVCQLVHMGRMVHSSVTGTQPVSSS
ATTAPGEVHTYEGKKPFEQARAIDAADISRILNDYENAARNAIRAGFDGVQIGAANGFLI
DEFLRNGTNHRTDEYGGVPENRIRFLKEVTERVIAAIGADRTGVRLSPNGDTQGCIDSAP
ETVFVPAAKLLQDLGVAWLELREPGPNGTFGKTDQPKLSPQIRKVFLRPLVLNQDYTFE
AAQTALAEGKADAIAFGRKFISNPDLPERFARGIALQPDDMKTFFSQGPEGYTDYPSATS
GPN*
GlowER H172G F100V DNA Sequence
ATGCCGACCCTGTTTGATCCTATTGATTTTGGCCCCATCCACGCCAAAAATCGTATCG
TTATGAGCCCGCTGACTCGTGGCCGTGCCGACAAAGAGGCCGTCCCGACCCCGATC
ATGGCGGAATATTATGCCCAACGGGCAAGCGCCGGACTGATCATTACGGAAGCAAC
GGGAATCTCACGCGAAGGCCTGGGTTTTCCGTTCGCGCCAGGCATTTGGAGCGACGC
GCAAGTTGAGGCCTGGAAACCCATTGTTGCAGGCGTCCACGCCAAAGGTGGTAAAA
TTGTTTGCCAATTGGTTCACATGGGCCGTATGGTGCATTCGAGCGTGACGGGTACGC
AACCAGTTAGCAGCAGCGCCACAACCGCTCCGGGGGAAGTGCATACCTACGAAGGG
AAAAAGCCGTTCGAACAAGCCCGCGCTATTGATGCCGCCGATATTAGTCGGATTTTA
AACGATTATGAAAACGCGGCCCGCAATGCCATCCGTGCGGGTTTCGATGGTGTTCAG
ATTGGTGCGGCAAACGGATTTTTAATTGATGAATTCCTGCGTAATGGGACTAATCAT
CGCACGGACGAATATGGGGGCGTGCCGGAAAACCGCATTCGTTTTTTGAAGGAAGT
GACGGAGCGCGTCATTGCGGCGATTGGGGCGGATCGCACTGGCGTGCGTCTGAGCC
CTAACGGGGACACCCAGGGCTGCATCGACAGTGCCCCGGAGACAGTGTTCGTGCCG
GCAGCGAAATTGCTGCAAGATCTGGGGGTAGCGTGGCTGGAACTTCGTGAACCGGG
CCCGAACGGCACCTTCGGGAAAACCGATCAGCCGAAATTATCTCCGCAGATCCGTA
AAGTGTTCCTGCGTCCACTGGTGCTCAATCAGGATTACACATTTGAAGCGGCGCAGA
CCGCCCTCGCGGAAGGTAAAGCGGACGCGATCGCATTTGGCCGGAAATTCATCTCT
AATCCCGACCTCCCTGAGCGTTTTGCGCGGGGCATTGCGCTGCAACCCGATGACATG

AAAACGTTTTTTCTCAAGGCCCGGAAGGTTACACCGATTATCCGTCCGCCACGTCA
GGCCCGAACTAA

GlowER H172G F100V M102L Protein Sequence
MPTLFDPIDFGPIHAKNRIVMSPLTRGRADKEAVPTPIMAEYYAQRASAGLIITEATGISR
EGLGFPFAPGIWSDAQVEAWKPIVAGVHAKGGKIVCQLVHLGRMVHSSVTGTQPVSSS
ATTAPGEVHTYEGKKPFEQARAIDAADISRILNDYENAARNAIRAGFDGVQIGAANGFLI
DEFLRNGTNHRTDEYGGVPENRIRFLKEVTERVIAAIGADRTGVRLSPNGDTQGCIDSAP
ETVFVPAAKLLQDLGVAWLELREPGPNGTFGKTDQPKLSPQIRKVFLRPLVLNQDYTFE
AAQTALAEGKADAIAFGRKFISNPDLPERFARGIALQPDDMKTFFSQGPEGYTDYPSATS
GPN*
GlowER H172G F100V M102L DNA Sequence
ATGCCGACCCTGTTTGATCCTATTGATTTTGGCCCCATCCACGCCAAAAATCGTATCG
TTATGAGCCCGCTGACTCGTGGCCGTGCCGACAAAGAGGCCGTCCCGACCCCGATC
ATGGCGGAATATTATGCCCAACGGGCAAGCGCCGGACTGATCATTACGGAAGCAAC
GGGAATCTCACGCGAAGGCCTGGGTTTTCCGTTCGCGCCAGGCATTTGGAGCGACGC
GCAAGTTGAGGCCTGGAAACCCATTGTTGCAGGCGTCCACGCCAAAGGTGGTAAAA
TTGTTTGCCAATTGGTTCACCTGGGCCGTATGGTGCATTCGAGCGTGACGGGTACGC
AACCAGTTAGCAGCAGCGCCACAACCGCTCCGGGGGAAGTGCATACCTACGAAGGG
AAAAAGCCGTTCGAACAAGCCCGCGCTATTGATGCCGCCGATATTAGTCGGATTTTA
AACGATTATGAAAACGCGGCCCGCAATGCCATCCGTGCGGGTTTCGATGGTGTTCAG
ATTGGTGCGGCAAACGGATTTTTAATTGATGAATTCCTGCGTAATGGGACTAATCAT
CGCACGGACGAATATGGGGGCGTGCCGGAAAACCGCATTCGTTTTTTGAAGGAAGT
GACGGAGCGCGTCATTGCGGCGATTGGGGCGGATCGCACTGGCGTGCGTCTGAGCC
CTAACGGGGACACCCAGGGCTGCATCGACAGTGCCCCGGAGACAGTGTTCGTGCCG
GCAGCGAAATTGCTGCAAGATCTGGGGGTAGCGTGGCTGGAACTTCGTGAACCGGG
CCCGAACGGCACCTTCGGGAAAACCGATCAGCCGAAATTATCTCCGCAGATCCGTA
AAGTGTTCCTGCGTCCACTGGTGCTCAATCAGGATTACACATTTGAAGCGGCGCAGA
CCGCCCTCGCGGAAGGTAAAGCGGACGCGATCGCATTTGGCCGGAAATTCATCTCT
AATCCCGACCTCCCTGAGCGTTTTGCGCGGGGCATTGCGCTGCAACCCGATGACATG
AAAACGTTTTTTCTCAAGGCCCGGAAGGTTACACCGATTATCCGTCCGCCACGTCA
GGCCCGAACTAA

GlowER H172G F100V M102L F66L Protein Sequence
MPTLFDPIDFGPIHAKNRIVMSPLTRGRADKEAVPTPIMAEYYAQRASAGLIITEATGISR
EGLGLPFAPGIWSDAQVEAWKPIVAGVHAKGGKIVCQLVHLGRMVHSSVTGTQPVSSS
ATTAPGEVHTYEGKKPFEQARAIDAADISRILNDYENAARNAIRAGFDGVQIGAANGFLI
DEFLRNGTNHRTDEYGGVPENRIRFLKEVTERVIAAIGADRTGVRLSPNGDTQGCIDSAP
ETVFVPAAKLLQDLGVAWLELREPGPNGTFGKTDQPKLSPQIRKVFLRPLVLNQDYTFE
AAQTALAEGKADAIAFGRKFISNPDLPERFARGIALQPDDMKTFFSQGPEGYTDYPSATS
GPN*
GlowER H172G F100V M102L F66L DNA Sequence
ATGCCGACCCTGTTTGATCCTATTGATTTTGGCCCCATCCACGCCAAAAATCGTATCG
TTATGAGCCCGCTGACTCGTGGCCGTGCCGACAAAGAGGCCGTCCCGACCCCGATC
ATGGCGGAATATTATGCCCAACGGGCAAGCGCCGGACTGATCATTACGGAAGCAAC
GGGAATCTCACGCGAAGGCCTGGGTCTGCCGTTCGCGCCAGGCATTTGGAGCGACG

CGCAAGTTGAGGCCTGGAAACCCATTGTTGCAGGCGTCCACGCCAAAGGTGGTAAA
ATTGTTTGCCAATTGGTTCACCTGGGCCGTATGGTGCATTCGAGCGTGACGGGTACG
CAACCAGTTAGCAGCAGCGCCACAACCGCTCCGGGGGAAGTGCATACCTACGAAGG
GAAAAAGCCGTTCGAACAAGCCCGCGCTATTGATGCCGCCGATATTAGTCGGATTTT
AAACGATTATGAAAACGCGGCCCGCAATGCCATCCGTGCGGGTTTCGATGGTGTTCA
GATTGGTGCGGCAAACGGATTTTTAATTGATGAATTCCTGCGTAATGGGACTAATCA
TCGCACGGACGAATATGGGGGCGTGCCGGAAAACCGCATTCGTTTTTTGAAGGAAG
TGACGGAGCGCGTCATTGCGGCGATTGGGGCGGATCGCACTGGCGTGCGTCTGAGC
CCTAACGGGGACACCCAGGGCTGCATCGACAGTGCCCCGGAGACAGTGTTCGTGCC
GGCAGCGAAATTGCTGCAAGATCTGGGGGTAGCGTGGCTGGAACTTCGTGAACCGG
GCCCGAACGGCACCTTCGGGAAAACCGATCAGCCGAAATTATCTCCGCAGATCCGT
AAAGTGTTCCTGCGTCCACTGGTGCTCAATCAGGATTACACATTTGAAGCGGCGCAG
ACCGCCCTCGCGGAAGGTAAAGCGGACGCGATCGCATTTGGCCGGAAATTCATCTC
TAATCCCGACCTCCCTGAGCGTTTTGCGCGGGGCATTGCGCTGCAACCCGATGACAT
GAAAACGTTTTTTCTCAAGGCCCGGAAGGTTACACCGATTATCCGTCCGCCACGTC
AGGCCCGAACTAA

GlowER H172G F100V M102L F66L Q232V Protein Sequence
MPTLFDPIDFGPIHAKNRIVMSPLTRGRADKEAVPTPIMAEYYAQRASAGLIITEATGISR
EGLGLPFAPGIWSDAQVEAWKPIVAGVHAKGGKIVCQLVHLGRMVHSSVTGTQPVSSS
ATTAPGEVHTYEGKKPFEQARAIDAADISRILNDYENAARNAIRAGFDGVQIGAANGFLI
DEFLRNGTNHRTDEYGGVPENRIRFLKEVTERVIAAIGADRTGVRLSPNGDTVGCIDSAP
ETVFVPAAKLLQDLGVAWLELREPGPNGTFGKTDQPKLSPQIRKVFLRPLVLNQDYTFE
AAQTALAEGKADAIAFGRKFISNPDLPERFARGIALQPDDMKTFFSQGPEGYTDYPSATS
GPN*

GlowER H172G F100V M102L F66L Q232V DNA Sequence
ATGCCGACCCTGTTTGATCCTATTGATTTTGGCCCCATCCACGCCAAAAATCGTATCG
TTATGAGCCCGCTGACTCGTGGCCGTGCCGACAAAGAGGCCGTCCCGACCCCGATC
ATGGCGGAATATTATGCCCAACGGGCAAGCGCCGGACTGATCATTACGGAAGCAAC
GGGAATCTCACGCGAAGGCCTGGGTCTGCCGTTCGCGCCAGGCATTTGGAGCGACG
CGCAAGTTGAGGCCTGGAAACCCATTGTTGCAGGCGTCCACGCCAAAGGTGGTAAA
ATTGTTTGCCAATTGGTTCACCTGGGCCGTATGGTGCATTCGAGCGTGACGGGTACG
CAACCAGTTAGCAGCAGCGCCACAACCGCTCCGGGGGAAGTGCATACCTACGAAGG
GAAAAAGCCGTTCGAACAAGCCCGCGCTATTGATGCCGCCGATATTAGTCGGATTTT
AAACGATTATGAAAACGCGGCCCGCAATGCCATCCGTGCGGGTTTCGATGGTGTTCA
GATTGGTGCGGCAAACGGATTTTTAATTGATGAATTCCTGCGTAATGGGACTAATCA
TCGCACGGACGAATATGGGGGCGTGCCGGAAAACCGCATTCGTTTTTTGAAGGAAG
TGACGGAGCGCGTCATTGCGGCGATTGGGGCGGATCGCACTGGCGTGCGTCTGAGC
CCTAACGGGGACACCGTGGGCTGCATCGACAGTGCCCCGGAGACAGTGTTCGTGCC
GGCAGCGAAATTGCTGCAAGATCTGGGGGTAGCGTGGCTGGAACTTCGTGAACCGG
GCCCGAACGGCACCTTCGGGAAAACCGATCAGCCGAAATTATCTCCGCAGATCCGT
AAAGTGTTCCTGCGTCCACTGGTGCTCAATCAGGATTACACATTTGAAGCGGCGCAG
ACCGCCCTCGCGGAAGGTAAAGCGGACGCGATCGCATTTGGCCGGAAATTCATCTC
TAATCCCGACCTCCCTGAGCGTTTTGCGCGGGGCATTGCGCTGCAACCCGATGACAT
GAAAACGTTTTTCTCTCAAGGCCCGGAAGGTTACACCGATTATCCGTCCGCCACGTC
AGGCCCGAACTAA

GlowER H172G F100V M102L F66L Q232V M105V Protein Sequence
MPTLFDPIDFGPIHAKNRIVMSPLTRGRADKEAVPTPIMAEYYAQRASAGLIITEATGISR
EGLGLPFAPGIWSDAQVEAWKPIVAGVHAKGGKIVCQLVHLGRVVHSSVTGTQPVSSS
ATTAPGEVHTYEGKKPFEQARAIDAADISRILNDYENAARNAIRAGFDGVQIGAANGFLI
DGFLRNGTNHRTDEYGGVPENRIRFLKEVTERVIAAIGADRTGVRLSPNGDTVGCIDSAP
ETVFVPAAKLLQDLGVAWLELREPGPNGTFGKTDQPKLSPQIRKVFLRPLVLNQDYTFE
AAQTALAEGKADAIAFGRKFISNPDLPERFARGIALQPDDMKTFFSQGPEGYTDYPSATS
GPN*
GlowER H172G F100V M102L F66L Q232V M105V DNA Sequence
ATGCCGACCCTGTTTGATCCTATTGATTTTGGCCCCATCCACGCCAAAAATCGTATCG
TTATGAGCCCGCTGACTCGTGGCCGTGCCGACAAAGAGGCCGTCCCGACCCCGATC
ATGGCGGAATATTATGCCCAACGGGCAAGCGCCGGACTGATCATTACGGAAGCAAC
GGGAATCTCACGCGAAGGCCTGGGTCTGCCGTTCGCGCCAGGCATTTGGAGCGACG
CGCAAGTTGAGGCCTGGAAACCCATTGTTGCAGGCGTCCACGCCAAAGGTGGTAAA
ATTGTTTGCCAATTGGTTCACCTGGGCCGTGTTGTGCATTCGAGCGTGACGGGTACG
CAACCAGTTAGCAGCAGCGCCACAACCGCTCCGGGGGAAGTGCATACCTACGAAGG
GAAAAAGCCGTTCGAACAAGCCCGCGCTATTGATGCCGCCGATATTAGTCGGATTTT
AAACGATTATGAAAACGCGGCCCGCAATGCCATCCGTGCGGGTTTCGATGGTGTTCA
GATTGGTGCGGCAAACGGATTTTTAATTGATGGTTTCCTGCGTAATGGGACTAATCA
TCGCACGGACGAATATGGGGGCGTGCCGGAAAACCGCATTCGTTTTTTGAAGGAAG
TGACGGAGCGCGTCATTGCGGCGATTGGGGCGGATCGCACTGGCGTGCGTCTGAGC
CCTAACGGGGACACCGTGGGCTGCATCGACAGTGCCCCGGAGACAGTGTTCGTGCC
GGCAGCGAAATTGCTGCAAGATCTGGGGGTAGCGTGGCTGGAACTTCGTGAACCGG
GCCCGAACGGCACCTTCGGGAAAACCGATCAGCCGAAATTATCTCCGCAGATCCGT
AAAGTGTTCCTGCGTCCACTGGTGCTCAATCAGGATTACACATTTGAAGCGGCGCAG
ACCGCCCTCGCGGAAGGTAAAGCGGACGCGATCGCATTTGGCCGGAAATTCATCTC
TAATCCCGACCTCCCTGAGCGTTTTGCGCGGGGCATTGCGCTGCAACCCGATGACAT
GAAAACGTTTTTCTCTCAAGGCCCGGAAGGTTACACCGATTATCCGTCCGCCACGTC
AGGCCCGAACTAA

GlowER H172G F100V M102L F66L Q232V M105V E181G Protein Sequence
MPTLFDPIDFGPIHAKNRIVMSPLTRGRADKEAVPTPIMAEYYAQRASAGLIITEATGISR
EGLGLPFAPGIWSDAQVEAWKPIVAGVHAKGGKIVCQLVHLGRVVHSSVTGTQPVSSS
ATTAPGEVHTYEGKKPFEQARAIDAADISRILNDYENAARNAIRAGFDGVQIGAANGFLI
DGFLRNGTNHRTDEYGGVPENRIRFLKEVTERVIAAIGADRTGVRLSPNGDTVGCIDSAP
ETVFVPAAKLLQDLGVAWLELREPGPNGTFGKTDQPKLSPQIRKVFLRPLVLNQDYTFE
AAQTALAEGKADAIAFGRKFISNPDLPERFARGIALQPDDMKTFFSQGPEGYTDYPSATS
GPN*
GlowER H172G F100V M102L F66L Q232V M105V E181G DNA Sequence
ATGCCGACCCTGTTTGATCCTATTGATTTTGGCCCCATCCACGCCAAAAATCGTATCG
TTATGAGCCCGCTGACTCGTGGCCGTGCCGACAAAGAGGCCGTCCCGACCCCGATC
ATGGCGGAATATTATGCCCAACGGGCAAGCGCCGGACTGATCATTACGGAAGCAAC
GGGAATCTCACGCGAAGGCCTGGGTCTGCCGTTCGCGCCAGGCATTTGGAGCGACG
CGCAAGTTGAGGCCTGGAAACCCATTGTTGCAGGCGTCCACGCCAAAGGTGGTAAA
ATTGTTTGCCAATTGGTTCACCTGGGCCGTGTTGTGCATTCGAGCGTGACGGGTACG
CAACCAGTTAGCAGCAGCGCCACAACCGCTCCGGGGGAAGTGCATACCTACGAAGG

GAAAAAGCCGTTCGAACAAGCCCGCGCTATTGATGCCGCCGATATTAGTCGGATTTT
AAACGATTATGAAAACGCGGCCCGCAATGCCATCCGTGCGGGTTTCGATGGTGTTCA
GATTGGTGCGGCAAACGGATTTTTAATTGATGGTTTCCTGCGTAATGGGACTAATCA
TCGCACGGACGAATATGGGGGCGTGCCGGAAAACCGCATTCGTTTTTTGAAGGAAG
TGACGGAGCGCGTCATTGCGGCGATTGGGGCGGATCGCACTGGCGTGCGTCTGAGC
CCTAACGGGGACACCGTGGGCTGCATCGACAGTGCCCCGGAGACAGTGTTCGTGCC
GGCAGCGAAATTGCTGCAAGATCTGGGGGTAGCGTGGCTGGAACTTCGTGAACCGG
GCCCGAACGGCACCTTCGGGAAAACCGATCAGCCGAAATTATCTCCGCAGATCCGT
AAAGTGTTCCTGCGTCCACTGGTGCTCAATCAGGATTACACATTTGAAGCGGCGCAG
ACCGCCCTCGCGGAAGGTAAAGCGGACGCGATCGCATTTGGCCGGAAATTCATCTC
TAATCCCGACCTCCCTGAGCGTTTTGCGCGGGGCATTGCGCTGCAACCCGATGACAT
GAAAACGTTTTTTTCTCAAGGCCCGGAAGGTTACACCGATTATCCGTCCGCCACGTC
AGGCCCGAACTAA

GlowER H172G F100V M102L F66L Q232V M105V E181G F343G Protein Sequence
MPTLFDPIDFGPIHAKNRIVMSPLTRGRADKEAVPTPIMAEYYAQRASAGLIITEATGISR
EGLGLPFAPGIWSDAQVEAWKPIVAGVHAKGGKIVCQLVHLGRVVHSSVTGTQPVSSS
ATTAPGEVHTYEGKKPFEQARAIDAADISRILNDYENAARNAIRAGFDGVQIGAANGFLI
DGFLRNGTNHRTDEYGGVPENRIRFLKEVTERVIAAIGADRTGVRLSPNGDTVGCIDSAP
ETVFVPAAKLLQDLGVAWLELREPGPNGTFGKTDQPKLSPQIRKVFLRPLVLNQDYTFE
AAQTALAEGKADAIAFGRKFISNPDLPERFARGIALQPDDMKTFGSQGPEGYTDYPSATS
GPN*
GlowER H172G F100V M102L F66L Q232V M105V E181G F343G DNA Sequence
ATGCCGACCCTGTTTGATCCTATTGATTTTGGCCCCATCCACGCCAAAAATCGTATCG
TTATGAGCCCGCTGACTCGTGGCCGTGCCGACAAAGAGGCCGTCCCGACCCCGATC
ATGGCGGAATATTATGCCCAACGGGCAAGCGCCGGACTGATCATTACGGAAGCAAC
GGGAATCTCACGCGAAGGCCTGGGTCTGCCGTTCGCGCCAGGCATTTGGAGCGACG
CGCAAGTTGAGGCCTGGAAACCCATTGTTGCAGGCGTCCACGCCAAAGGTGGTAAA
ATTGTTTGCCAATTGGTTCACCTGGGCCGTGTTGTGCATTCGAGCGTGACGGGTACG
CAACCAGTTAGCAGCAGCGCCACAACCGCTCCGGGGGAAGTGCATACCTACGAAGG
GAAAAAGCCGTTCGAACAAGCCCGCGCTATTGATGCCGCCGATATTAGTCGGATTTT
AAACGATTATGAAAACGCGGCCCGCAATGCCATCCGTGCGGGTTTCGATGGTGTTCA
GATTGGTGCGGCAAACGGATTTTTAATTGATGGTTTCCTGCGTAATGGGACTAATCA
TCGCACGGACGAATATGGGGGCGTGCCGGAAAACCGCATTCGTTTTTTGAAGGAAG
TGACGGAGCGCGTCATTGCGGCGATTGGGGCGGATCGCACTGGCGTGCGTCTGAGC
CCTAACGGGGACACCGTGGGCTGCATCGACAGTGCCCCGGAGACAGTGTTCGTGCC
GGCAGCGAAATTGCTGCAAGATCTGGGGGTAGCGTGGCTGGAACTTCGTGAACCGG
GCCCGAACGGCACCTTCGGGAAAACCGATCAGCCGAAATTATCTCCGCAGATCCGT
AAAGTGTTCCTGCGTCCACTGGTGCTCAATCAGGATTACACATTTGAAGCGGCGCAG
ACCGCCCTCGCGGAAGGTAAAGCGGACGCGATCGCATTTGGCCGGAAATTCATCTC
TAATCCCGACCTCCCTGAGCGTTTTGCGCGGGGCATTGCGCTGCAACCCGATGACAT
GAAAACGTTTGGTTCTCAAGGCCCGGAAGGTTACACCGATTATCCGTCCGCCACGTC
AGGCCCGAACTAA

GlowER H172G F100V M102L F66L Q232V M105V E181G F343G F342V Protein Sequence

MPTLFDPIDFGPIHAKNRIVMSPLTRGRADKEAVPTPIMAEYYAQRASAGLIITEATGISR
EGLGLPFMPGIWSDAQVEAWKPIVAGVHAKGGKIVCQLVHLGRVVHSSVTGTQPVSSS
ATTAPGEVHTYEGKKPFEQARAIDAADISRILNDYENAARNAIRAGFDGVQIGAANGFLI
DGFLRNGTNHRTDEYGGVPENRIRFLKEVTERVIAAIGADRTGVRLSPNGDTVGCIDSAP
ETVFVPAAKLLQDLGVAWLELREPGPNGTFGKTDQPKLSPQIRKVFLRPLVLNQDYTFE
AAQTALAEGKADAIAFGRKFISNPDLPERFARGIALQPDDMKTVGSQGPEGYTDYPSAT
SGPN*

GlowER H172G F100V M102L F66L Q232V M105V E181G F343G F342V DNA Sequence

ATGCCGACCCTGTTTGATCCTATTGATTTTGGCCCCATCCACGCCAAAAATCGTATCG
TTATGAGCCCGCTGACTCGTGGCCGTGCCGACAAAGAGGCCGTCCCGACCCCGATC
ATGGCGGAATATTATGCCCAACGGGCAAGCGCCGGACTGATCATTACGGAAGCAAC
GGGAATCTCACGCGAAGGCCTGGGTCTGCCGTTCATGCCAGGCATTTGGAGCGACG
CGCAAGTTGAGGCCTGGAAACCCATTGTTGCAGGCGTCCACGCCAAAGGTGGTAAA
ATTGTTTGCCAATTGGTTCACCTGGGCCGTGTTGTGCATTCGAGCGTGACGGGTACG
CAACCAGTTAGCAGCAGCGCCACAACCGCTCCGGGGGAAGTGCATACCTACGAAGG
GAAAAAGCCGTTCGAACAAGCCCGCGCTATTGATGCCGCCGATATTAGTCGGATTTT
AAACGATTATGAAAACGCGGCCCGCAATGCCATCCGTGCGGGTTTCGATGGTGTTCA
GATTGGTGCGGCAAACGGATTTTTAATTGATGGTTTCCTGCGTAATGGGACTAATCA
TCGCACGGACGAATATGGGGGCGTGCCGGAAAACCGCATTCGTTTTTTGAAGGAAG
TGACGGAGCGCGTCATTGCGGCGATTGGGGCGGATCGCACTGGCGTGCGTCTGAGC
CCTAACGGGGACACCGTGGGCTGCATCGACAGTGCCCCGGAGACAGTGTTCGTGCC
GGCAGCGAAATTGCTGCAAGATCTGGGGGTAGCGTGGCTGGAACTTCGTGAACCGG
GCCCGAACGGCACCTTCGGGAAAACCGATCAGCCGAAATTATCTCCGCAGATCCGT
AAAGTGTTCCTGCGTCCACTGGTGCTCAATCAGGATTACACATTTGAAGCGGCGCAG
ACCGCCCTCGCGGAAGGTAAAGCGGACGCGATCGCATTTGGCCGGAAATTCATCTC
TAATCCCGACCTCCCTGAGCGTTTTGCGCGGGGCATTGCGCTGCAACCCGATGACAT
GAAAACGGTTGGTTCTCAAGGCCCGGAAGGTTACACCGATTATCCGTCCGCCACGTC
AGGCCCGAACTAA

GlowER H172G F100V M102L F66L Q232V M105V E181G F343G F342V A69M Protein Sequence

MPTLFDPIDFGPIHAKNRIVMSPLTRGRADKEAVPTPIMAEYYAQRASAGLIITEATGISR
EGLGLPFMPGIWSDAQVEAWKPIVAGVHAKGGKIVCQLVHLGRVVHSSVTGTQPVSSS
ATTAPGEVHTYEGKKPFEQARAIDAADISRILNDYENAARNAIRAGFDGVQIGAANGFLI
DGFLRNGTNHRTDEYGGVPENRIRFLKEVTERVIAAIGADRTGVRLSPNGDTVGCIDSAP
ETVFVPAAKLLQDLGVAWLELREPGPNGTFGKTDQPKLSPQIRKVFLRPLVLNQDYTFE
AAQTALAEGKADAIAFGRKFISNPDLPERFARGIALQPDDMKTVGSQGPEGYTDYPSAT
SGPN*

GlowER H172G F100V M102L F66L Q232V M105V E181G F343G F342V A69M DNA Sequence

ATGCCGACCCTGTTTGATCCTATTGATTTTGGCCCCATCCACGCCAAAAATCGTATCG
TTATGAGCCCGCTGACTCGTGGCCGTGCCGACAAAGAGGCCGTCCCGACCCCGATC
ATGGCGGAATATTATGCCCAACGGGCAAGCGCCGGACTGATCATTACGGAAGCAAC

GGGAATCTCACGCGAAGGCCTGGGTCTGCCGTTCATGCCAGGCATTTGGAGCGACG
CGCAAGTTGAGGCCTGGAAACCCATTGTTGCAGGCGTCCACGCCAAAGGTGGTAAA
ATTGTTTGCCAATTGGTTCACCTGGGCCGTGTTGTGCATTCGAGCGTGACGGGTACG
CAACCAGTTAGCAGCAGCGCCACAACCGCTCCGGGGGAAGTGCATACCTACGAAGG
GAAAAAGCCGTTCGAACAAGCCCGCGCTATTGATGCCGCCGATATTAGTCGGATTTT
AAACGATTATGAAAACGCGGCCCGCAATGCCATCCGTGCGGGTTTCGATGGTGTTCA
GATTGGTGCGGCAAACGGATTTTTAATTGATGGTTTCCTGCGTAATGGGACTAATCA
TCGCACGGACGAATATGGGGGCGTGCCGGAAAACCGCATTCGTTTTTTGAAGGAAG
TGACGGAGCGCGTCATTGCGGCGATTGGGGCGGATCGCACTGGCGTGCGTCTGAGC
CCTAACGGGGACACCGTGGGCTGCATCGACAGTGCCCCGGAGACAGTGTTCGTGCC
GGCAGCGAAATTGCTGCAAGATCTGGGGGTAGCGTGGCTGGAACTTCGTGAACCGG
GCCCGAACGGCACCTTCGGGAAAACCGATCAGCCGAAATTATCTCCGCAGATCCGT
AAAGTGTTCCTGCGTCCACTGGTGCTCAATCAGGATTACACATTTGAAGCGGCGCAG
ACCGCCCTCGCGGAAGGTAAAGCGGACGCGATCGCATTTGGCCGGAAATTCATCTC
TAATCCCGACCTCCCTGAGCGTTTTGCGCGGGGCATTGCGCTGCAACCCGATGACAT
GAAAACGGTTGGTTCTCAAGGCCCGGAAGGTTACACCGATTATCCGTCCGCCACGTC
AGGCCCGAACTAA

GlowER H172G F100V M102L F66L Q232V M105V E181G F343G F342V A69M T341G Protein Sequence
MPTLFDPIDFGPIHAKNRIVMSPLTRGRADKEAVPTPIMAEYYAQRASAGLIITEATGISR
EGLGLPFMPGIWSDAQVEAWKPIVAGVHAKGGKIVCQLVHLGRVVHSSVTGTQPVSSS
ATTAPGEVHTYEGKKPFEQARAIDAADISRILNDYENAARNAIRAGFDGVQIGAANGFLI
DGFLRNGTNHRTDEYGGVPENRIRFLKEVTERVIAAIGADRTGVRLSPNGDTVGCIDSAP
ETVFVPAAKLLQDLGVAWLELREPGPNGTFGKTDQPKLSPQIRKVFLRPLVLNQDYTFE
AAQTALAEGKADAIAFGRKFISNPDLPERFARGIALQPDDMKGVGSQGPEGYTDYPSAT
SGPN*

GlowER H172G F100V M102L F66L Q232V M105V E181G F343G F342V A69M T341G DNA Sequence
ATGCCGACCCTGTTTGATCCTATTGATTTTGGCCCCATCCACGCCAAAAATCGTATCG
TTATGAGCCCGCTGACTCGTGGCCGTGCCGACAAAGAGGCCGTCCCGACCCCGATC
ATGGCGGAATATTATGCCCAACGGGCAAGCGCCGGACTGATCATTACGGAAGCAAC
GGGAATCTCACGCGAAGGCCTGGGTCTGCCGTTCATGCCAGGCATTTGGAGCGACG
CGCAAGTTGAGGCCTGGAAACCCATTGTTGCAGGCGTCCACGCCAAAGGTGGTAAA
ATTGTTTGCCAATTGGTTCACCTGGGCCGTGTTGTGCATTCGAGCGTGACGGGTACG
CAACCAGTTAGCAGCAGCGCCACAACCGCTCCGGGGGAAGTGCATACCTACGAAGG
GAAAAAGCCGTTCGAACAAGCCCGCGCTATTGATGCCGCCGATATTAGTCGGATTTT
AAACGATTATGAAAACGCGGCCCGCAATGCCATCCGTGCGGGTTTCGATGGTGTTCA
GATTGGTGCGGCAAACGGATTTTTAATTGATGGTTTCCTGCGTAATGGGACTAATCA
TCGCACGGACGAATATGGGGGCGTGCCGGAAAACCGCATTCGTTTTTTGAAGGAAG
TGACGGAGCGCGTCATTGCGGCGATTGGGGCGGATCGCACTGGCGTGCGTCTGAGC
CCTAACGGGGACACCGTGGGCTGCATCGACAGTGCCCCGGAGACAGTGTTCGTGCC
GGCAGCGAAATTGCTGCAAGATCTGGGGGTAGCGTGGCTGGAACTTCGTGAACCGG
GCCCGAACGGCACCTTCGGGAAAACCGATCAGCCGAAATTATCTCCGCAGATCCGT
AAAGTGTTCCTGCGTCCACTGGTGCTCAATCAGGATTACACATTTGAAGCGGCGCAG
ACCGCCCTCGCGGAAGGTAAAGCGGACGCGATCGCATTTGGCCGGAAATTCATCTC

TAATCCCGACCTCCCTGAGCGTTTTGCGCGGGGCATTGCGCTGCAACCCGATGACAT
GAAAGGTGTTGGTTCTCAAGGCCCGGAAGGTTACACCGATTATCCGTCCGCCACGTC
AGGCCCGAACTAA

H. Deuterium incorporation studies

Deuterated KP_i pH 8 was prepared by lyophilizing protonated KP_i pH 8 followed by reconstitution with an equal volume of H_2O. Mixed solvents were prepared by combining deuterated and protonated buffers (v/v)

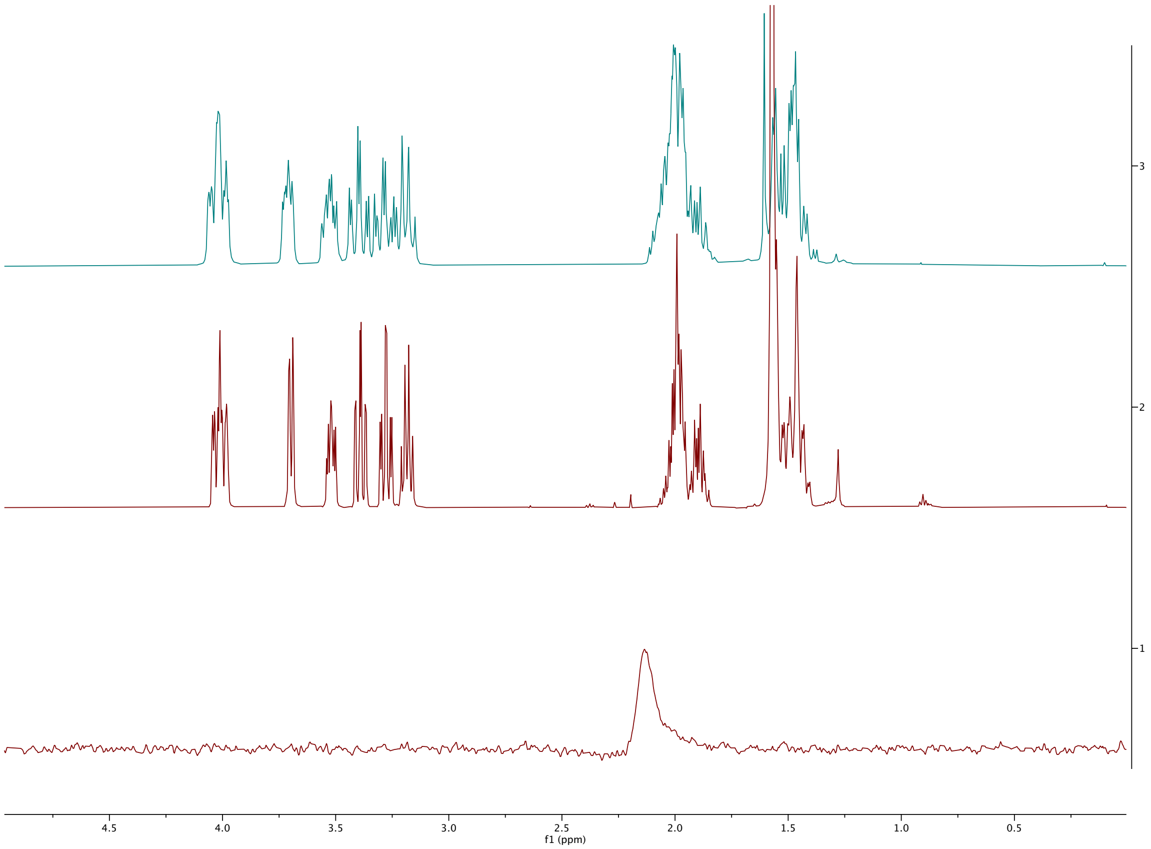

Fig. S1. Comparison of ^{1}H NMR spectra of **2** prepared in H_2O (top) and D_2O (middle) buffers and ^{2}H NMR spectrum **2** prepared in D_2O (bottom) buffer highlighting the disappearance of a resonance at ~2.07 ppm for the β-aniline proton and the collapse of the α-proton at 3.72 ppm to a doublet. The spectra changes are consistent with deuterium incorporation β to the aniline.

Table S5: Yield and e.r. in deuterated buffer for enzymes across engineering campaign

**Mutations added to GlowER**	**Yield (%)**	**e.r. (D_2O)**	**e.r (H_2O)**
+H172G	24	56:45	52:48
+ F100V/M102L	44	66:34	55:45
+ F66L	38	69:31	55:45
+ Q232V	34	75:25	60:40
+ E181G	28	89:11	78:22
+ F343G	30	91:9	80:20
+ F342V	17	93:7	89:11
+ A69M/T341G	33	96:4	92:8

Derivation of relationship between e.r. in H_2O/D_2O and enantiomer KIEs

If hydrogen transfer is enantiodetermining, then:

$$e.r._H = \frac{k_{H(S)}}{k_{H(R)}} \text{ and } e.r._D = \frac{k_{D(S)}}{k_{D(R)}}$$

The KIE for each enantiomer is:

$$KIE_S = \frac{k_{H(S)}}{k_{D(S)}} \text{ and } KIE_R = \frac{k_{H(R)}}{k_{D(R)}}$$

Rearranging:

$$\frac{e.r._H}{e.r_D} = \frac{k_{H(S)}}{k_{H(R)}} * \frac{k_{D(R)}}{k_{D(S)}} = \frac{KIE_S}{KIE_R}$$

I. UV-vis spectroscopic details

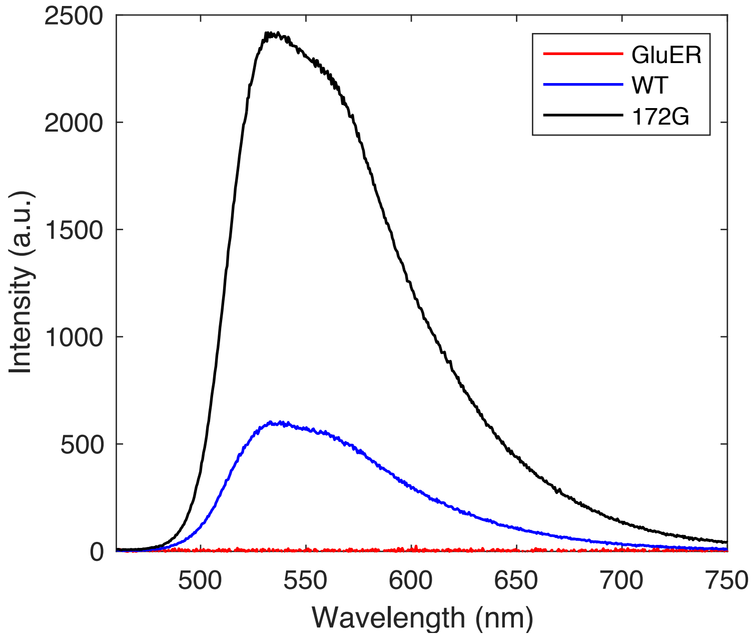

Figure S2: Fluorescence spectra of GluER T36A (red), GlowER WT (blue) and GlowER H172G (black) showing the increased fluorescence intensity from GluER to GlowER to GlowER H172G. Excitation wavelength: 450 nm.

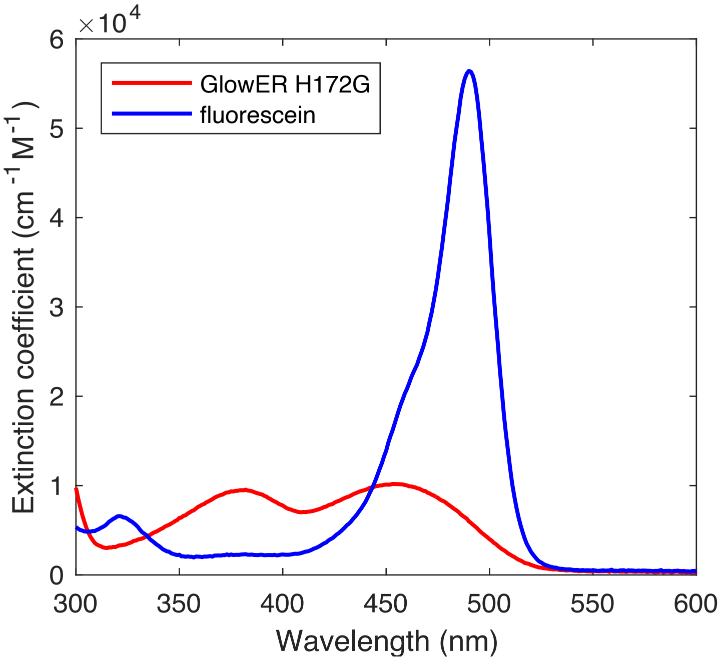

Figure S3: Absorbance of GlowER H172G and fluorescein overlaid at identical concentrations. At 456 nm, GlowER H172G and fluorescein have similar extinction coefficients.

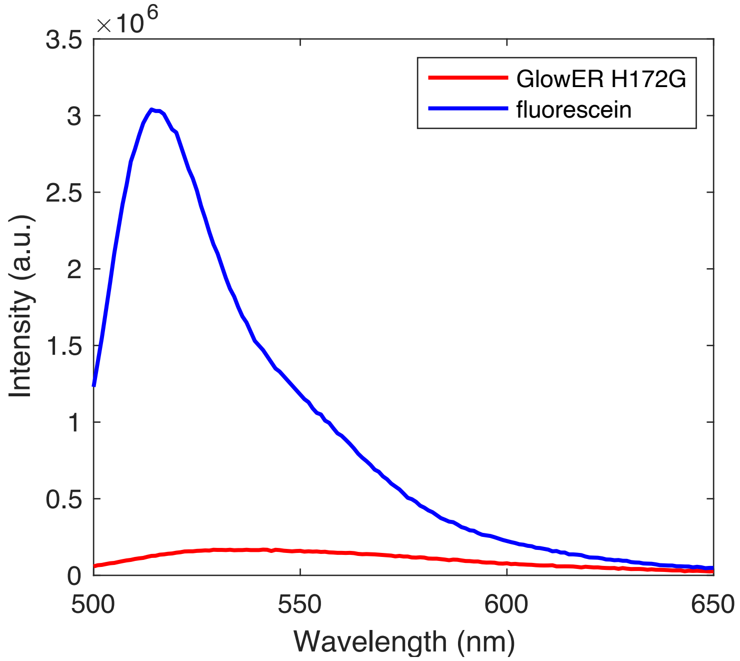

Figure S4: Emission of GlowER H172G and fluorescein overlaid at identical concentrations (450 nm excitation). Fluoresein fluoresces much more intensely than GlowER H172G.

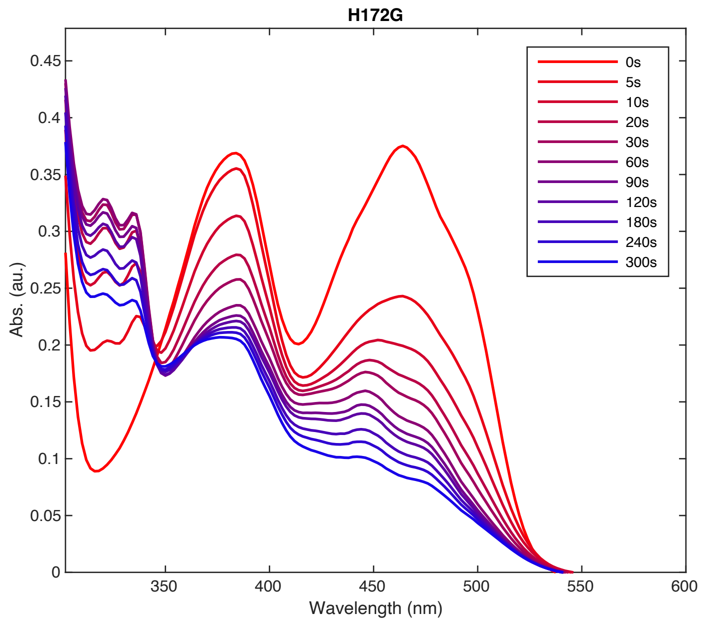

Figure S5: Photoreduction timecourse for GlowER H172G from 0 to 300s of irradiation with 456 nm light.

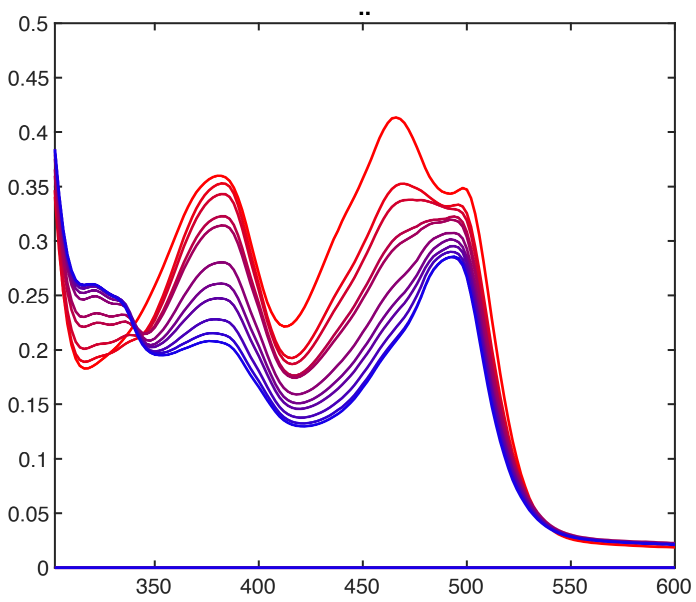

Figure S6: Photoreduction timecourse for GlowER H172G + 1 equiv. fluorescein from 0 to 300s of irradiation with 456 nm light with contributions from fluorescein subtracted with an independent timecourse of fluorescein photoreduction (Fig. S5). Much lower accumulations of the species at 334 nm are apparent.

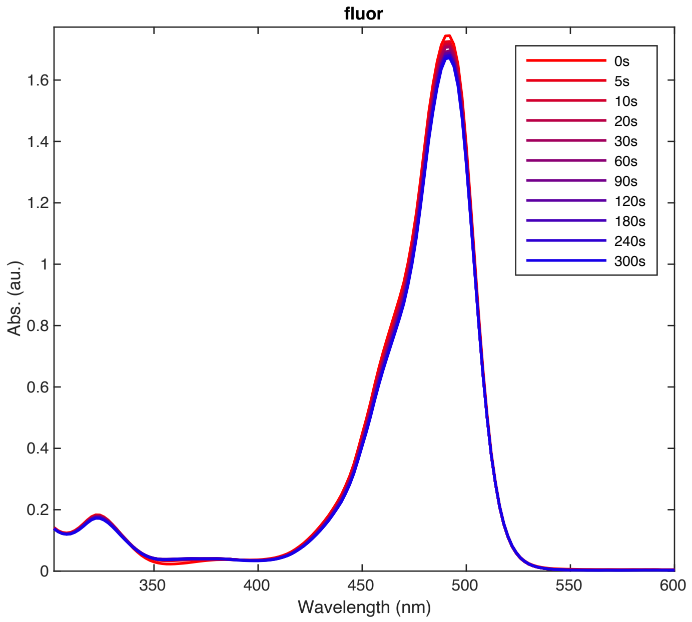

Figure S7: Photoreduction timecourse for fluorescein from 0 to 300s of irradiation with 456 nm light.

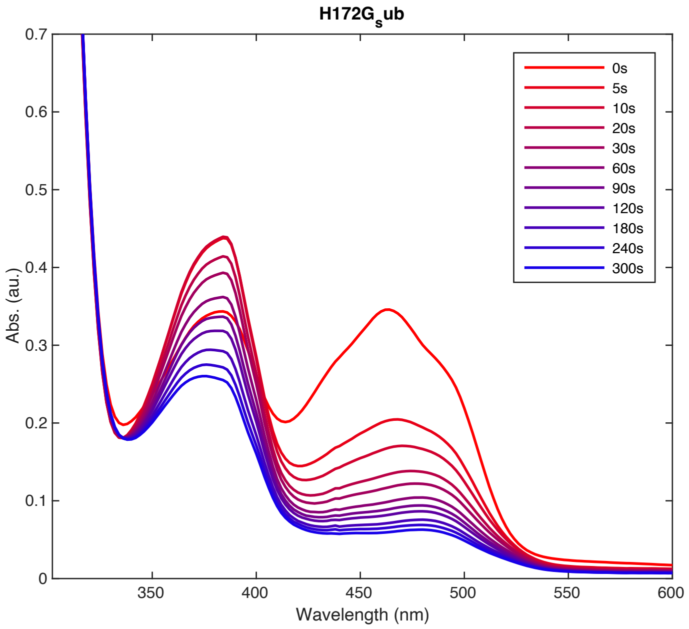

Figure S8: Photoreduction timecourse for GlowER H172G + 100 equiv. substrate **11** from 0 to 300s of irradiation with 456 nm light. Semiquinone (380 nm) accumulates at short timescales

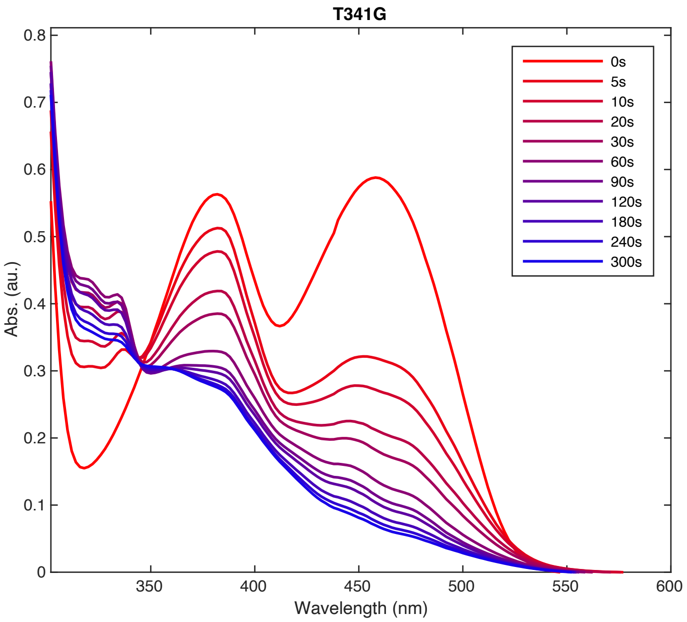

Figure S9: Photoreduction timecourse for GlowHA from 0 to 300s of irradiation with 456 nm light.

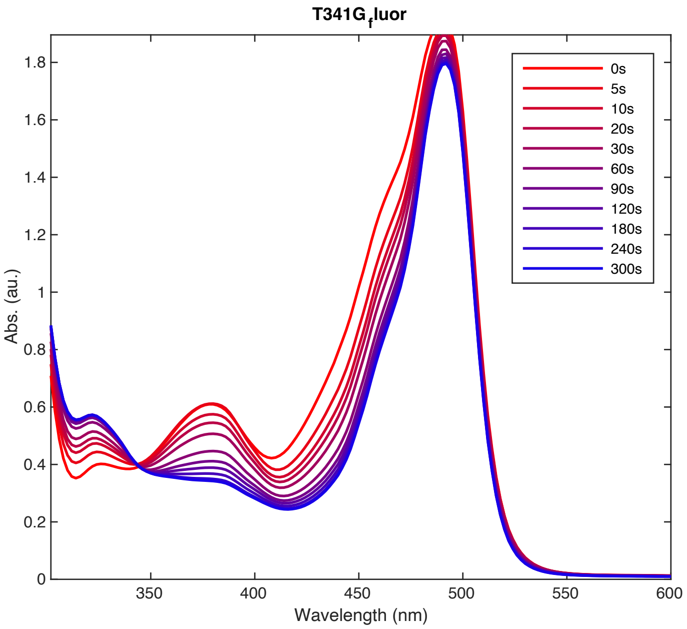

Figure S10: Photoreduction timecourse for GlowHA + 1 equiv. fluorescein from 0 to 300s of irradiation with 456 nm light.

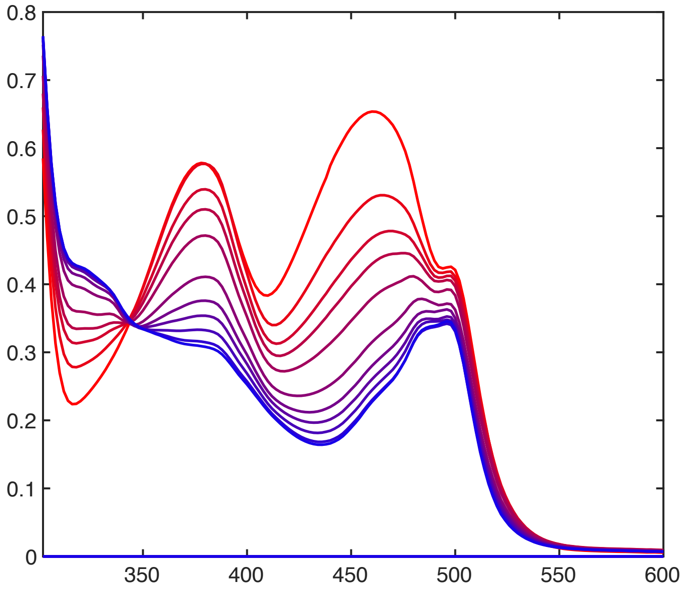

Figure S11: Photoreduction timecourse for GlowHA + 1 equiv. fluorescein from 0 to 300s of irradiation with 456 nm light with contributions from fluorescein subtracted with an independent timecourse of fluorescein photoreduction (Fig. S5). Much lower accumulations of the species at 334 nm are apparent.

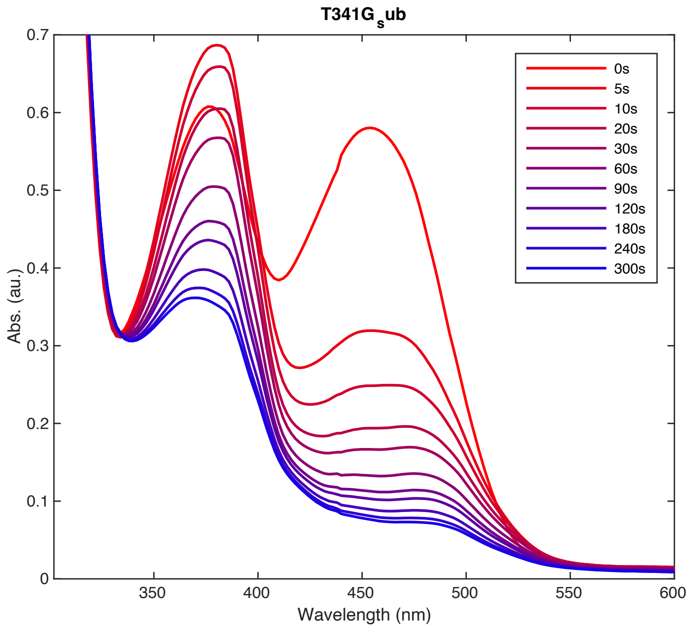

Figure S12: Photoreduction timecourse for GlowHA + 100 equiv substrate from 0 to 300s of irradiation with 456 nm light. Semiquinone accumulates at short timepoints.

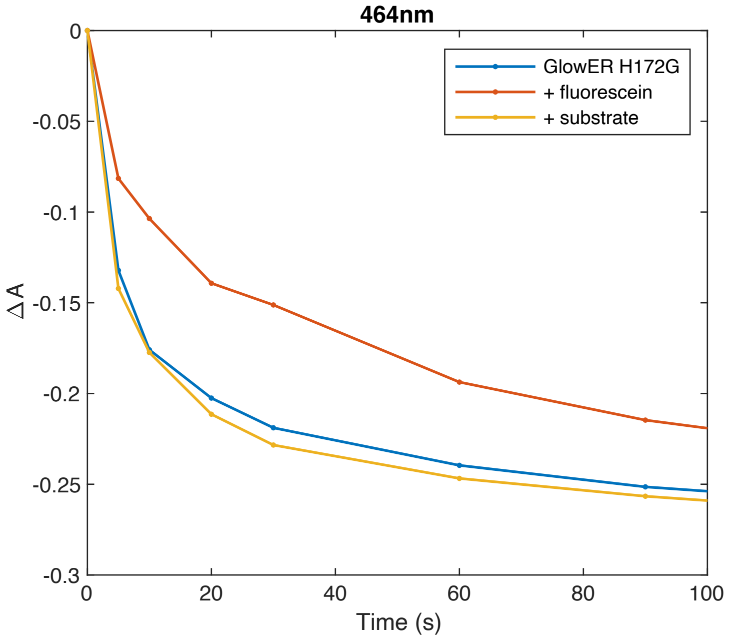

Figure S13: Photoreduction timecourse for GlowER H172G at 464 nm. Enzyme alone (blue), with 1 equiv. fluorescein (orange) or with 100 equiv. substrate (yellow).

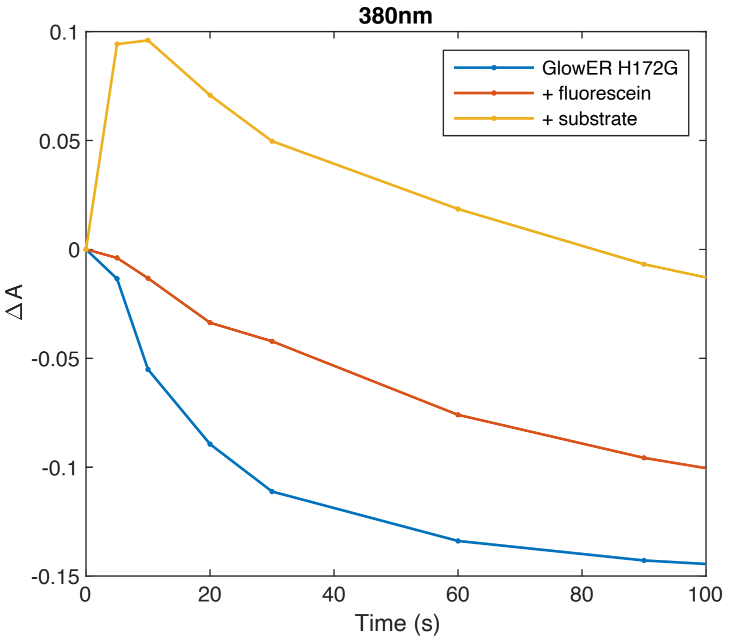

Figure S14: Photoreduction timecourse for GlowER H172G at 380 nm. Enzyme alone (blue), with 1 equiv. fluorescein (orange) or with 100 equiv. substrate (yellow). Absorbance at 380 corresponds to accumulation of the semiquinone.

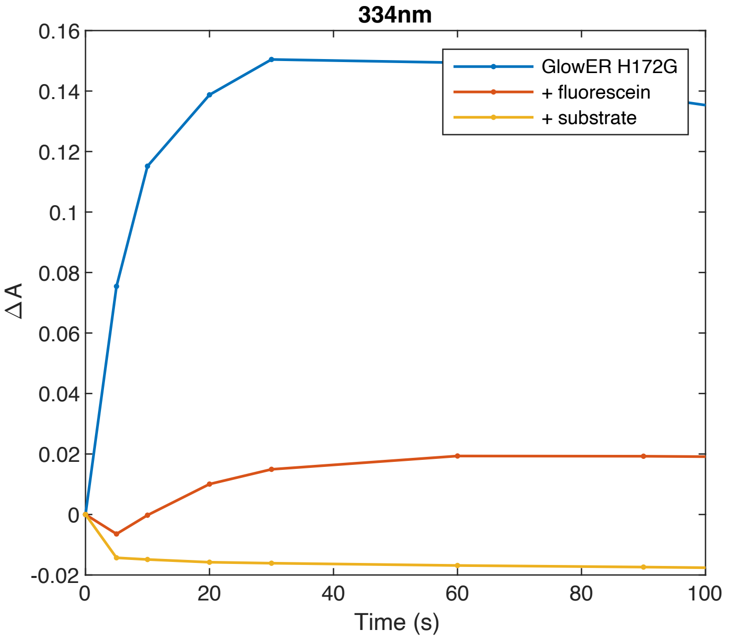

Figure S15: Photoreduction timecourse for GlowER H172G at 334 nm. Enzyme alone (blue), with 1 equiv. fluorescein (orange) or with 100 equiv. substrate (yellow). Both fluorescein and substrate suppress the accumulation of the 334 nm species

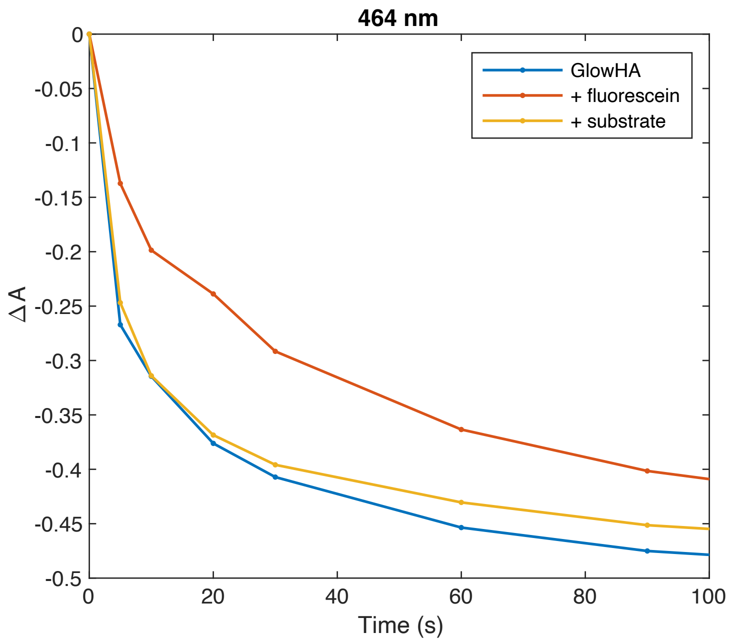

Figure S16: Photoreduction timecourse for GlowHA nm. Enzyme alone (blue), with 1 equiv. fluorescein (orange) or with 100 equiv. substrate (yellow).

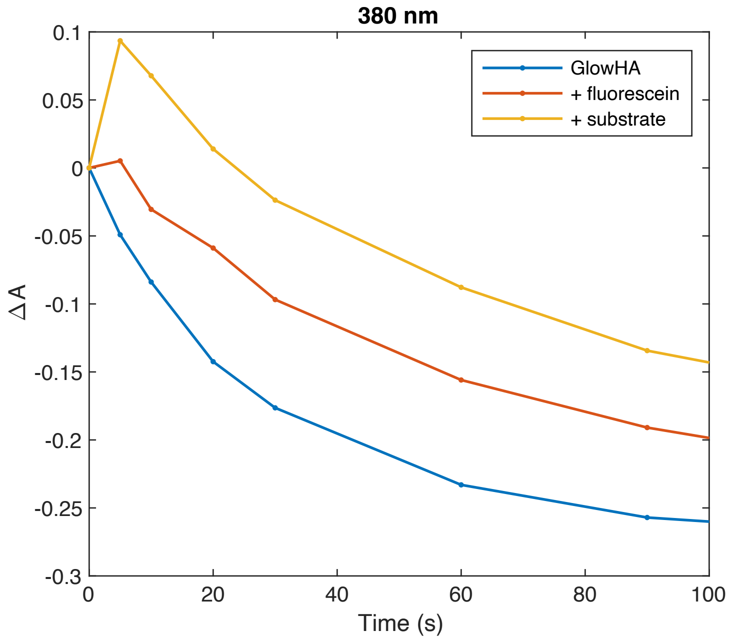

Figure S17: Photoreduction timecourse for GlowER H172G at 380 nm. Enzyme alone (blue), with 1 equiv. fluorescein (orange) or with 100 equiv. substrate (yellow). Absorbance at 380 corresponds to accumulation of the semiquinone.

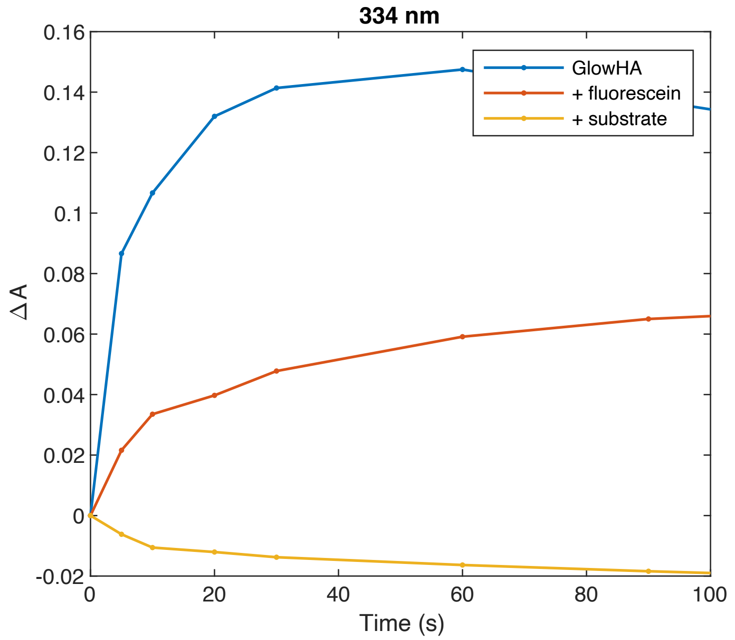

Figure S18: Photoreduction timecourse for GlowER H172G at 334 nm. Enzyme alone (blue), with 1 equiv. fluorescein (orange) or with 100 equiv. substrate (yellow). Both fluorescein and substrate suppress the accumulation of the 334 nm species

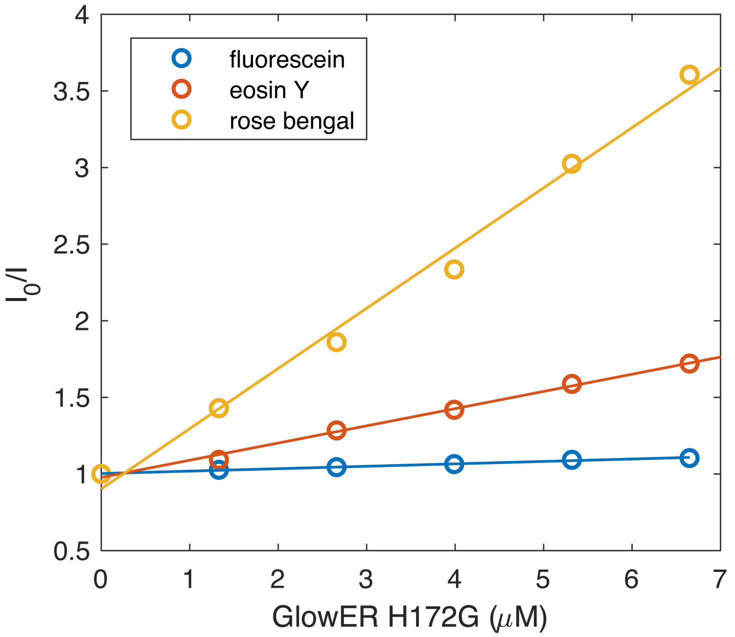

Figure S19: Static fluorescence quenching of fluorescein, eosin Y and rose bengal fluorescence by GlowER H172G. The dissociation constant (K_D) for the photocatalysts can be extracted as the reciprocal of the slope.

J. Transient Absorption Spectroscopic Details

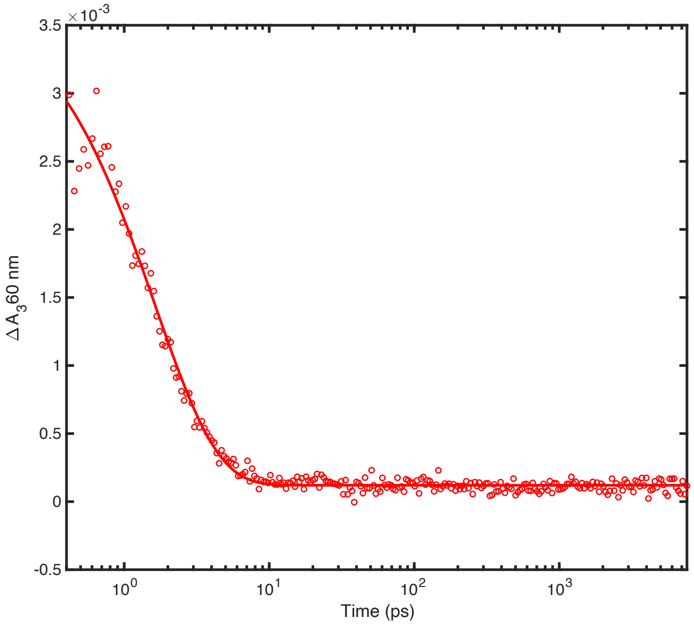

Figure S20: Decay and fit for the excited state absorbance of GluER T36A at 360 nm. τ = 1.63 ps.

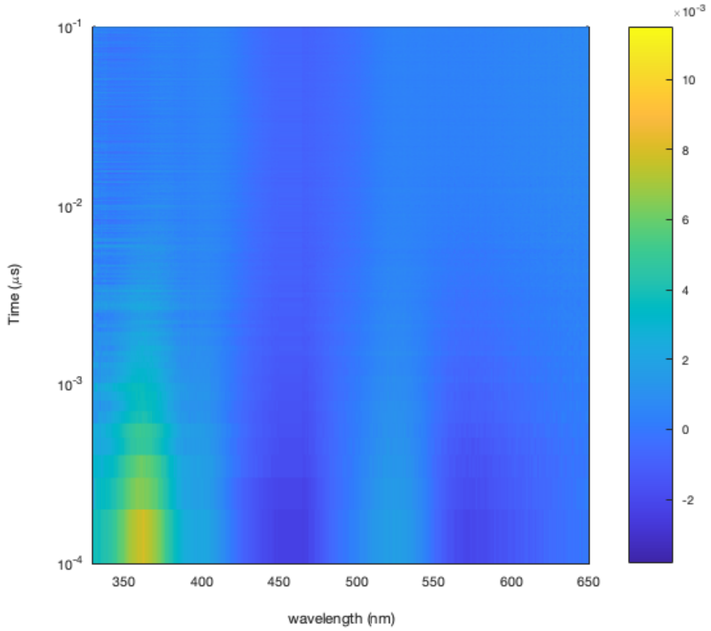

Figure S21: TA surface for photoexcitation of GlowER

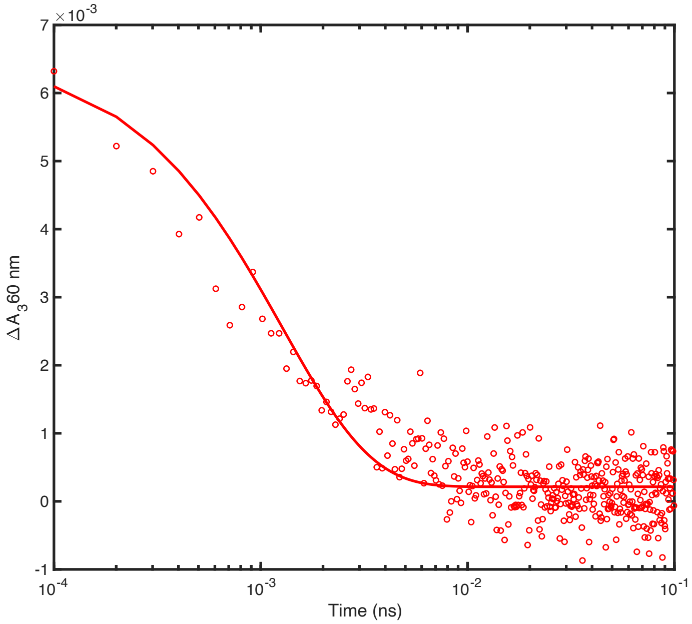

Figure S22: Decay and fit for the excited state absorbance of GlowER at 360 nm. $\tau = 1.25$ ns.

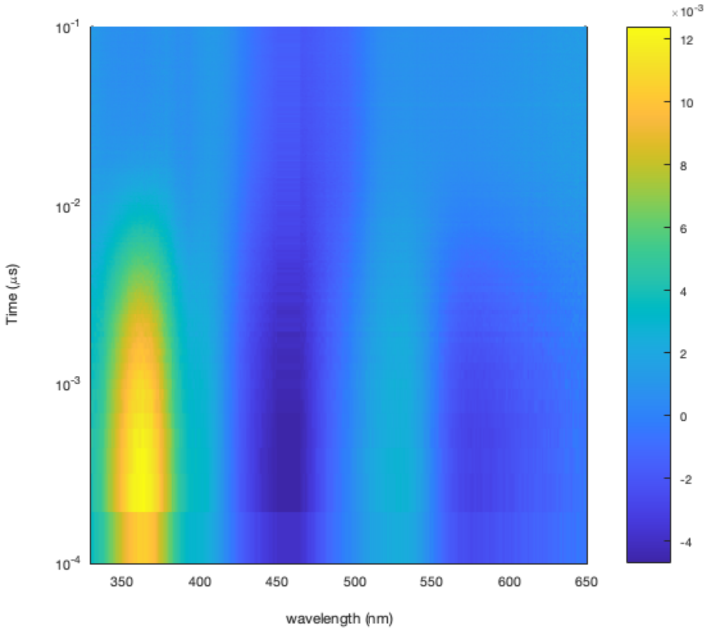

Figure S23: TA surface for photoexcitation of GlowER H172G.

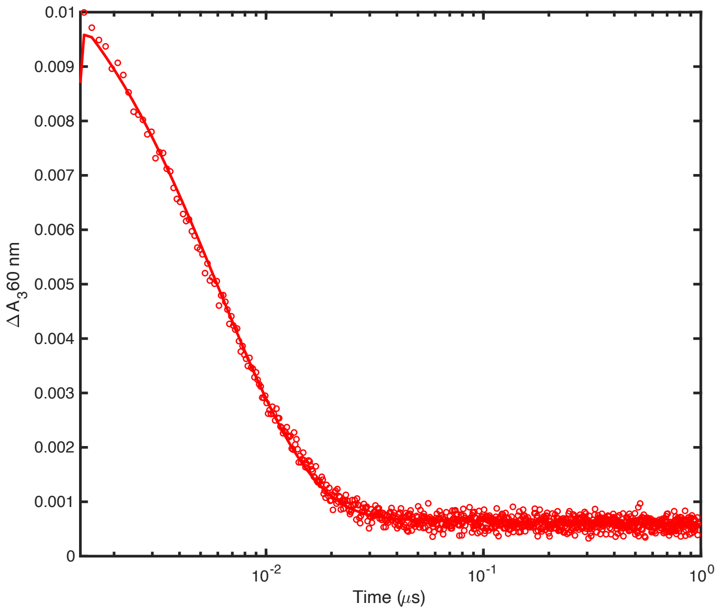

Figure S24: Decay and fit for the excited state absorbance of GlowER H172G at 350 nm. τ = 6.1 ns.

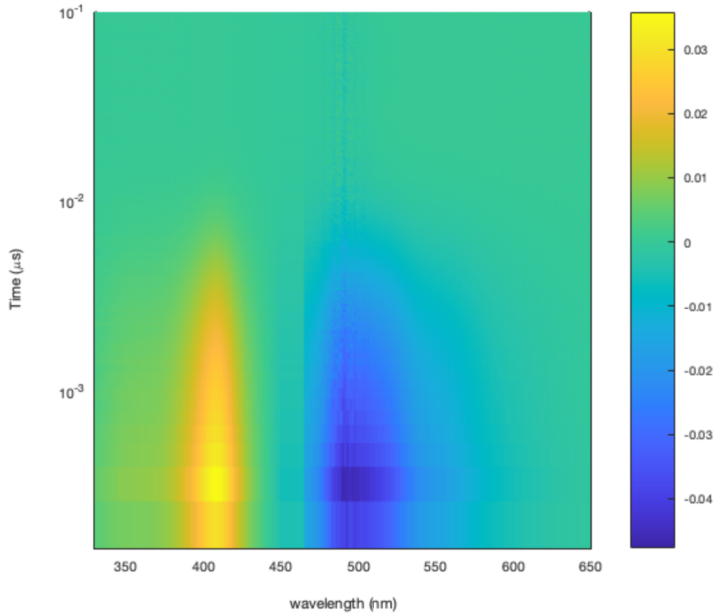

Figure S25: TA surface for photoexcitation of GlowER H172G + 1 equiv. fluorescein

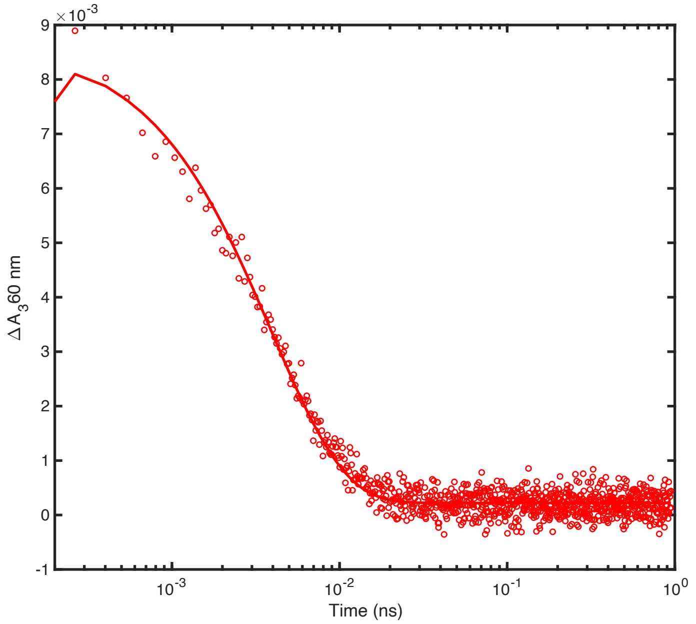

Figure S26: Representative decay and fit for the excited state absorbance of GlowER H172G + 1 equiv. fluorescein at 350 nm. τ = 3.97 ns.

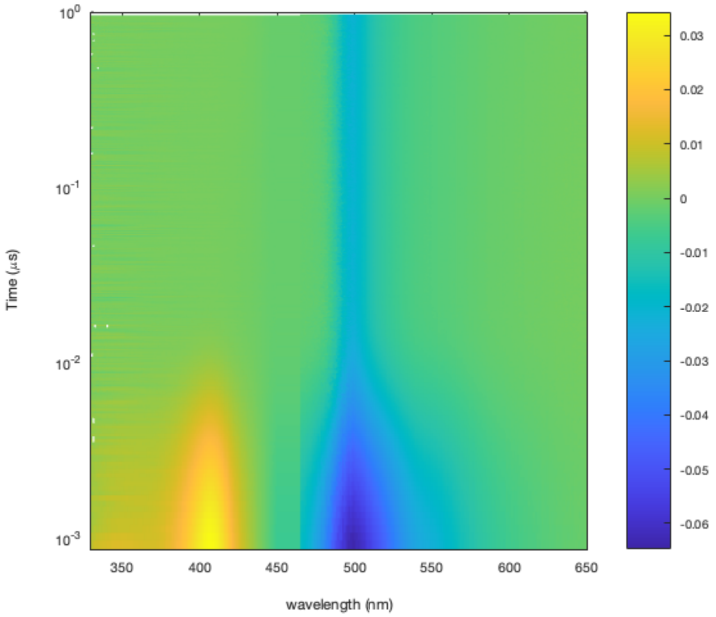

Figure S27: TA surface for photoexcitation of fluorescein + 1 equiv. GlowER H172G

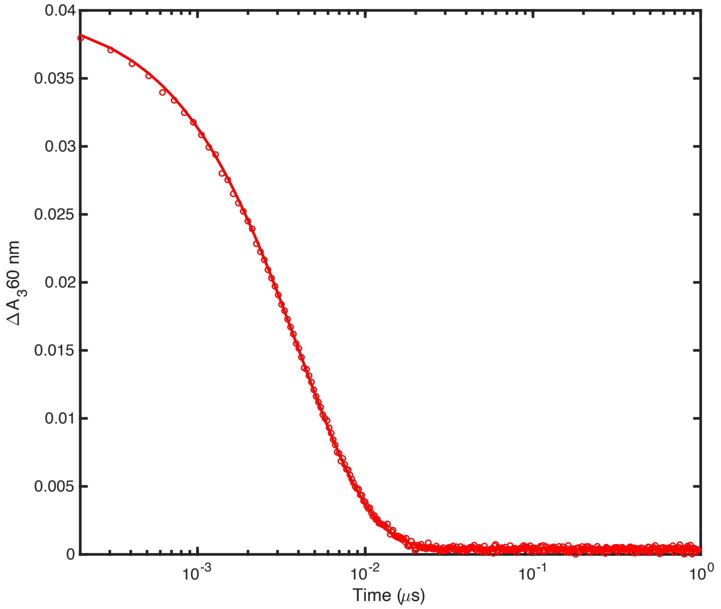

Figure S28: Decay and fit for the excited state absorbance of fluorescein + 1 equiv. GlowER H172G at 405 nm. τ = 4.02 ns.

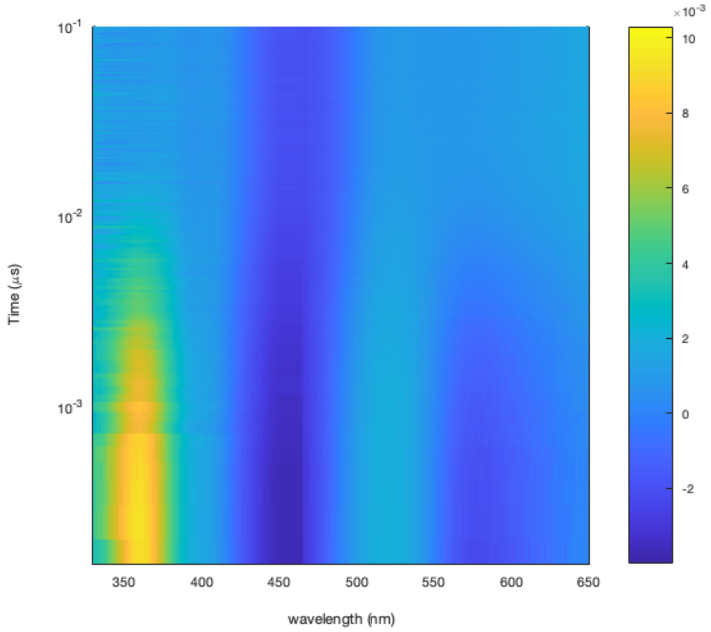

Figure S29: TA surface for photoexcitation of GlowHA

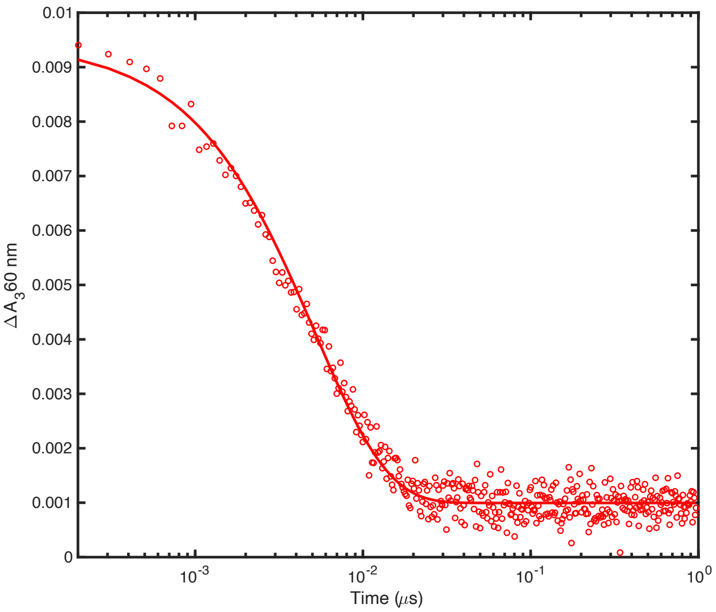

Figure S30: Decay and fit for the excited state absorbance of GlowHA at 350 nm. τ = 5.16 ns.

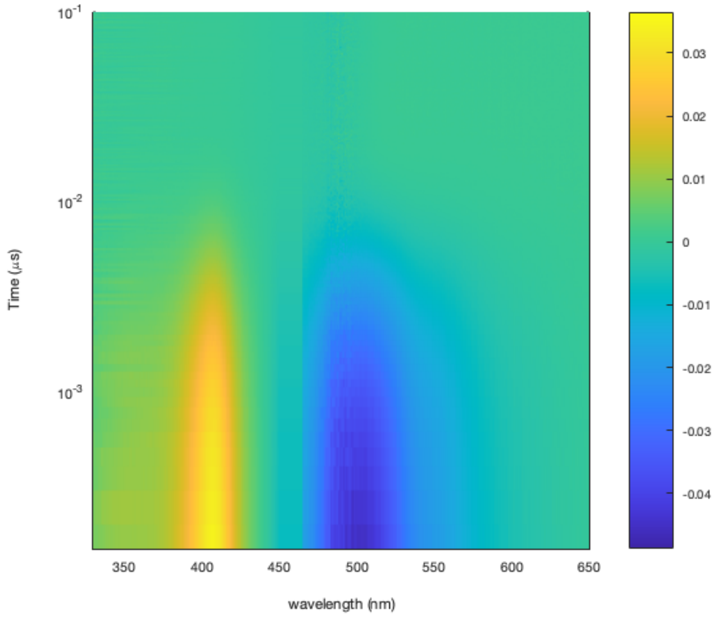

Figure S31: TA surface for photoexcitation of GlowHA + 1 equiv. fluorescein

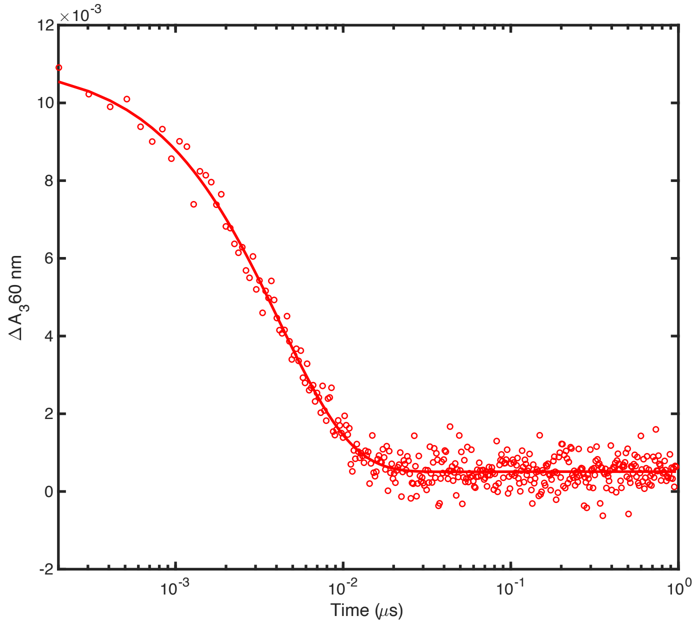

Figure S32: Decay and fit for the excited state absorbance of GlowHA + 1 equiv. fluorescein at 350 nm. τ = 4.16 ns.

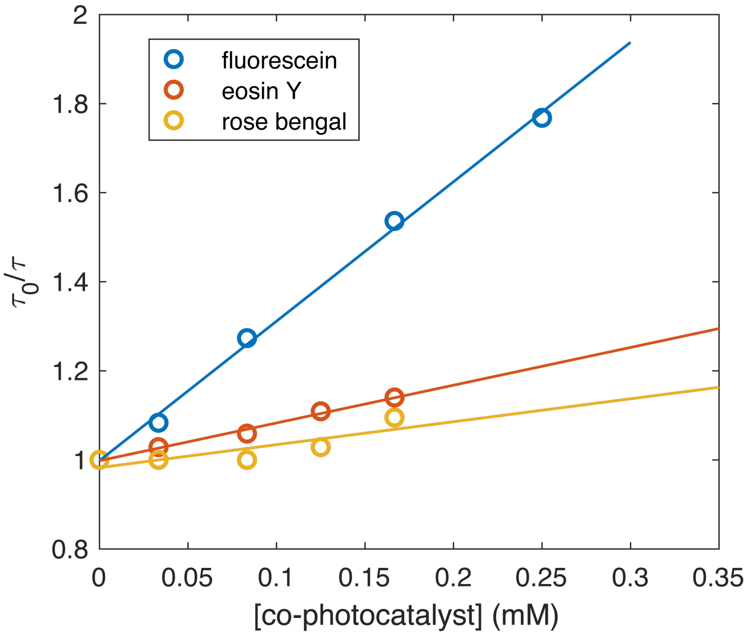

Figure S33: Excited state lifetime quenching of GlowHA with addition of fluorescein, eosin Y, and rose bengal

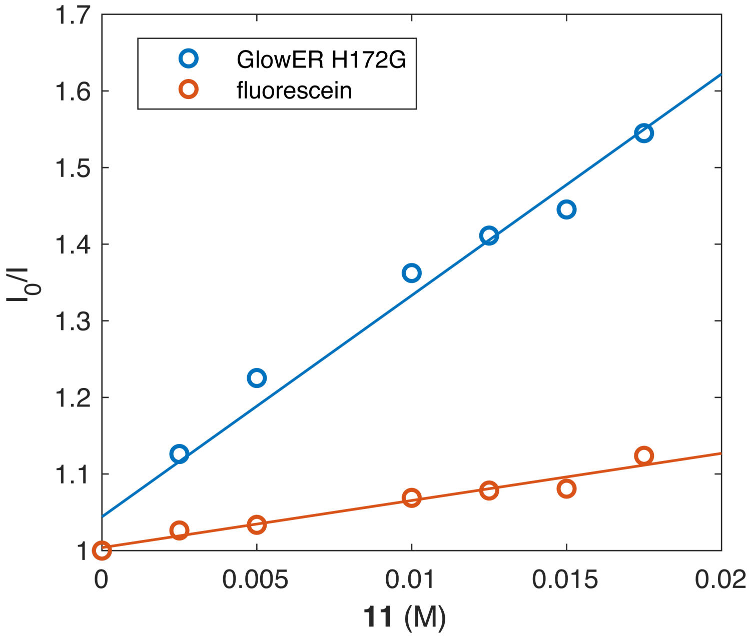

Figure S34: Steady-state fluorescence quenching of GlowER H172G and fluorescein by addition of **11**

K. Biochemical characterization of GlowHA and GlowER

Table S6: Protein concentration (mg/mL) obtained via UV-vis (464 nm, $\varepsilon = 12.0\ cm^{-1}\ M^{-1}$) and Bradford analysis for a 100-fold dilution of the aliquoted protein. The identical concentrations obtained via UV-vis and Bradford analysis indicate that ~100% of the protein purified with added FMN contains a bound flavin cofactor.

Variant	FMN added in purification	Conc. (UV-vis, mg/mL)	Conc. (Bradford, mg/mL)
GlowER H172G	1 mg/mL	0.71	0.70
GlowHA	1 mg/mL	0.92	0.90
GlowER H172G	none	0.48	1.81
GlowHA	none	0.23	1.43

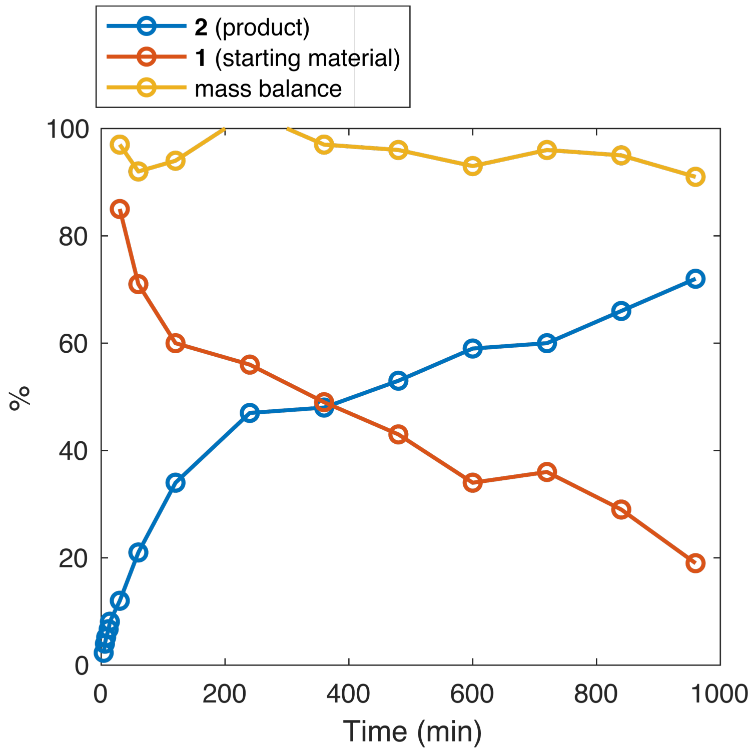

Figure S35: Timecourse for the reaction of GlowHA with **1**.

Table S7: Recharge experiments. Substrate **1** was subjected to the standard reaction conditions (10 umol **1**, 150 nmol GlowHA, 150 nmol fluorescein, KPi pH 8, 5% DMSO, 2mL total volume, 16h irradiation with 456 nm Kessils).

After 16 h, an additional 10 umol of **1**, plus either additional GlowHA or fluorescein was added and further conversion monitored. Table shows additional yield (above the 7.2 μmol typically obtained after 16 h)

Components added after 16 h	% of 1 added at 16 h converted to 2
10 umol **1**	21
10 umol **1**, 150 nmol GlowHA	46
10 umol **1**, 150 nmol fluorescein	24

This experiment shows that the enzyme retains some activity after 16 h of irradiation, with the overall reaction rate suppressed by the depletion of **1**. Addition of fluorescein after 16h does not increase the conversion, indicating that this co-catalyst has not been depleted over

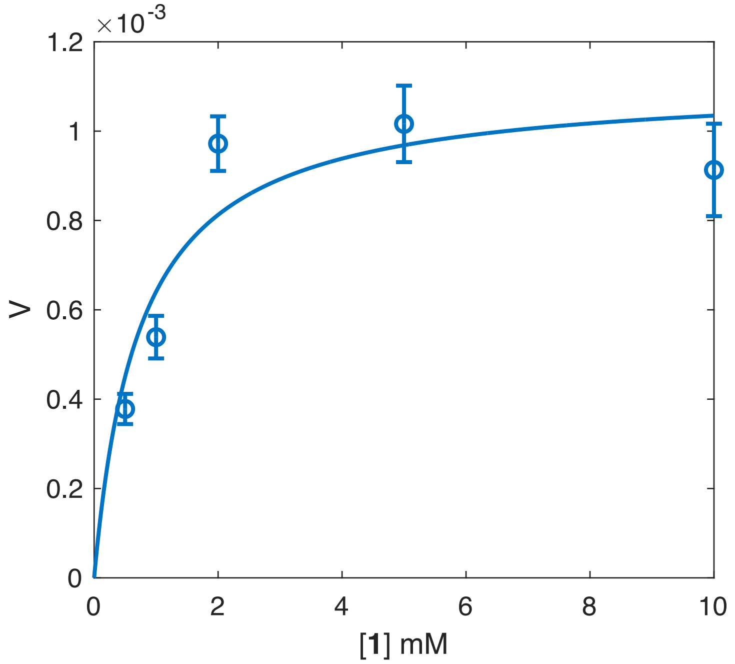

Figure S36: Michaelis-Menten kinetics for the hydroamination of **1** by GlowHA. Fitting gives V_{max} = 1.11 μM/s, K_m = 780 μM, and k_{cat} = 0.44 min^{-1}. Conditions: 100 nmol GlowHA, 100 mM KPi pH 8, 10% DMSO, 1 mL total volume.

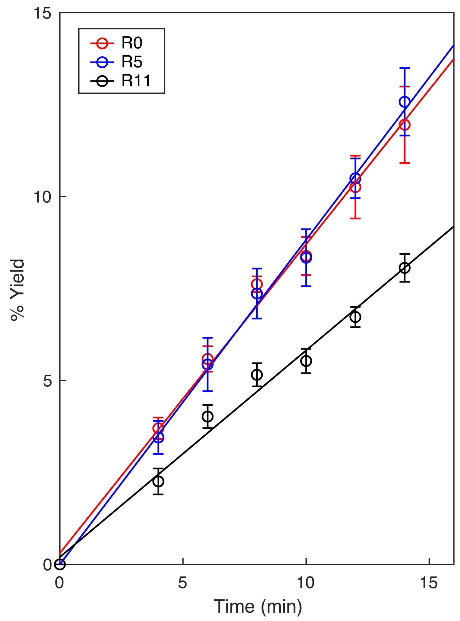

Figure S37: Initial rate kinetics for the hydroamination of **1** by GlowHA under the standard reaction. Conditions: 100 nmol enzyme, 10 μmol **1** 100 mM KPi pH 8, 10% DMSO, 1 mL total volume. R0 = GlowER H172G, R5 = GlowER R5, R11 = GlowHA. Initial rates for GlowER H172G and GlowER R5 and GlowHA were 0.84(7), 0.88(6) nmol/min, and 0.56(7) respectively.

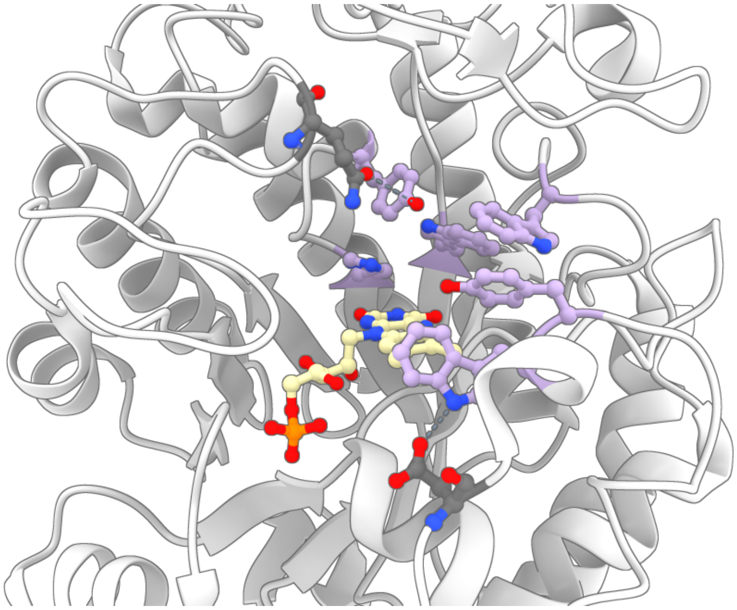

Figure S38: GluER active site showing H-bonds to residues modified to constrct GlowER. Two hydrogen bonds are modified: Y100-Q232 and W342-D337

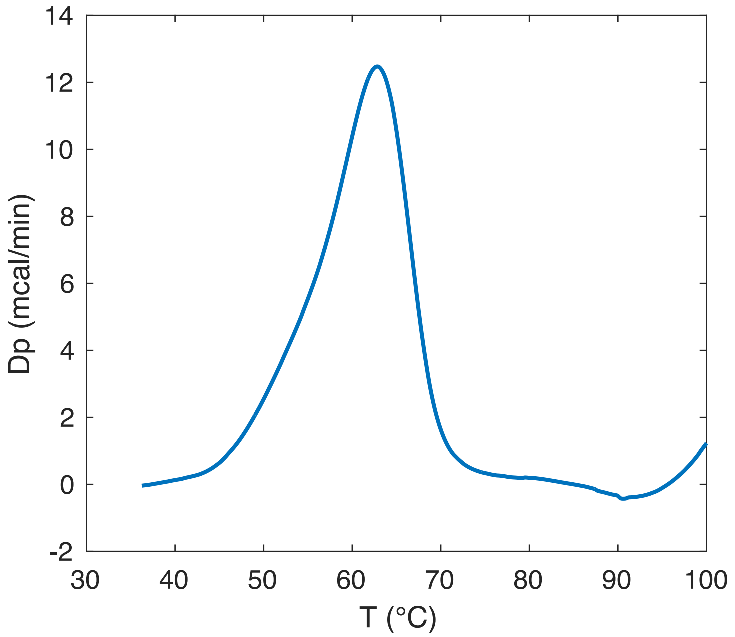

Figure S39: DSC curve for GlowER H172G showing a melting point of 62.8 °C

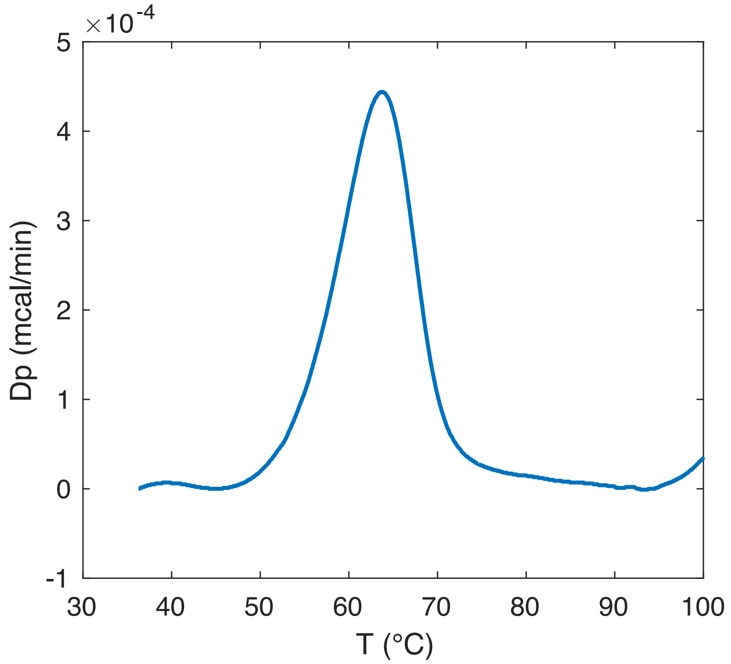

Figure S40: DSC curve for GlowHA showing a melting point of 63.7 °C

Residual native activity for GlowER H172G and GlowHA.

The variants of GluER prepared for radical hydroamination have the catalytically essential residues for either NADP+ binding and/or alkene reduction removed. To assess if GlowER variants were capable of (A) FMN Reduction by NADPH and (B) catalytic alkene reduction, we subjected the enzymes to two assays and qualitatively compared their activity to that of GluER T36A.

A. To a sample of 50 µM enzyme (GluER T36A, GlowER H172G, or GlowHA) in KPi pH 8 was added NADPH to a final concentration of 500 µM. Reduction of the flavin cofactor was monitored by UV-vis following the disappearance of the oxidized flavin absorbance at 464 nm.

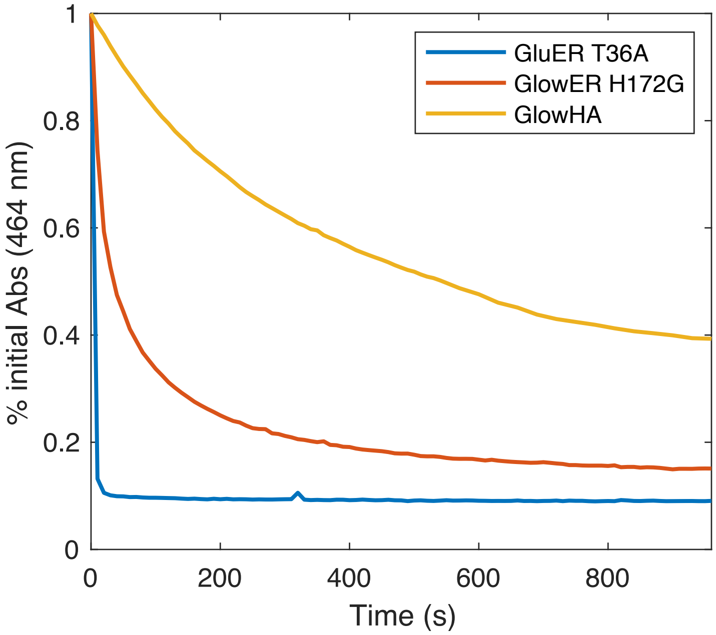

Figure S41: Reduction of GluER T36A, GlowER H172G and GlowHA by NADPH following the disappearance of oxidized flavin at 464 nm.

Table S8: $t_{1/2}$ for reduction of GluER variants by NADPH

**Variant**	**Time for <50% remaining oxidized flavin**
GluER T36A	<10 s
GlowER H172G	40 s
GlowHA	9 min

B. A sample of 50 µM enzyme (GluER T36A, GlowER H172G, or GlowHA) in KPi pH 8 was was mixed with NADPH (500 µM) and allowed to undergo reduction for 30 min. After 30 min, cyclohexenone was added and the reappearance of oxidized FMN was monitored by UV-vis. Reduction of the flavin cofactor was monitored by UV-vis following the disappearance of the oxidized flavin absorbance at 464 nm.

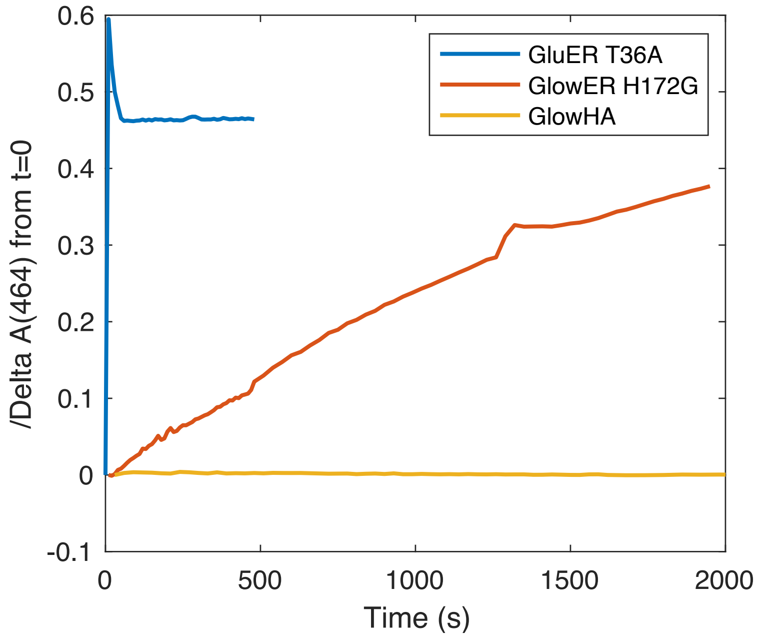

Figure S42: Oxidation of FMN by cyclohexenone monitored by reappearance of the FMN absorbance at 464 nm.

Table S9: $t_{1/2}$ for oxidation of GluER variants by cyclohexeneone

**Variant**	**Time for >50% recovery of initial FMN absorbance**
GluER T36A	<10 s
GlowER H172G	10 min
GlowHA	> 1 h

L. Protein mass spectrometry

Two GlowER H172G samples were prepared for mass spectrometry. Each sample contained 100 nmol of GlowER H172G at a concentration of 4 mg/mL in KPi pH 8. The first was kept in the dark while the second was irradiated with 456 nm light for 30 min. Both samples were dialyzed against MilliQ water to remove the buffer.

Both samples were subjected to trypsin digests as follows: 5 µL of a 1 mg/mL sample was reduced with DTT and alkylated with iodoacetamide using an SP3 protocol. Next, 0.2 µg of trypsin, 50 µl of proteomax 0.01%, 2 mM $CaCl_2$ and 100 mM NH_4CO_2 were added and the sample was incubated for 2 h at 37 °C. The digested peptide was then washed with 100 µL of 50% CAN, 0.1% TFA and the volume was reduced to 20 µL. Lastly, 0.1% TFA was added to a total volume of 50 µL. LC-MS analysis was performed on a Vanquish Flex-Eclipse and analysis was performed using Proteome Discoverer

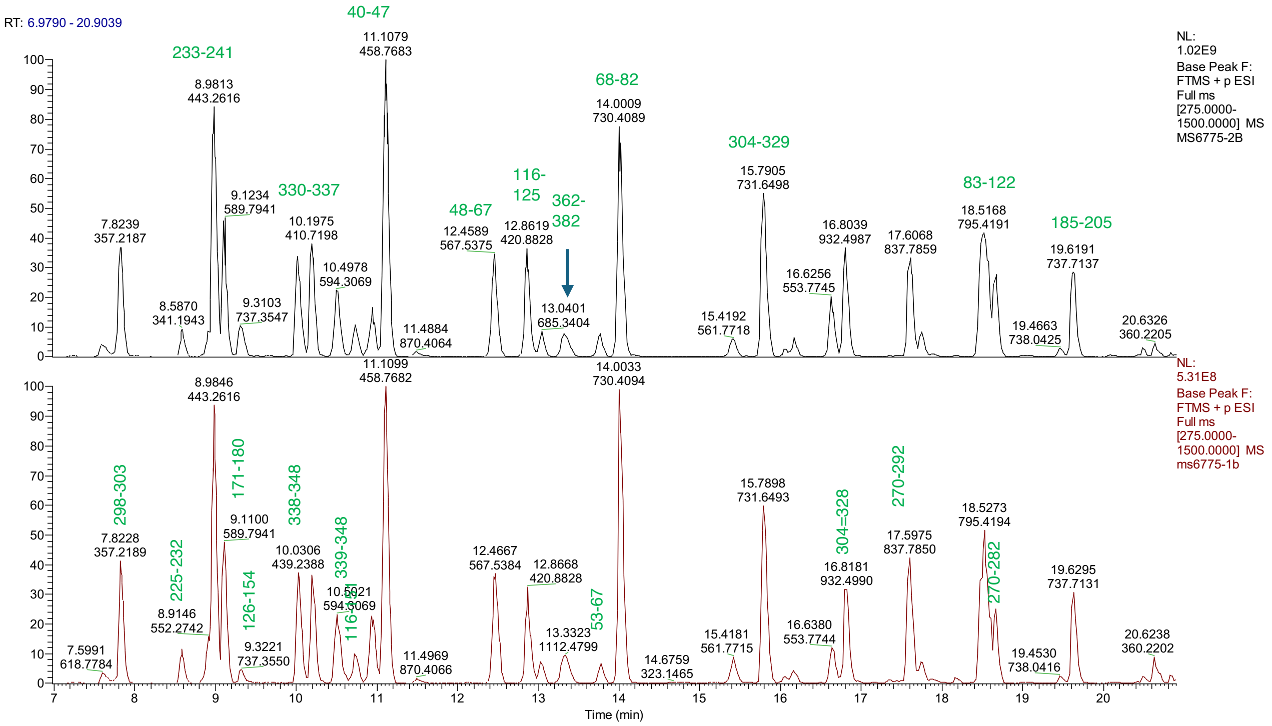

Figure S43: LC-MS base peaks for trypsin digest of GlowER H172G kept in the dark (dark, top) and GlowER H172G irradiated with light for 30 min (irradiated, bottom). The base peaks are very similar in both samples.

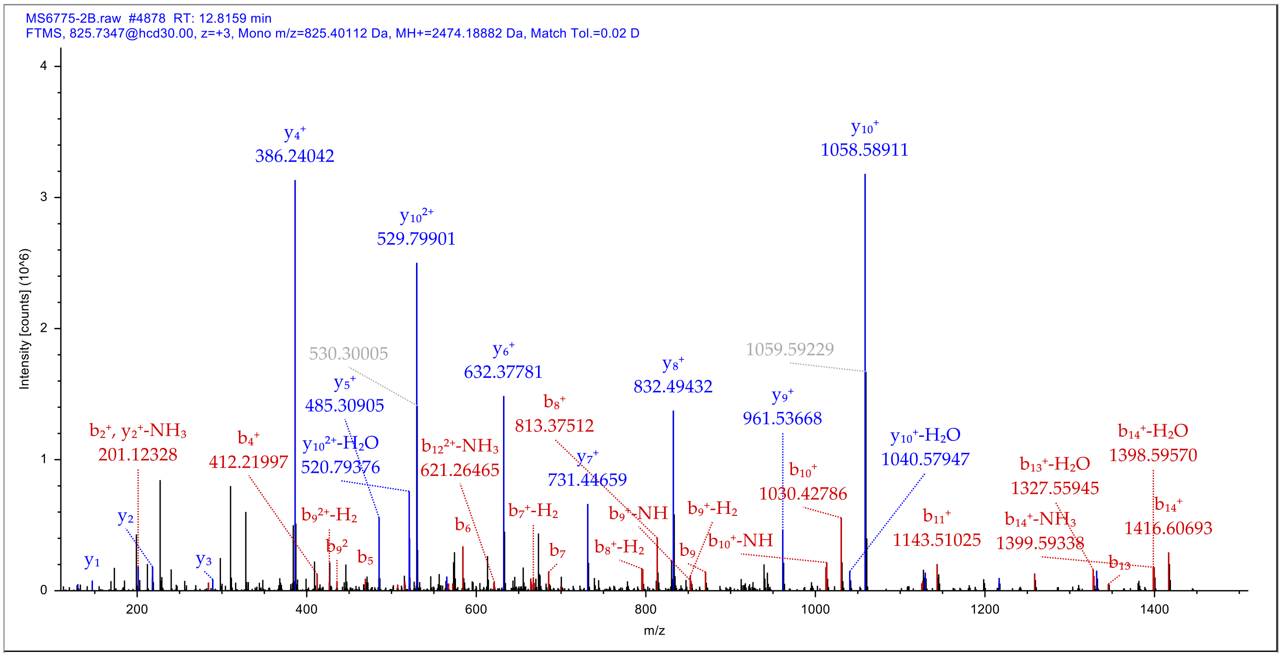

Figure S44: MS-MS for the unmodified peptide fragment 224-LSPNGDTQGCIDSAPETVFVPAAK-248 in the dark GlowER H172G sample.

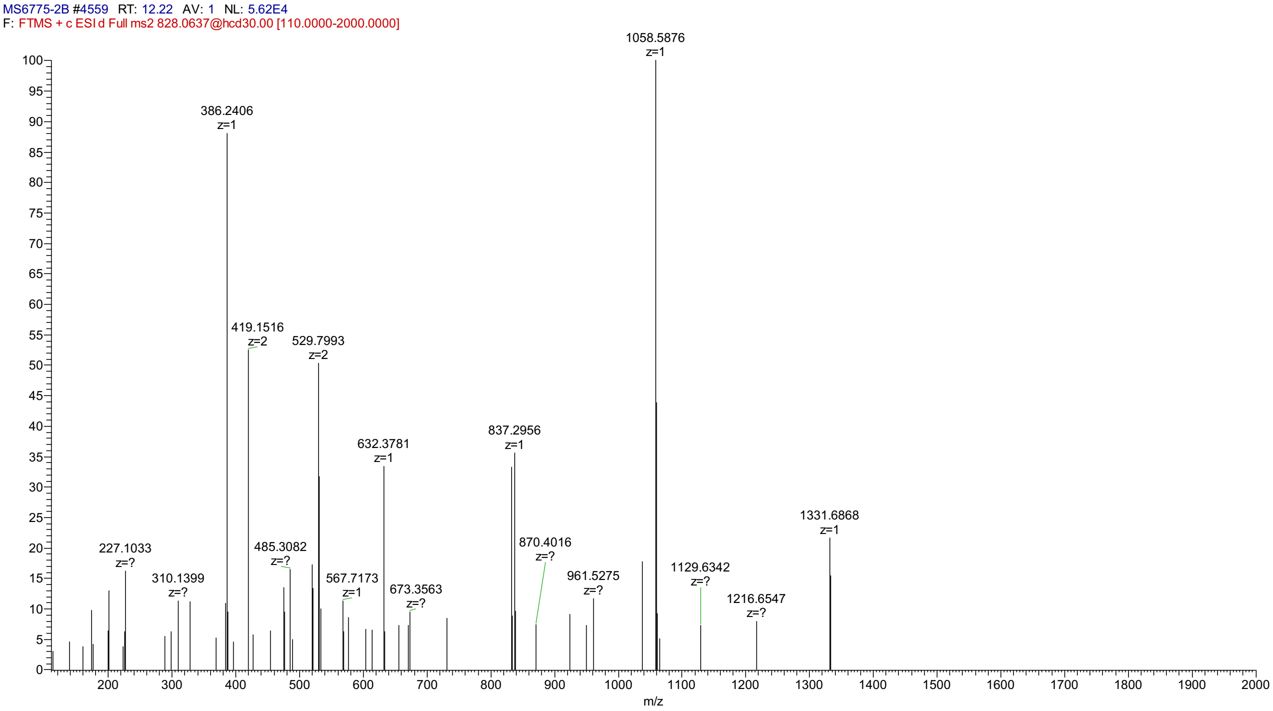

Figure S45: MS-MS for a modified peptide fragment derived from 224-LSPNGDTQGCIDSAPETVFVPAAK-248 that is only found in the irradiated GlowER H172G sample

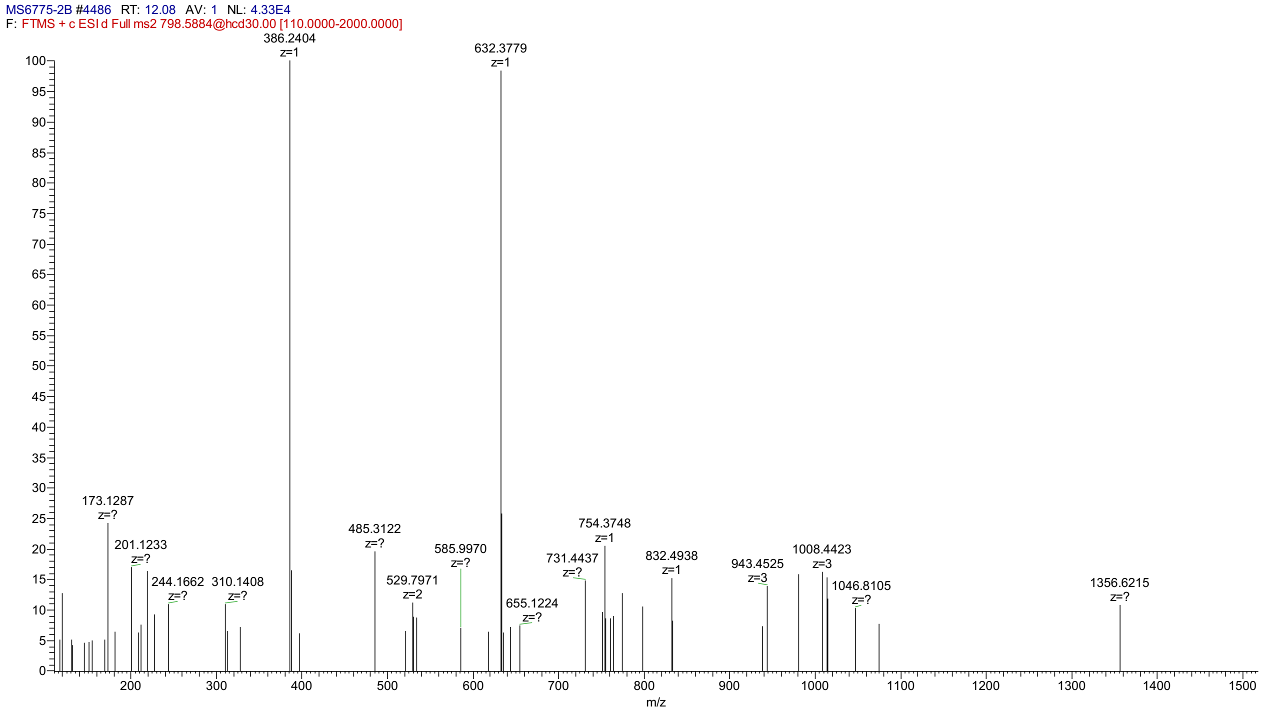

Figure S46: MS-MS for a second for a modified peptide fragment derived from 224-LSPNGDTQGCIDSAPETVFVPAAK-248 that is only found in the irradiated GlowER H172G sample

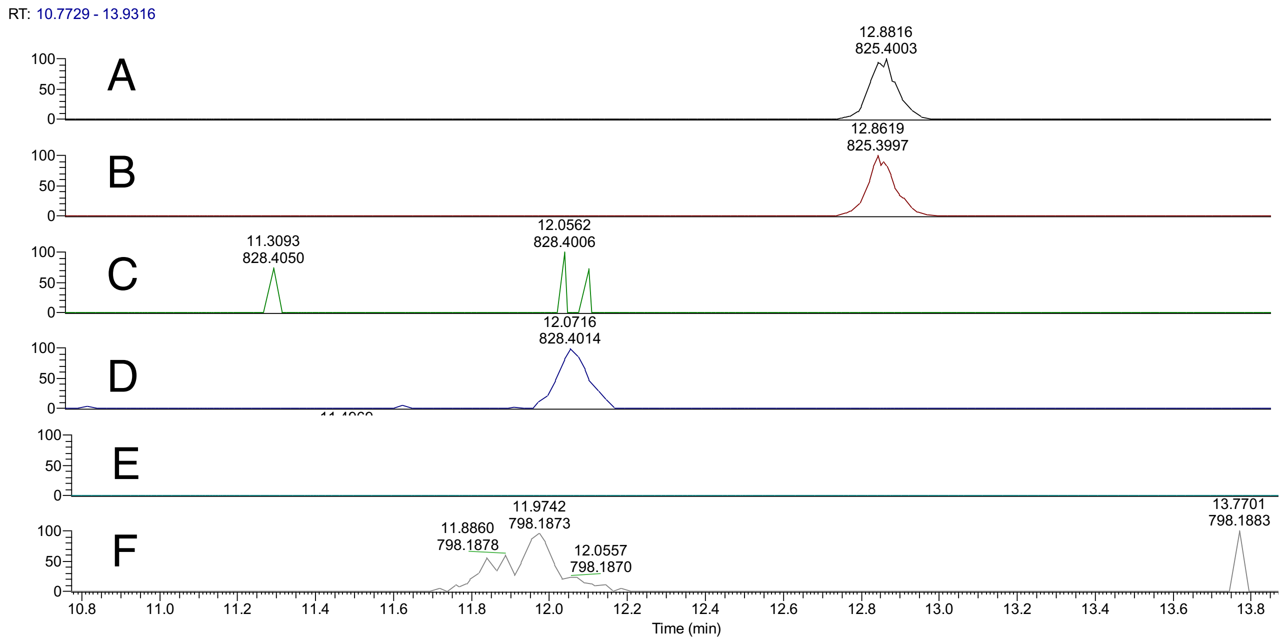

Figure S47: Summary of protein MS results: two modified peptides, derived from 224-LSPNGDTQGCIDSAPETVFVPAAK-248 are only found in the sample irradiated with blue light for 30 min.

A and B: control XIC of +3 charged ion representing peptide 224-248 LSPNGDTQGCIDSAPETVFVPAAK with only C-iodoacetamethyl modification.
A: dark sample, relative abundance of 100 equal 8.2E7.
B: irradiated sample, relative abundance of 100 equal 1.22E8.

C and D: XIC of _3 charged ion representing peptide 224-248 LSPNGDTQGCIDSAPETVFVPAAK modified with unknown +66 Da modification.
C: dark sample, relative abundance of 100 equal 9.08E3 (not found).
D: irradiated sample, relative abundance of 100 equal 4.4E5.

E and F: XIC of +5 charged ion representing peptide 224-248LSPNGDTQGCIDSAPETVFVPAAK with unknown crosslinking or modification.
E: dark sample, relative abundance of 100 equal 0 (no peak found).
F: irradiated sample, relative abundance of 100 equal 1.68E5.

M. Chiral HPLC traces

1-phenyl-2-(tetrahydro-2*H*-pyran-4-yl)pyrrolidine (**2**)

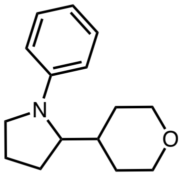

Conditions: ChiralPak® IG, 1% isopropanol in hexanes

Racemic **2**: 50:50 e.r.

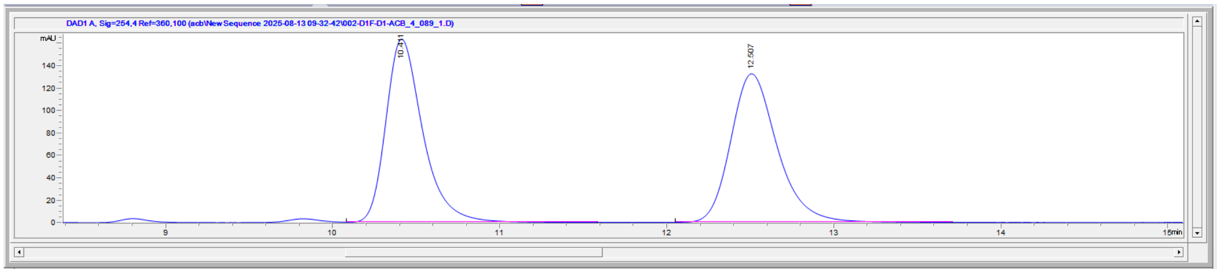

2 from GlowHA: 90:10 e.r.

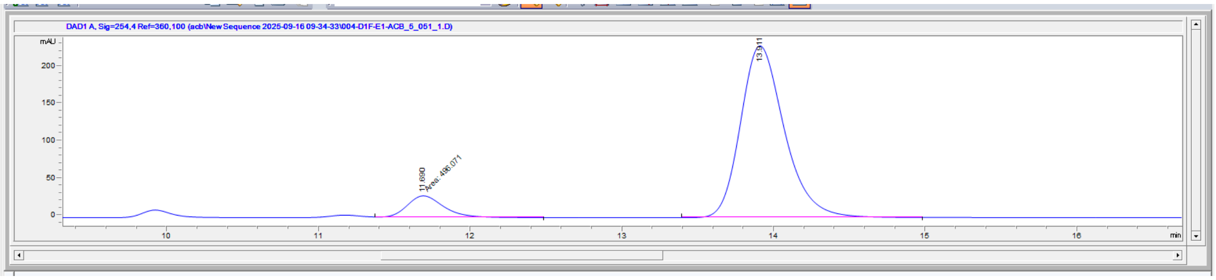

2 from GlowER R5: 64:36 e.r.

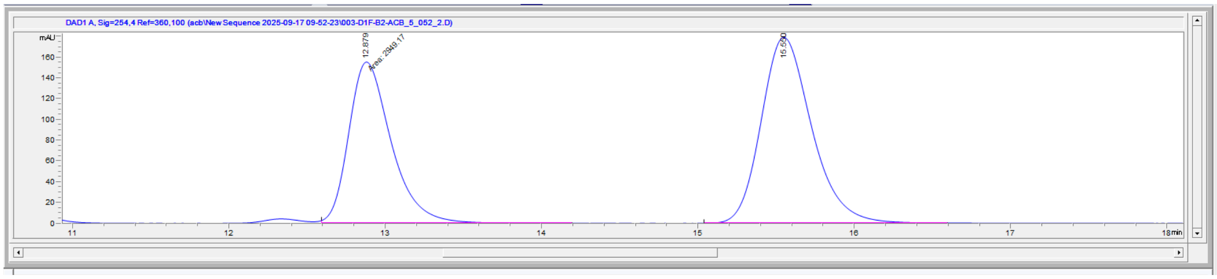

2-(tetrahydro-2*H*-pyran-4-yl)-1-(*p*-tolyl)pyrrolidine (**3**)

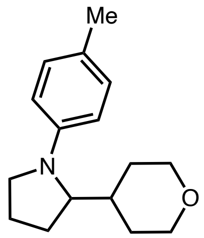

Conditions: ChiralPak® ID, 1% isopropanol in hexanes

Racemic **3**: 50:50 e.r.

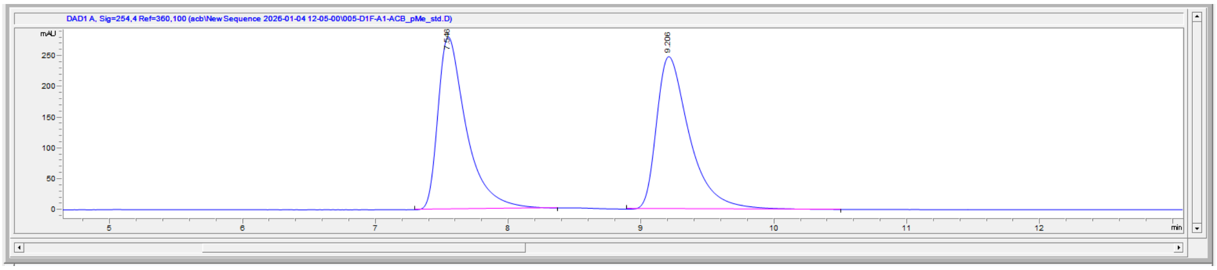

3 from GlowHA: 93:7 e.r.

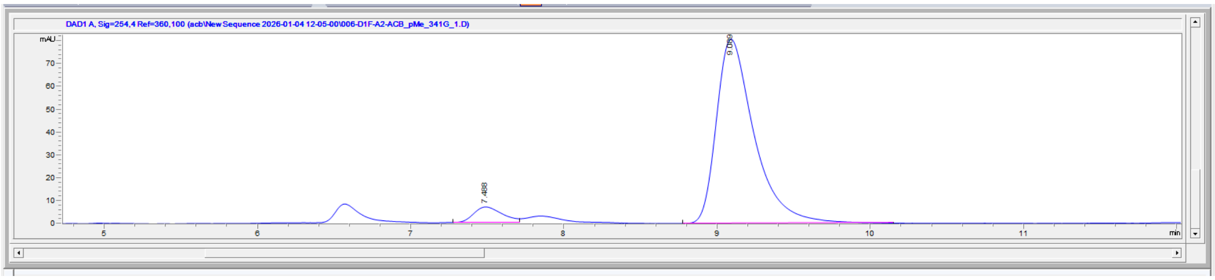

3 from GlowER R5: 67:33 e.r.

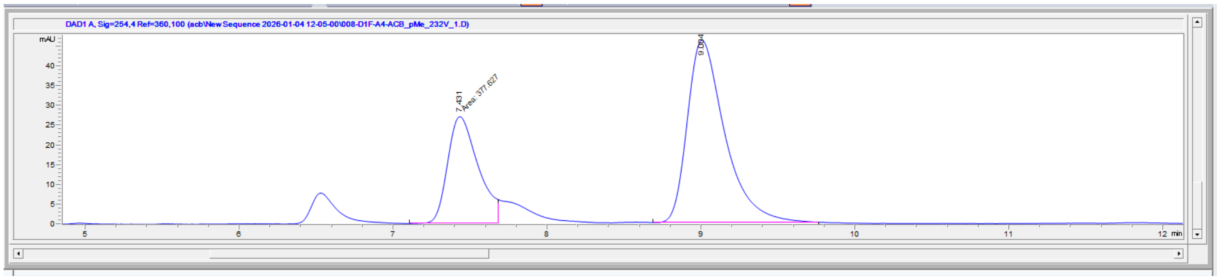

1-(4-chlorophenyl)-2-(tetrahydro-2*H*-pyran-4-yl)pyrrolidine (**4**)

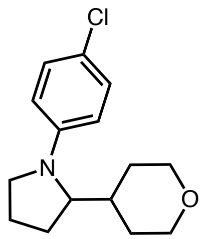

Conditions: ChiralPak® IG, 0.5% isopropanol in hexanes

Racemic **4**: 50:50 e.r.

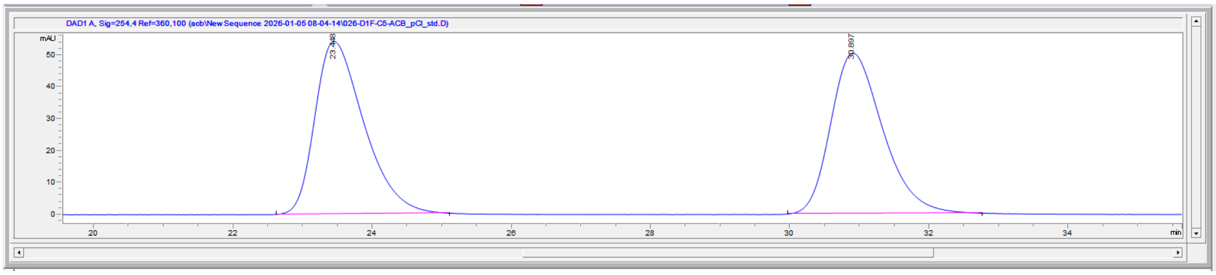

4 from GlowHA: 91:9 e.r.

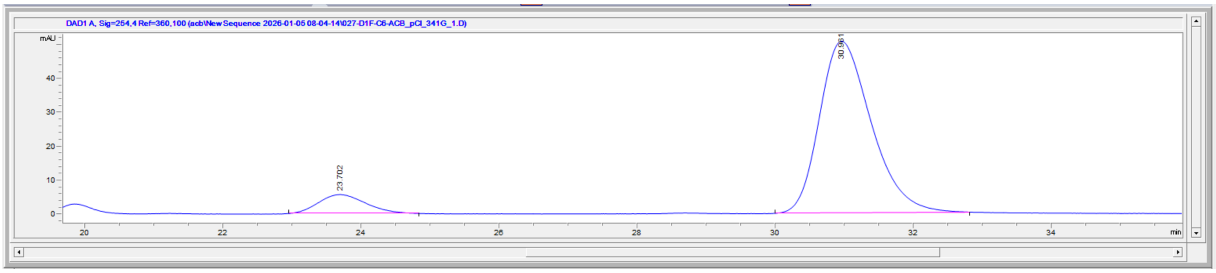

4 from GlowER R5: 60:40 e.r.

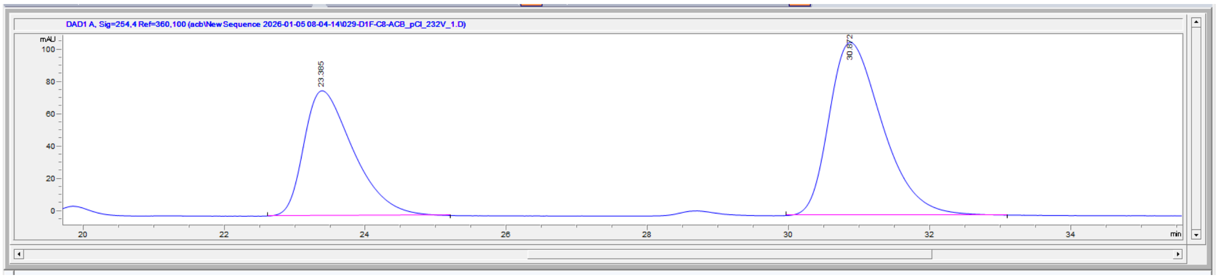

1-(4-bromophenyl)-2-(tetrahydro-2*H*-pyran-4-yl)pyrrolidine (**5**)

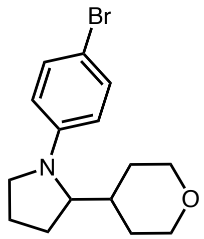

Conditions: ChiralPak® IG, 0.5% isopropanol in hexanes

Racemic **5**: 50:50 e.r.

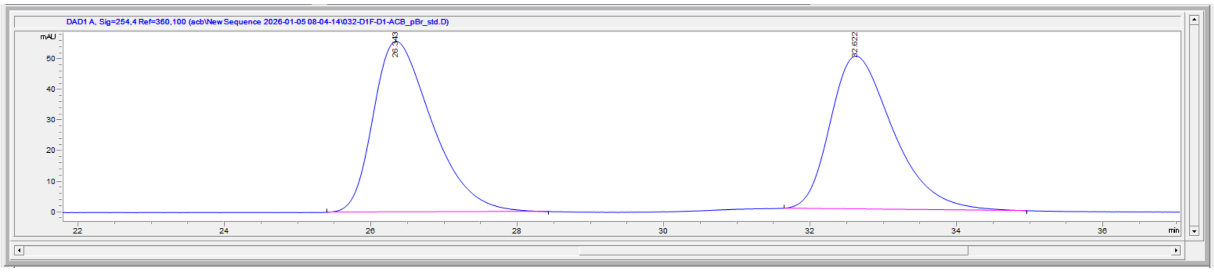

5 from GlowHA: 89:11 e.r.

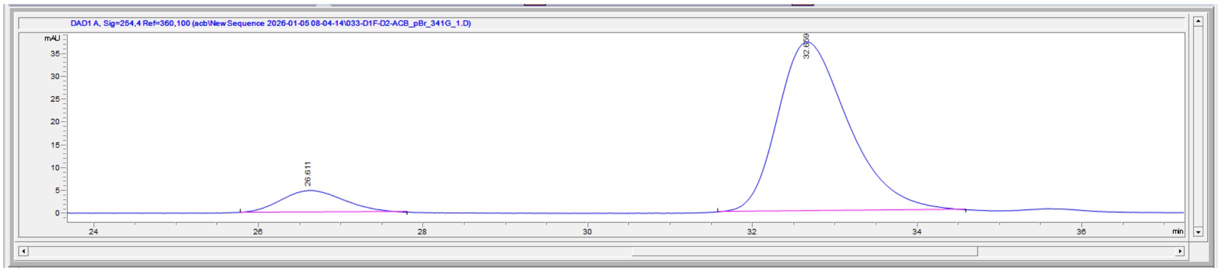

5 from GlowER R5: 58:42 e.r.

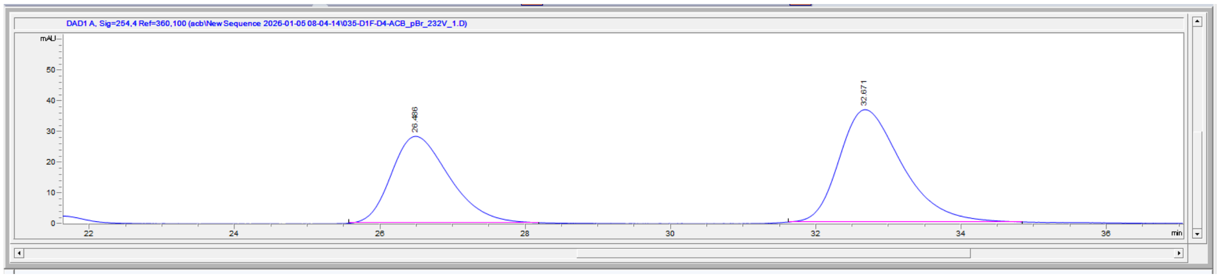

2-(tetrahydro-2*H*-pyran-4-yl)-1-(4-(trifluoromethyl)phenyl)pyrrolidine (**6**)

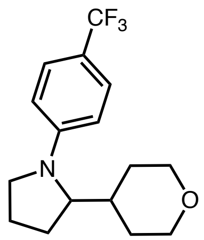

Conditions: ChiralPak® IG, 0.5% isopropanol in hexanes

Racemic **6**: 50:50 e.r.

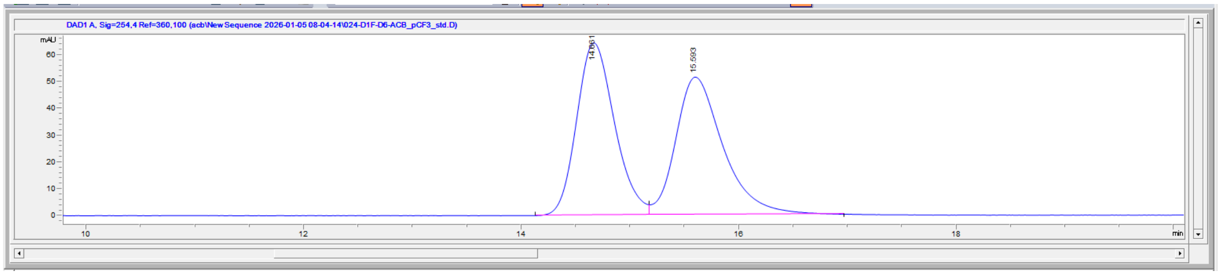

6 from GlowHA: 79:21 e.r.

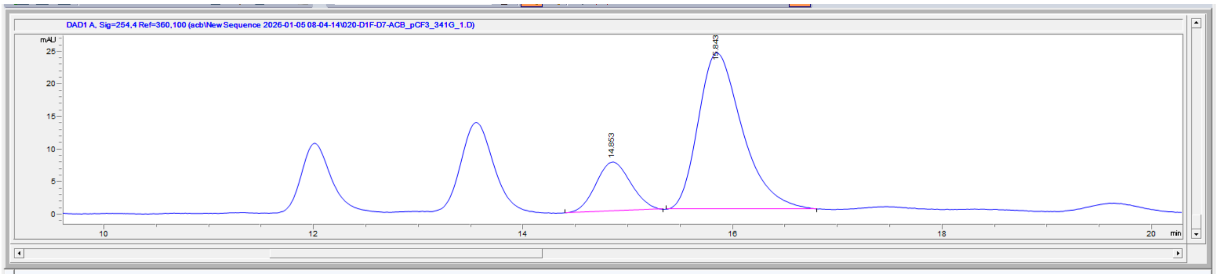

6 from GlowER R5: 53:47 e.r.

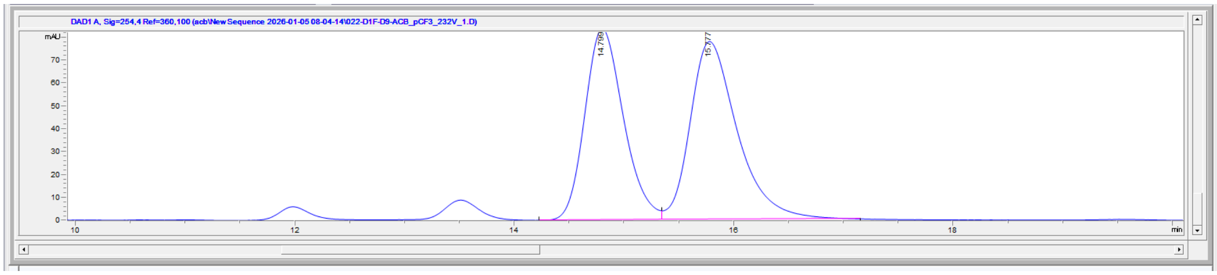

1-(3-methoxyphenyl)-2-(tetrahydro-2*H*-pyran-4-yl)pyrrolidine (**7**)

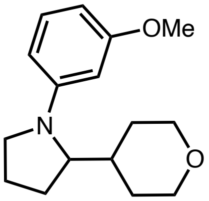

Conditions: ChiralPak® IC, 2.5% isopropanol in hexanes

Racemic **7**: 50:50 e.r.

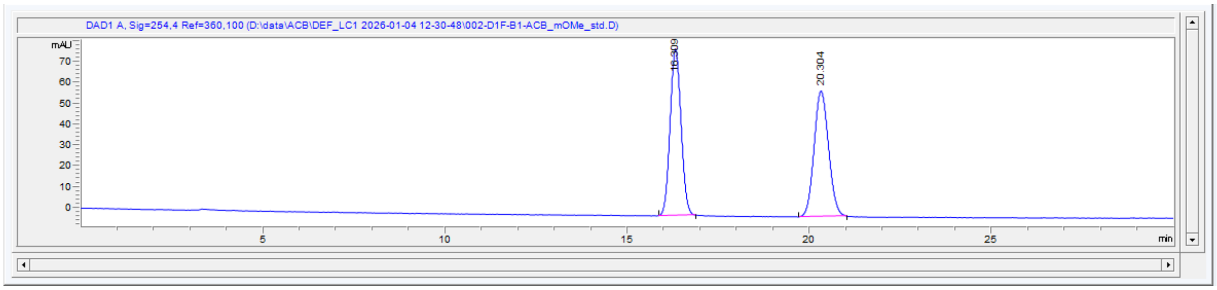

7 from GlowHA: 90:10 e.r.

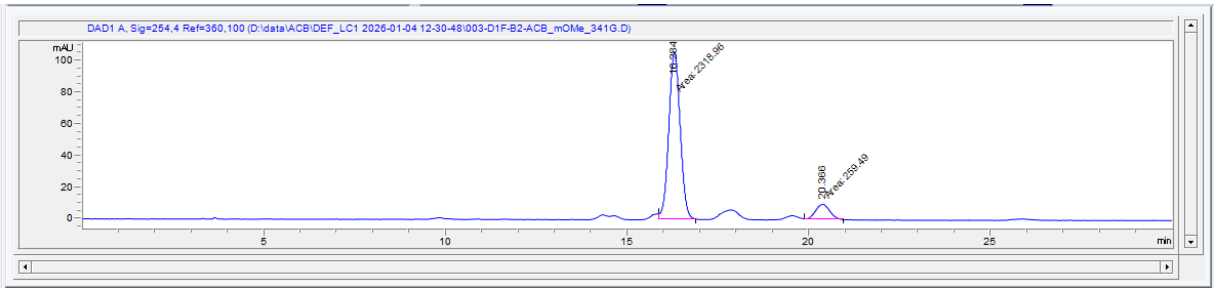

7 from GlowER R5: 63:37 e.r.

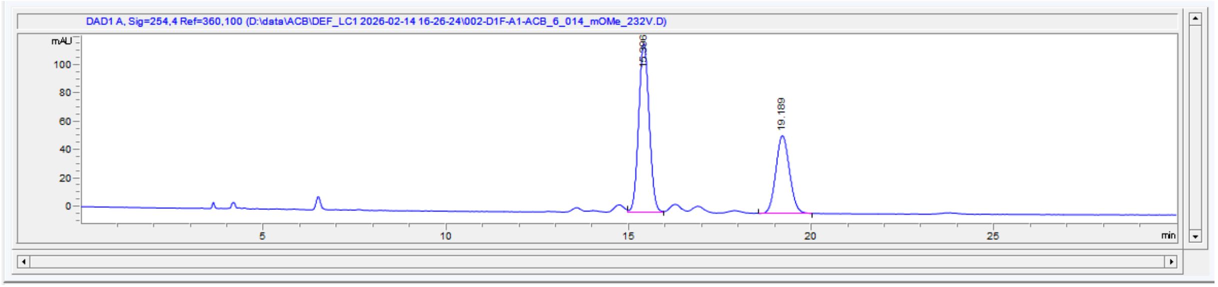

1-(3-bromophenyl)-2-(tetrahydro-2*H*-pyran-4-yl)pyrrolidine (**8**)

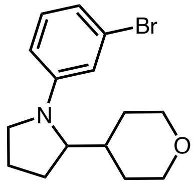

Conditions: ChiralPak® ID, 0.5% isopropanol in hexanes

Racemic **8**: 50:50 e.r.

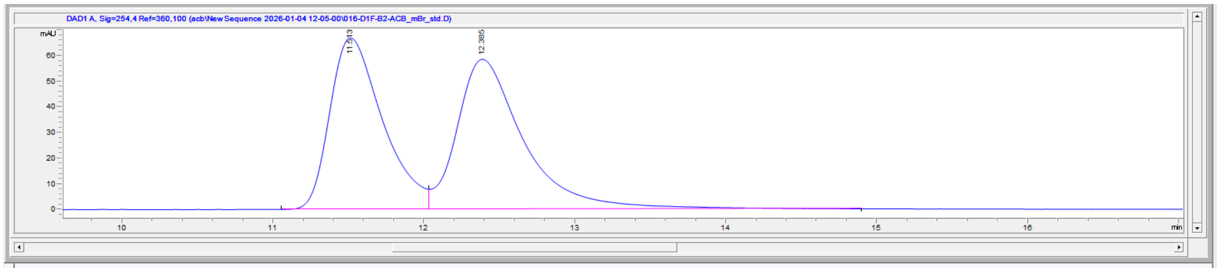

8 from GlowHA: 86:14 e.r.

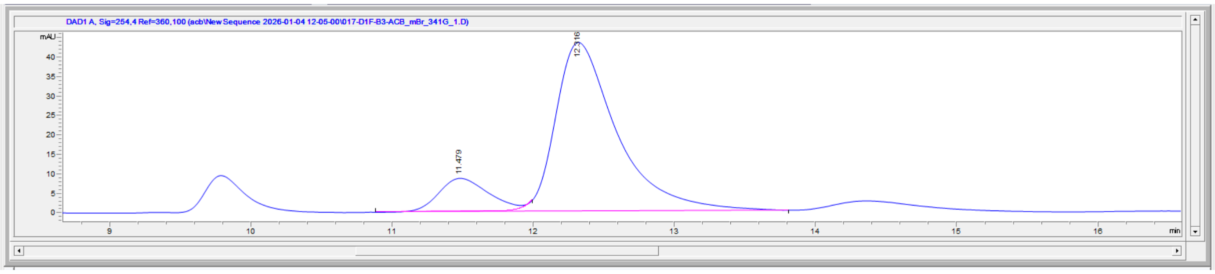

8 from GlowER R5: 63:36 e.r.

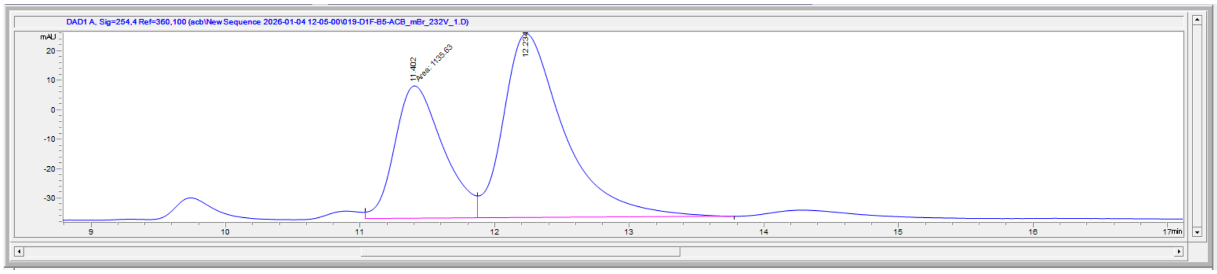

1-(3-fluorophenyl)-2-(tetrahydro-2*H*-pyran-4-yl)pyrrolidine (**9**)

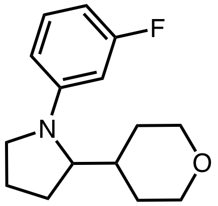

Conditions: ChiralPak® IG, 1% isopropanol in hexanes

Racemic **9**: 50:50 e.r.

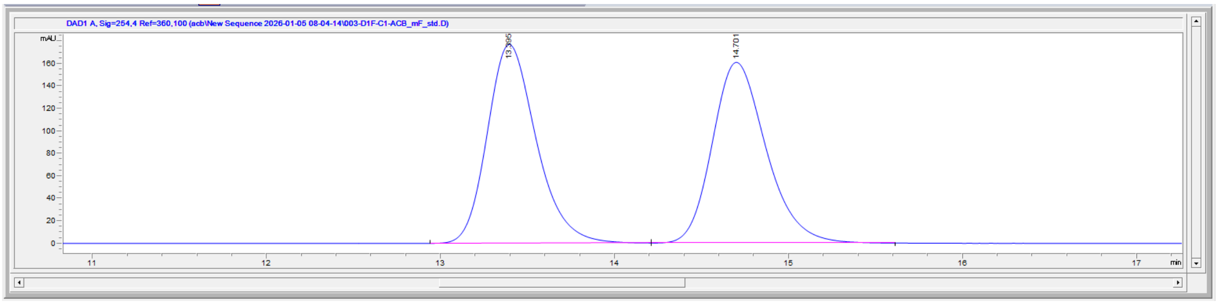

9 from GlowHA: 81:19 e.r.

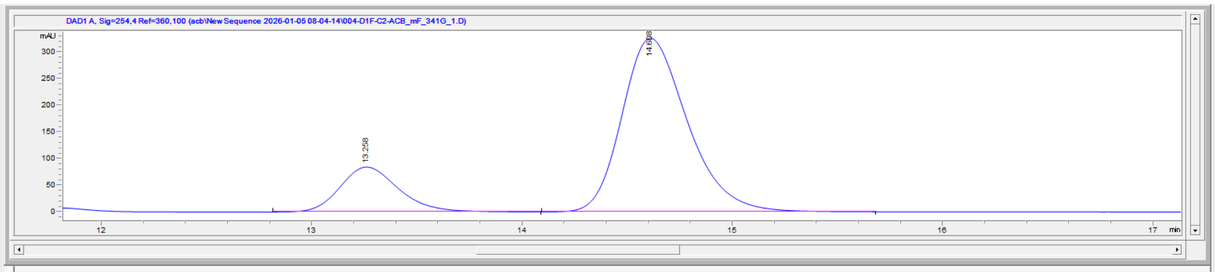

9 from GlowER R5: 55:45 e.r.

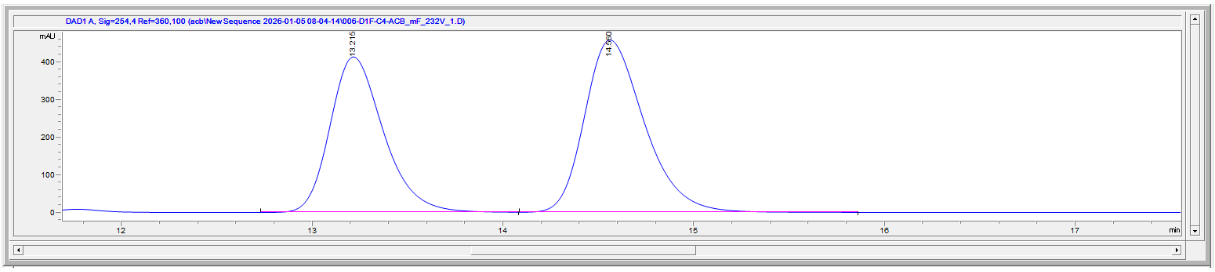

2-(tetrahydro-2*H*-pyran-4-yl)-1-(*m*-tolyl)pyrrolidine (**10**)

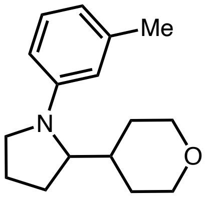

Conditions: ChiralPak® ID, 1% isopropanol in hexanes

Racemic **10**: 50:50 e.r.

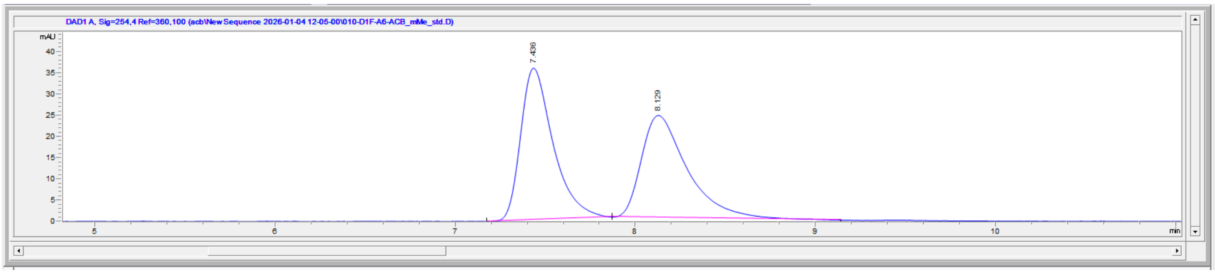

10 from GlowHA: 90:10 e.r.

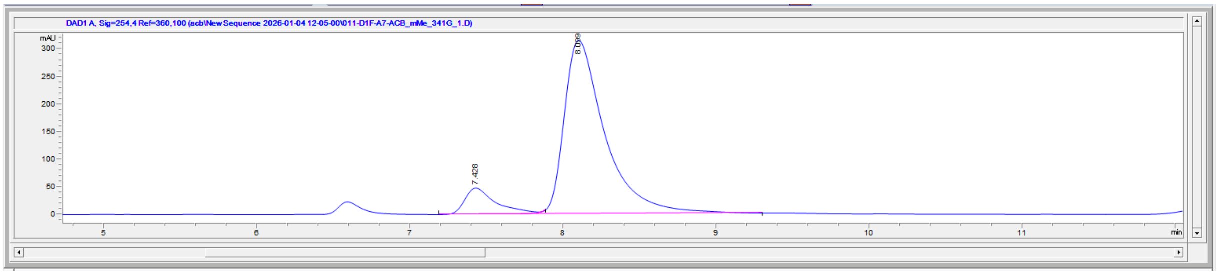

10 from GlowER R5: 64:36 e.r.

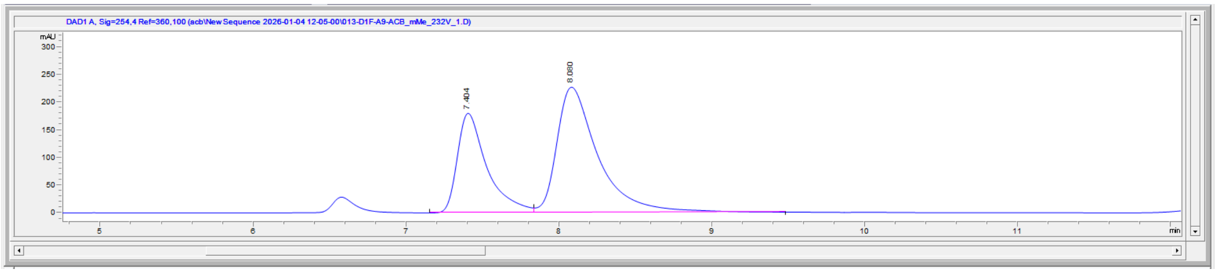

4-(2-(tetrahydro-2*H*-pyran-4-yl)pyrrolidin-1-yl)pyridine (**11**)

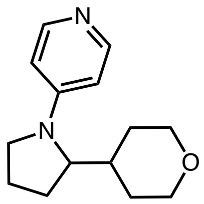

Conditions: Chiral SFC CHIRALPAK IC 2.0 mL/min 20% IPA (0.1% diethylamine)

Racemic **11**: 50:50 e.r.

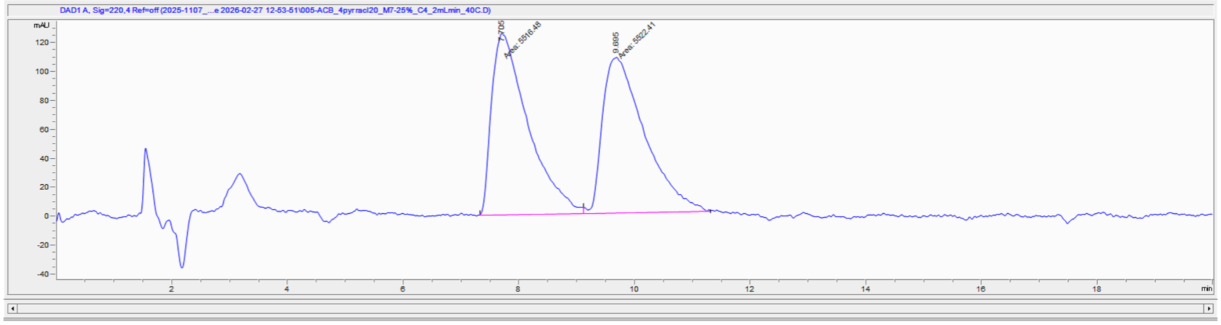

11 from GlowHA: 76:24 e.r.

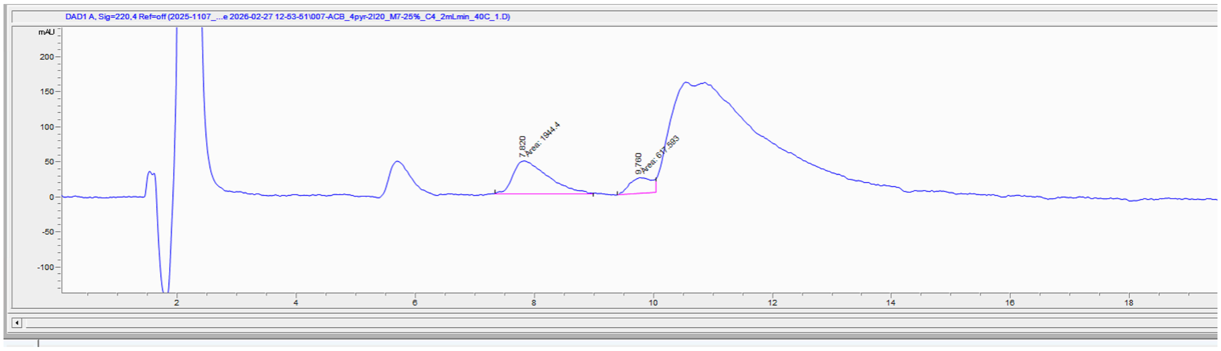

11 from GlowER R5: 71:29 e.r.

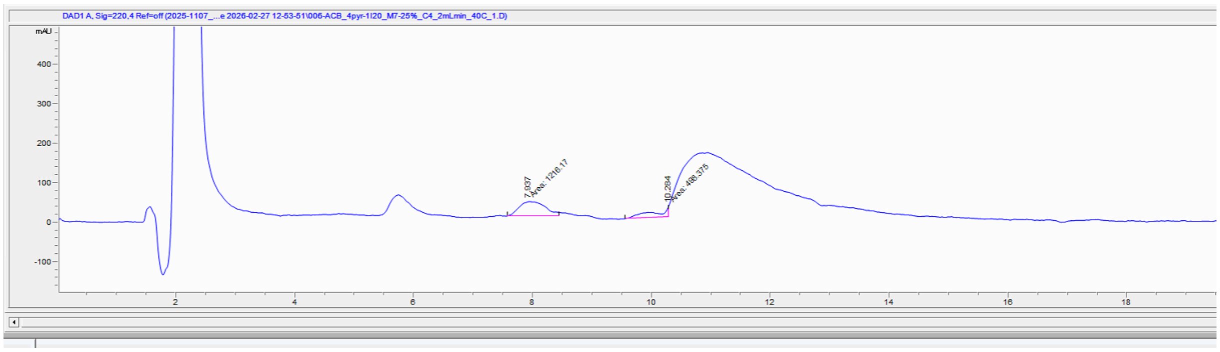

2-(2-(tetrahydro-2*H*-pyran-4-yl)pyrrolidin-1-yl)pyridine (**12**)

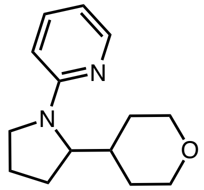

Conditions: ChiralPak® ID, 10% isopropanol in hexanes

Racemic **12**: 50:50 e.r.

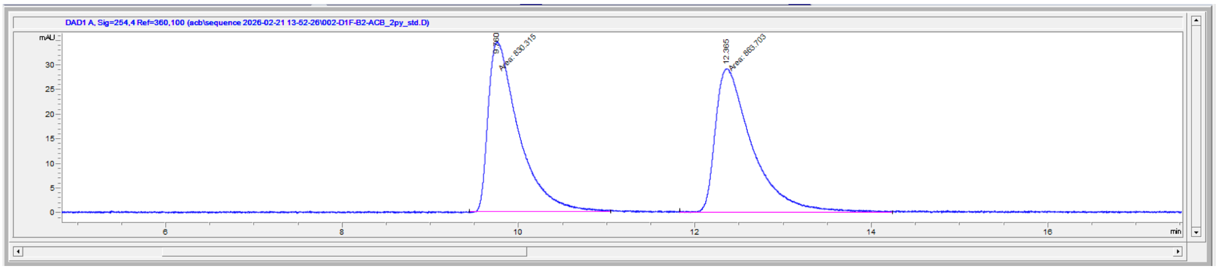

12 from GlowHA: 91:9 e.r.

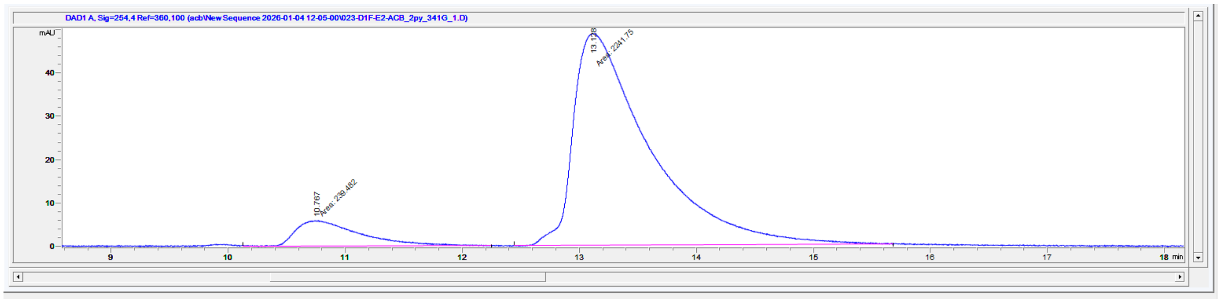

12 from GlowER R5: 61:39 e.r.

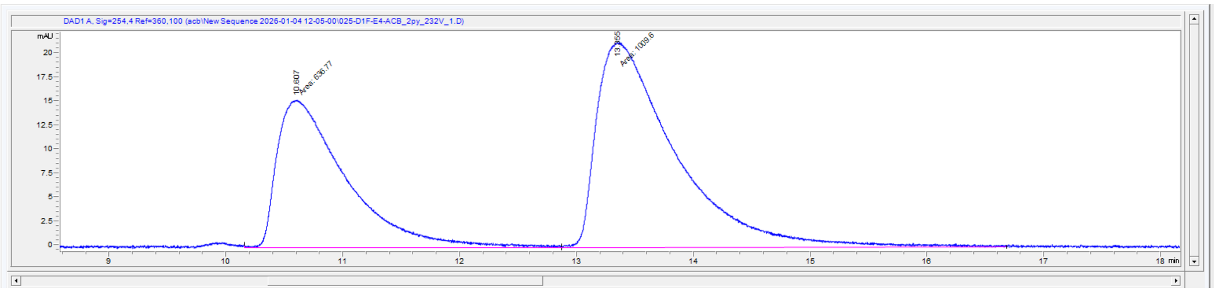

2-cyclohexyl-1-phenylpyrrolidine (**13**)

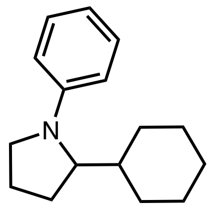

Conditions: ChiralPak® OJ-H, 0.5% isopropanol in hexanes

Racemic **13**: 50:50 e.r.

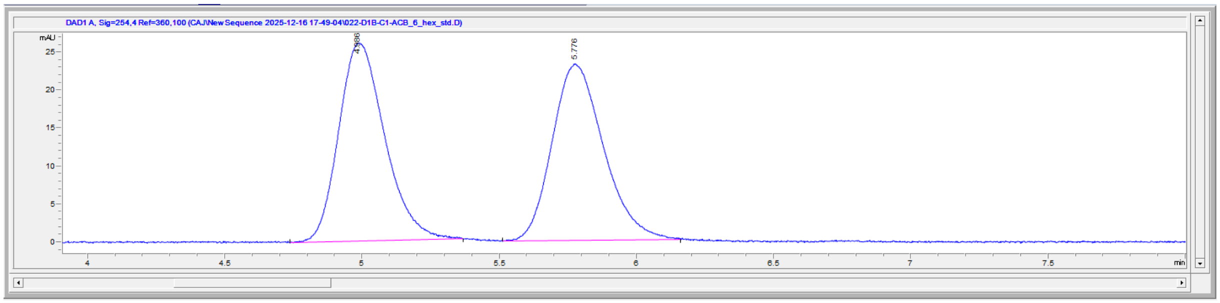

13 from GlowHA: 63:37 e.r.

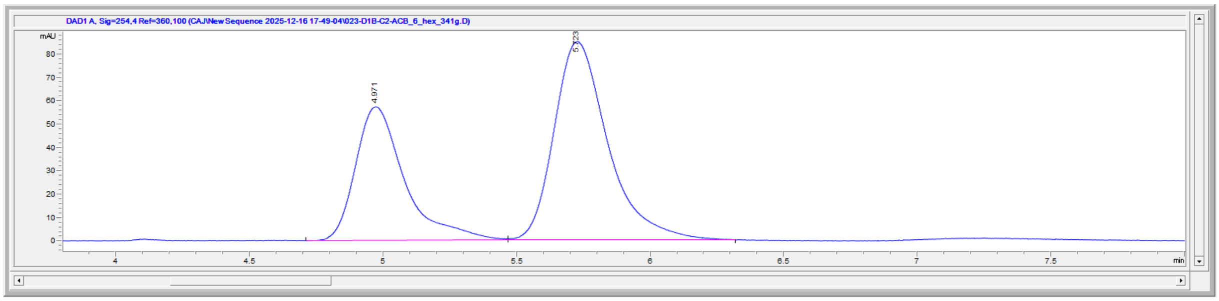

13 from GlowER R5: 50:50 e.r.

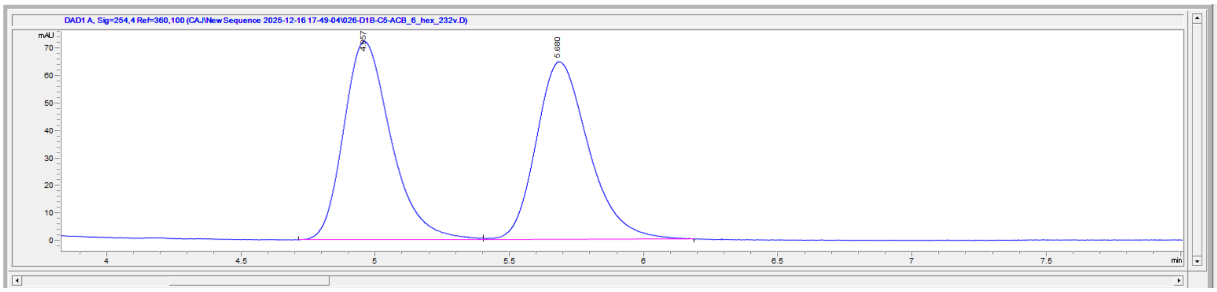

4-(1-phenylpyrrolidin-2-yl)piperidine (**14**)

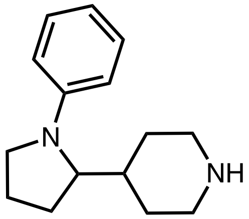

Conditions: ChiralPak® IG, 1% isopropanol in hexanes following derivatization with Boc_2O (20 eq. Boc_2O and 20 equiv. NEt_3 were added to a solution of the enzymatic reaction mixture extracted into DCM. The reaction was stirred for 16 h, evaporated to dryness, redissolved in hexanes and subjected to chiral analysis).

Racemic **16**: 50:50 e.r.

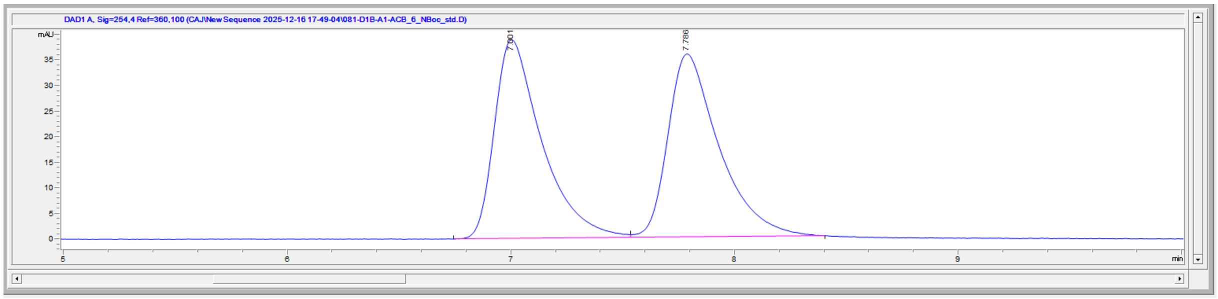

14 + Boc from GlowHA: 90:10 e.r.

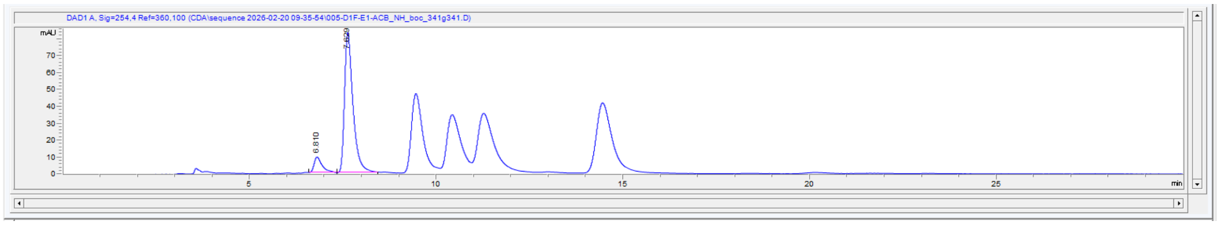

14 + Boc from GlowER R5: 93:7 e.r.

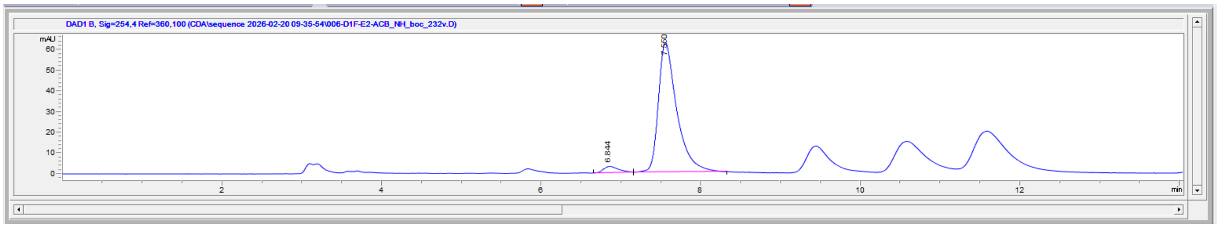

1-methyl-4-(1-phenylpyrrolidin-2-yl)piperidine (**15**)

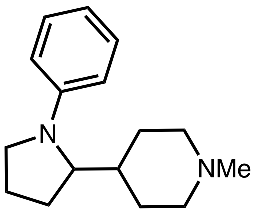

Conditions: Chiral SFC CHIRALPAK IC 2.0 mL/min 20% IPA (0.1% diethylamine)

Racemic **15**: 50:50 e.r.

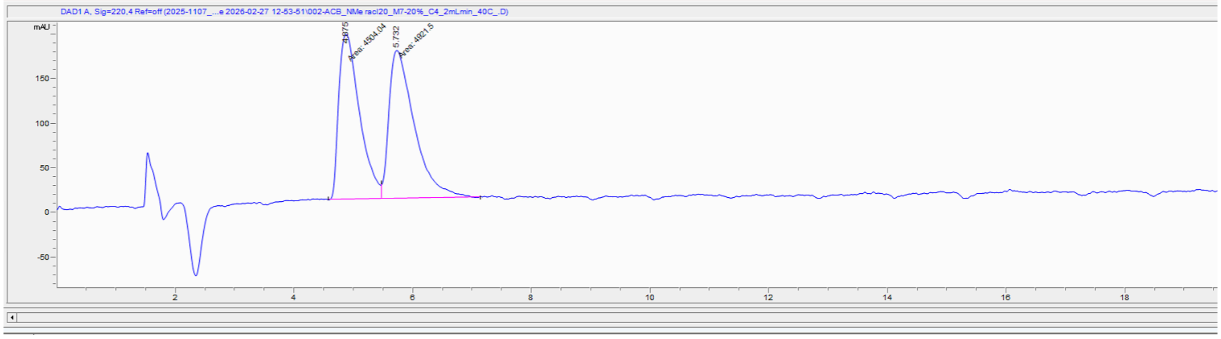

15 from GlowHA: 65:35 e.r.

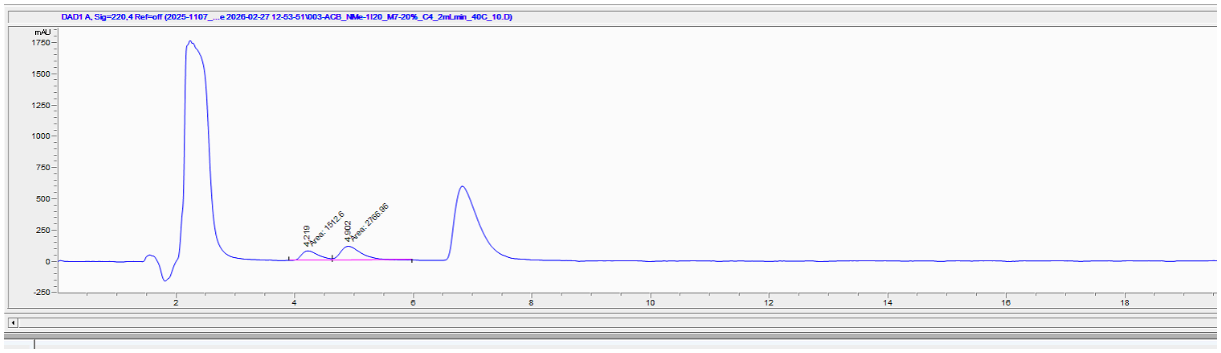

15 from GlowER R5: 64:36 e.r.

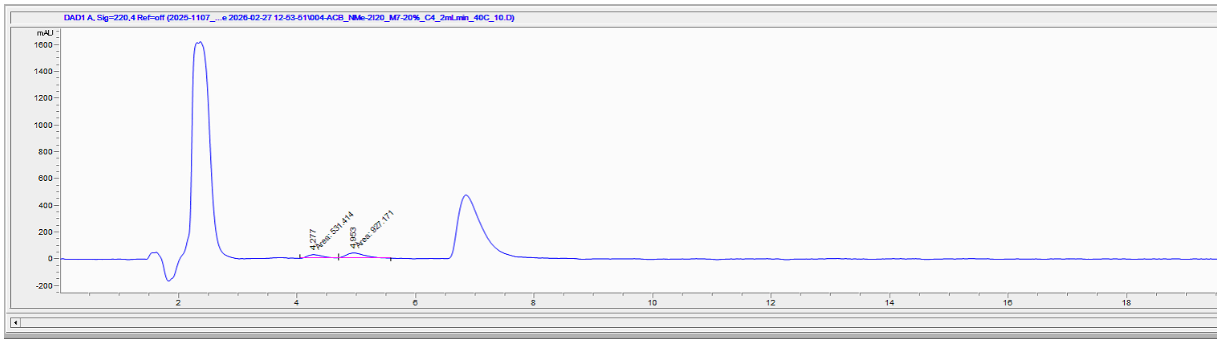

tert-butyl 4-(1-phenylpyrrolidin-2-yl)piperidine-1-carboxylate (**16**)

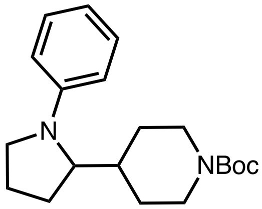

Conditions: ChiralPak® IA, 1% isopropanol in hexanes

Racemic **16**: 50:50 e.r.

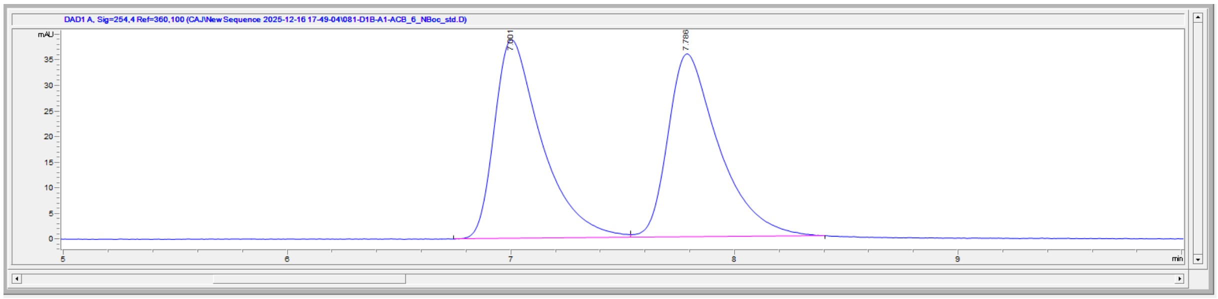

16 from GlowHA: 67:33 e.r.

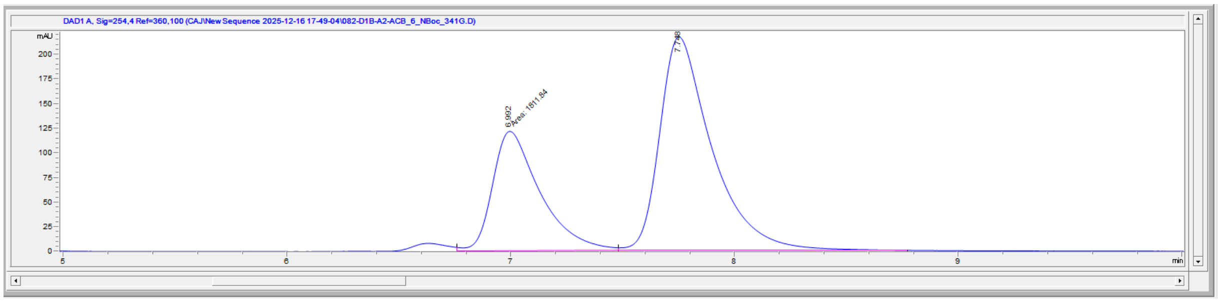

16 from GlowER R5: 55:45 e.r.

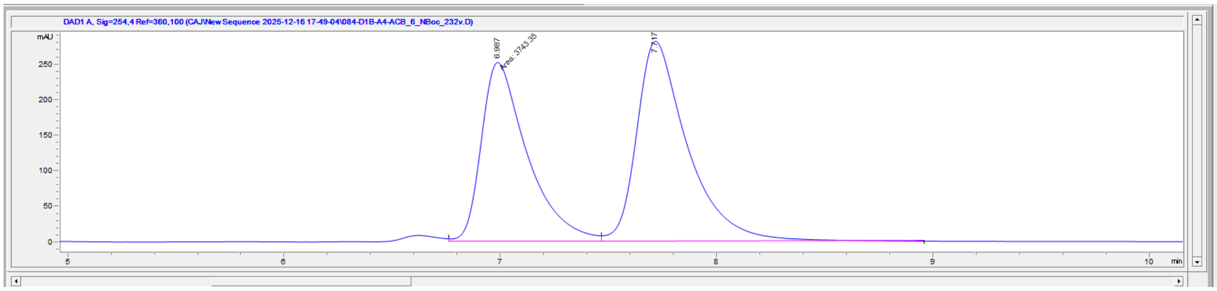

2-(oxetan-3-yl)-1-phenylpyrrolidine (**17**)

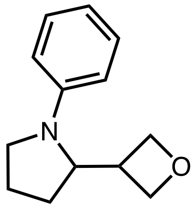

Conditions: ChiralPak® IG, 1% isopropanol in hexanes

Racemic **17**: 50:50 e.r.

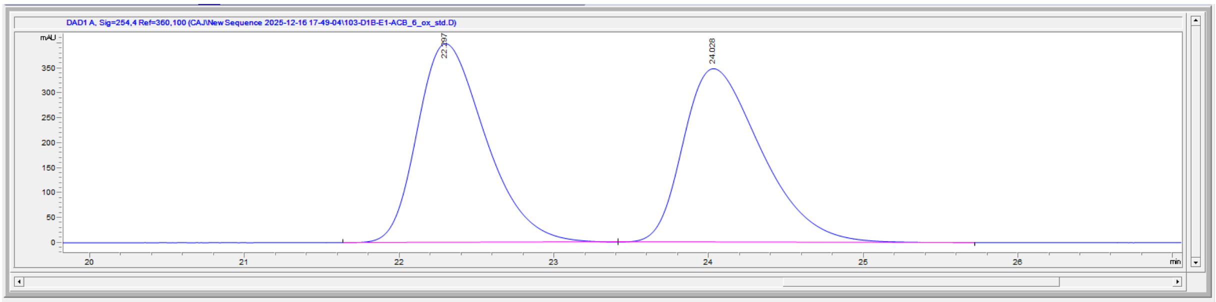

17 from GlowHA: 51:49 e.r.

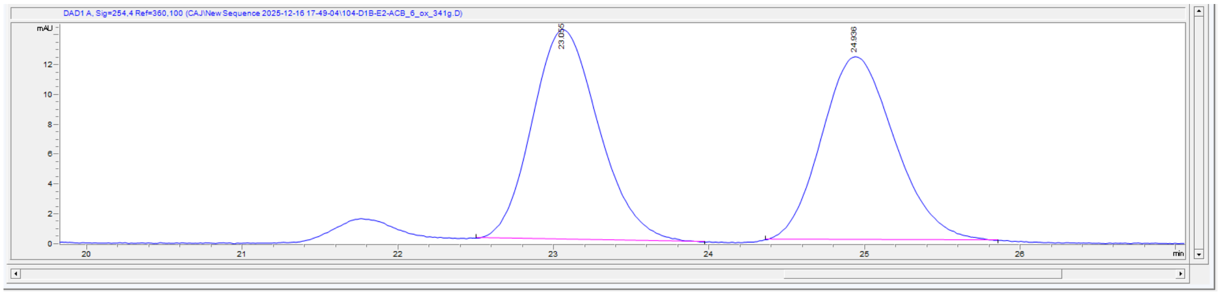

17 from GlowER R5: 51:49 e.r.

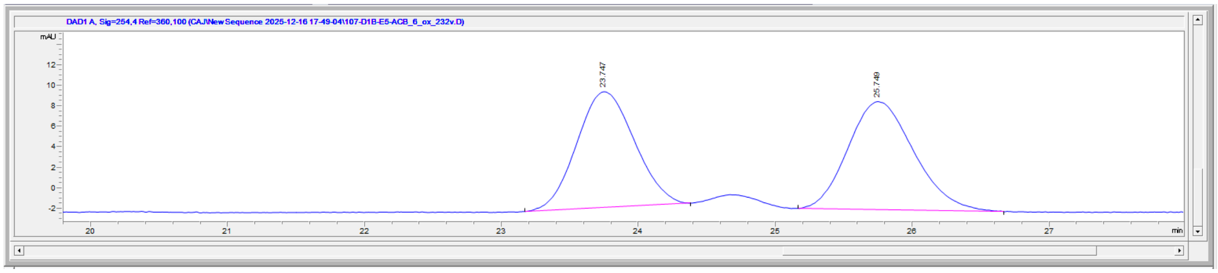

4,4-dimethyl-1-phenyl-2-(tetrahydro-2*H*-pyran-4-yl)pyrrolidine (**18**)

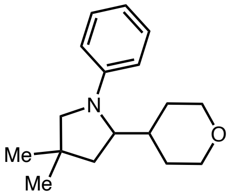

Conditions: ChiralPak® IG, 0.5% isopropanol in hexanes

Racemic **18**: 50:50 e.r.

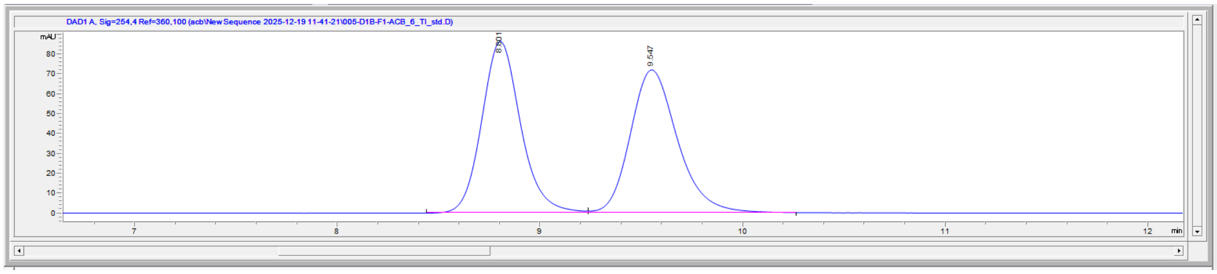

18 from GlowHA: 71:28 e.r.

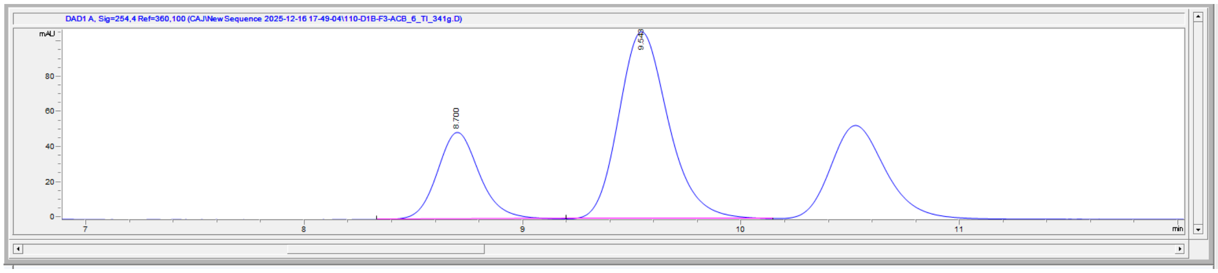

18 from GlowER R5: 56:44 e.r.

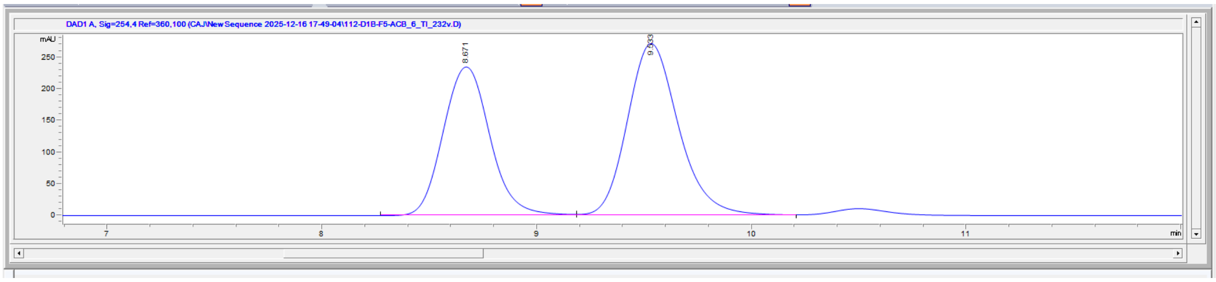

2-isopropyl-1-phenylpyrrolidine (**19**)

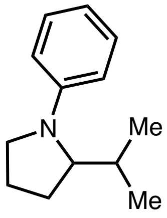

Conditions: ChiralPak® OJ-H 0.5% isopropanol in hexanes

Racemic **19**: 50:50 e.r.

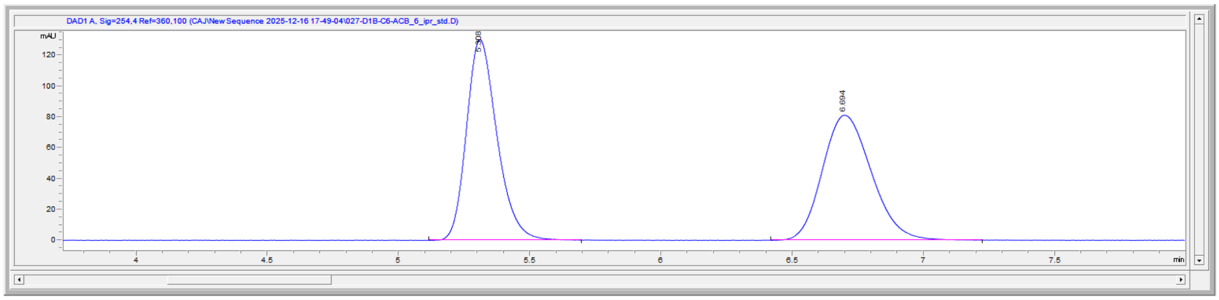

19 from GlowHA: 53:47 e.r.

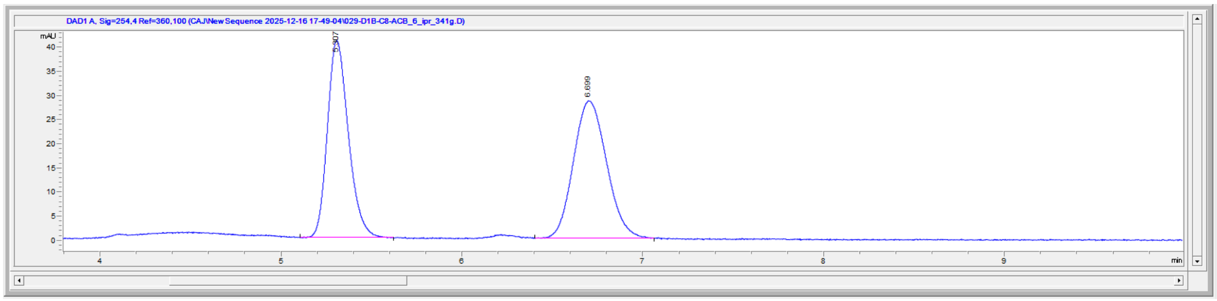

19 from GlowER R5: 52:48 e.r.

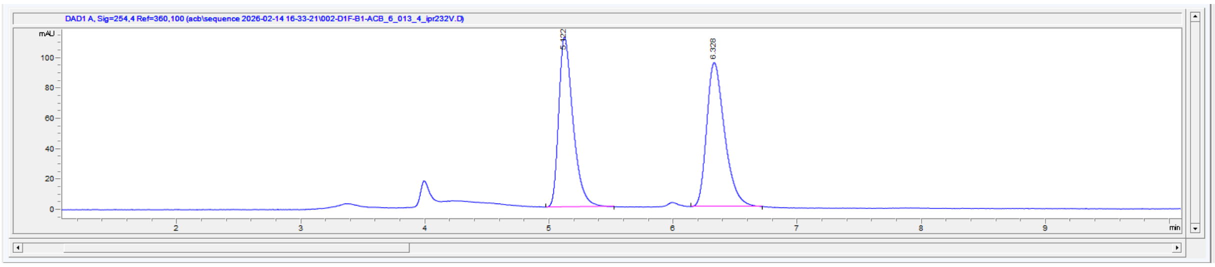

2-ethyl-1-phenylpyrrolidine (**20**)

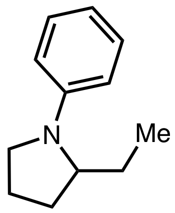

Conditions: ChiralPak® IG, 100% hexanes

Racemic **20**: 50:50 e.r.

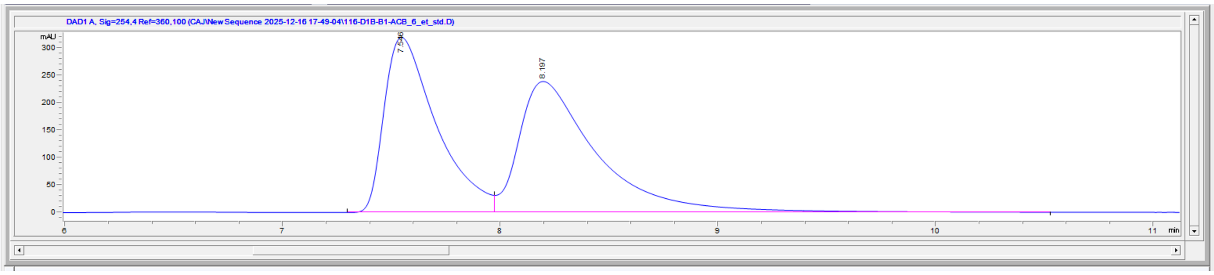

20 from GlowHA: 51:49 e.r.

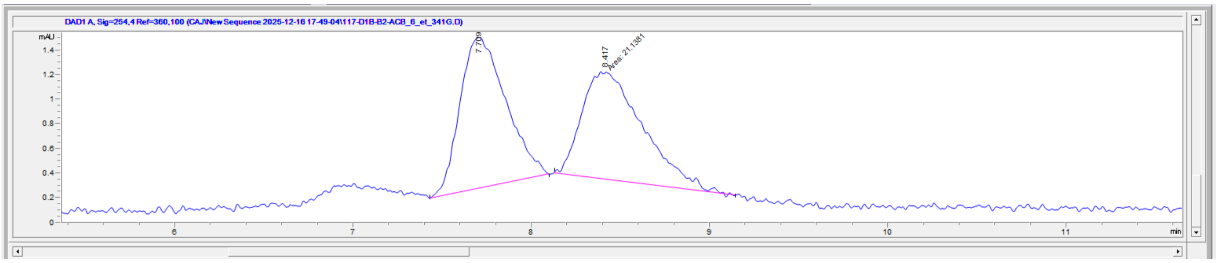

20 from GlowER R5: 56:44 e.r.

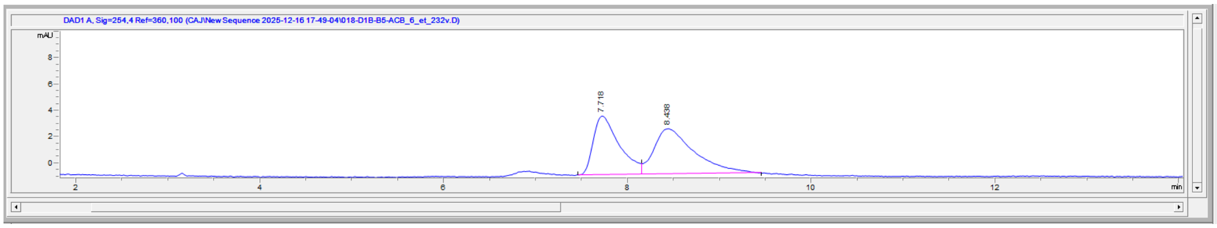

2-benzyl-1-phenylpyrrolidine (**21**)

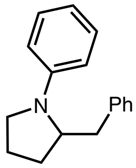

Conditions: ChiralPak® OJ-H, 1% isopropanol in hexanes

Racemic **21**: 50:50 e.r.

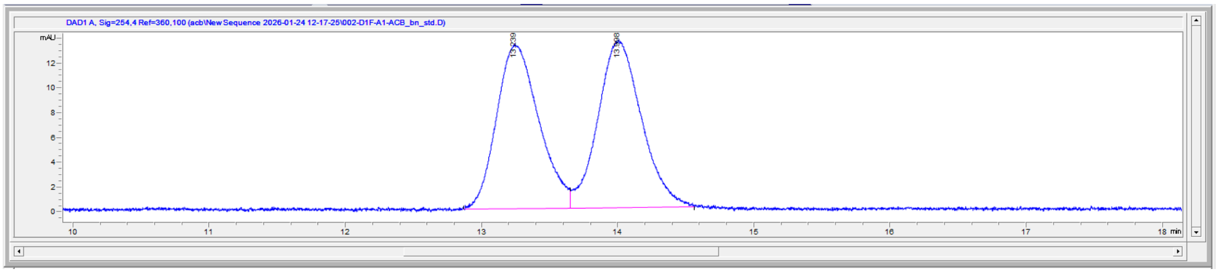

21 from GlowHA: 52:48 e.r.

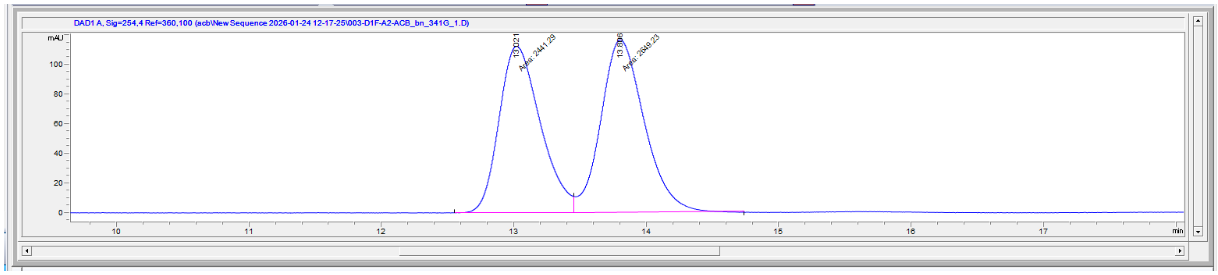

21 from GlowER R5: 50:50 e.r.

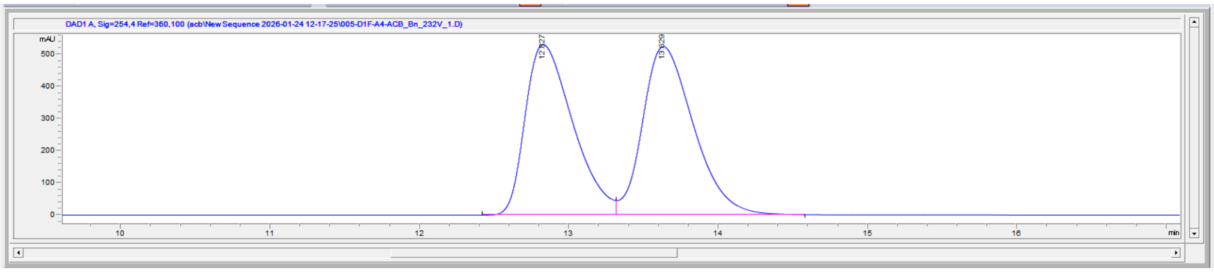

1,3-diphenylpiperidine (**22**)

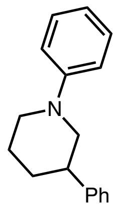

Conditions: ChiralPak® OJ-H, 1% isopropanol in hexanes

Racemic **22**: 50:50 e.r.

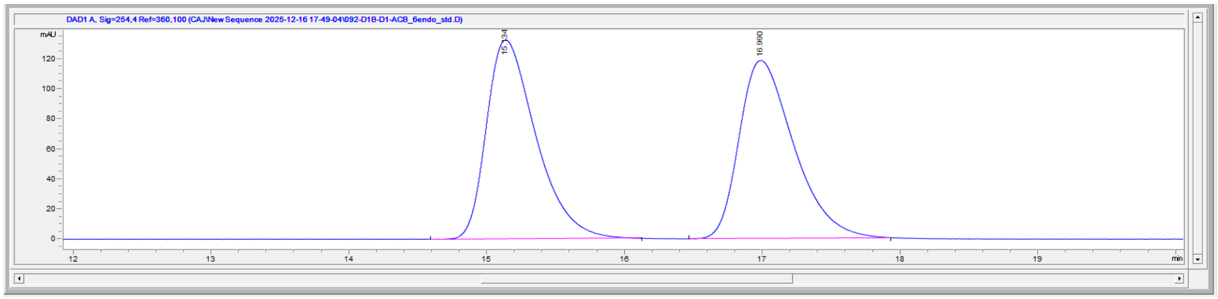

22 from GlowHA: 55:45 e.r.

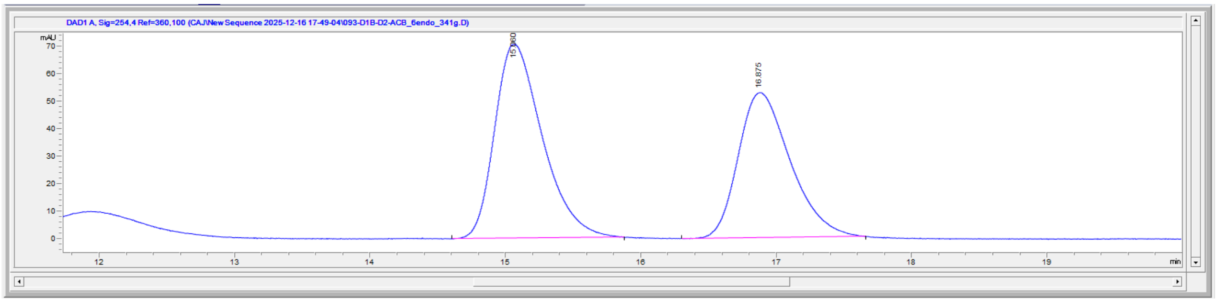

22 from GlowER R5: 63:37 e.r.

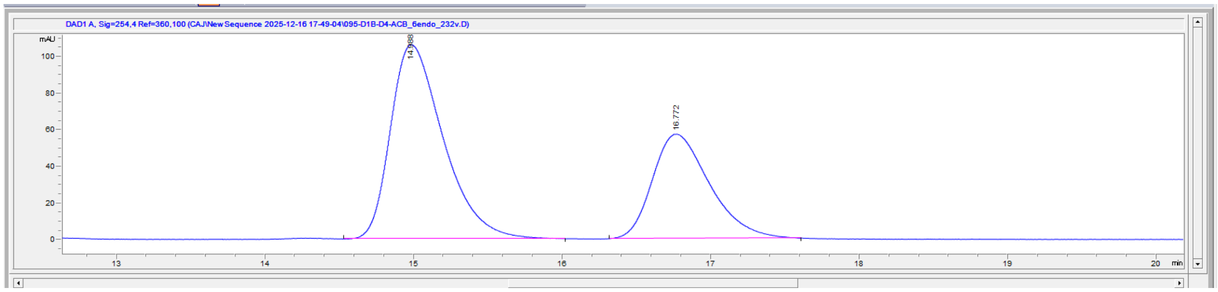

2-(4-methoxybenzyl)-1-phenylpyrrolidine (**23**)

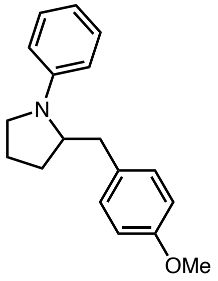

Conditions: ChiralPak® OJ-H, 1% isopropanol in hexanes

Racemic **23**: 50:50 e.r.

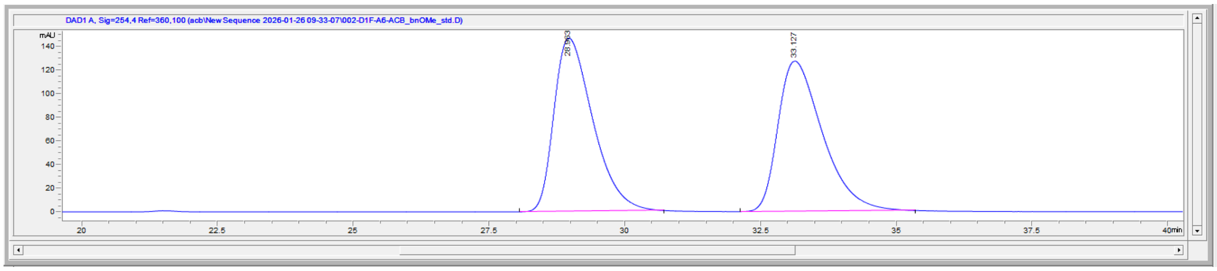

23 from GlowHA: 52:48 e.r.

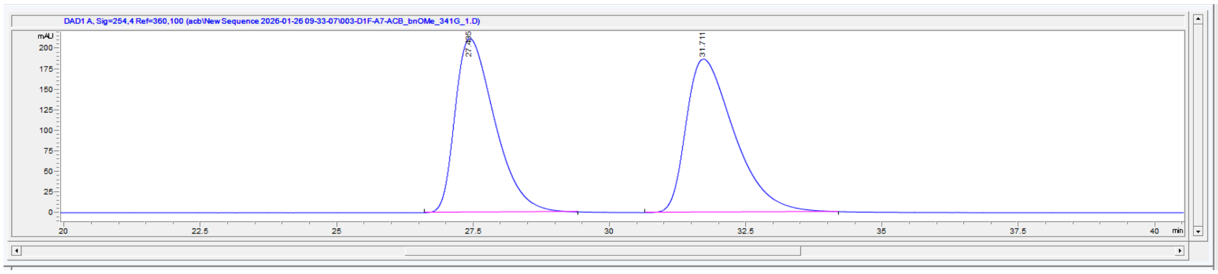

23 from GlowER R5: 52:48 e.r.

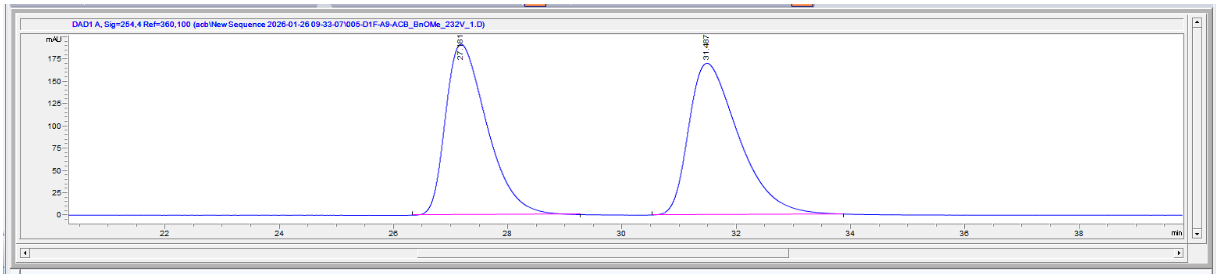

1-phenyl-2-(4-(trifluoromethyl)benzyl)pyrrolidine (**24**)

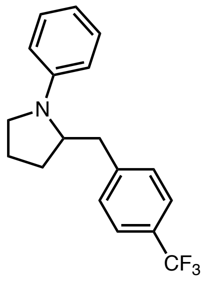

Conditions: ChiralPak® OJ-H, 1% isopropanol in hexanes

Racemic **24**: 50:50 e.r.

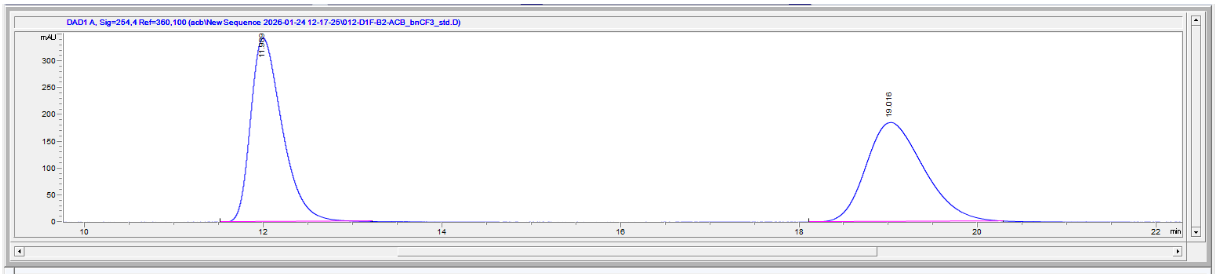

24 from GlowHA: 51:49 e.r.

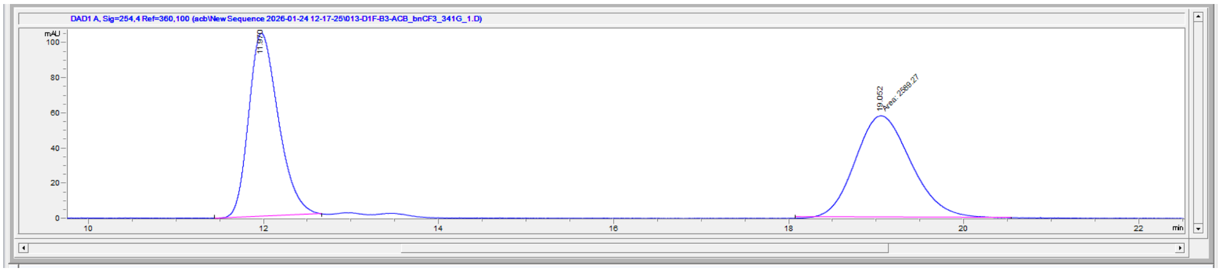

24 from GlowER R5: 50:50 e.r.

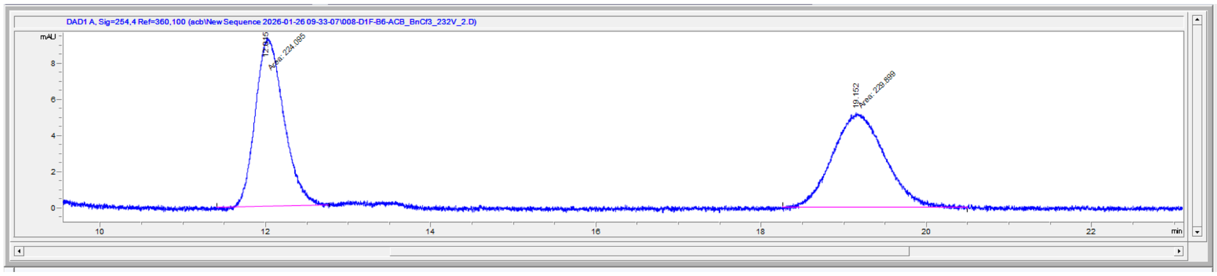

1-phenyl-2-(tetrahydro-2*H*-pyran-3-yl)pyrrolidine (**25**, 1:1.5 mixture of diastereomers)

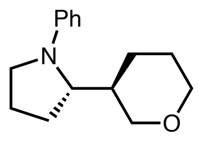

Conditions: ChiralPak® IH, 1% isopropanol in hexanes

Racemic **25**: 50:50, 50:50 e.r.

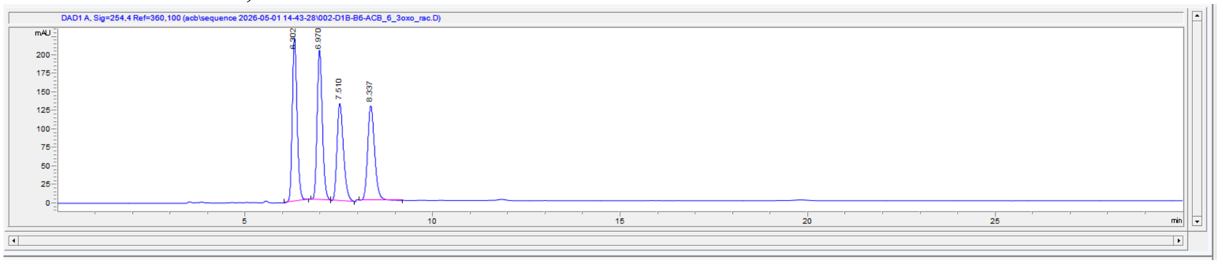

25 from GlowHA: 55:45, 71:29 e.r.

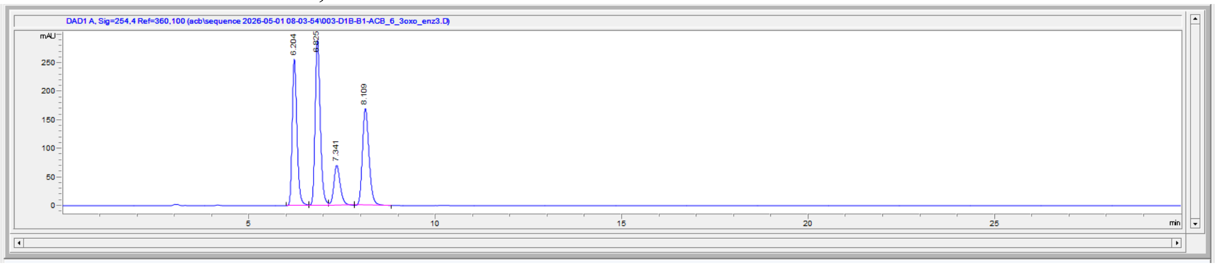

25 from GlowER R5: 60:40, 66:34 e.r.

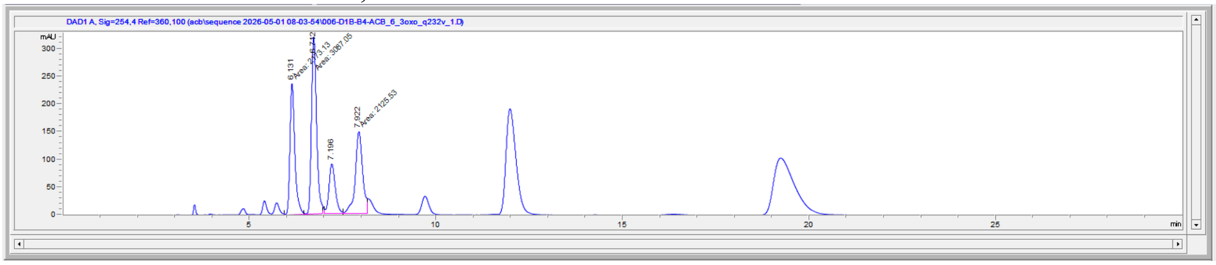

1-phenyl-2-(1-phenylethyl)pyrrolidine (**26**, 1:3.5 cis:trans mixture of diastereomers)

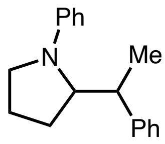

Conditions: ChiralPak® OJ-H, 1% isopropanol in hexanes

Racemic **26**: 50:50, 50:50 e.r.

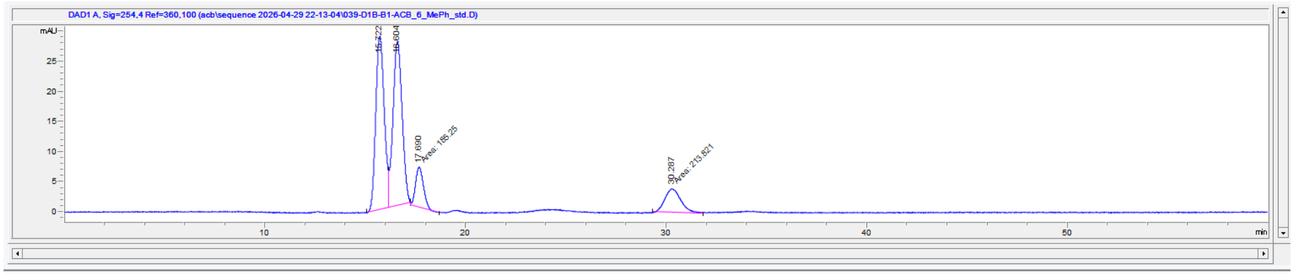

26 from GlowHA: 78:22, 93:7 e.r.

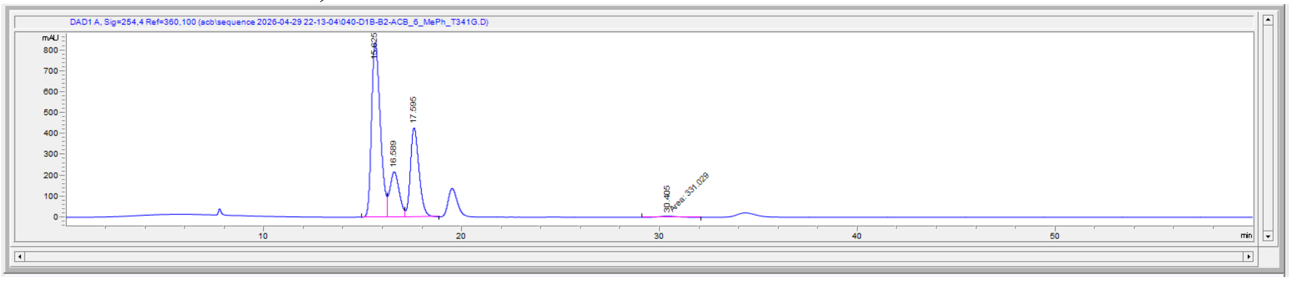

26 from GlowER R5: 66:33, 95:5 e.r.

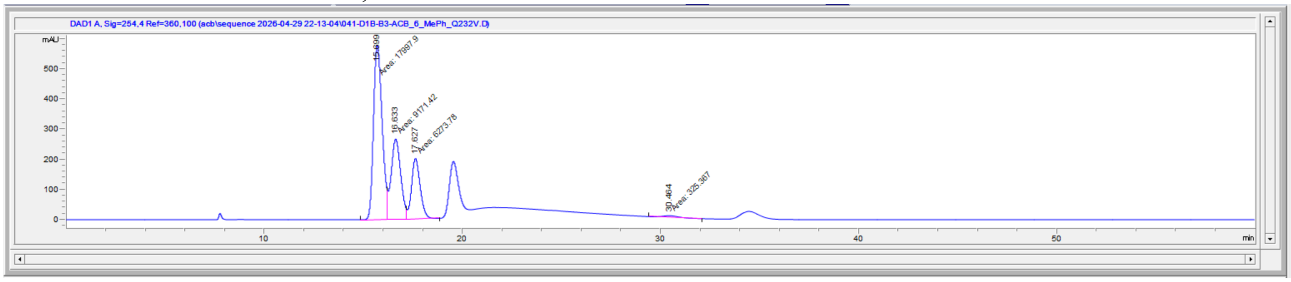

L. NMR spectra

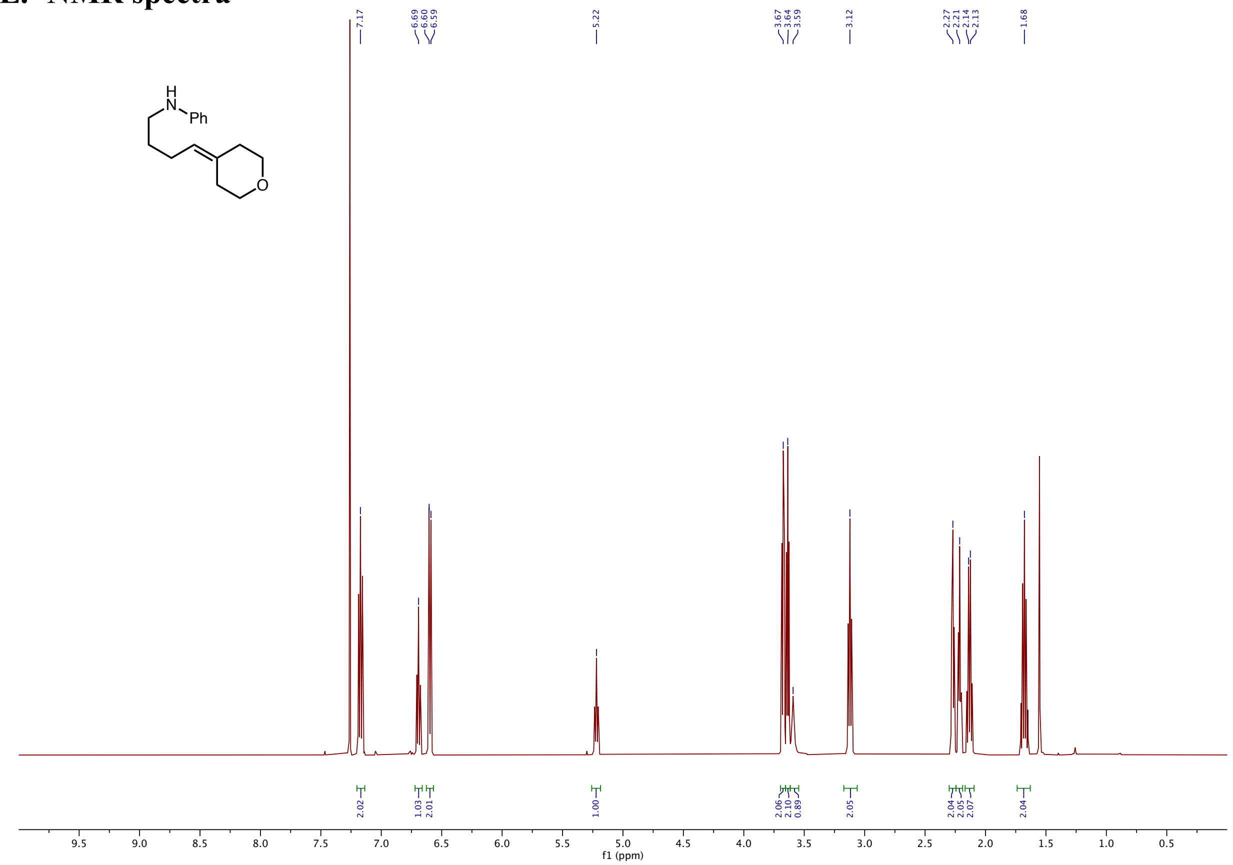

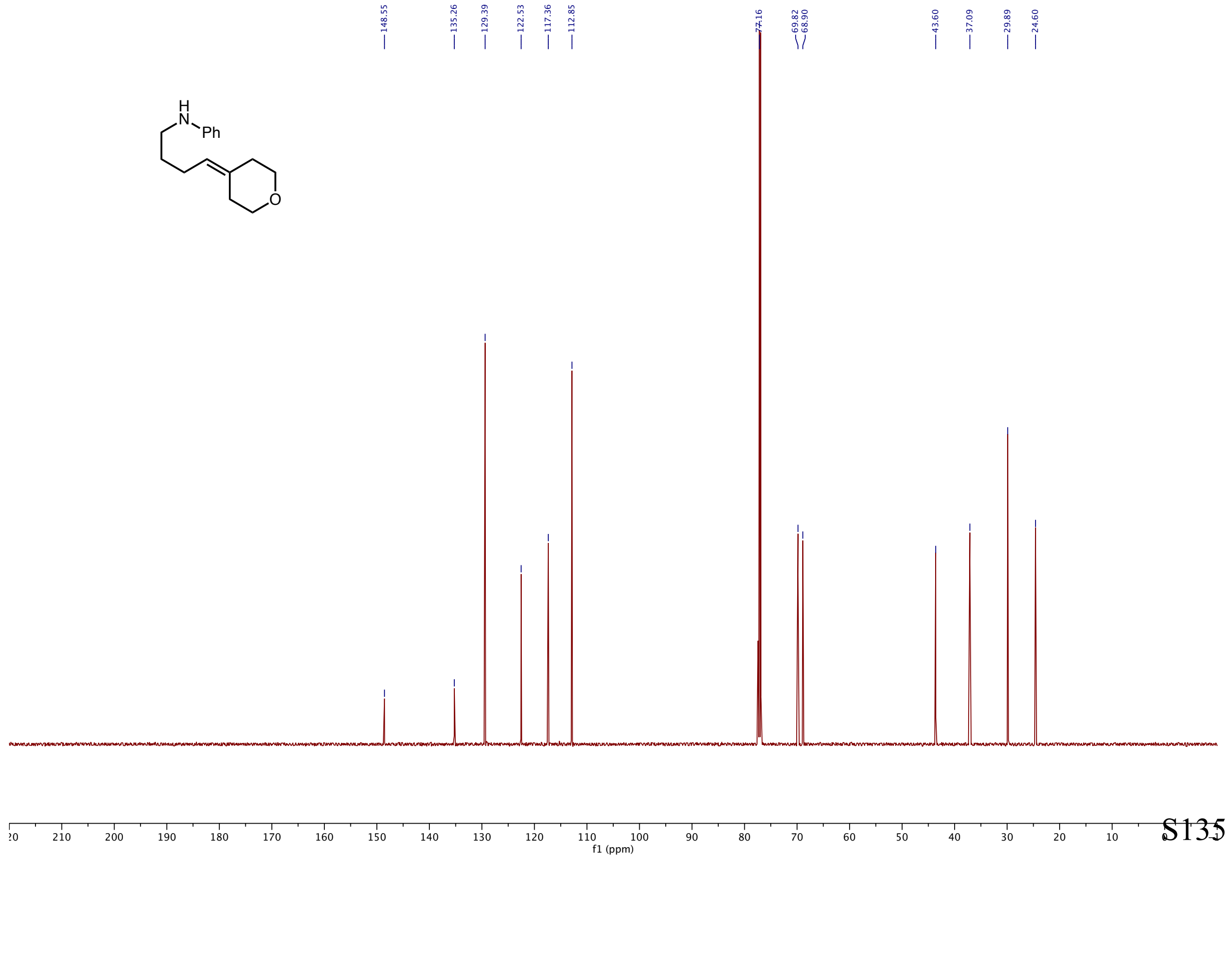

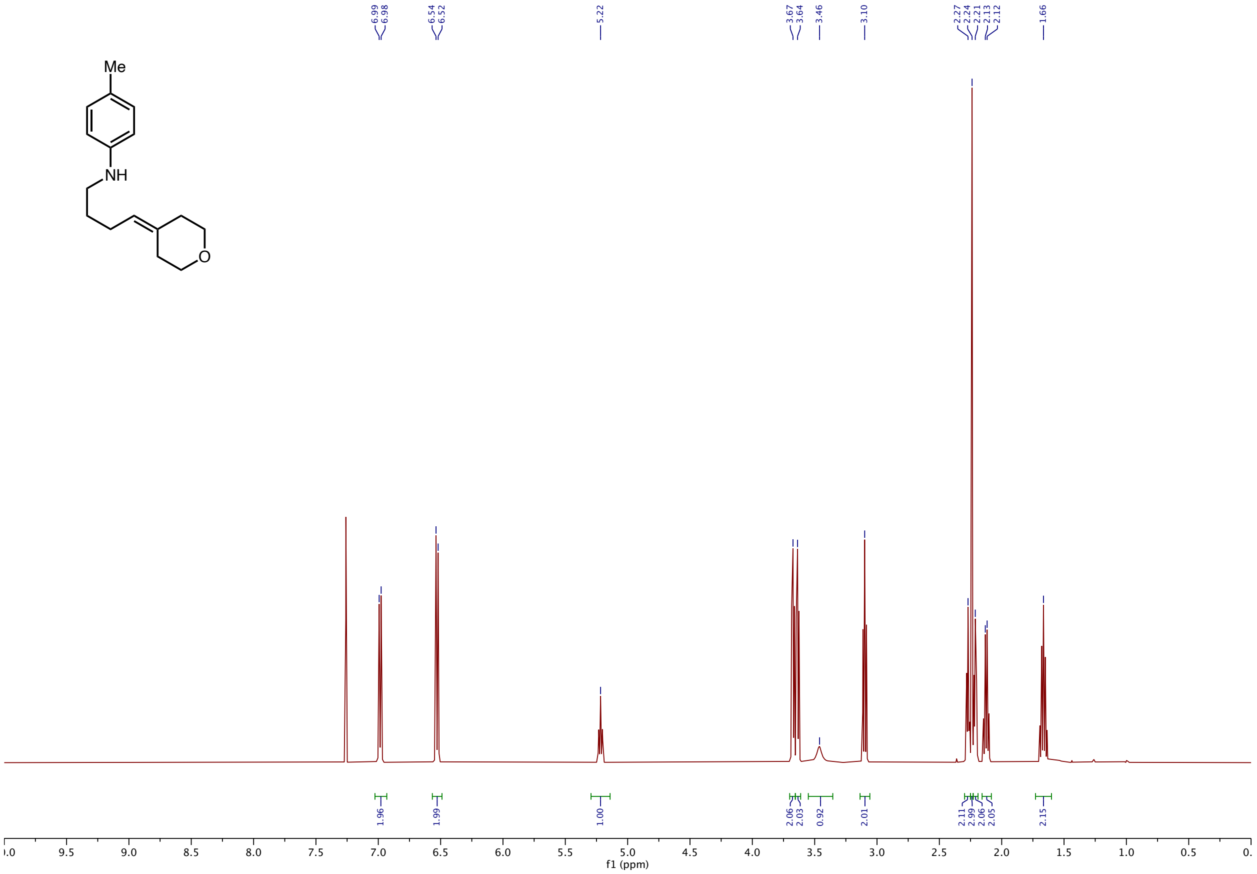
Me
NH
O
6.99
6.98
6.54
6.52
5.22
3.67
3.64
3.46
3.10
2.27
2.24
2.21
2.13
2.12
1.66
1.96
1.99
1.00
2.06
2.03
0.92
2.01
2.11
2.99
2.06
2.05
2.15
f1 (ppm)

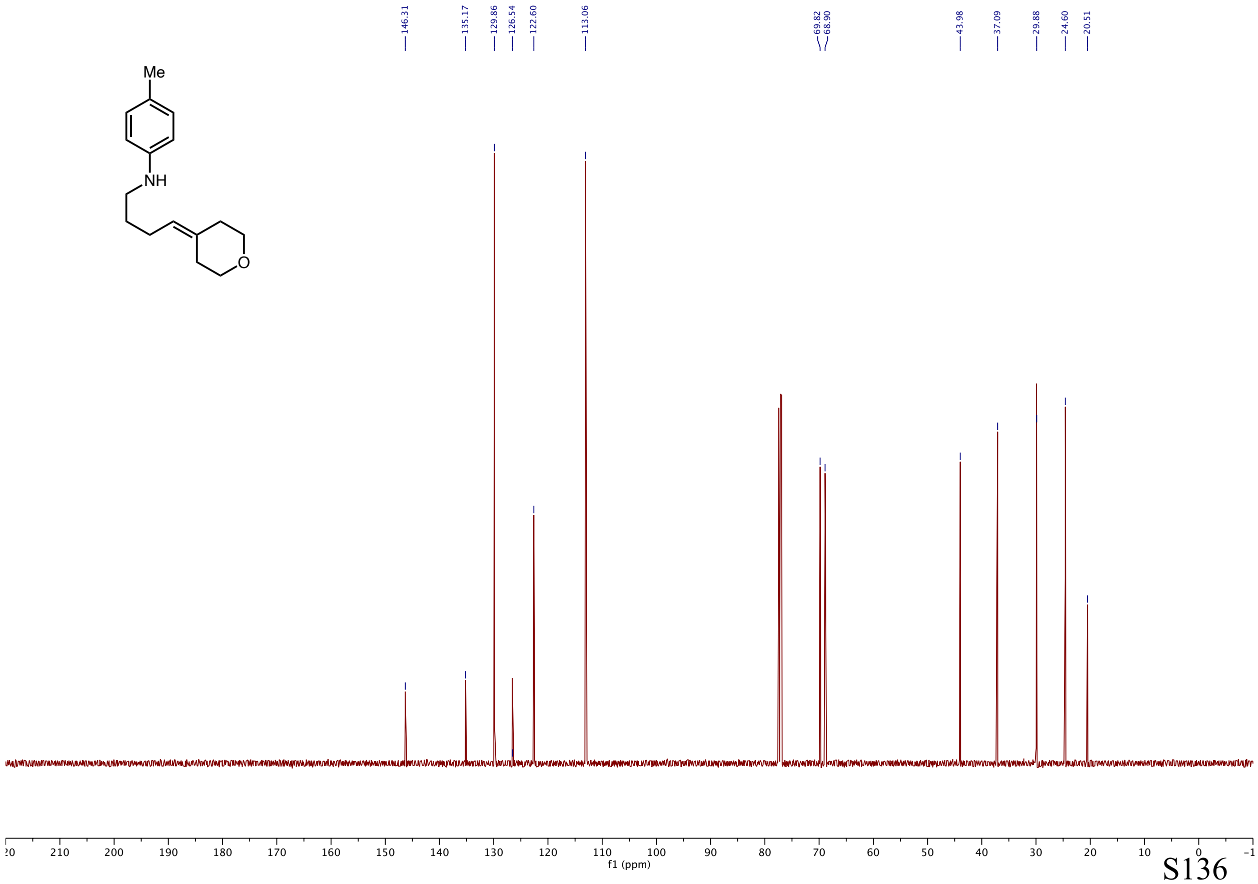
Me
NH
O
146.31
135.17
129.86
126.54
122.60
113.06
69.82
68.90
43.98
37.09
29.88
24.60
20.51
f1 (ppm)

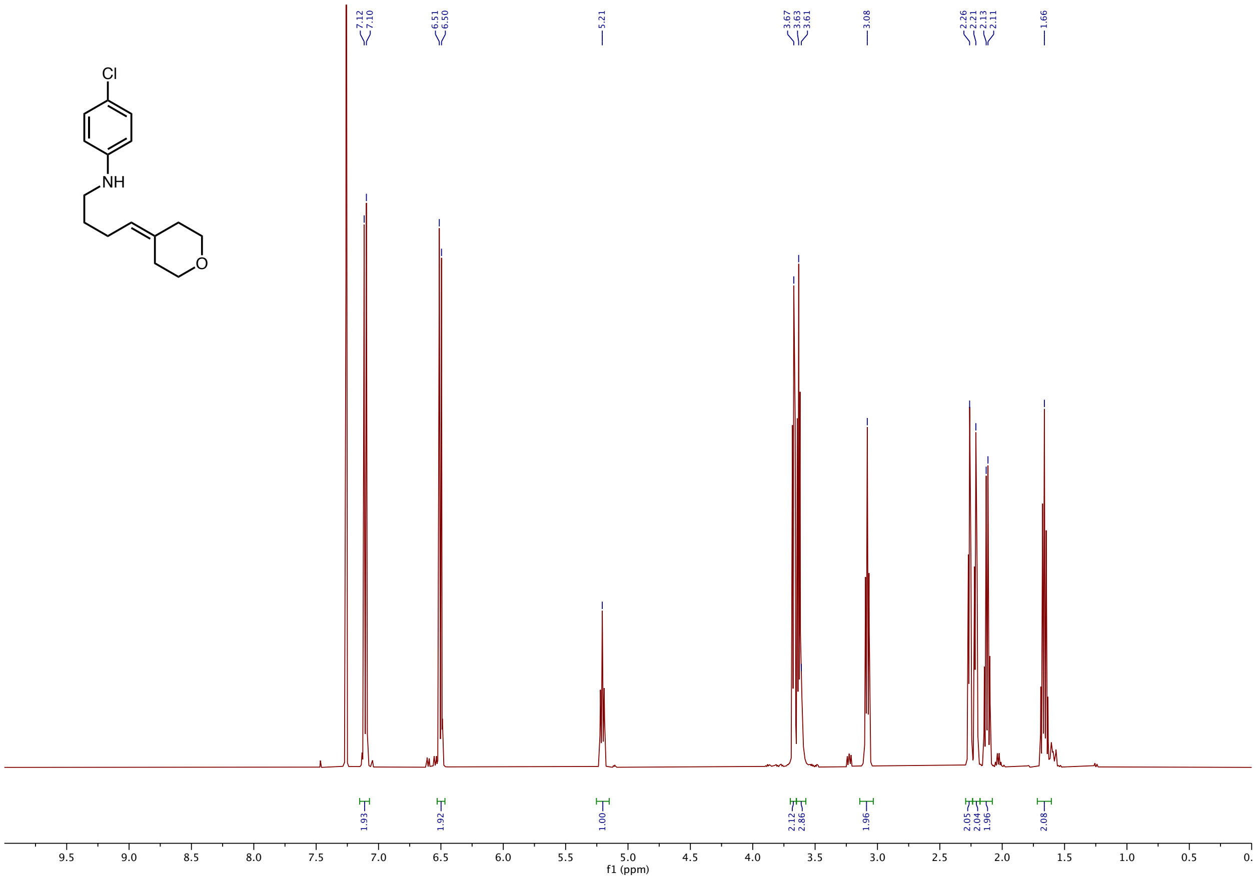
Cl
NH
O
7.12
7.10
6.51
6.50
5.21
3.67
3.63
3.61
3.08
2.26
2.21
2.13
2.11
1.66
1.93
1.92
1.00
2.12
2.86
1.96
2.05
2.04
1.96
2.08
f1 (ppm)

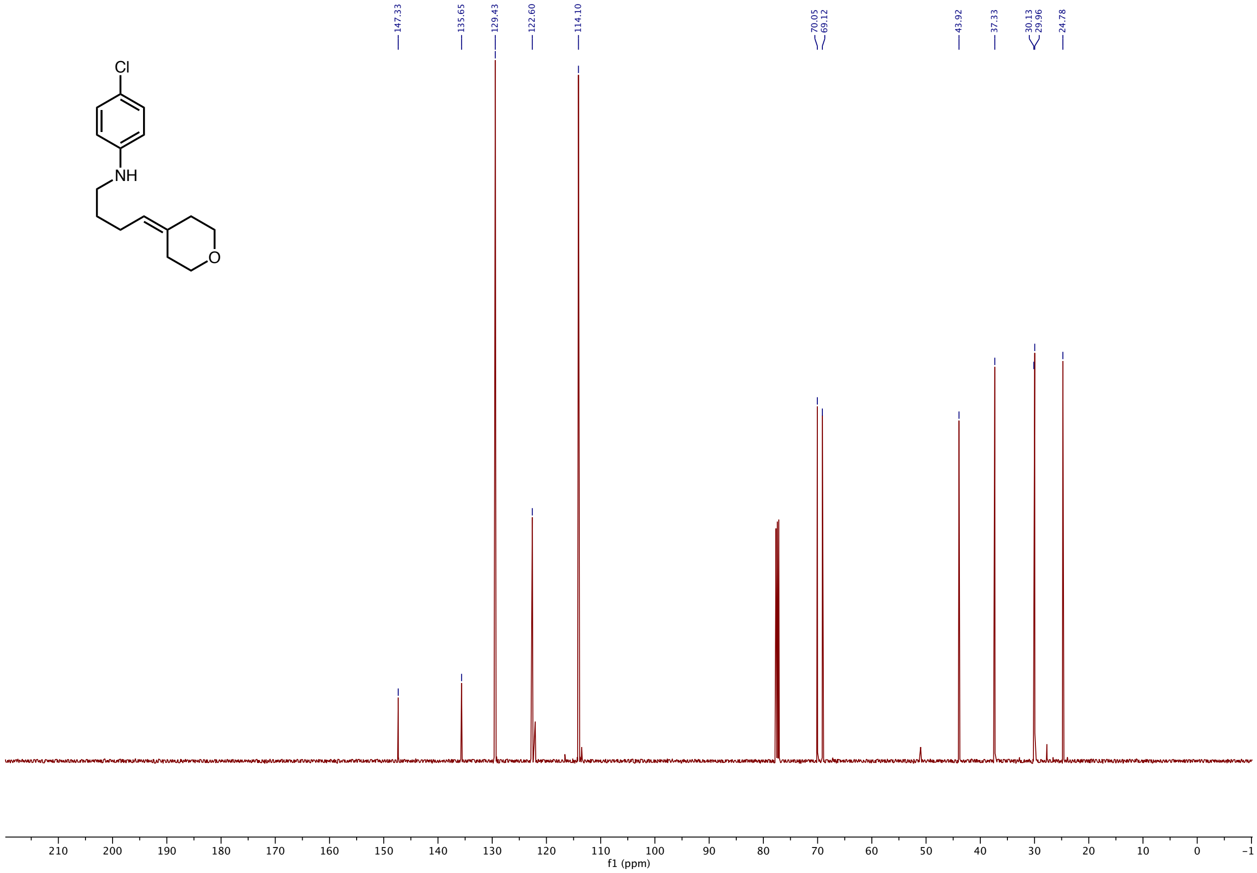
Cl
NH
O
147.33
135.65
129.43
122.60
114.10
70.05
69.12
43.92
37.33
30.13
29.96
24.78
f1 (ppm)

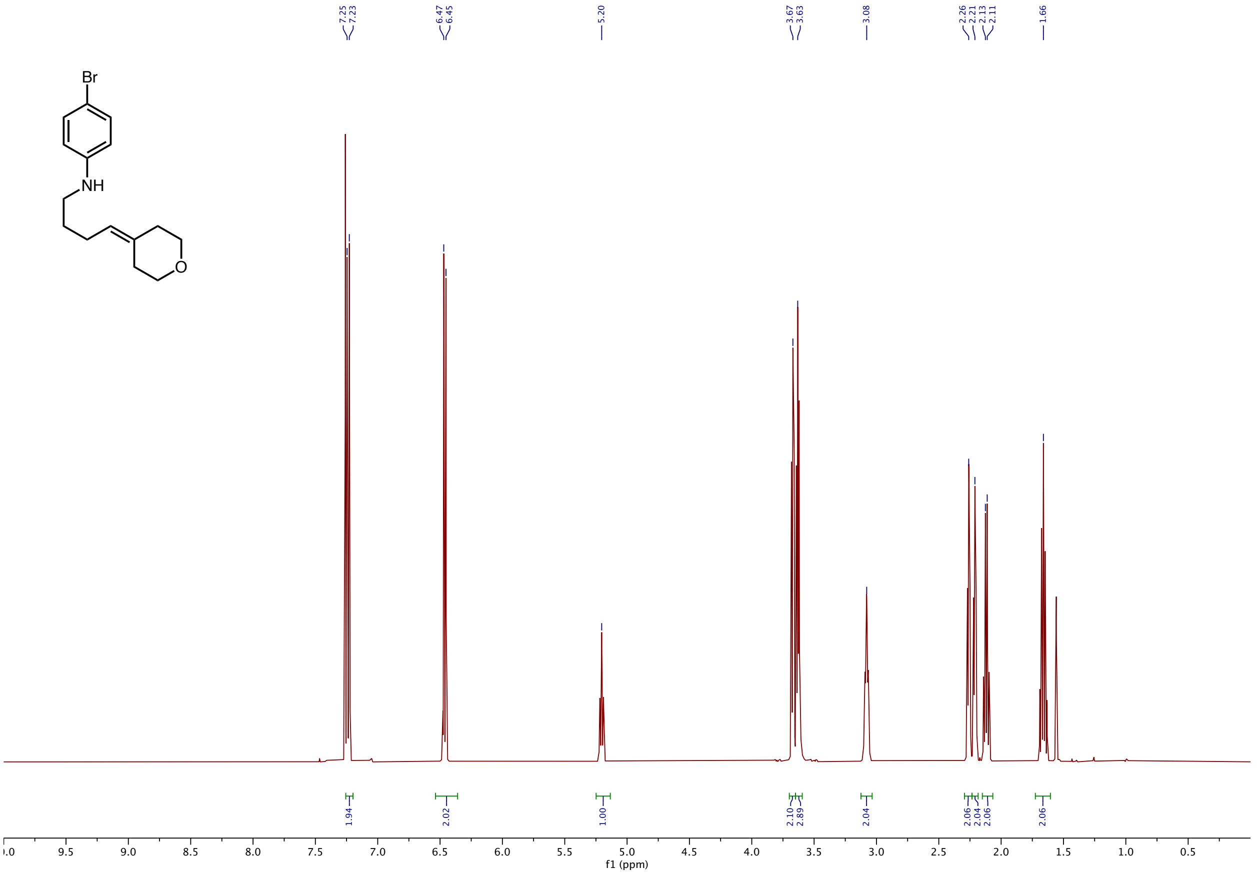

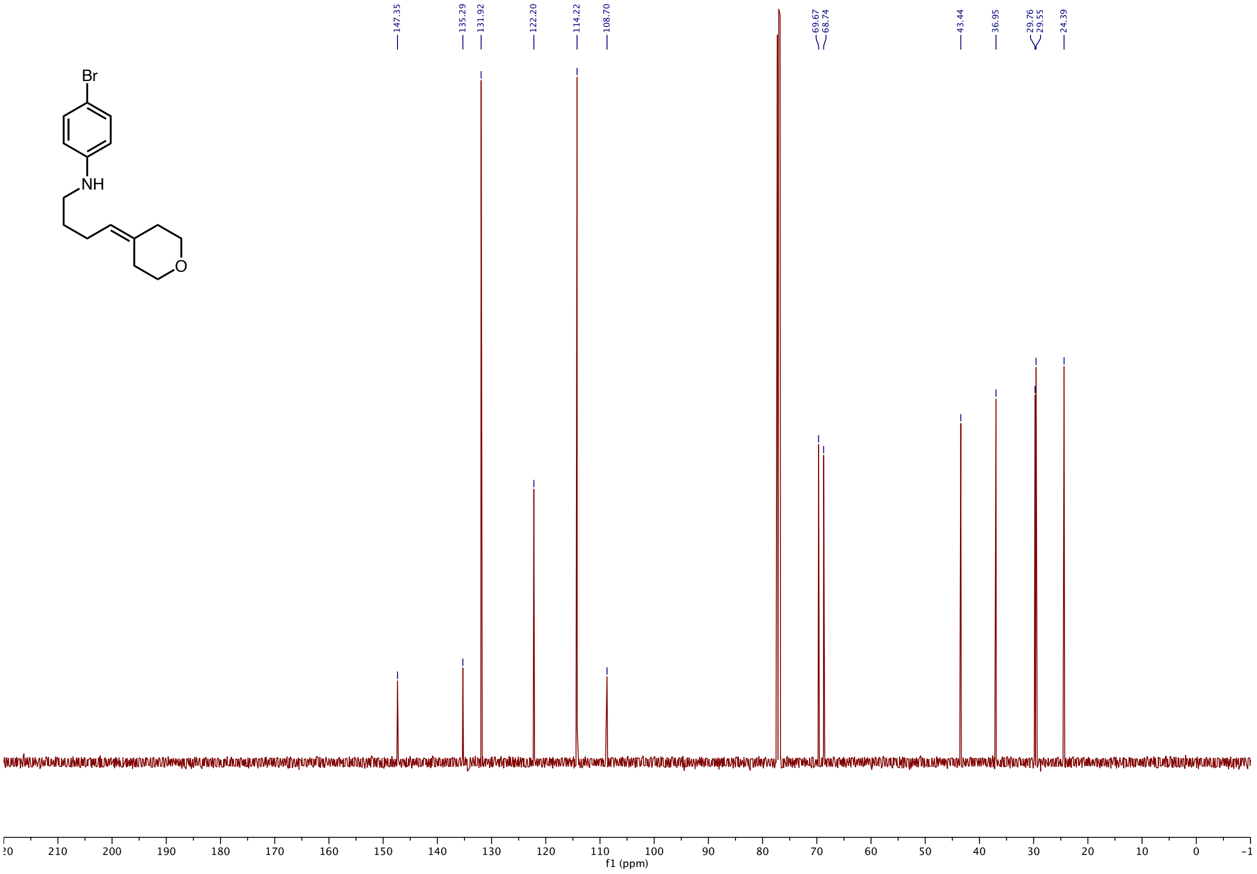

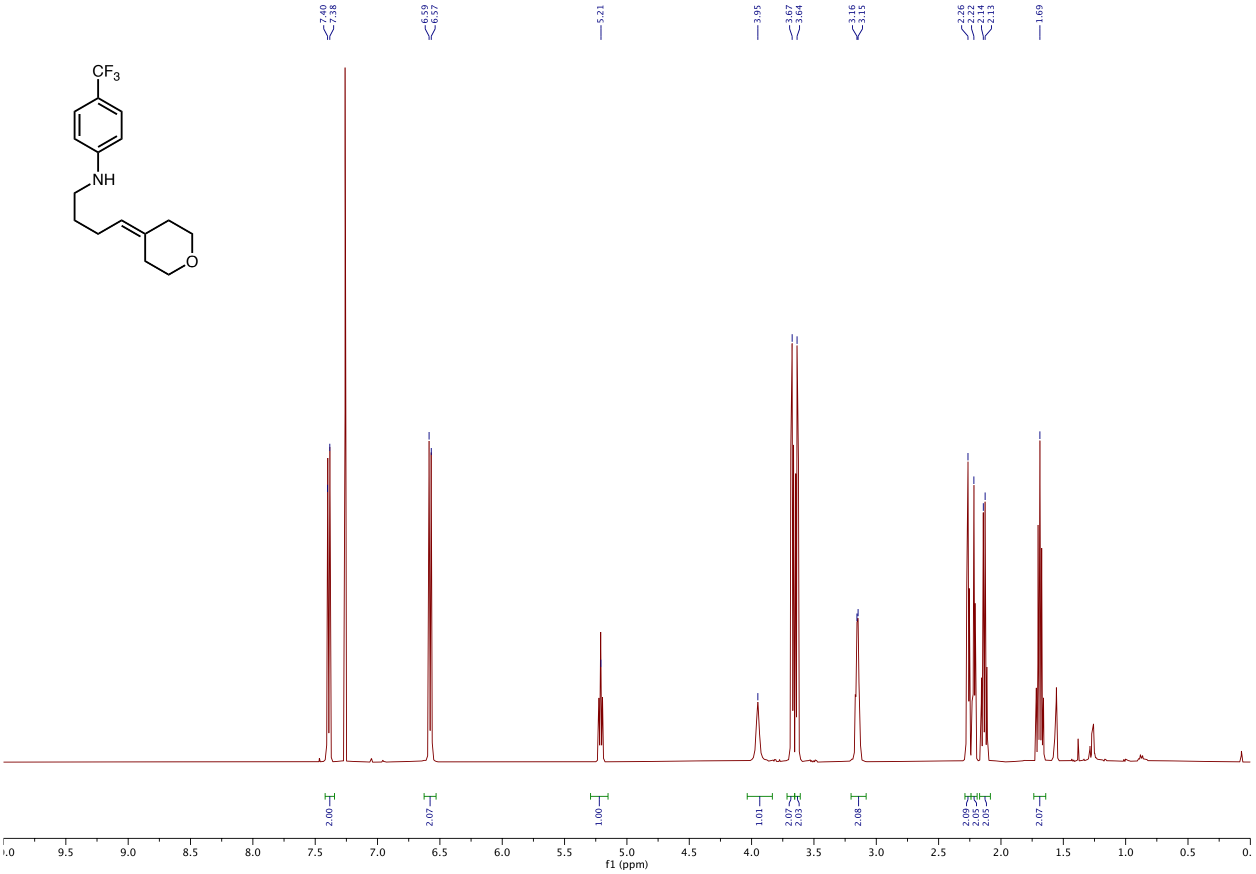
7.40
7.38
6.59
6.57
5.21
3.95
3.67
3.64
3.16
3.15
2.26
2.22
2.14
2.13
1.69
CF3
NH
O
2.00
2.07
1.00
1.01
2.07
2.03
2.08
2.09
2.05
2.05
2.07
f1 (ppm)

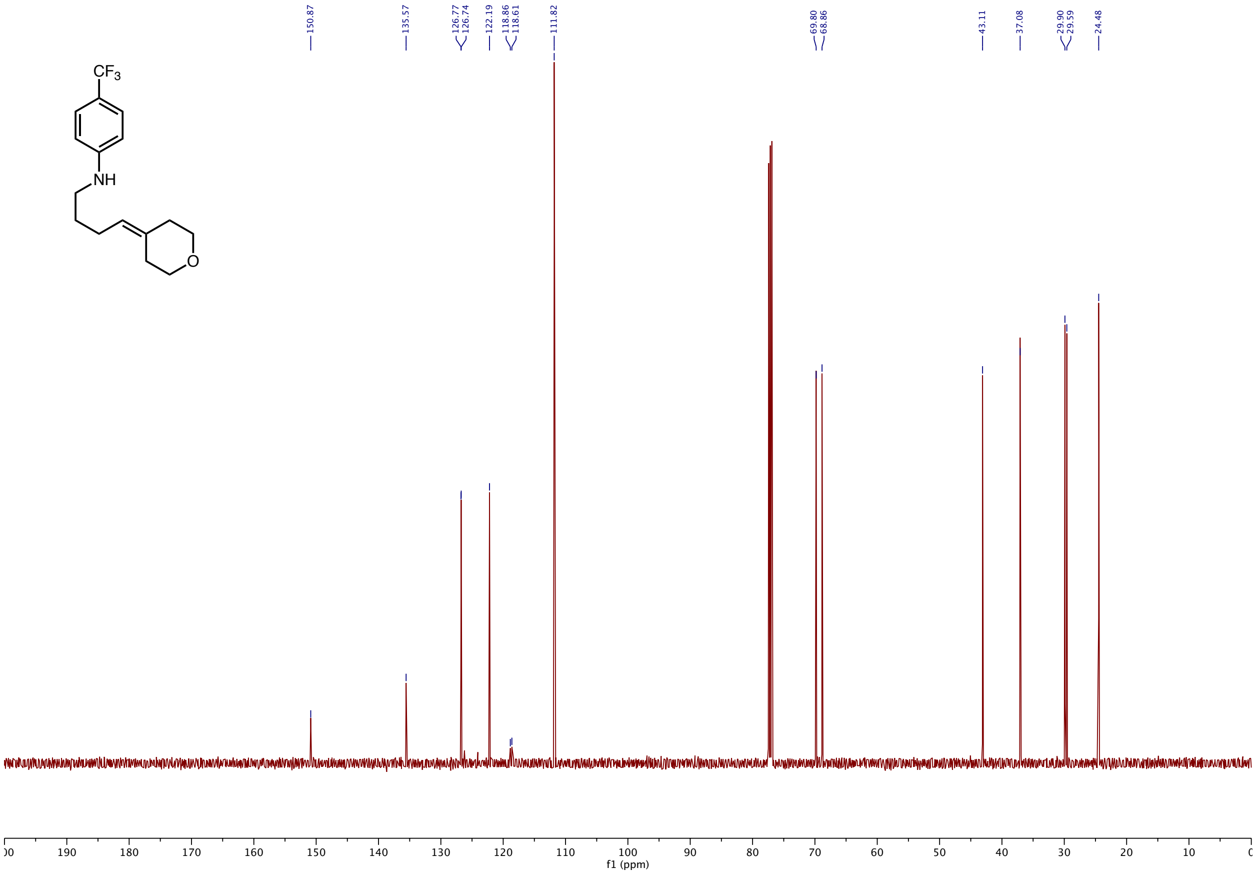
150.87
135.57
126.77
126.74
122.19
118.86
118.61
111.82
69.80
68.86
43.11
37.08
29.90
29.59
24.48
CF3
NH
O
f1 (ppm)

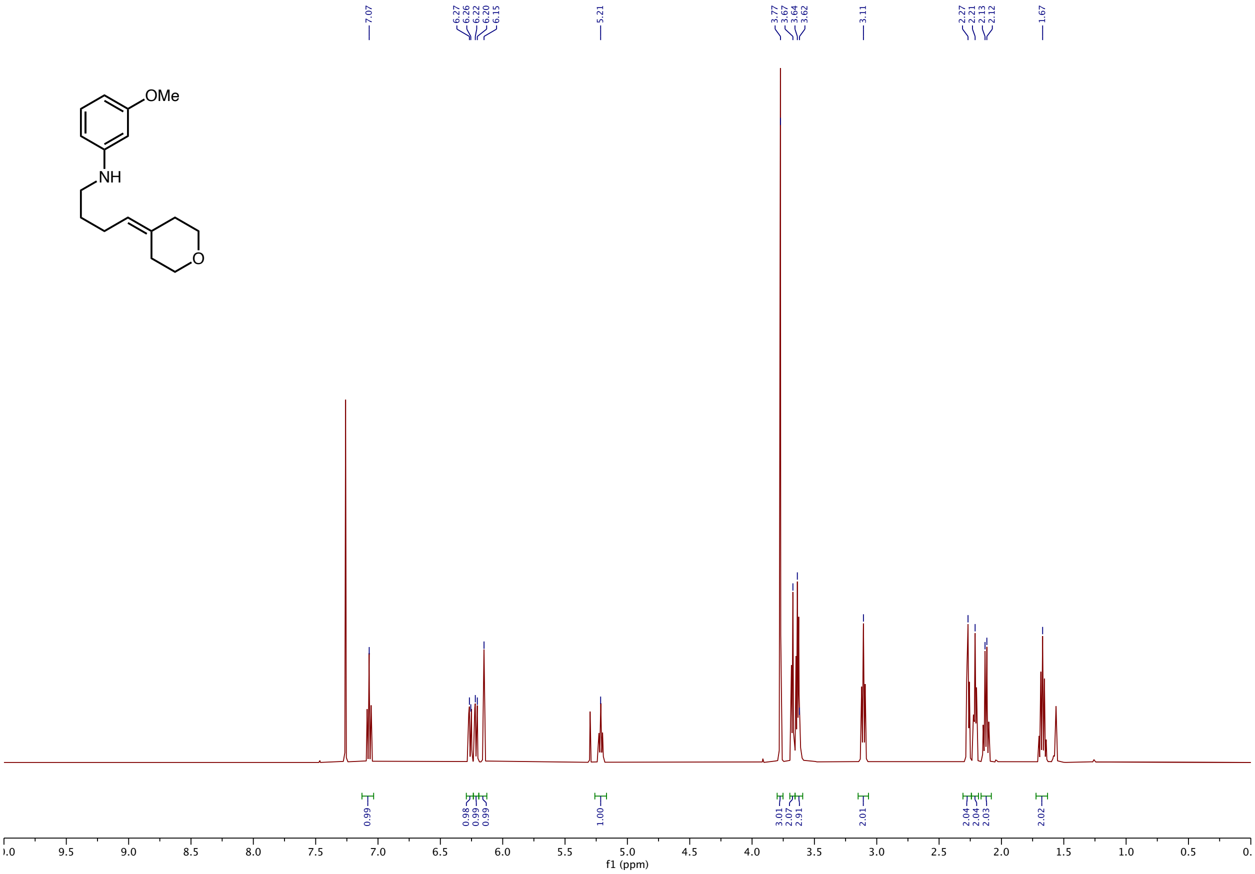
OMe
NH
O
f1 (ppm)

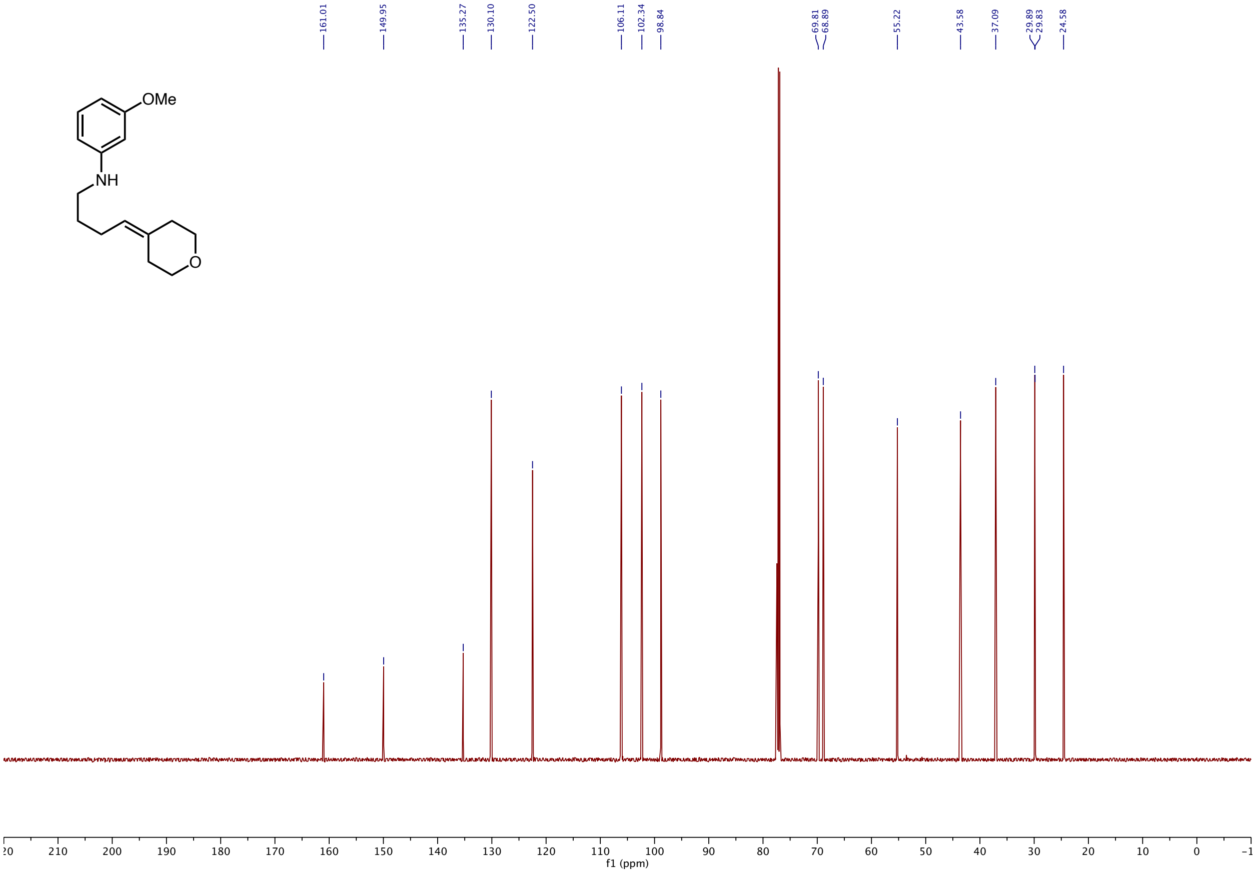
OMe
NH
O
f1 (ppm)

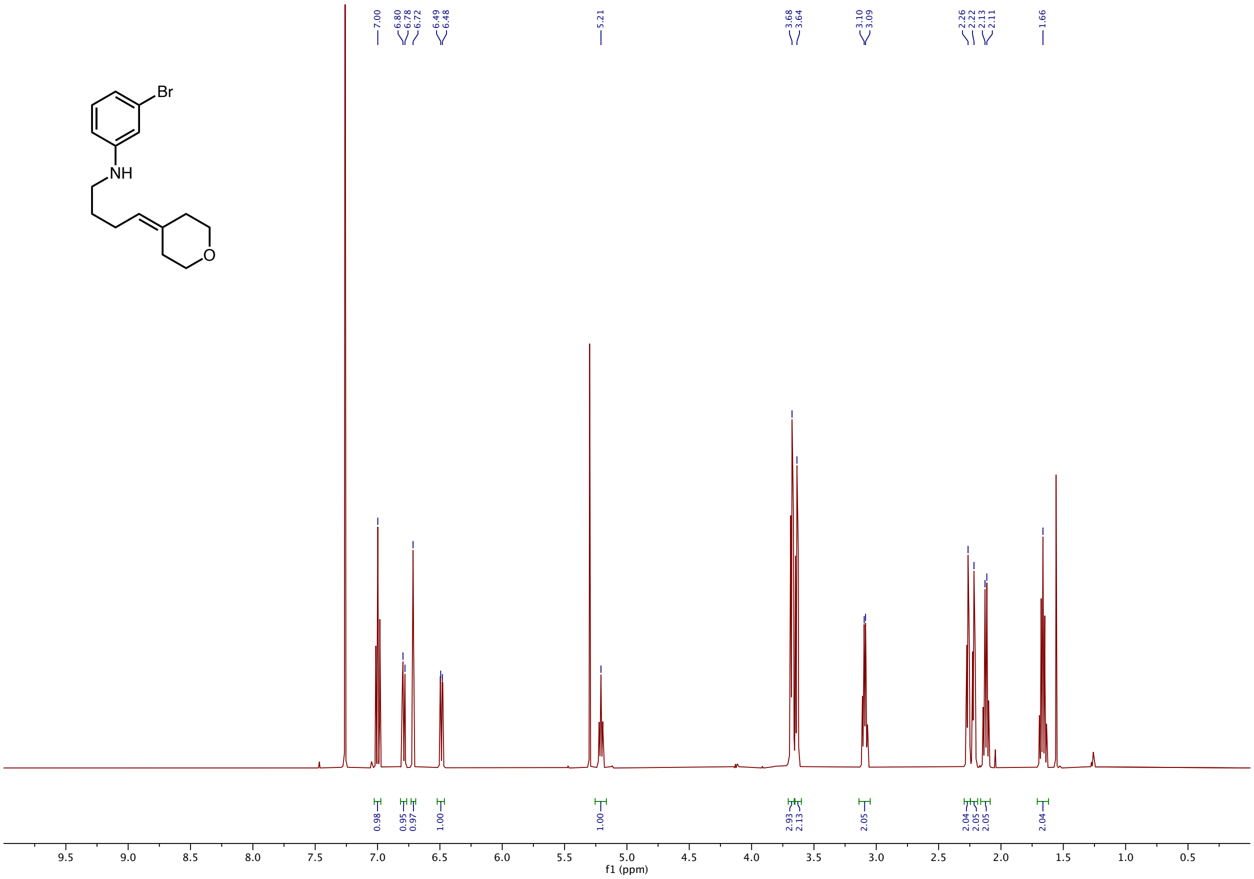
Br
NH
O
7.00
6.80
6.78
6.72
6.49
6.48
5.21
3.68
3.64
3.10
3.09
2.26
2.22
2.13
2.11
1.66
0.98
0.95
0.97
1.00
1.00
2.93
2.13
2.05
2.04
2.05
2.05
2.04
f1 (ppm)

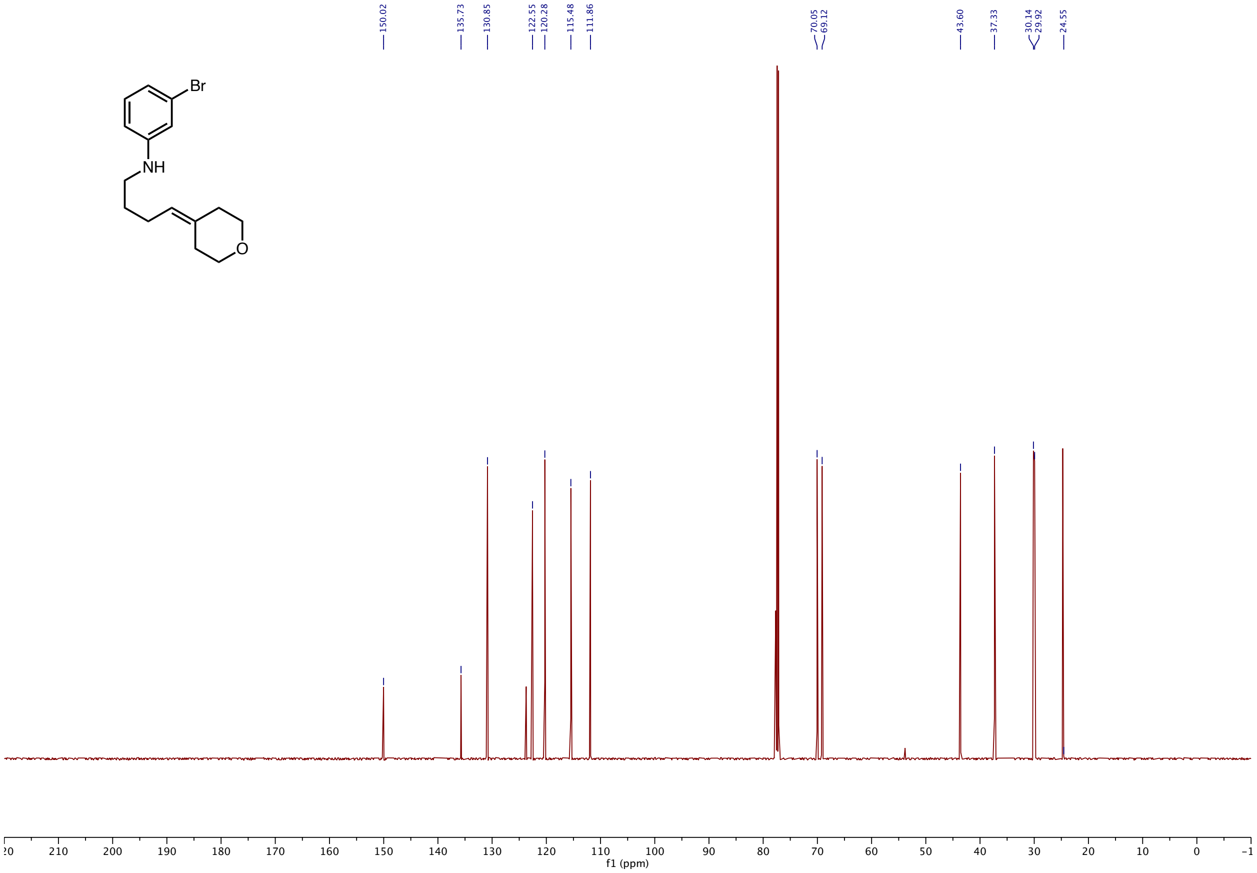
Br
NH
O
150.02
135.73
130.85
122.55
120.28
115.48
111.86
70.05
69.12
43.60
37.33
30.14
29.92
24.55
f1 (ppm)

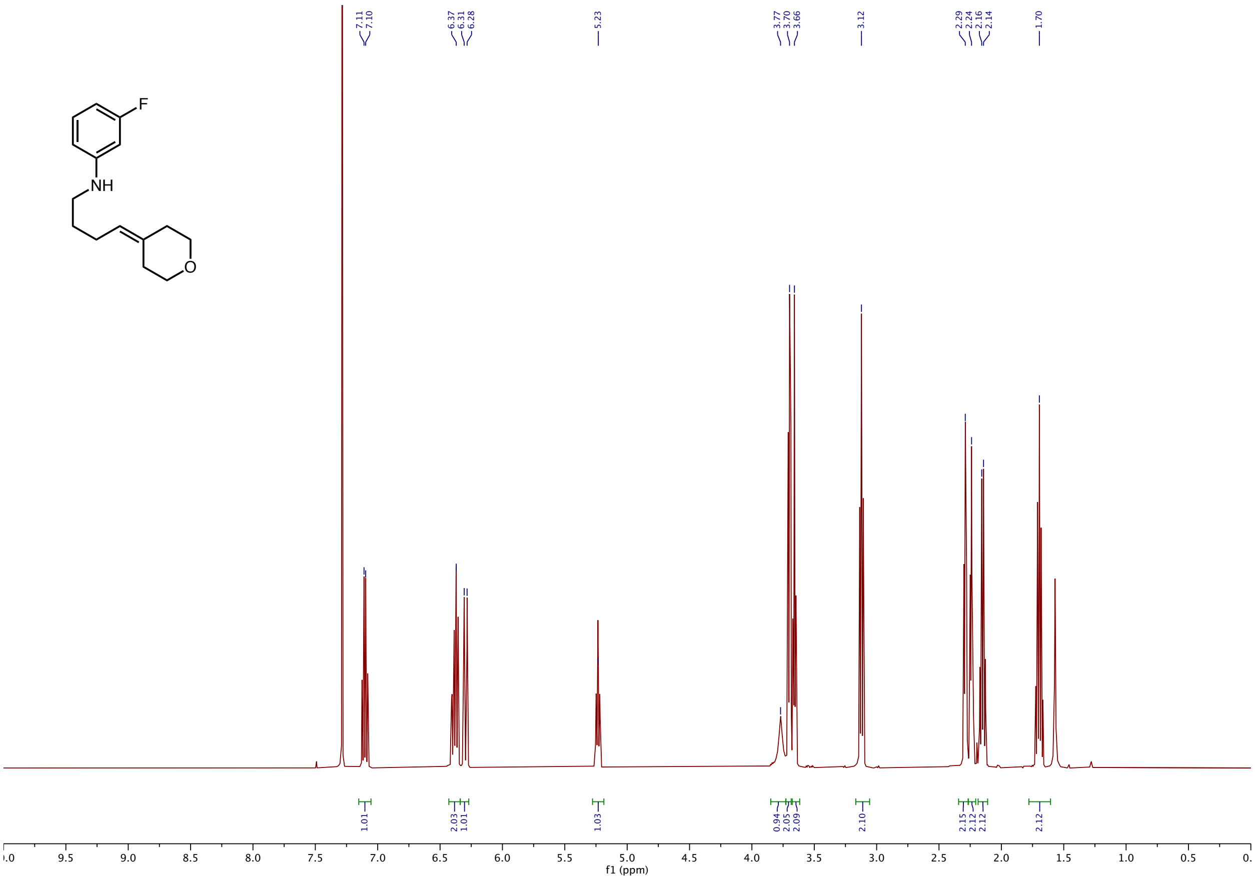

7.11
7.10
6.37
6.31
6.28
5.23
3.77
3.70
3.66
3.12
2.29
2.24
2.16
2.14
1.70
F
NH
O
1.01
2.03
1.01
1.03
0.94
2.05
2.09
2.10
2.15
2.12
2.12
2.12
f1 (ppm)

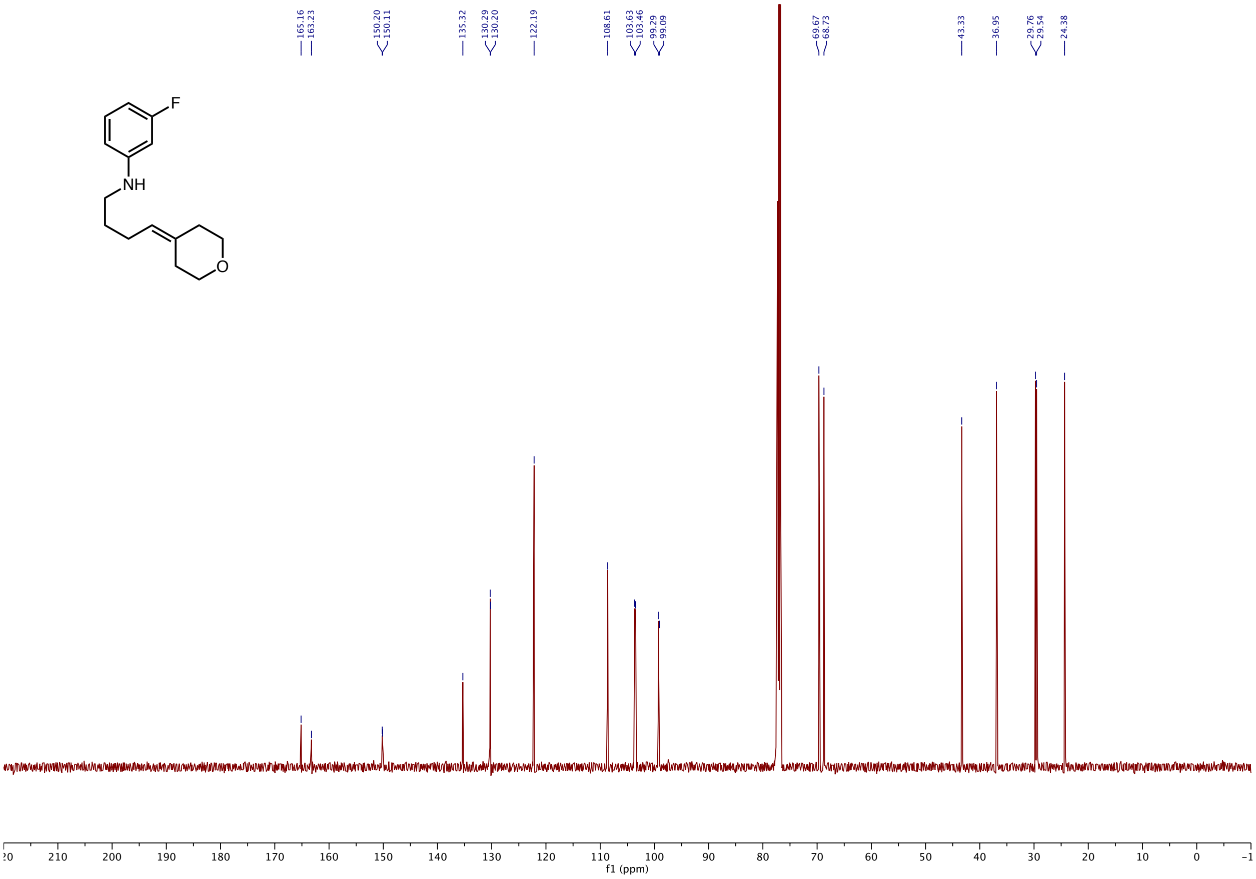

165.16
163.23
150.20
150.11
135.32
130.29
130.20
122.19
108.61
103.63
103.46
99.29
99.09
69.67
68.73
43.33
36.95
29.76
29.54
24.38
F
NH
O
f1 (ppm)

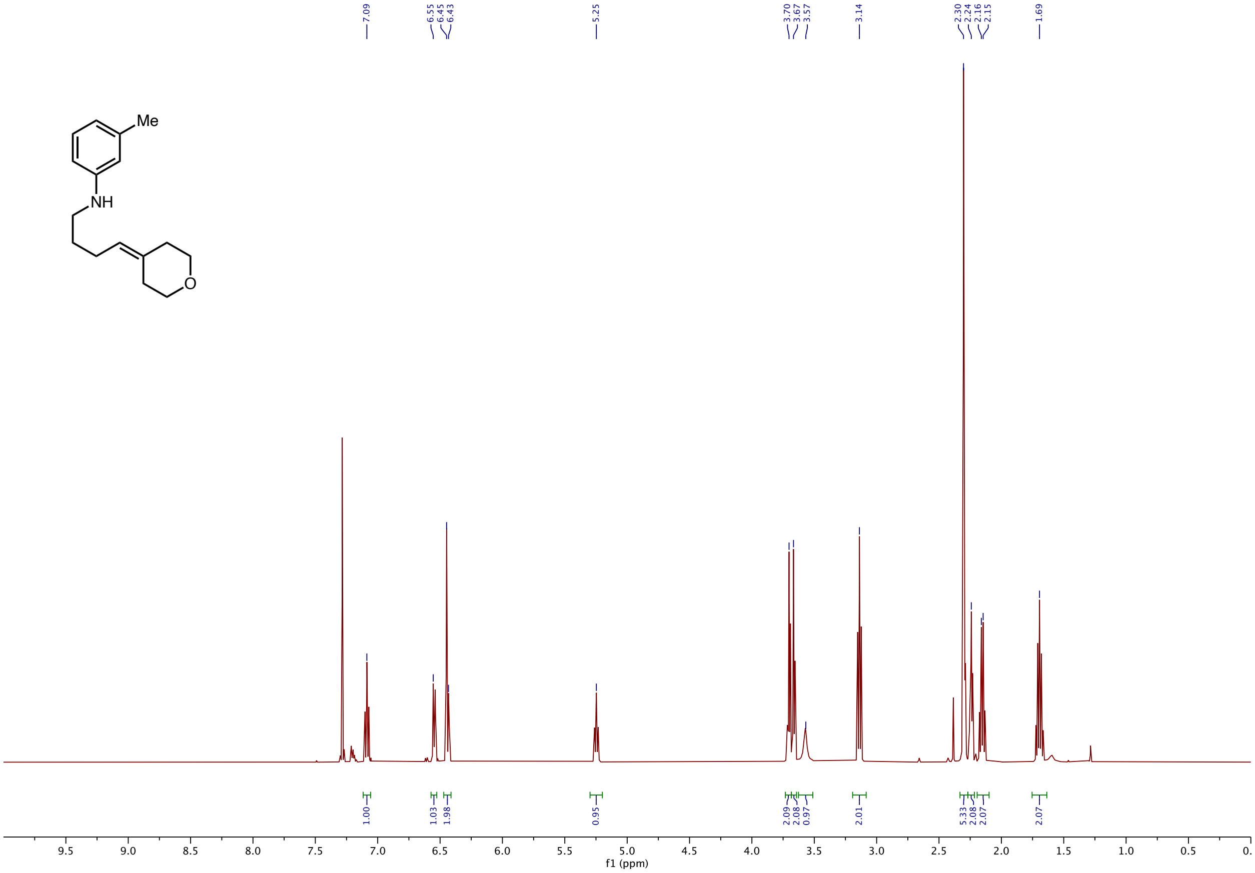

Me
NH
O
7.09
6.55 6.45 6.43
5.25
3.70 3.67 3.57
3.14
2.30 2.24 2.16 2.15
1.69
1.00 1.03 1.98 0.95 2.09 2.08 0.97 2.01 5.33 2.08 2.07 2.07
9.5 9.0 8.5 8.0 7.5 7.0 6.5 6.0 5.5 5.0 4.5 4.0 3.5 3.0 2.5 2.0 1.5 1.0 0.5
f1 (ppm)

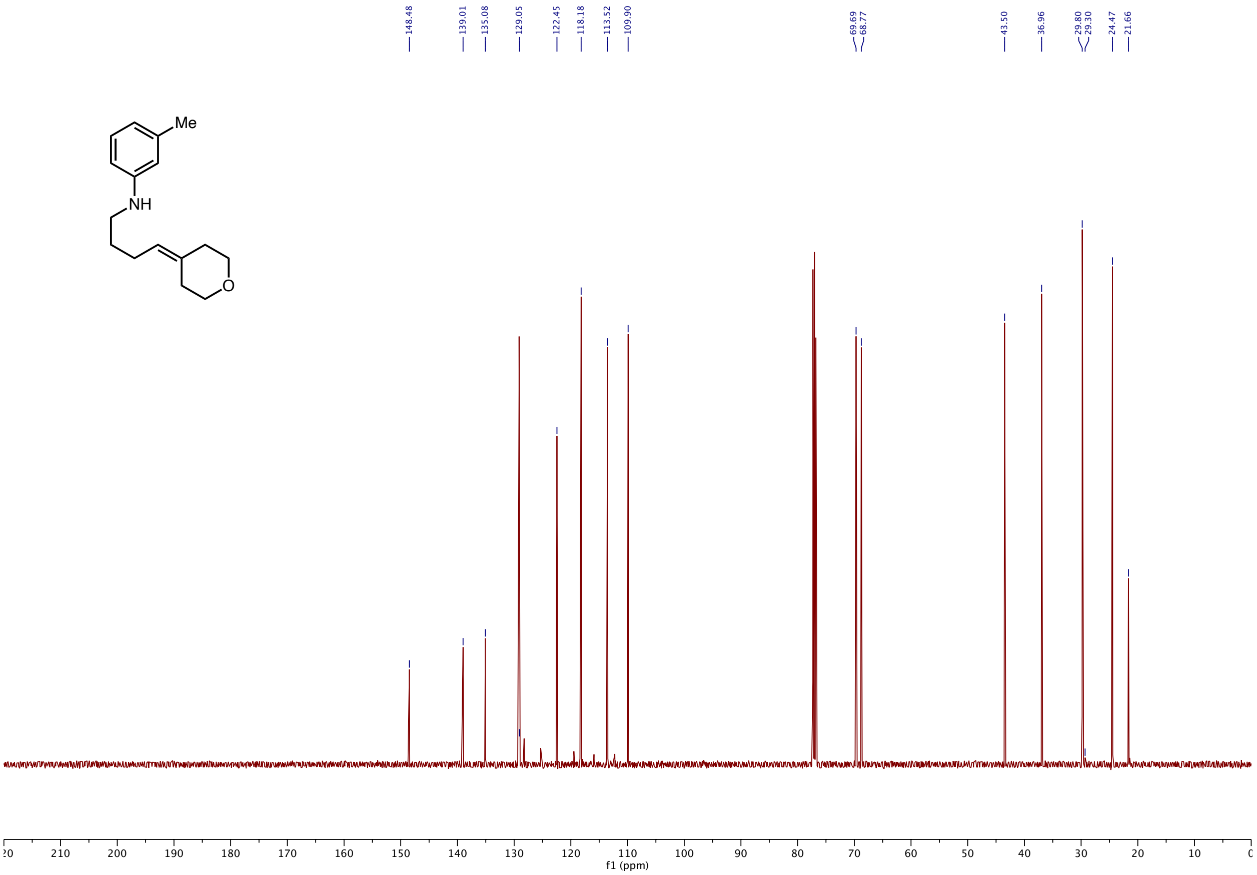

Me
NH
O
148.48
139.01 135.08
129.05
122.45 118.18 113.52 109.90
69.69 68.77
43.50
36.96
29.80 29.30
24.47 21.66
210 200 190 180 170 160 150 140 130 120 110 100 90 80 70 60 50 40 30 20 10
f1 (ppm)

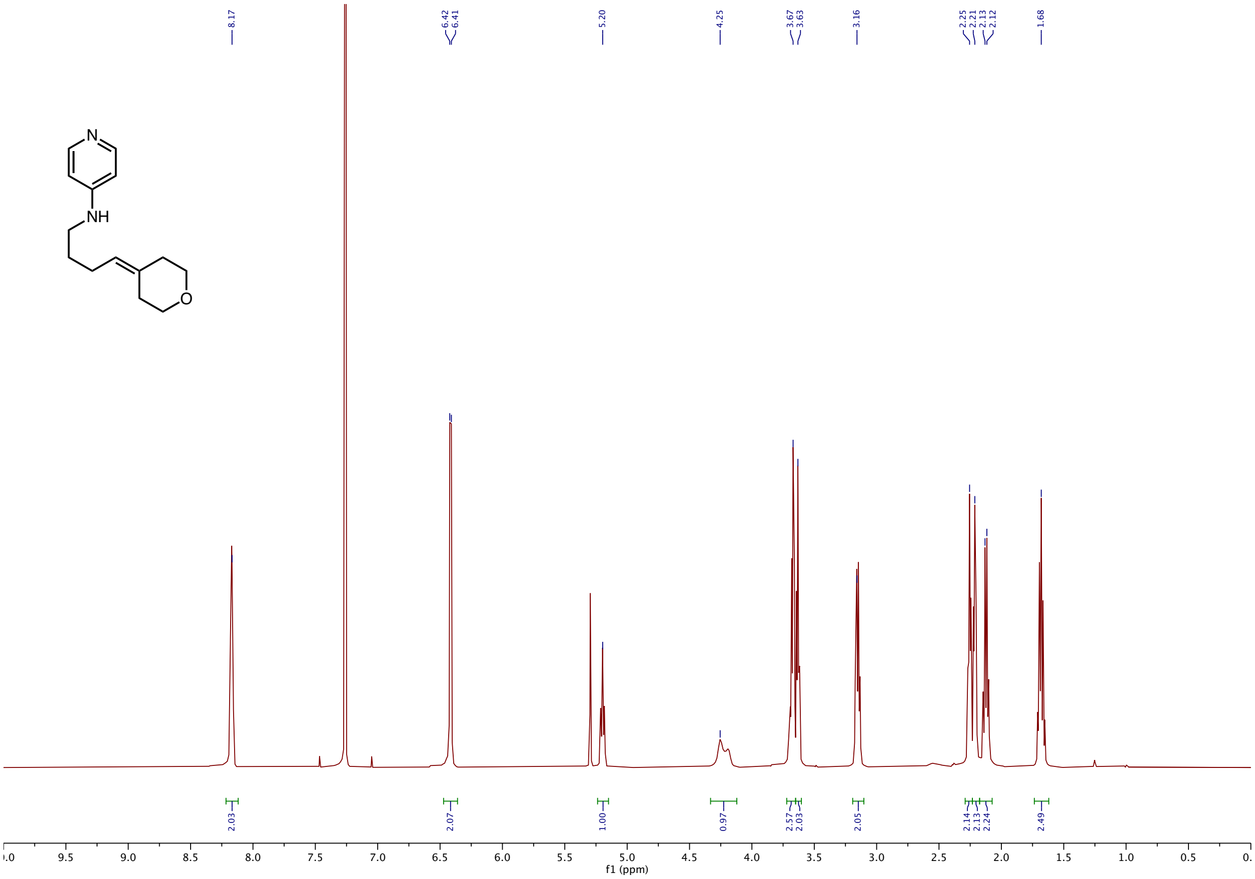
N
NH
O
8.17
6.42
6.41
5.20
4.25
3.67
3.63
3.16
2.25
2.21
2.13
2.12
1.68
2.03
2.07
1.00
0.97
2.57
2.03
2.05
2.14
2.13
2.24
2.49
f1 (ppm)

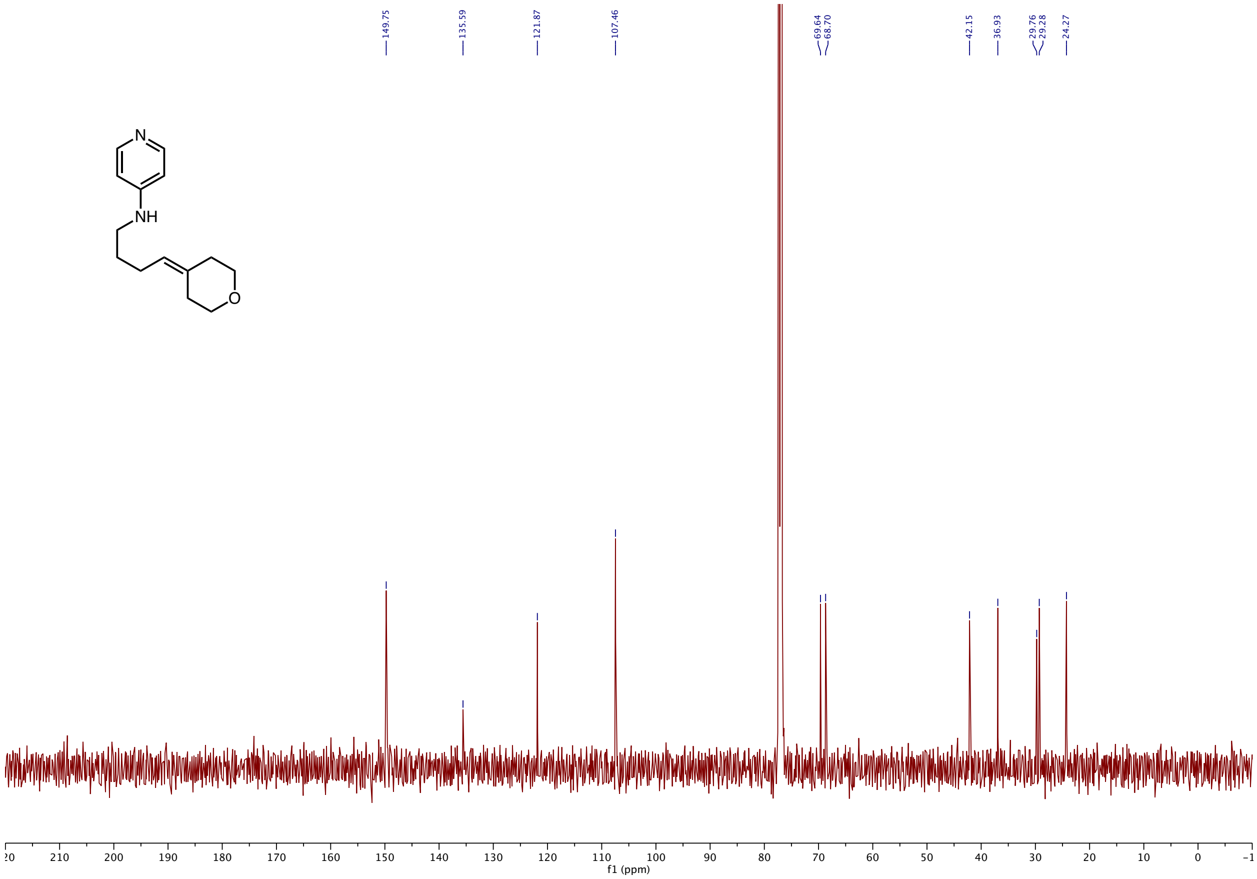
N
NH
O
149.75
135.59
121.87
107.46
69.64
68.70
42.15
36.93
29.76
29.28
24.27
f1 (ppm)

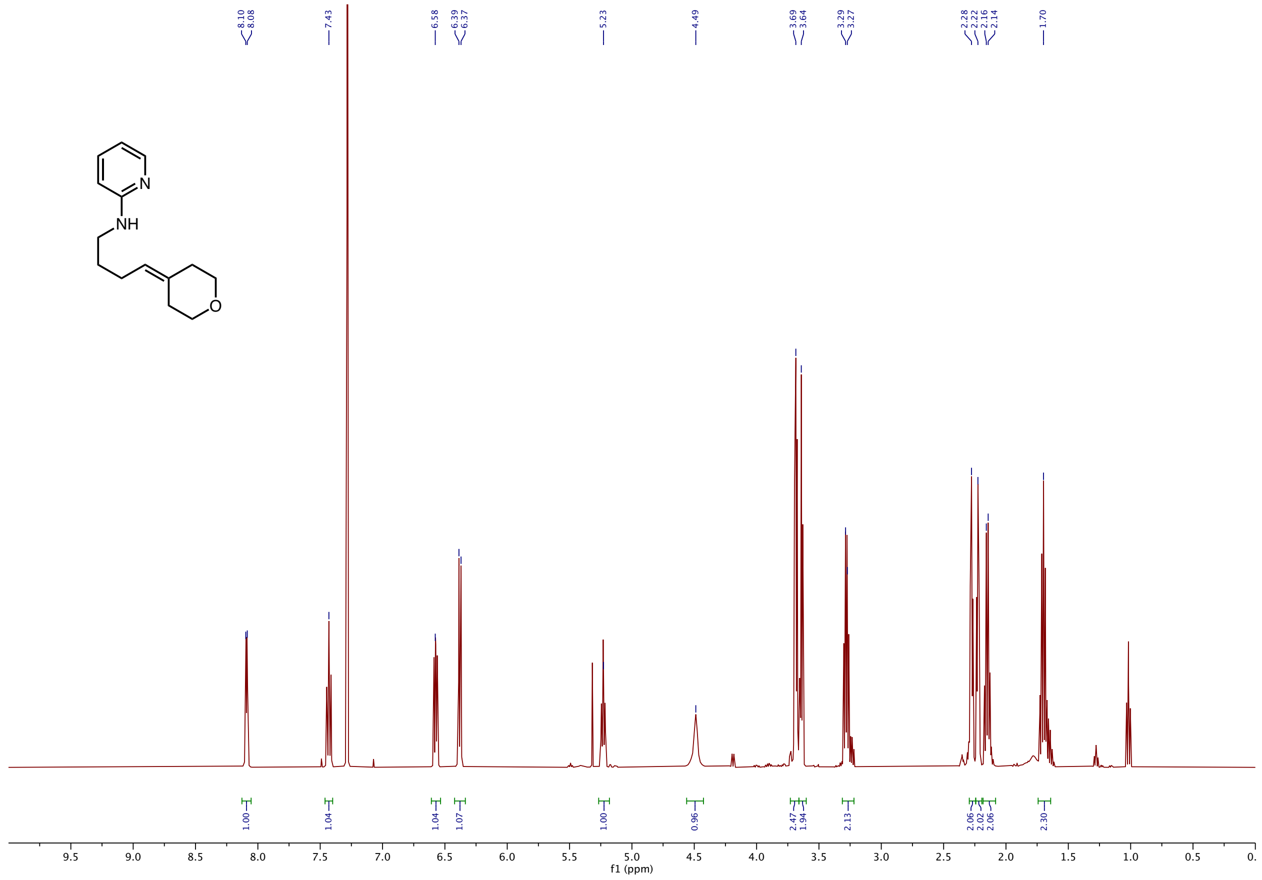

8.10
8.08
7.43
6.58
6.39
6.37
5.23
4.49
3.69
3.64
3.29
3.27
2.28
2.22
2.16
2.14
1.70
NH
N
O
1.00
1.04
1.04
1.07
1.00
0.96
2.47
1.94
2.13
2.06
2.02
2.06
2.30
f1 (ppm)

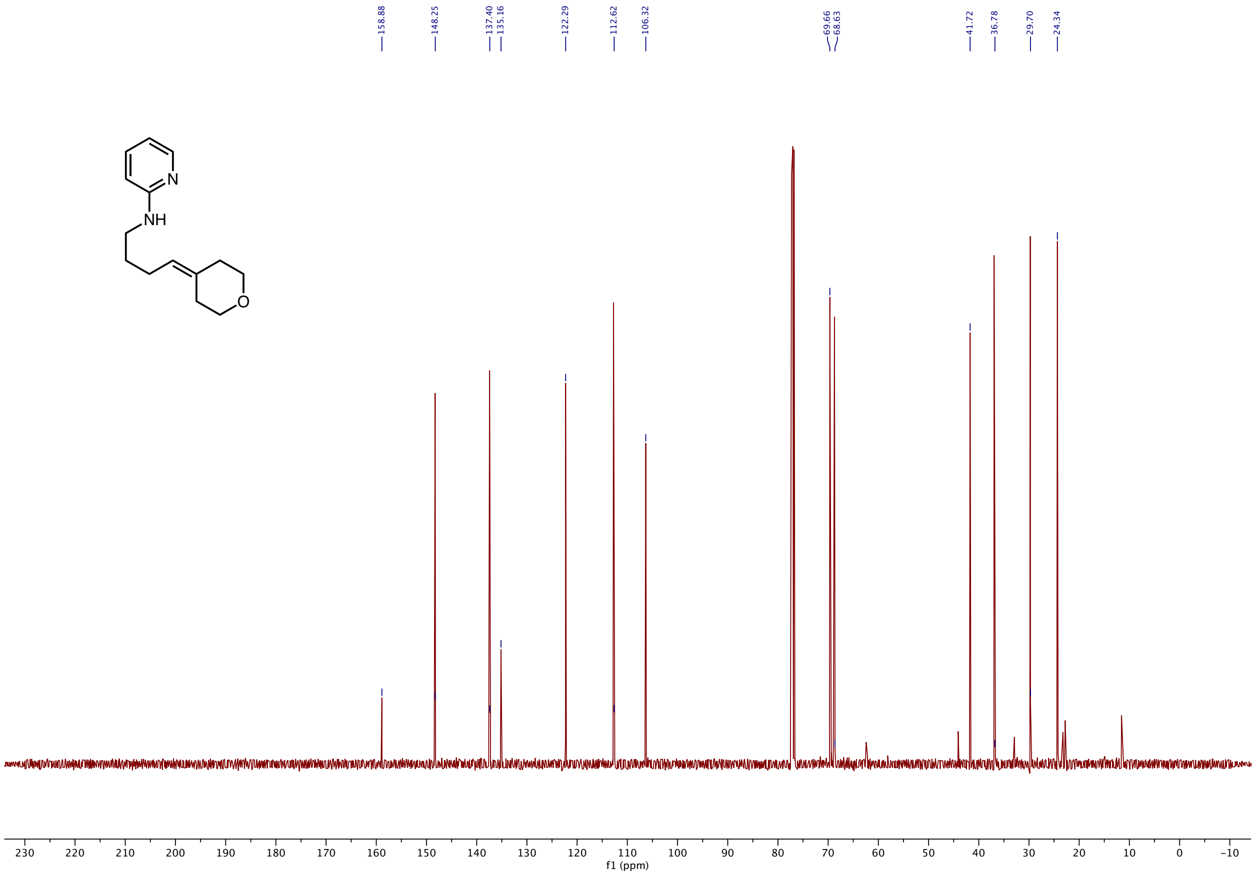

158.88
148.25
137.40
135.16
122.29
112.62
106.32
69.66
68.63
41.72
36.78
29.70
24.34
NH
N
O
f1 (ppm)

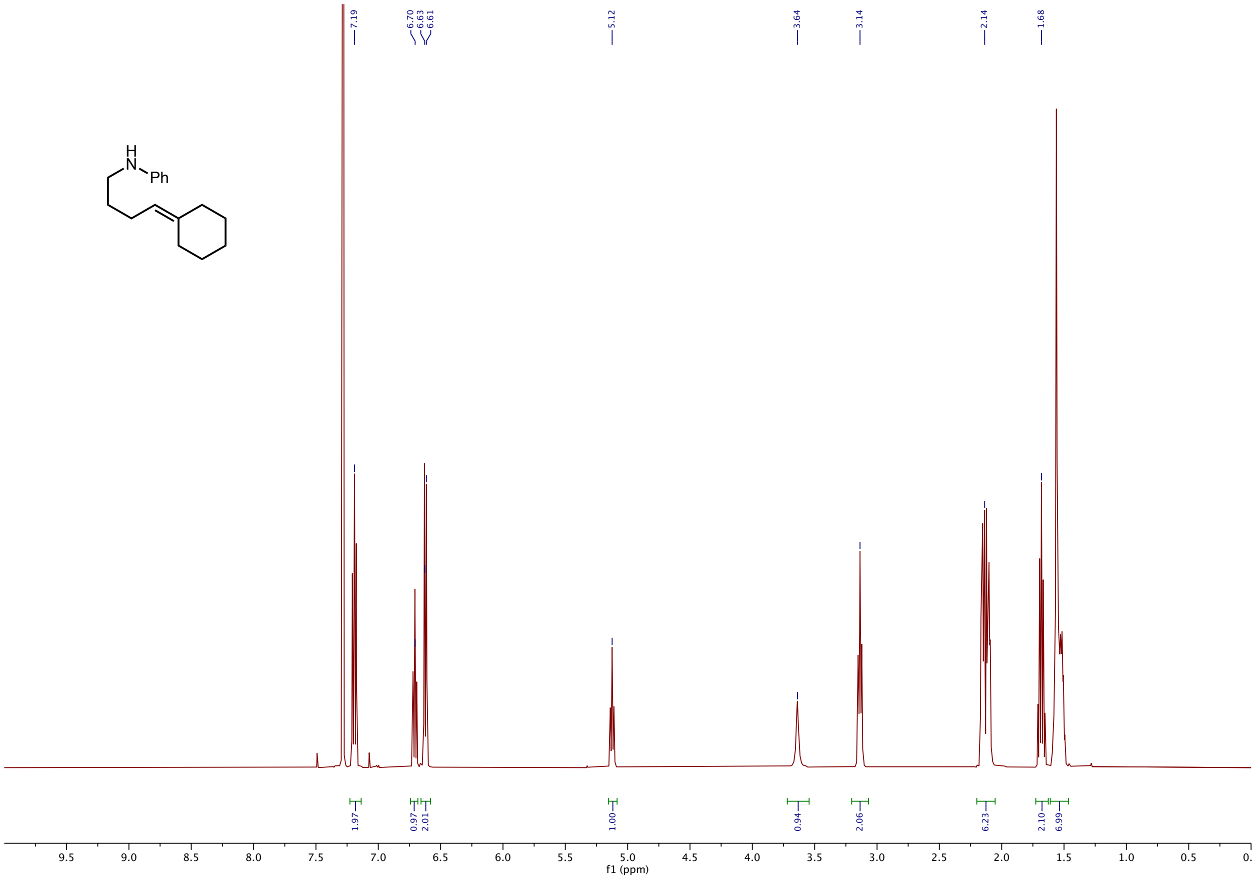

7.19
6.70
6.63
6.61
5.12
3.64
3.14
2.14
1.68
1.97
0.97
2.01
1.00
0.94
2.06
6.23
2.10
6.99
f1 (ppm)

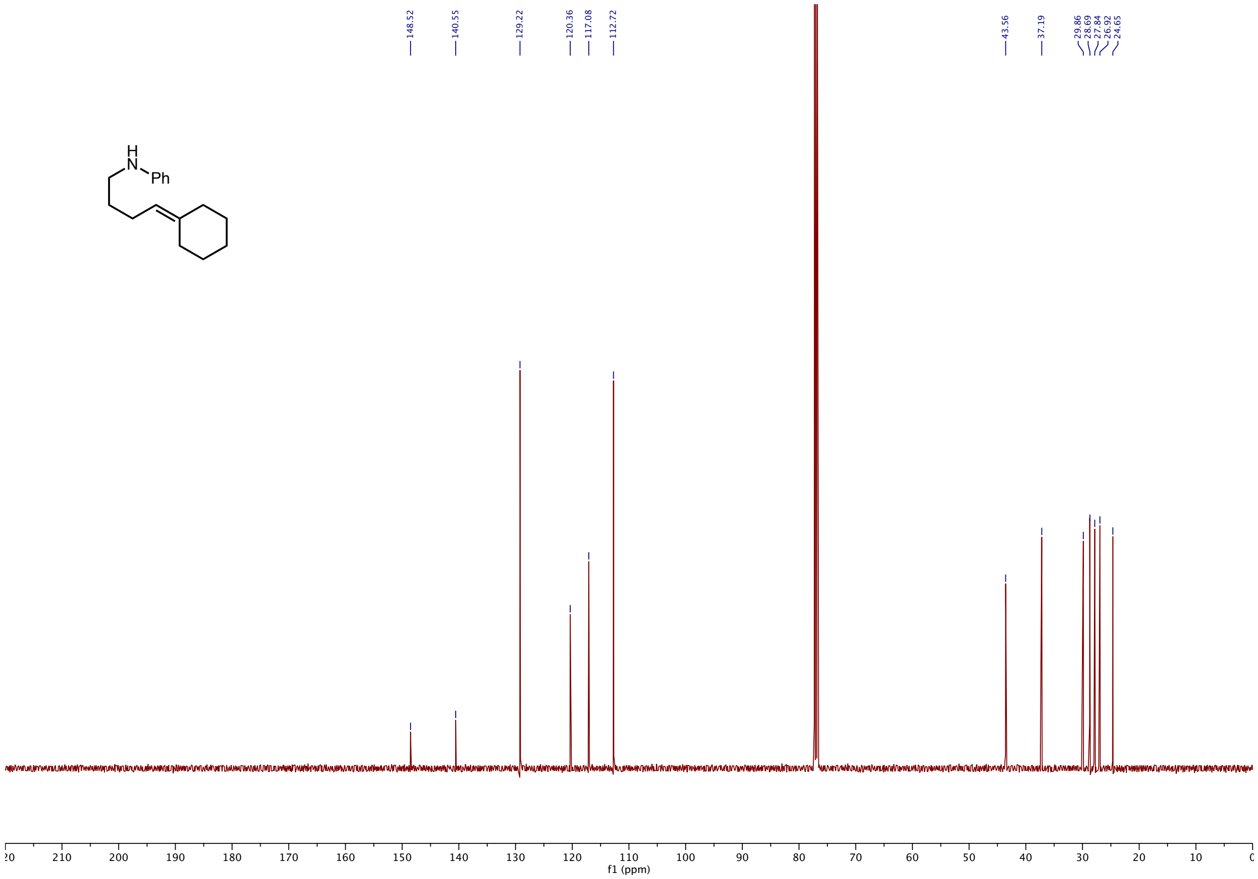

148.52
140.55
129.22
120.36
117.08
112.72
43.56
37.19
29.86
28.69
27.84
26.92
24.65
f1 (ppm)

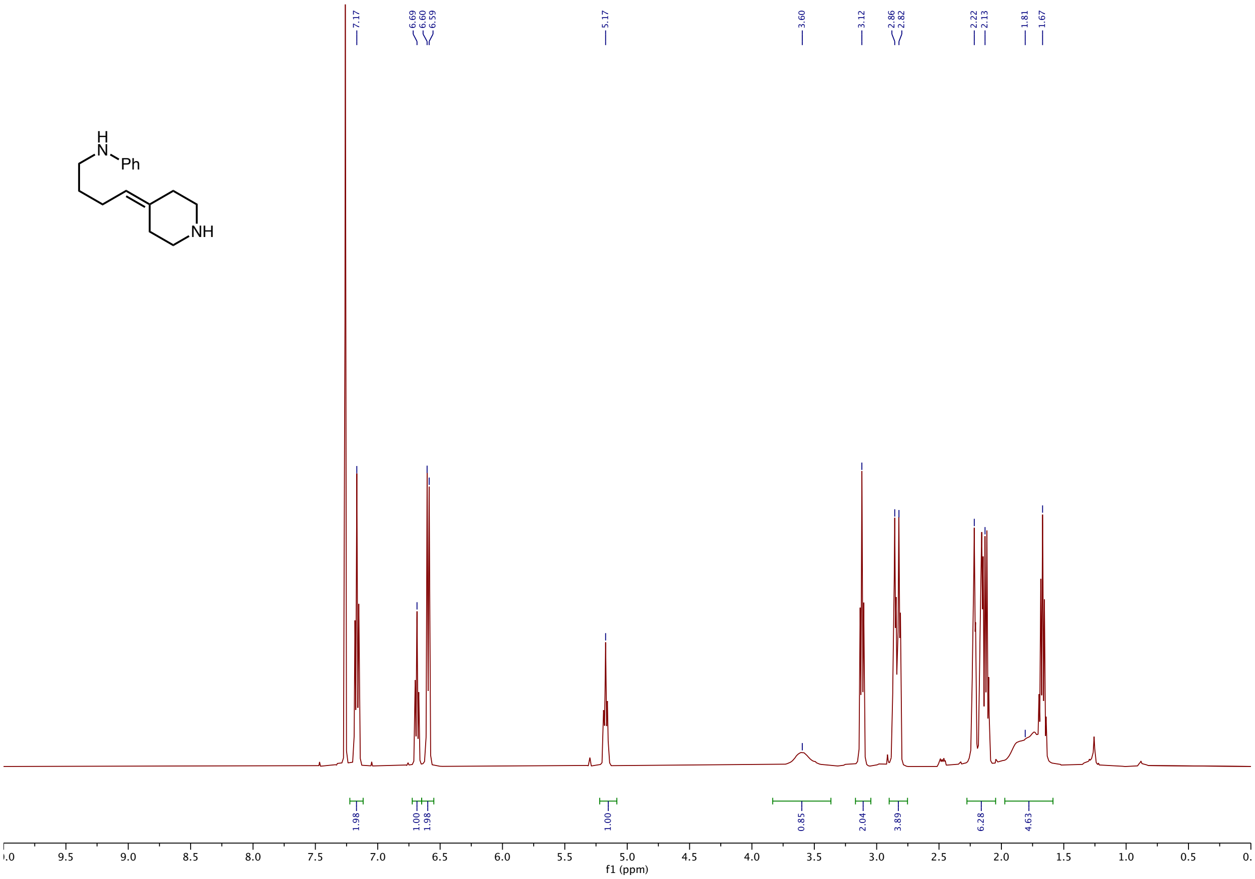

7.17
6.69
6.60
6.59
5.17
3.60
3.12
2.86
2.82
2.22
2.13
1.81
1.67
H
N
Ph
NH
1.98
1.00
1.98
1.00
0.85
2.04
3.89
6.28
4.63
9.5
9.0
8.5
8.0
7.5
7.0
6.5
6.0
5.5
5.0
4.5
4.0
3.5
3.0
2.5
2.0
1.5
1.0
0.5
f1 (ppm)

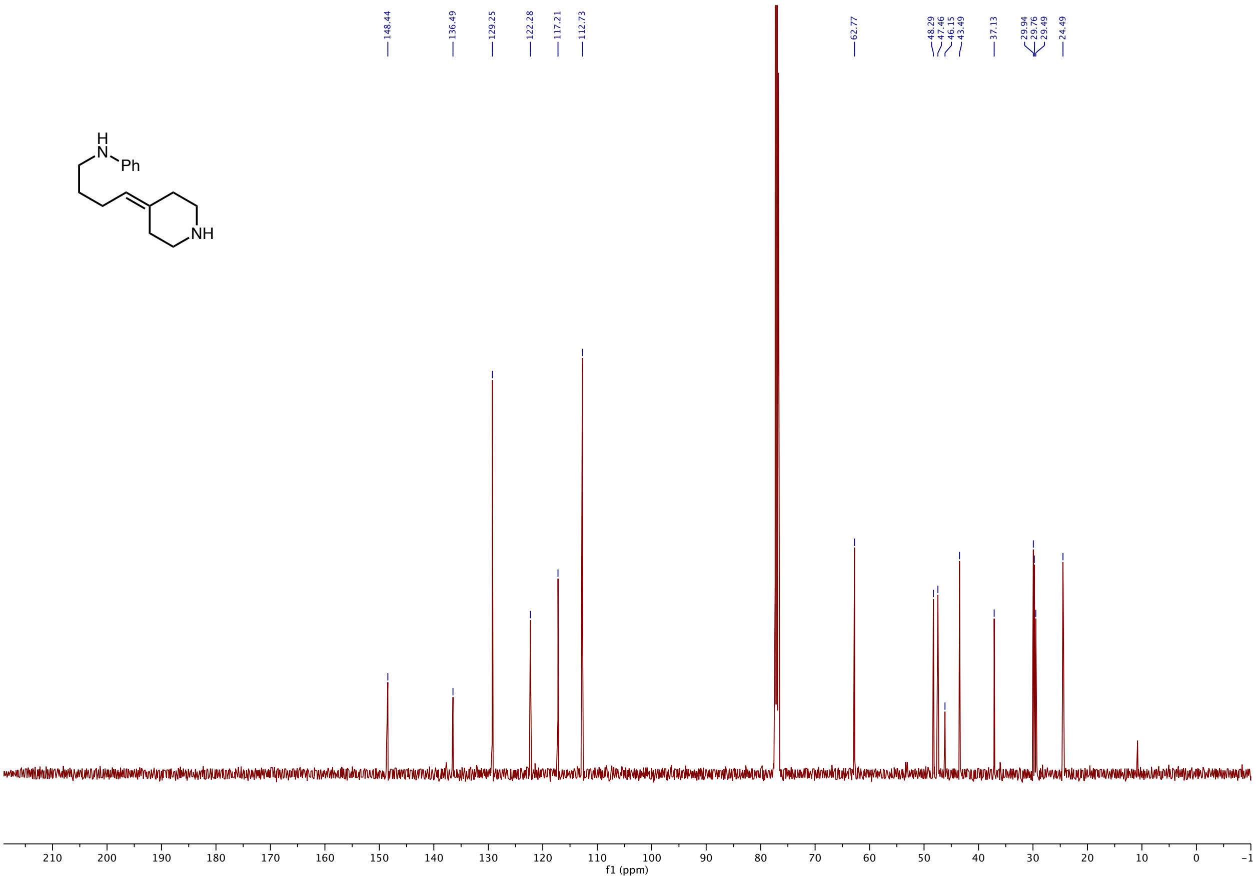

148.44
136.49
129.25
122.28
117.21
112.73
62.77
48.29
47.46
46.15
43.49
37.13
29.94
29.76
29.49
24.49
H
N
Ph
NH
210
200
190
180
170
160
150
140
130
120
110
100
90
80
70
60
50
40
30
20
10
0
f1 (ppm)

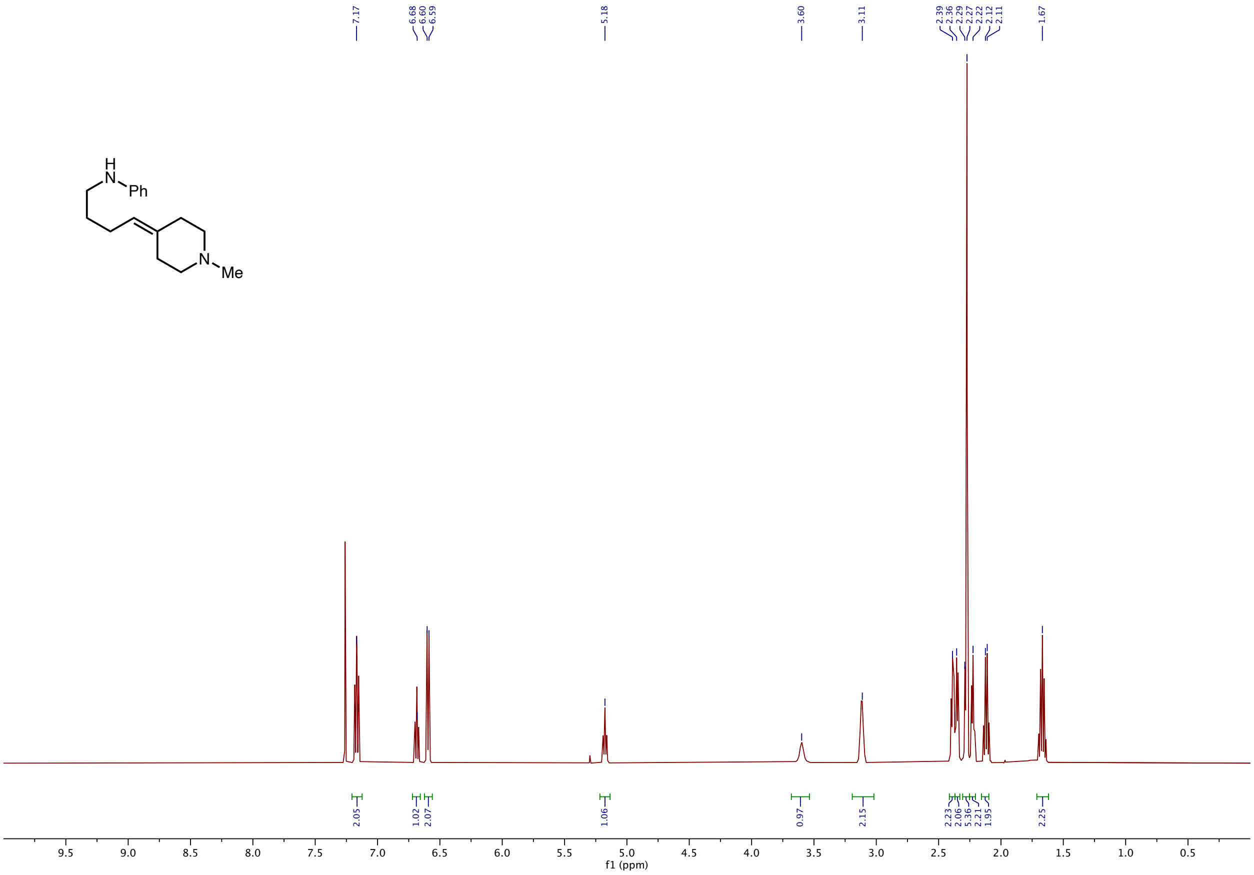

N
H
Ph
N
Me
7.17
6.68
6.60
6.59
5.18
3.60
3.11
2.39
2.36
2.29
2.27
2.22
2.12
2.11
1.67
2.05
1.02
2.07
1.06
0.97
2.15
2.23
2.06
5.36
2.21
1.95
2.25
f1 (ppm)

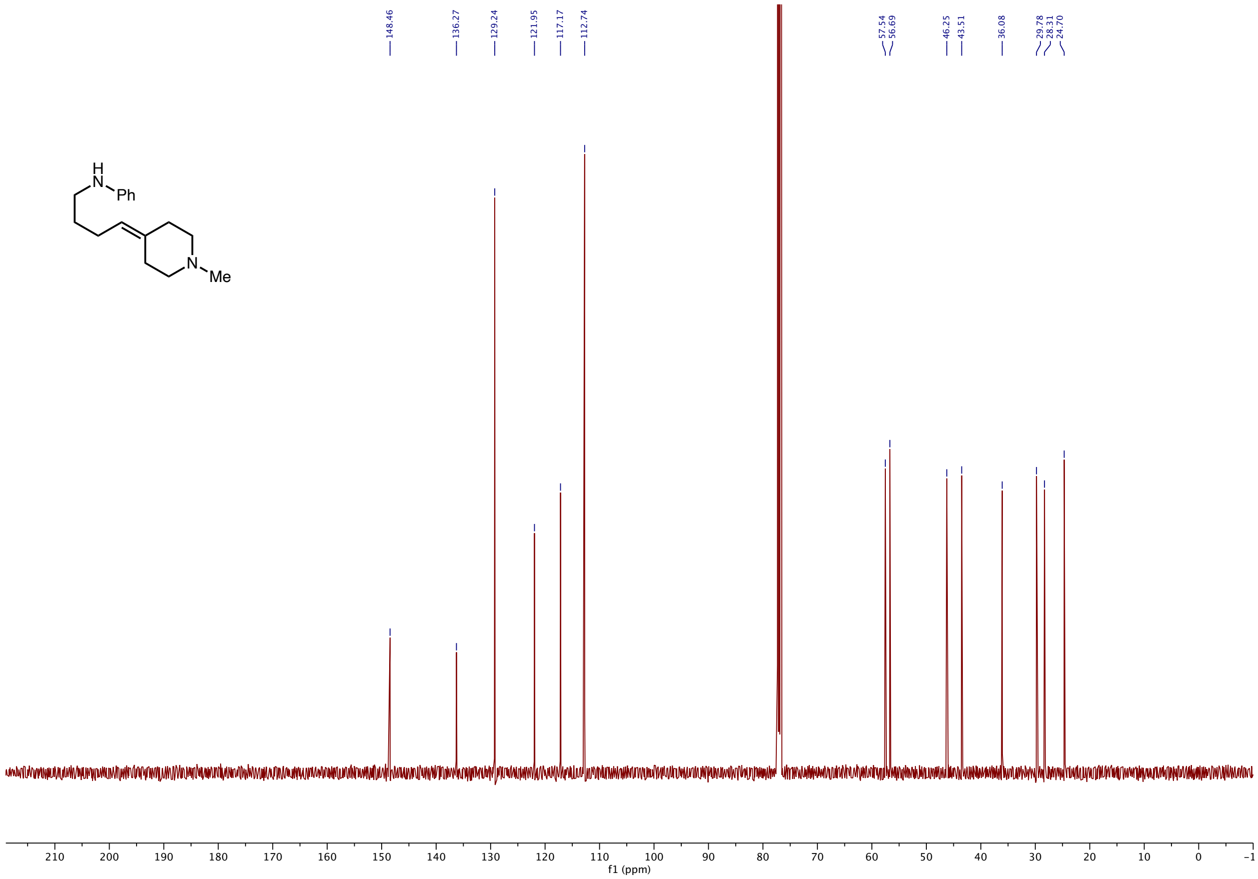

N
H
Ph
N
Me
148.46
136.27
129.24
121.95
117.17
112.74
57.54
56.69
46.25
43.51
36.08
29.78
28.31
24.70
f1 (ppm)

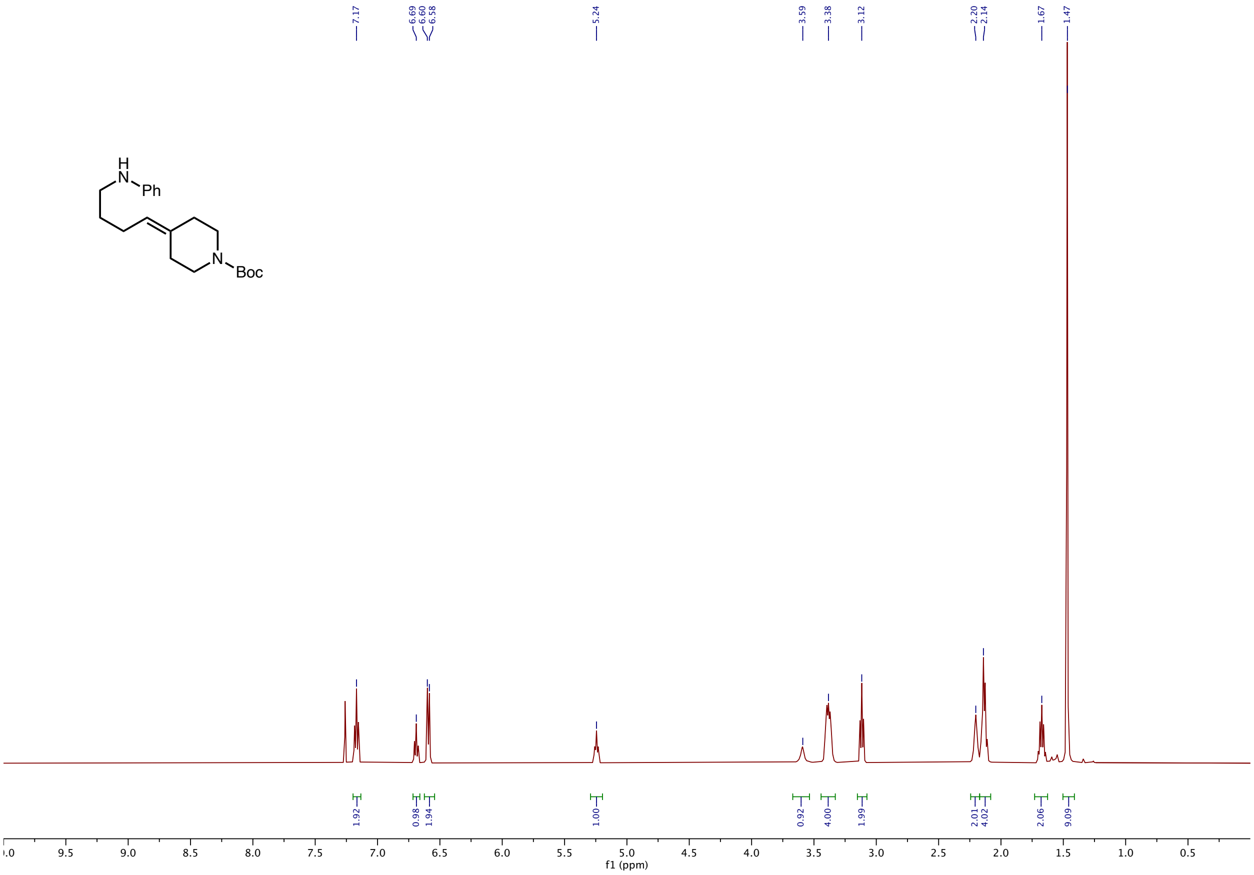

7.17
6.69
6.60
6.58
5.24
3.59
3.38
3.12
2.20
2.14
1.67
1.47
H
N
Ph
N
Boc
1.92
0.98
1.94
1.00
0.92
4.00
1.99
2.01
4.02
2.06
9.09
f1 (ppm)

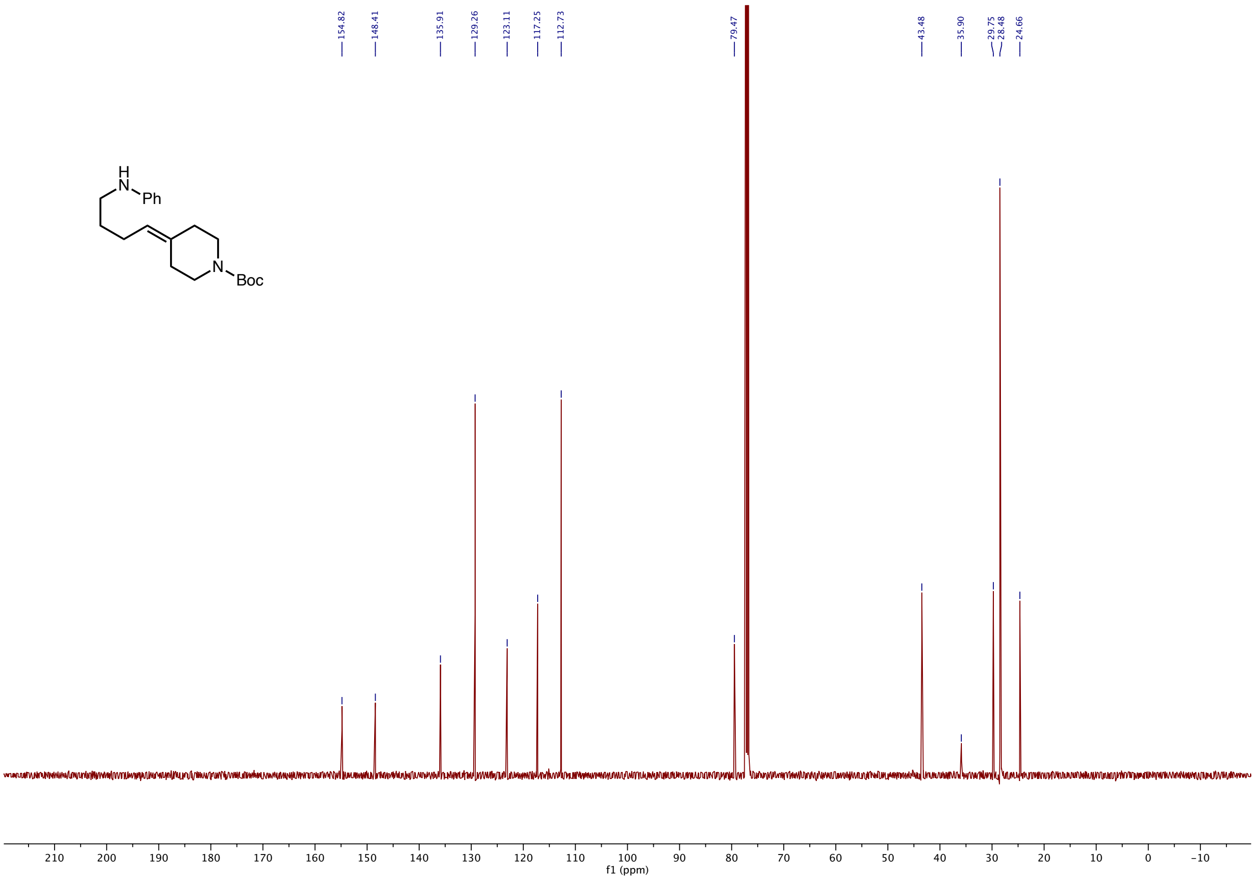

154.82
148.41
135.91
129.26
123.11
117.25
112.73
79.47
43.48
35.90
29.75
28.48
24.66
H
N
Ph
N
Boc
f1 (ppm)

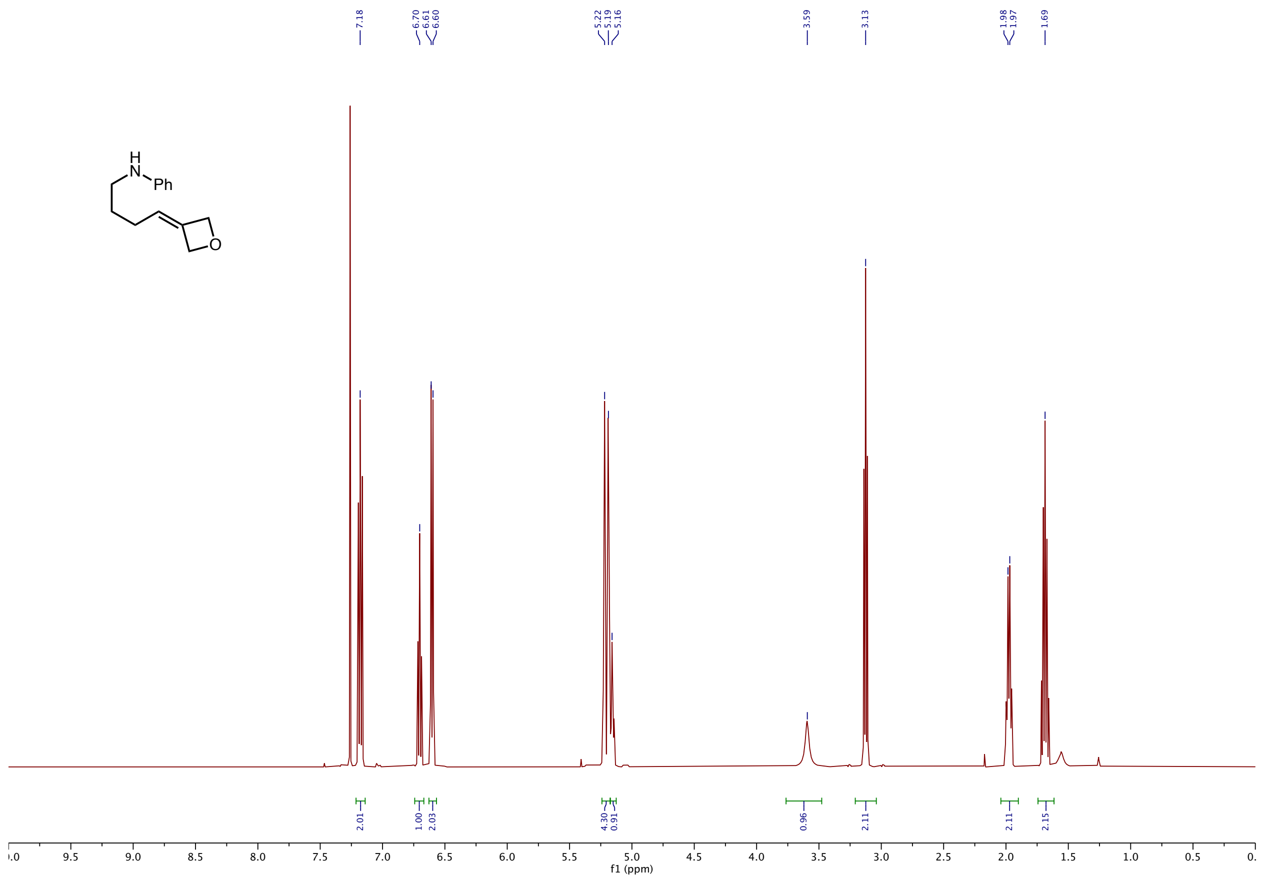

7.18
6.70
6.61
6.60
5.22
5.19
5.16
3.59
3.13
1.98
1.97
1.69
Ph
O
2.01
1.00
2.03
4.30
0.91
0.96
2.11
2.11
2.15
f1 (ppm)

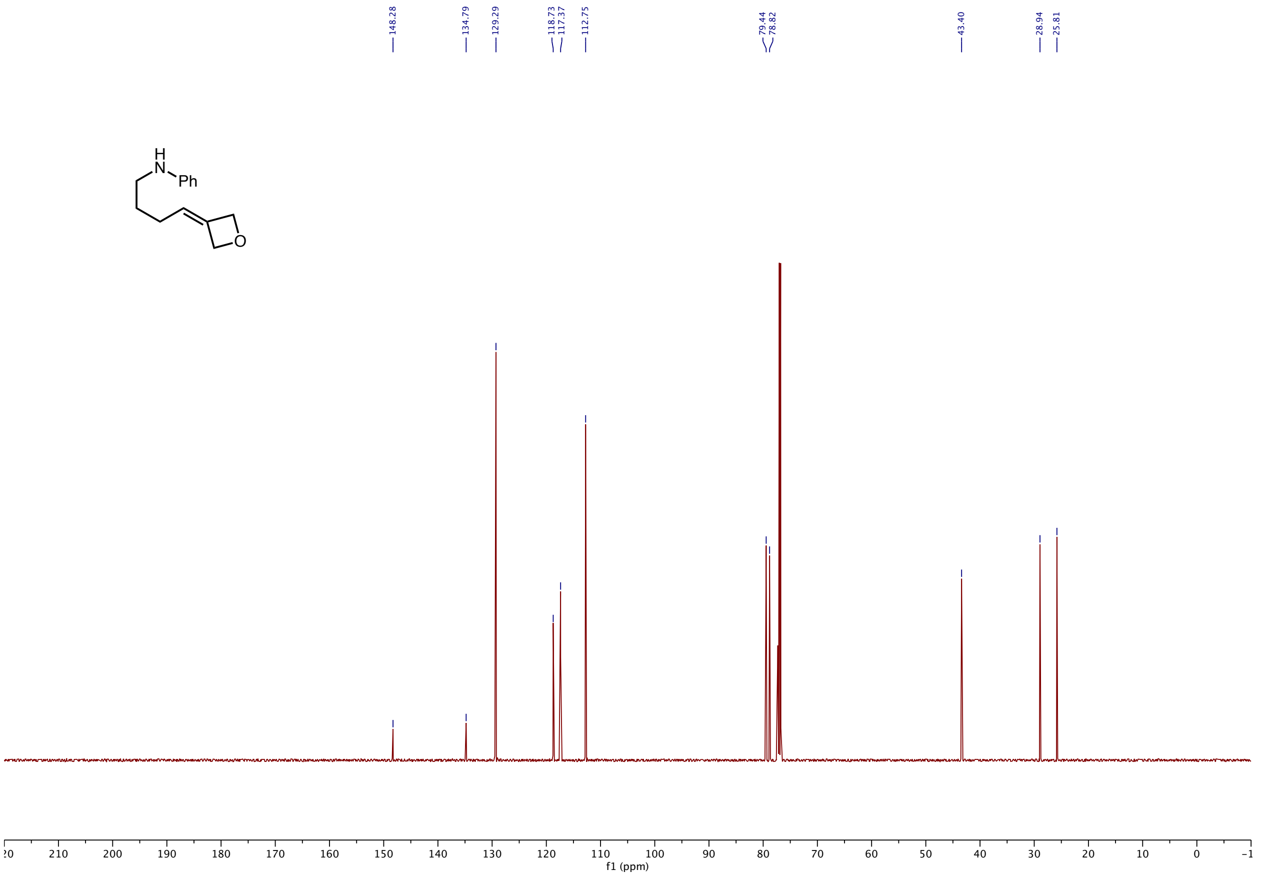

148.28
134.79
129.29
118.73
117.37
112.75
79.44
78.82
43.40
28.94
25.81
Ph
O
f1 (ppm)

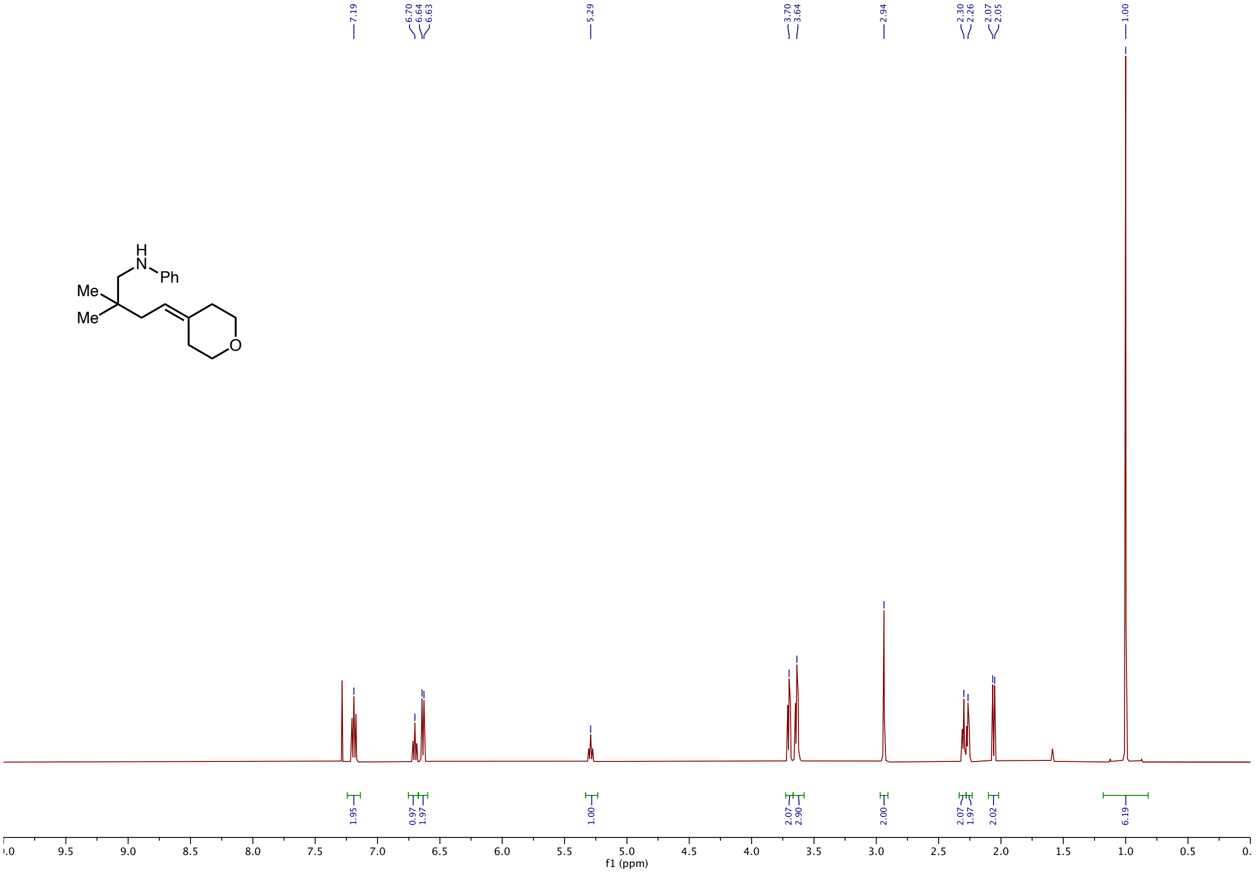
7.19
6.70
6.64
6.63
5.29
3.70
3.64
2.94
2.30
2.26
2.07
2.05
1.00
H
N
Ph
Me
Me
O
1.95
0.97
1.97
1.00
2.07
2.90
2.00
2.07
1.97
2.02
6.19
f1 (ppm)

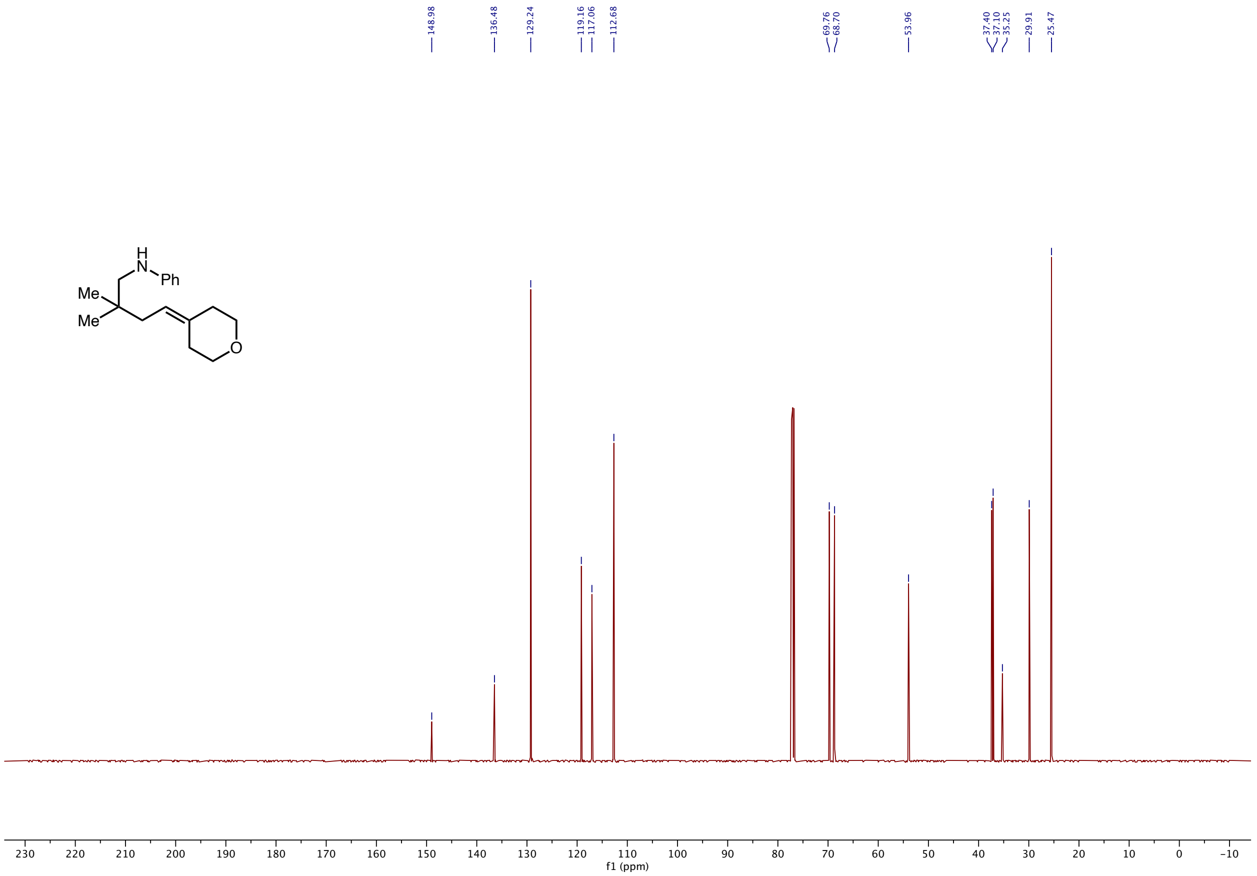
148.98
136.48
129.24
119.16
117.06
112.68
69.76
68.70
53.96
37.40
37.10
35.25
29.91
25.47
H
N
Ph
Me
Me
O
f1 (ppm)

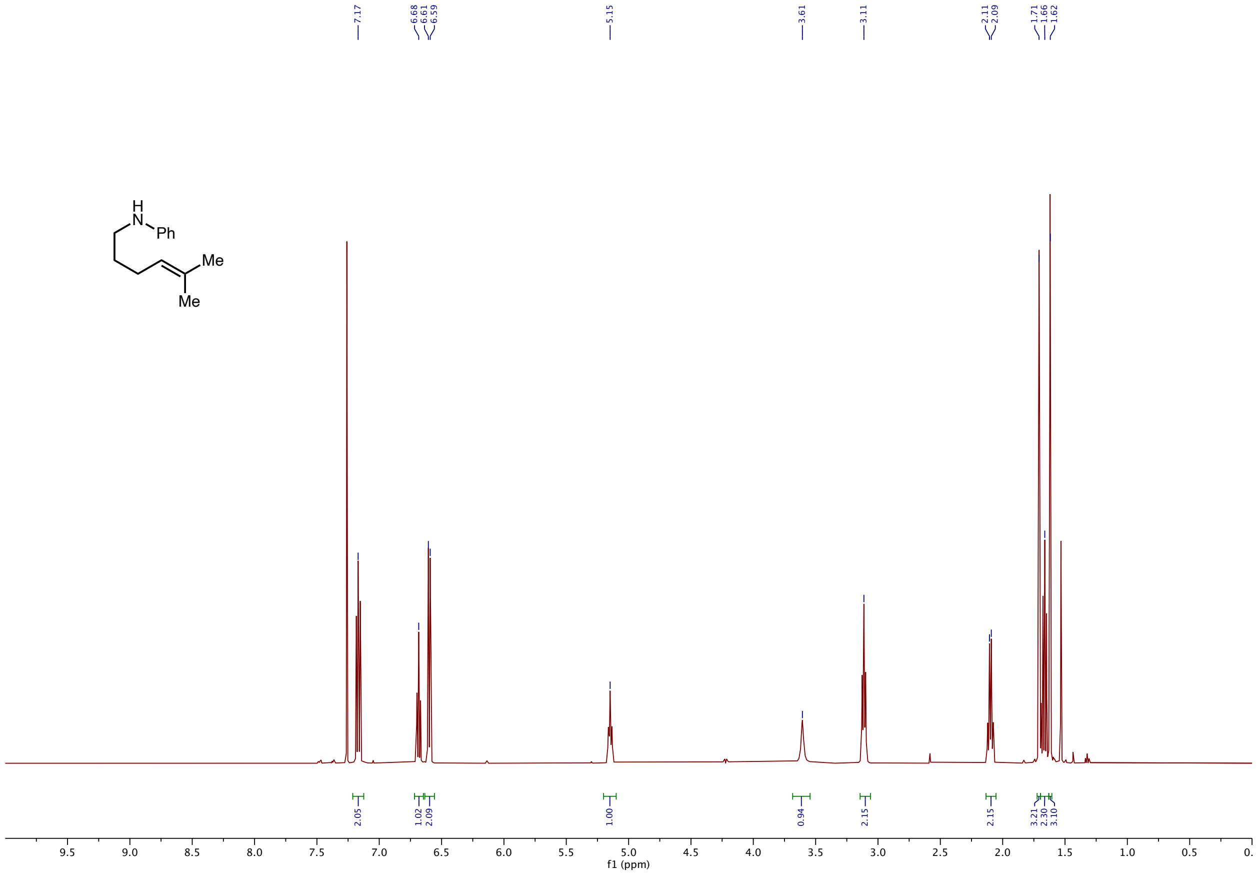

7.17
6.68
6.61
6.59
5.15
3.61
3.11
2.11
2.09
1.71
1.66
1.62
H
N
Ph
Me
Me
2.05
1.02
2.09
1.00
0.94
2.15
2.15
3.21
2.30
3.10
f1 (ppm)

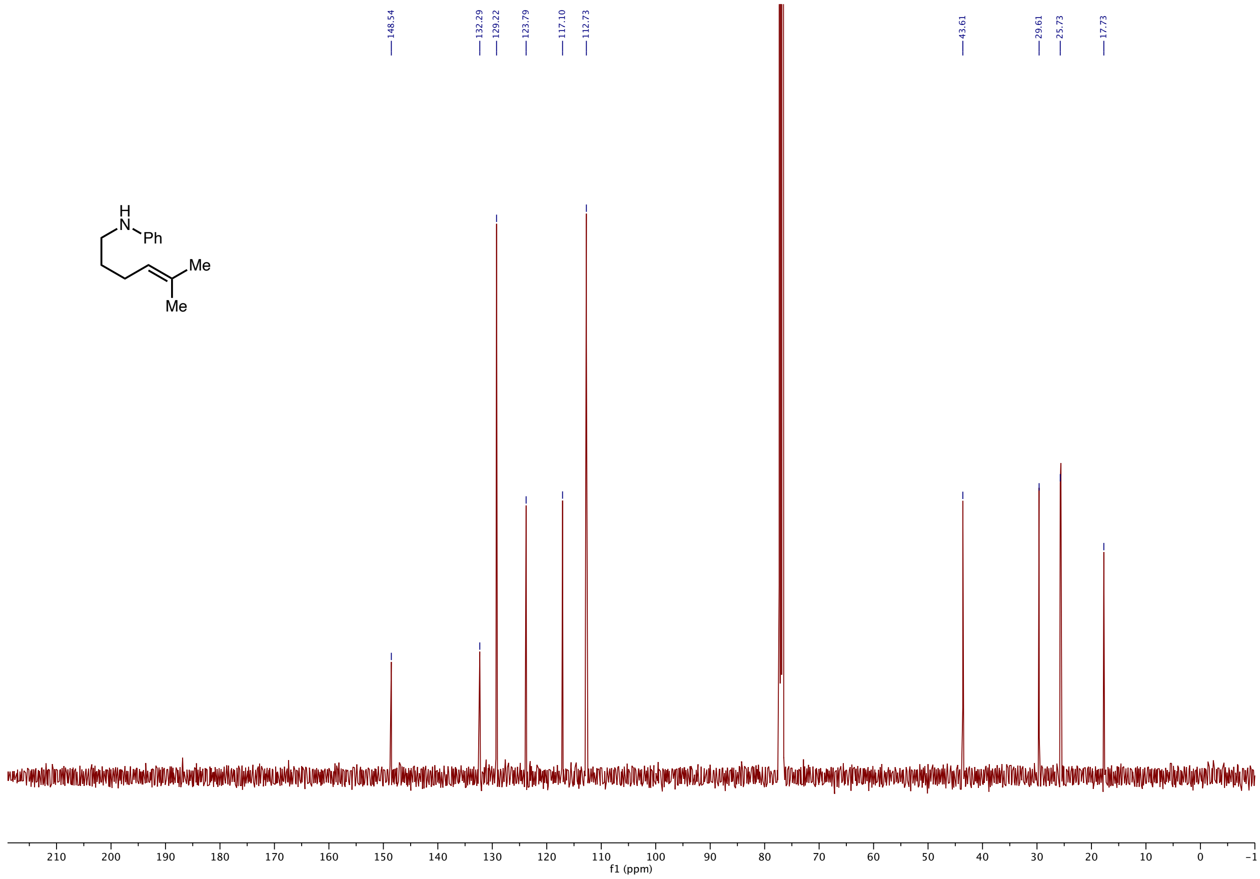

148.54
132.29
129.22
123.79
117.10
112.73
43.61
29.61
25.73
17.73
H
N
Ph
Me
Me
f1 (ppm)

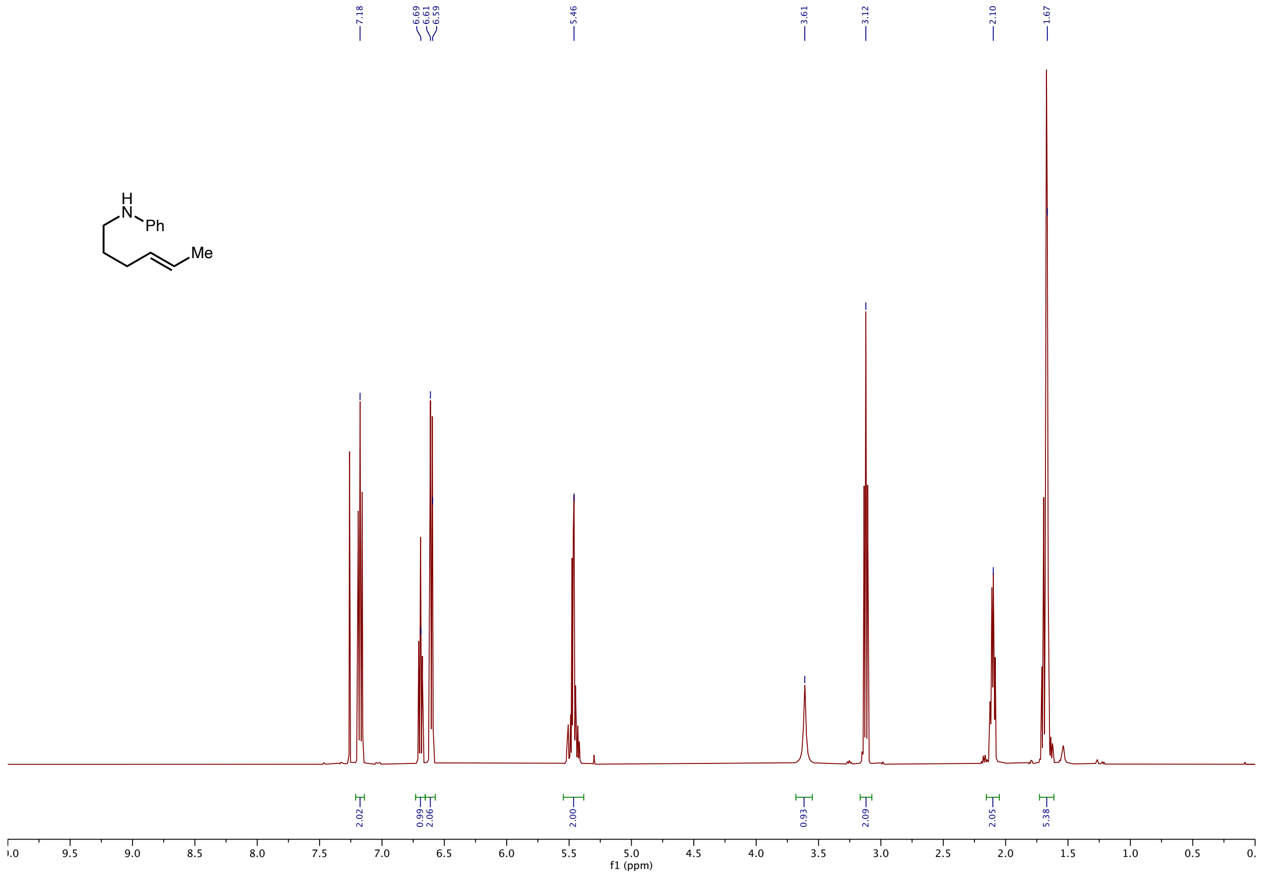
7.18
6.69
6.61
6.59
5.46
3.61
3.12
2.10
1.67
H
N
Ph
Me
2.02
0.99
2.06
2.00
0.93
2.09
2.05
5.38
f1 (ppm)

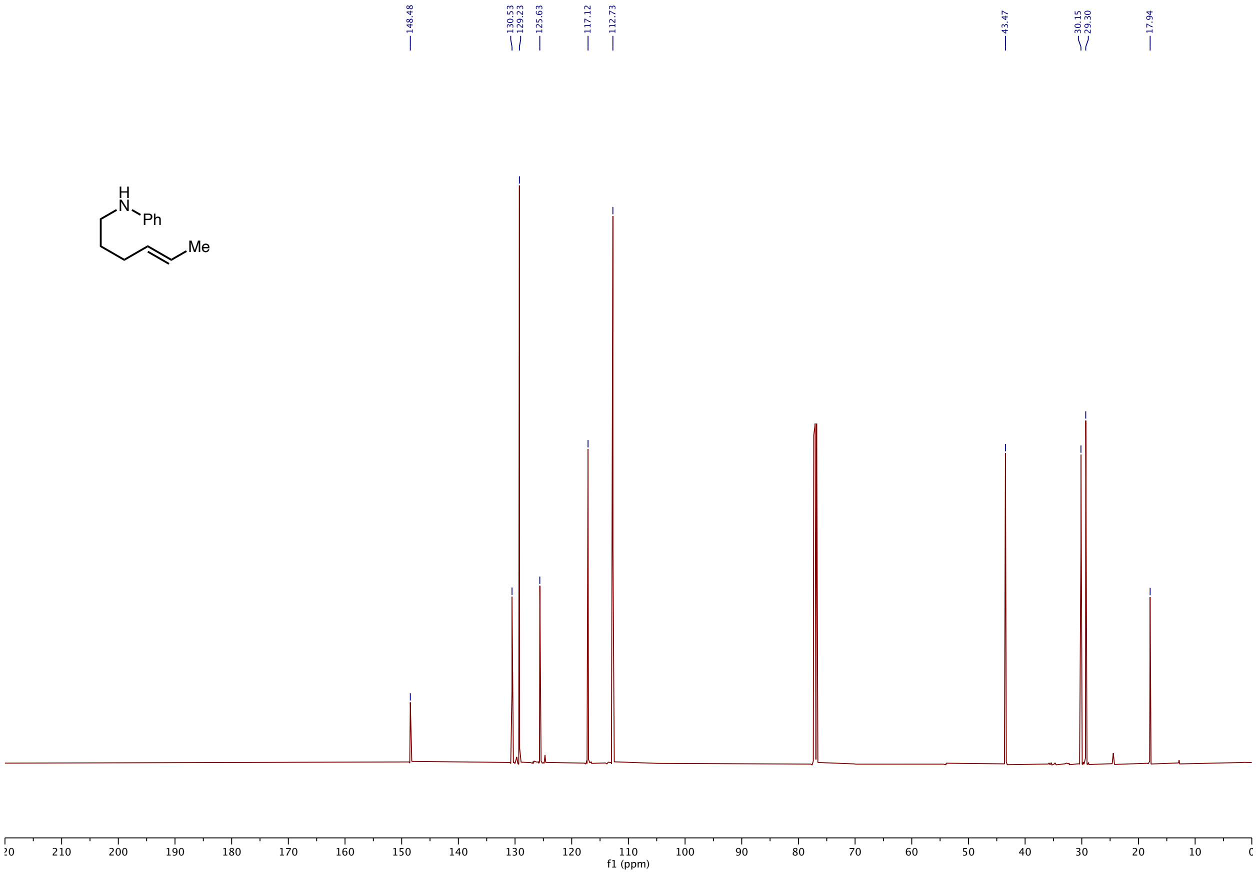
148.48
130.53
129.23
125.63
117.12
112.73
43.47
30.15
29.30
17.94
H
N
Ph
Me
f1 (ppm)

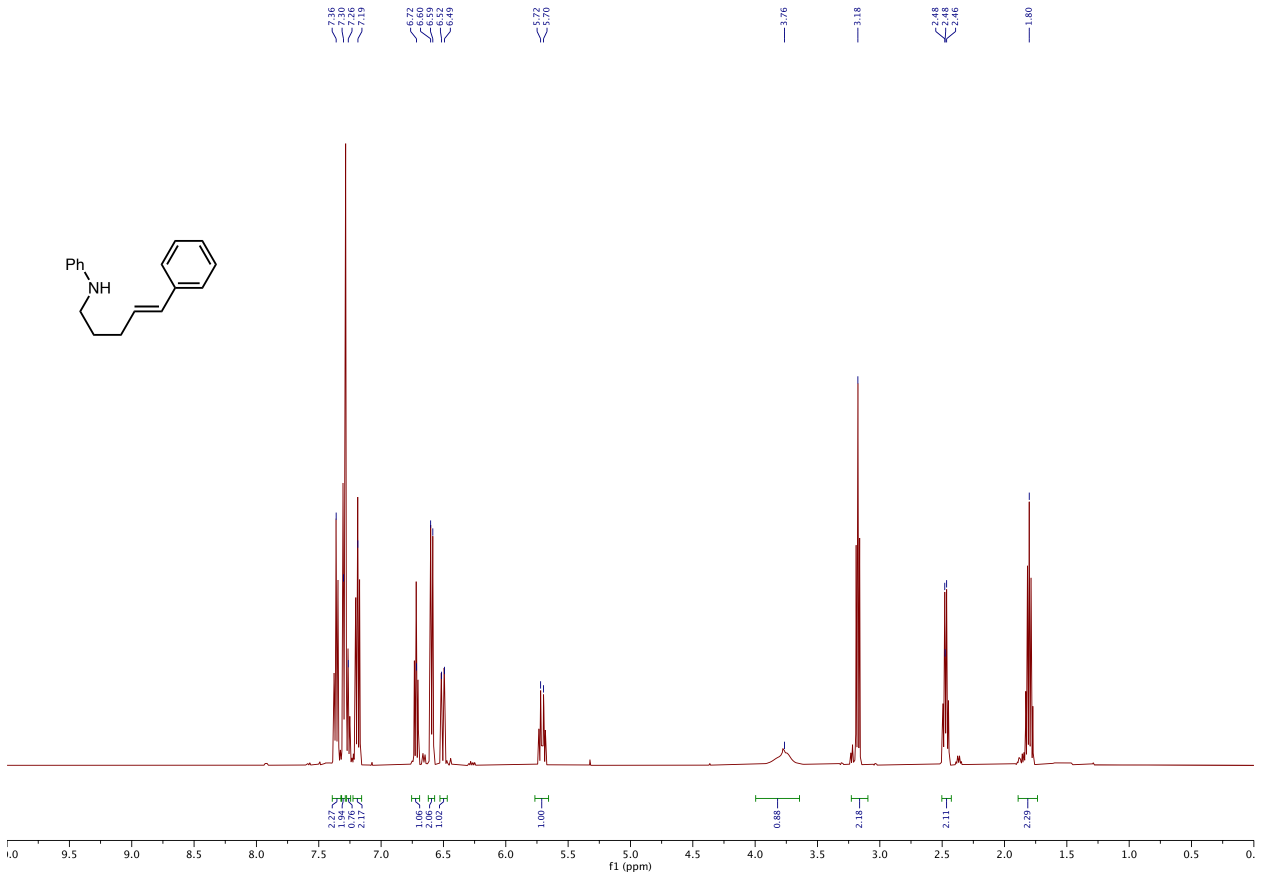

7.36
7.30
7.26
7.19
6.72
6.60
6.59
6.52
6.49
5.72
5.70
3.76
3.18
2.48
2.48
2.46
1.80
Ph
NH
2.27
1.94
0.76
2.17
1.06
2.06
1.02
1.00
0.88
2.18
2.11
2.29
f1 (ppm)

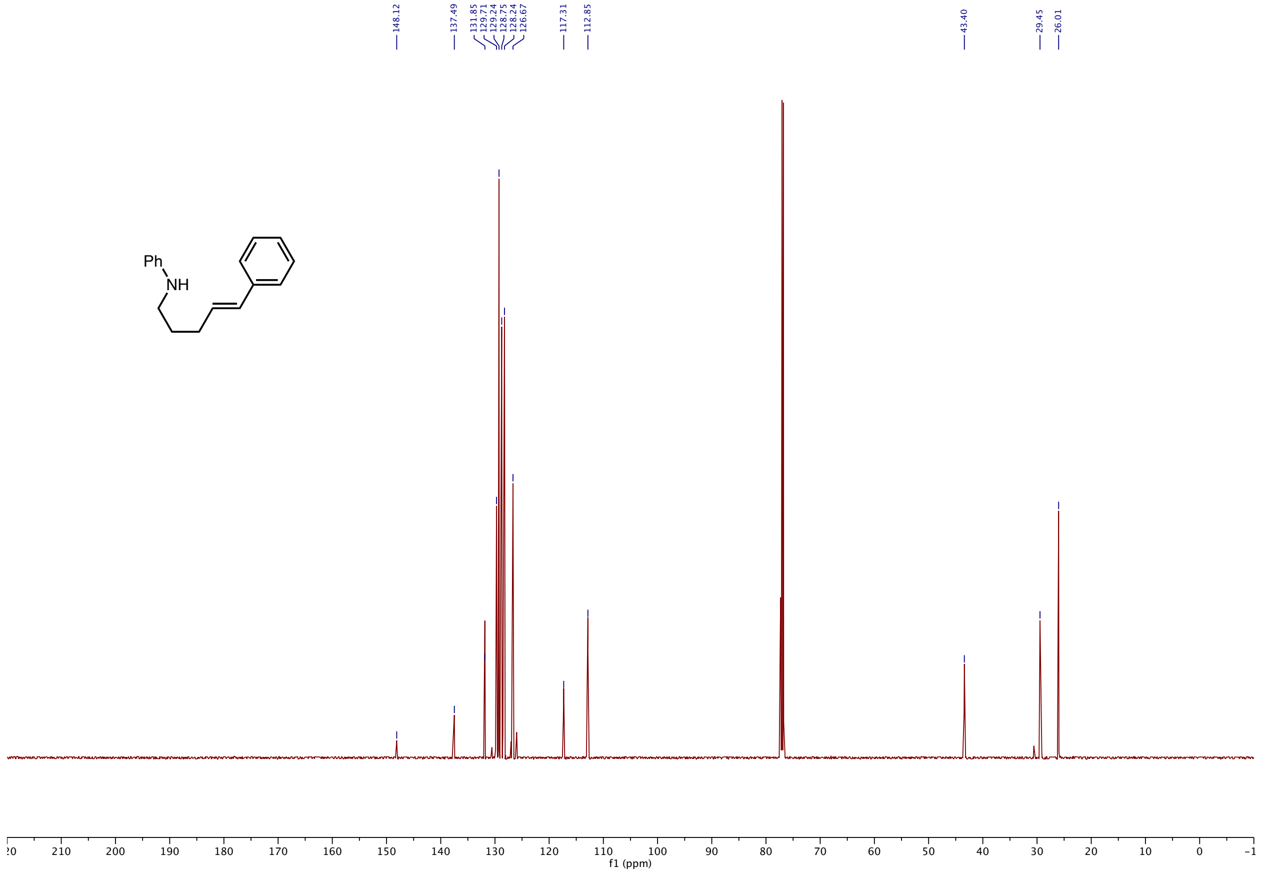

148.12
137.49
131.85
129.71
129.24
128.75
128.24
126.67
117.31
112.85
43.40
29.45
26.01
Ph
NH
f1 (ppm)

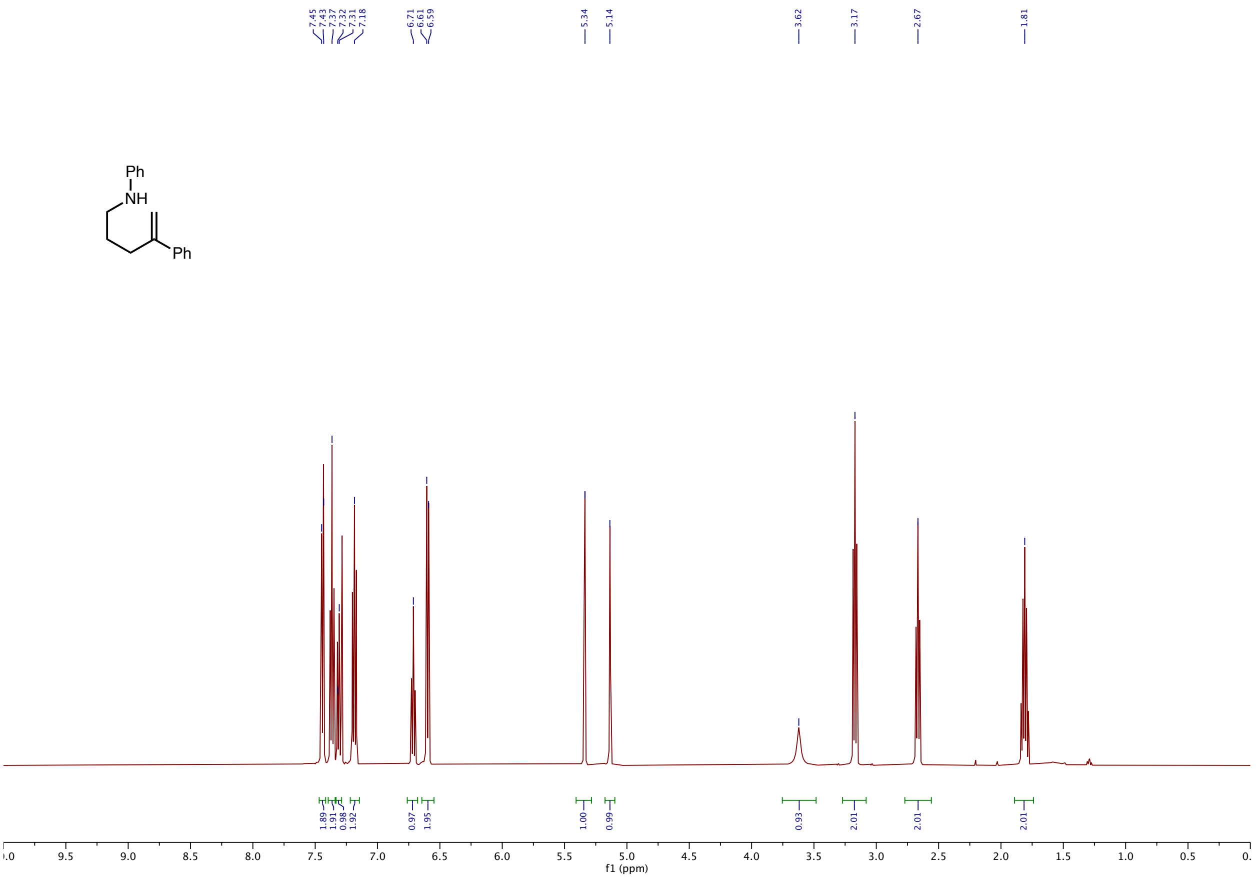

7.45
7.43
7.37
7.32
7.31
7.18
6.71
6.61
6.59
5.34
5.14
3.62
3.17
2.67
1.81
Ph
NH
Ph
1.89
1.91
0.98
1.92
0.97
1.95
1.00
0.99
0.93
2.01
2.01
2.01
f1 (ppm)

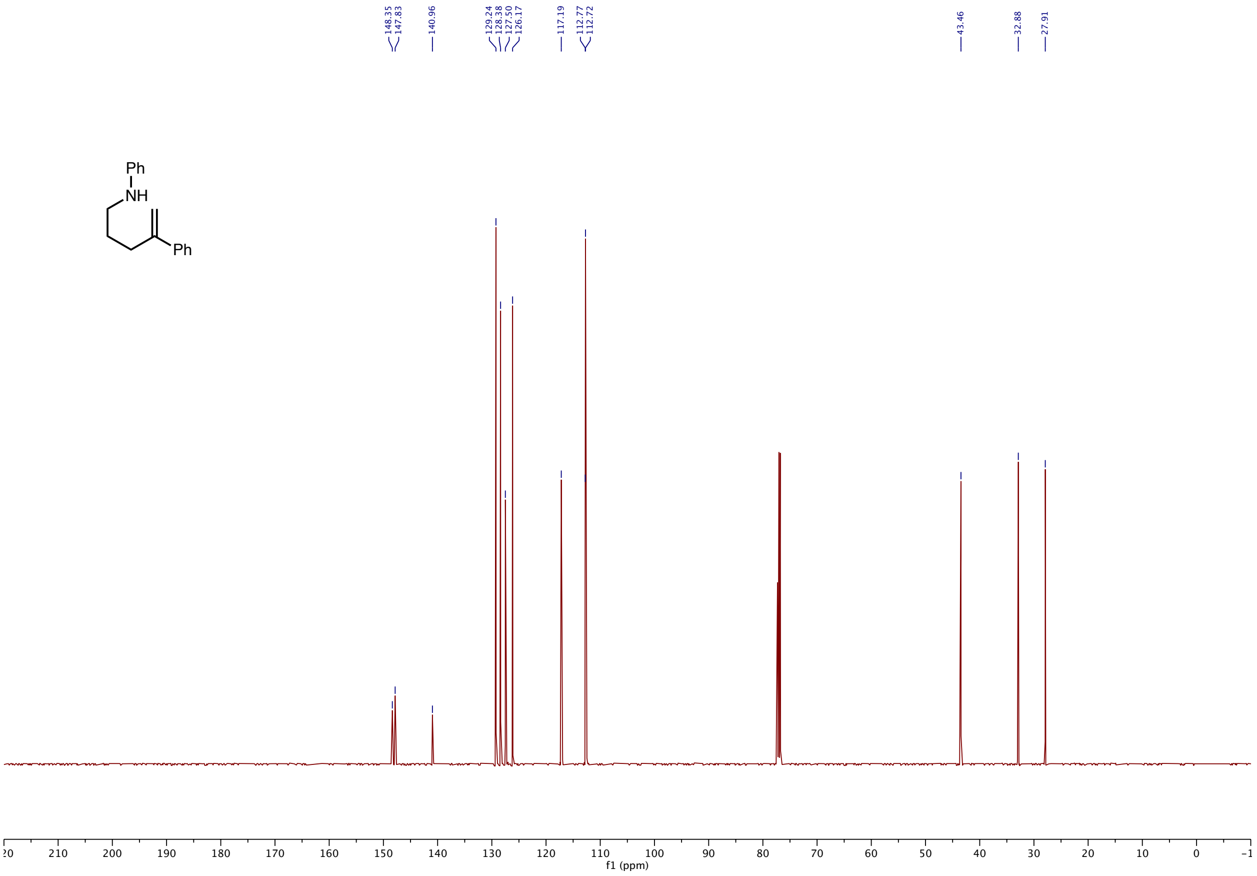

148.35
147.83
140.96
129.24
128.38
127.50
126.17
117.19
112.77
112.72
43.46
32.88
27.91
Ph
NH
Ph
f1 (ppm)

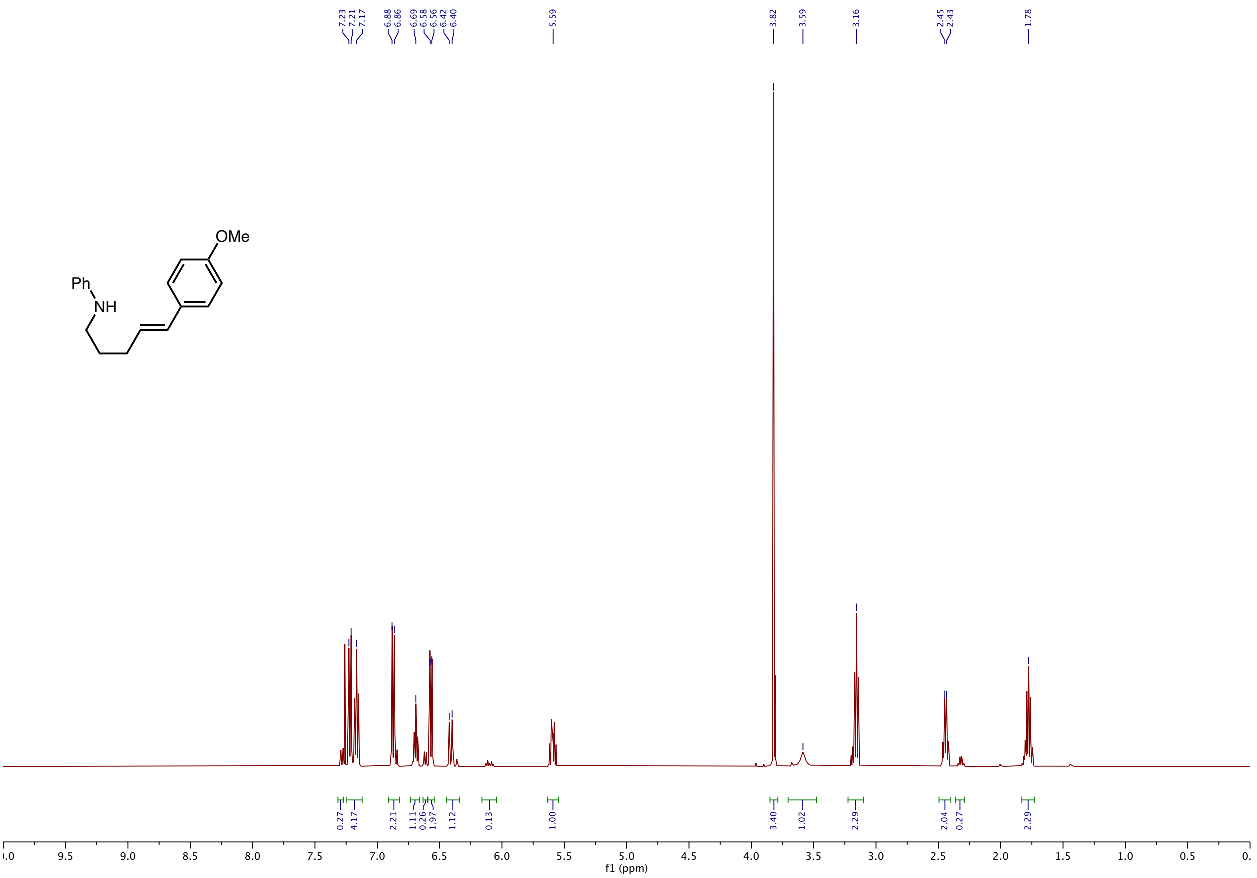

7.23
7.21
7.17
6.88
6.86
6.69
6.58
6.56
6.42
6.40
5.59
3.82
3.59
3.16
2.45
2.43
1.78
OMe
Ph
NH
0.27
4.17
2.21
1.11
0.26
1.97
1.12
0.13
1.00
3.40
1.02
2.29
2.04
0.27
2.29
f1 (ppm)

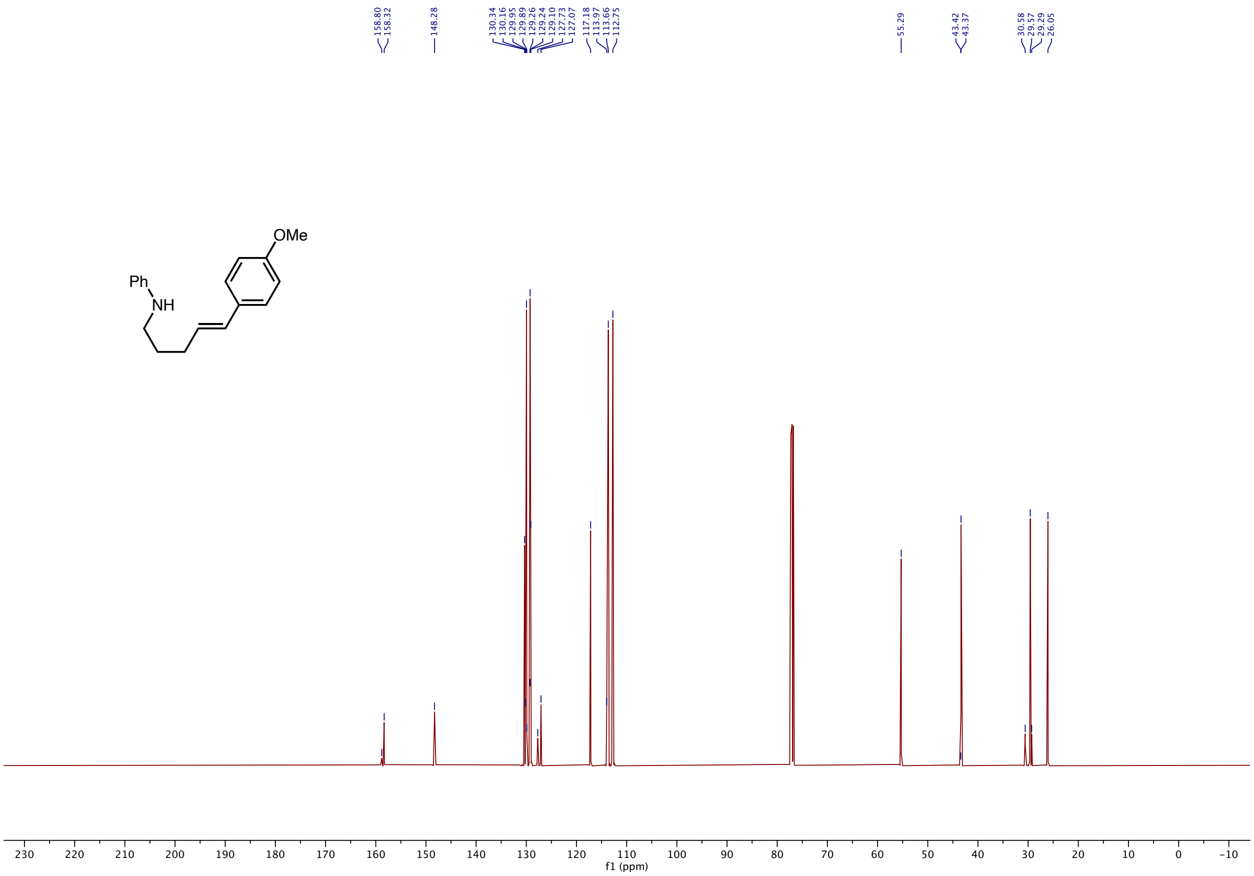

158.80
158.32
148.28
130.34
130.16
129.95
129.89
129.26
129.24
129.10
127.73
127.07
117.18
113.97
113.66
112.75
55.29
43.42
43.37
30.58
29.57
29.29
26.05
OMe
Ph
NH
f1 (ppm)

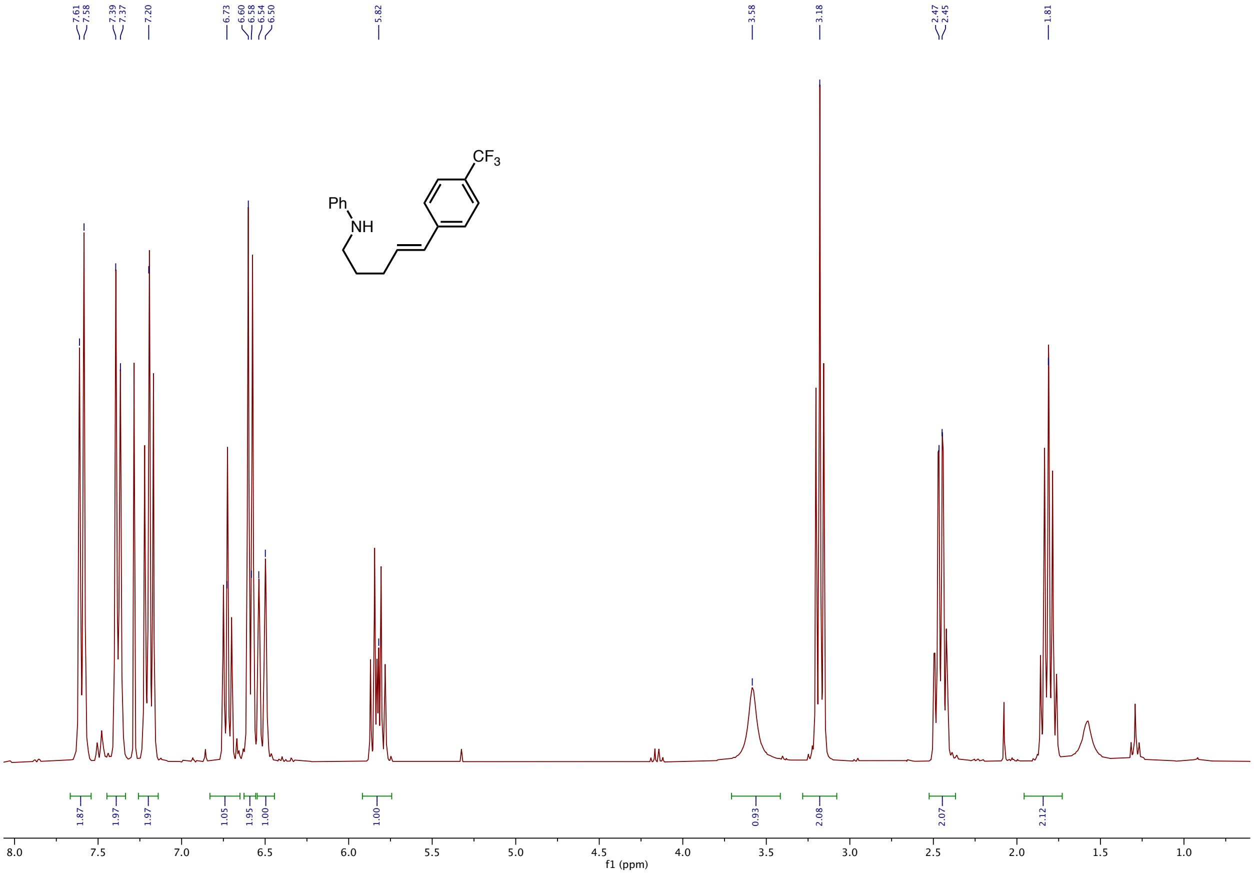

7.61
7.58
7.39
7.37
7.20
6.73
6.60
6.58
6.54
6.50
5.82
3.58
3.18
2.47
2.45
1.81
CF3
Ph
NH
1.87
1.97
1.97
1.05
1.95
1.00
1.00
0.93
2.08
2.07
2.12
f1 (ppm)

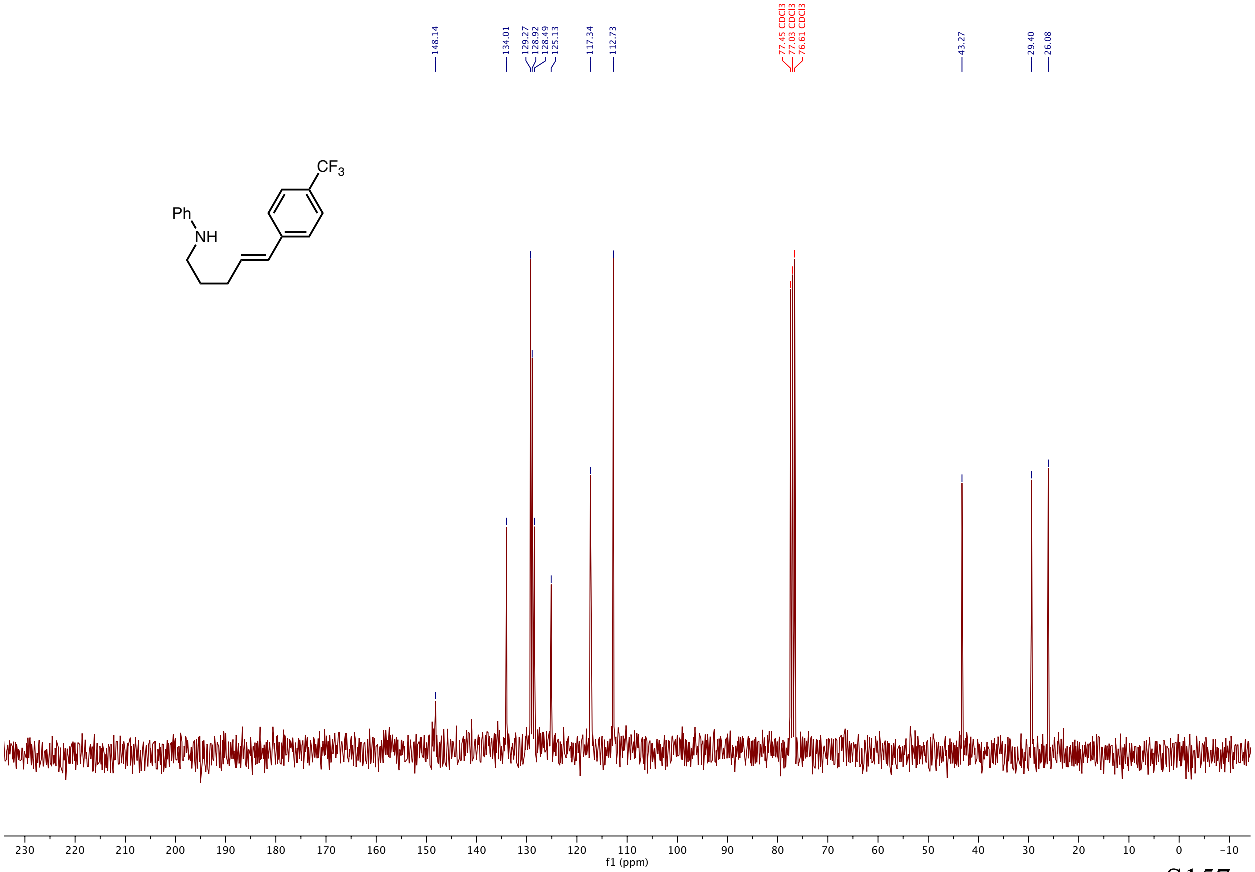

148.14
134.01
129.27
128.92
128.49
125.13
117.34
112.73
77.45 CDCl3
77.03 CDCl3
76.61 CDCl3
43.27
29.40
26.08
CF3
Ph
NH
f1 (ppm)

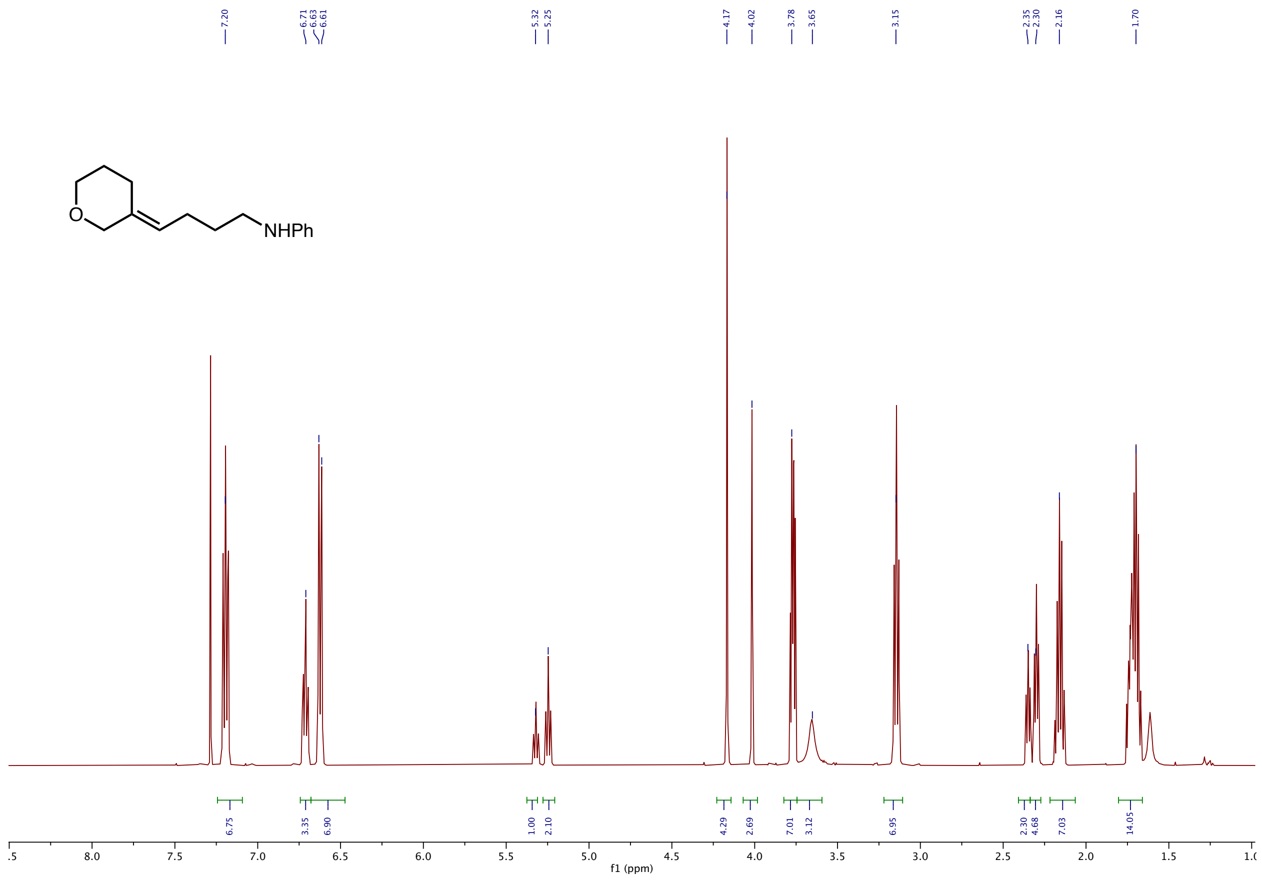
7.20
6.71
6.63
6.61
5.32
5.25
4.17
4.02
3.78
3.65
3.15
2.35
2.30
2.16
1.70
NHPh
O
6.75
3.35
6.90
1.00
2.10
4.29
2.69
7.01
3.12
6.95
2.30
4.68
7.03
14.05
f1 (ppm)

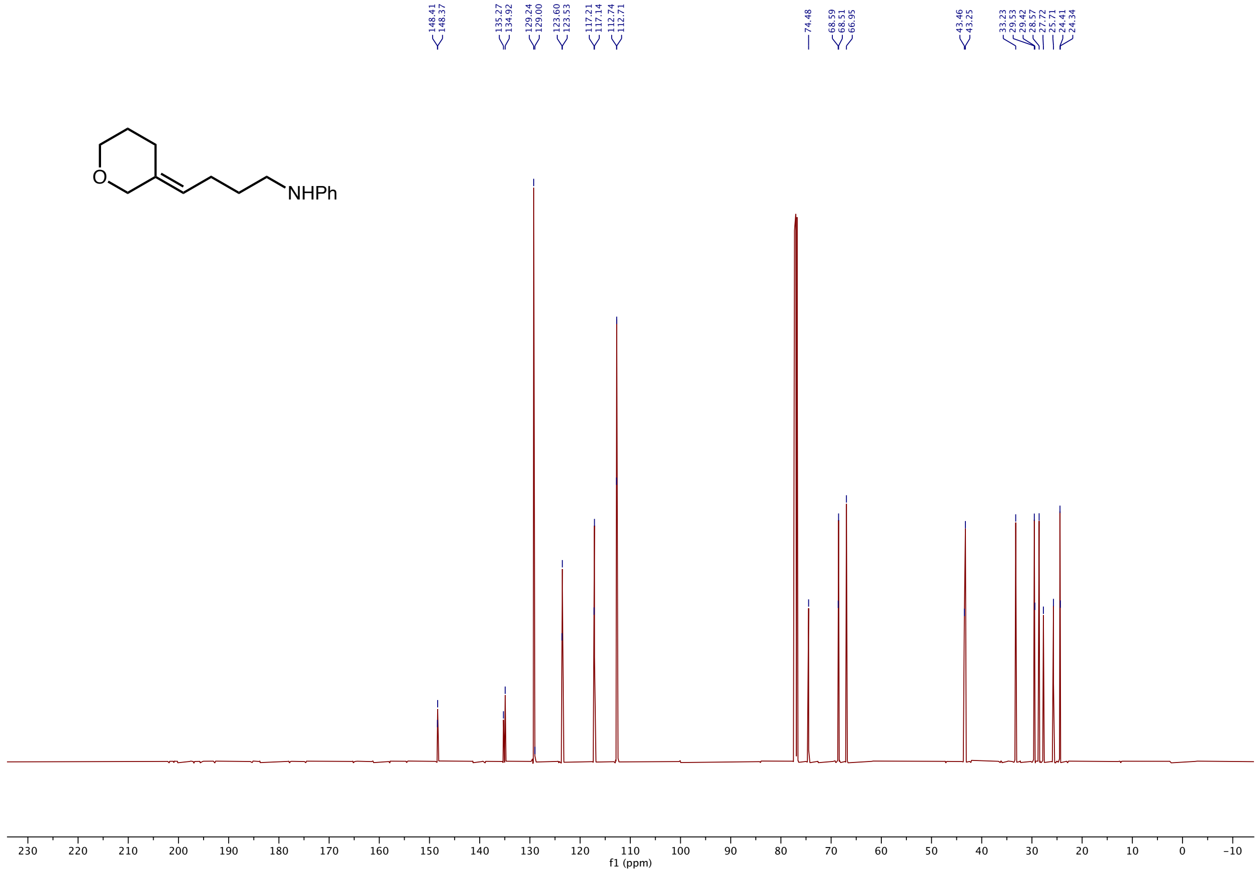
148.41
148.37
135.27
134.92
129.24
129.00
123.60
123.53
117.21
117.14
112.74
112.71
74.48
68.59
68.51
66.95
43.46
43.25
33.23
29.53
29.42
28.57
27.72
25.71
24.41
24.34
NHPh
O
f1 (ppm)

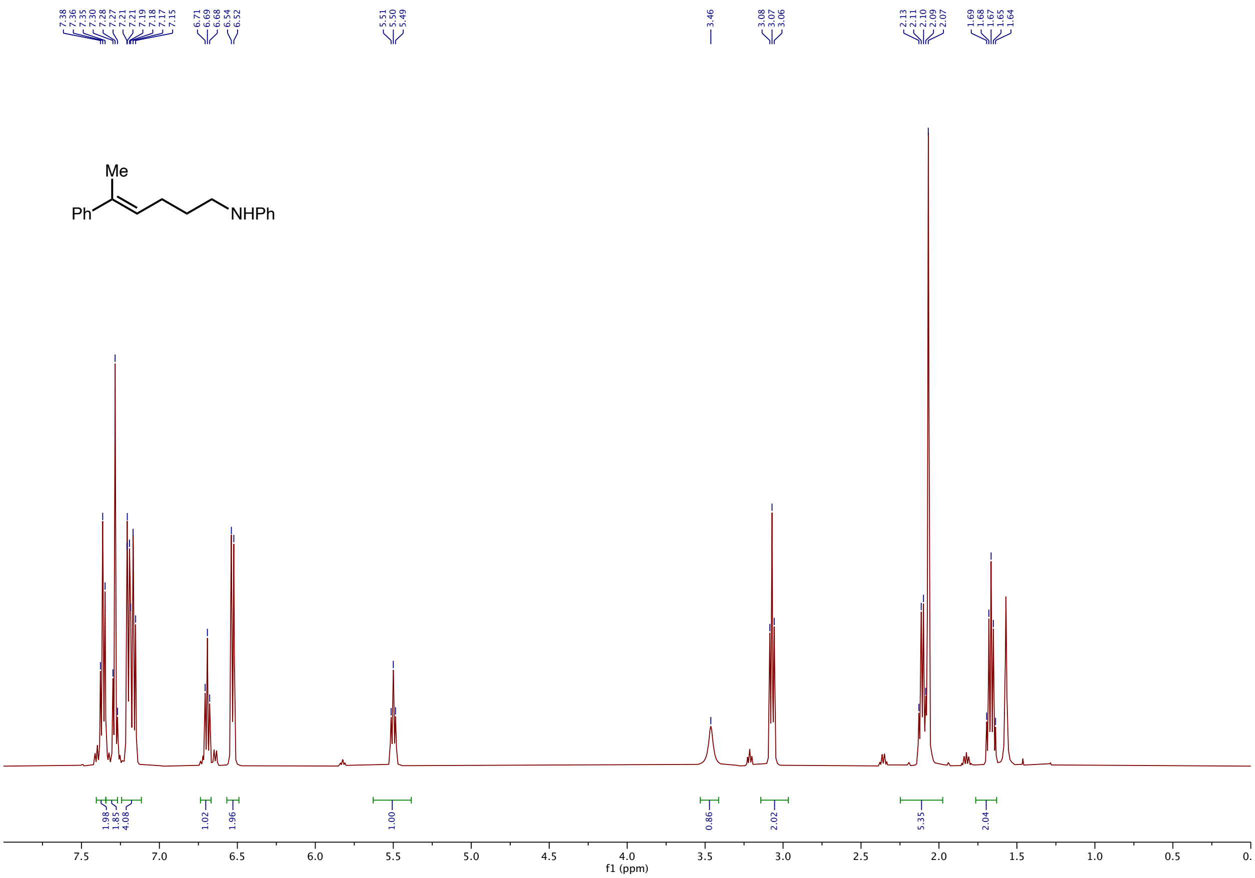

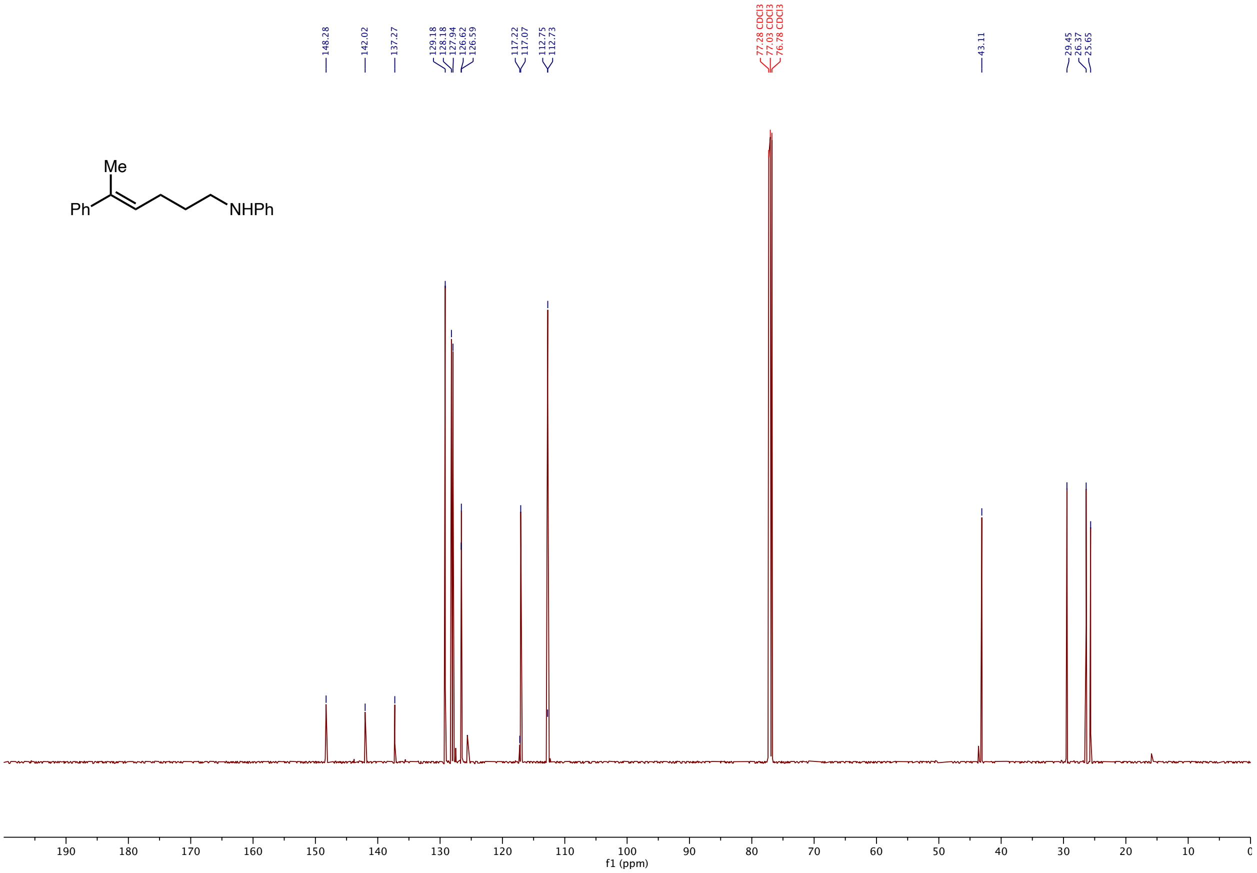

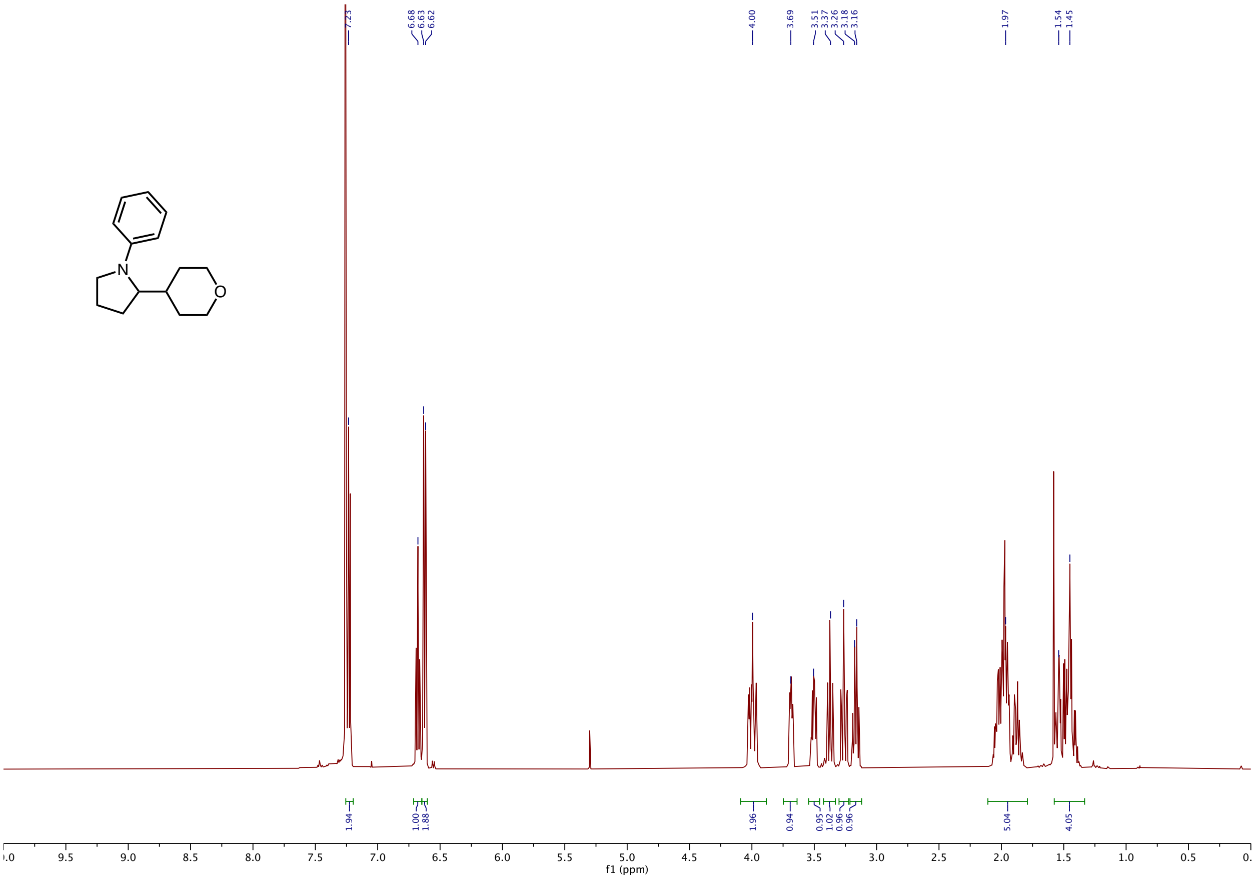
7.23
6.68
6.63
6.62
4.00
3.69
3.51
3.37
3.26
3.18
3.16
1.97
1.54
1.45
1.94
1.00
1.88
1.96
0.94
0.95
1.02
0.96
0.96
5.04
4.05
f1 (ppm)

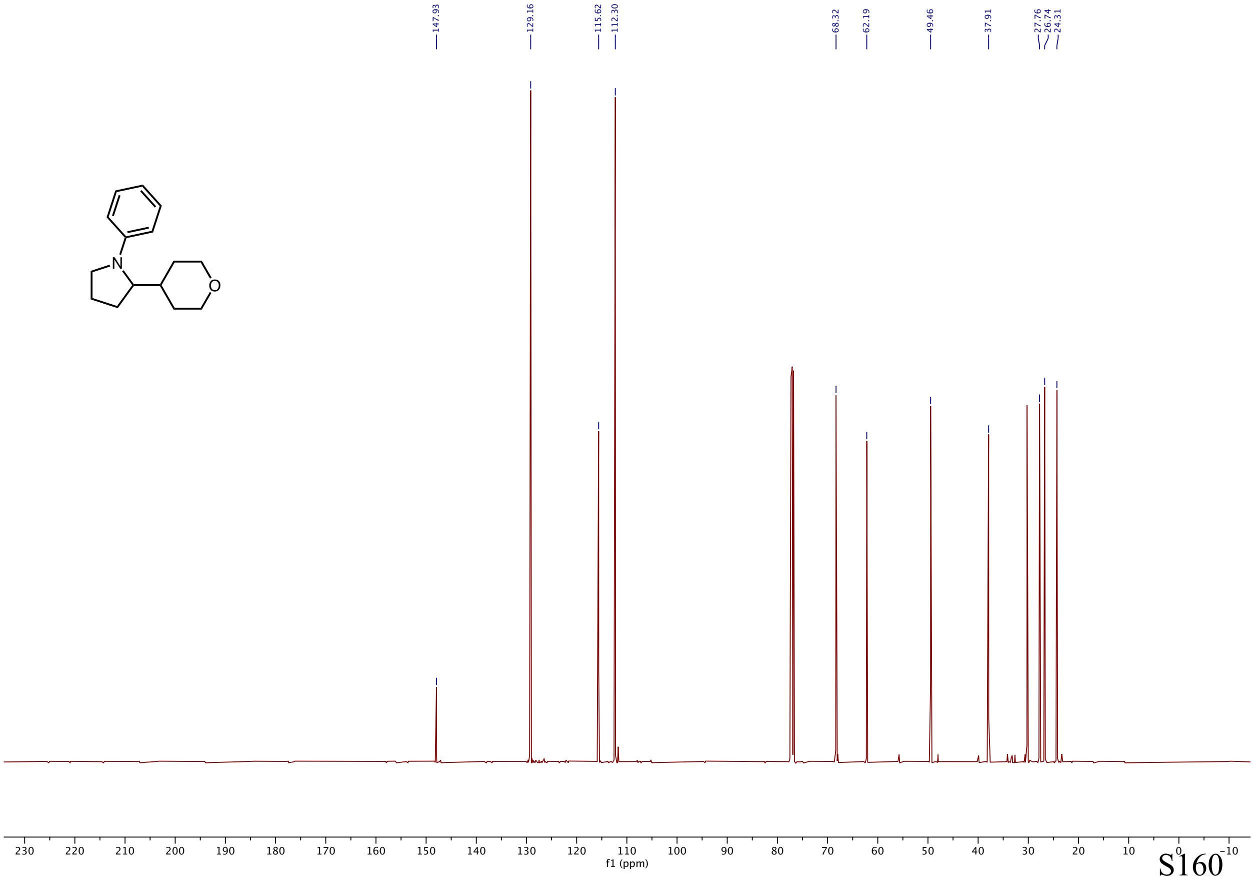
147.93
129.16
115.62
112.30
68.32
62.19
49.46
37.91
27.76
26.74
24.31
f1 (ppm)

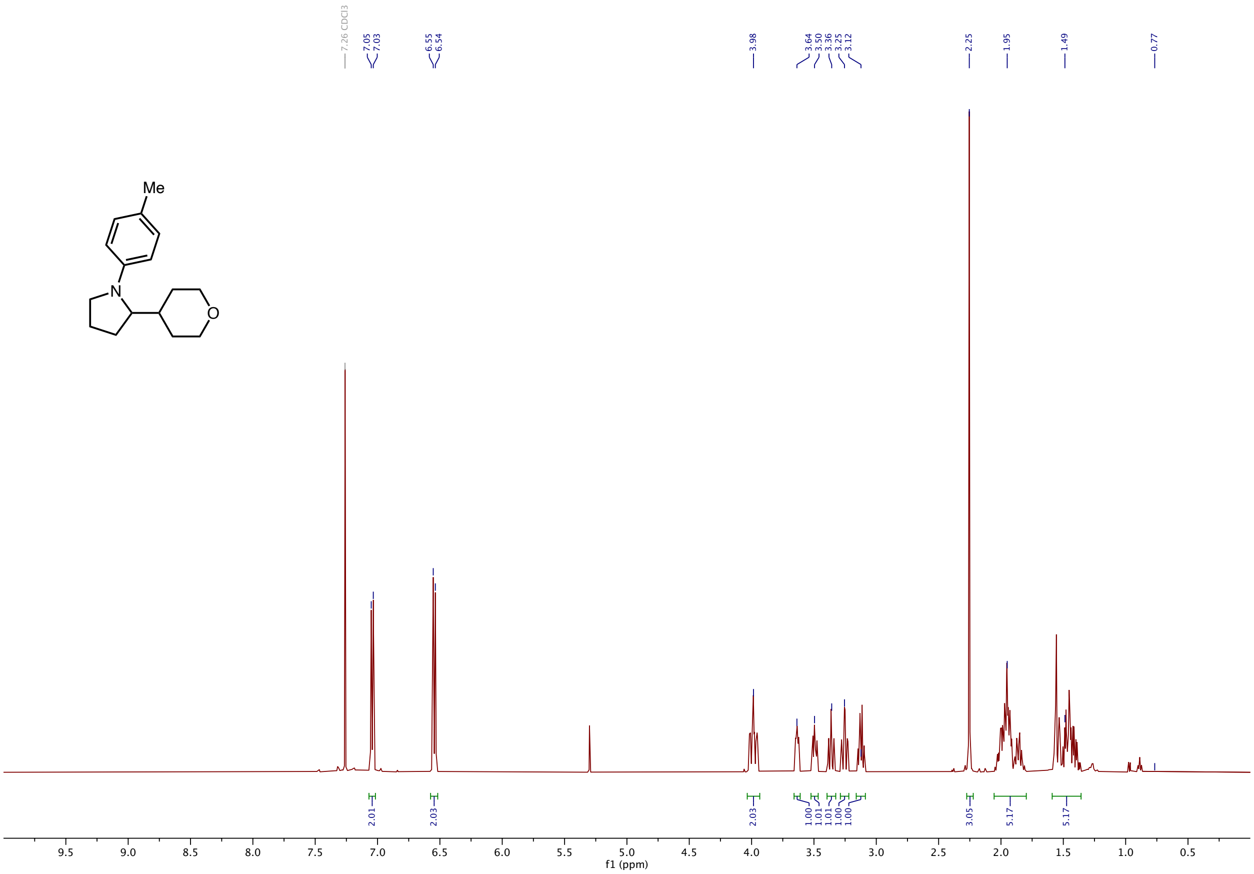
7.26 CDCl3
7.05
7.03
6.55
6.54
3.98
3.64
3.50
3.36
3.25
3.12
2.25
1.95
1.49
0.77
Me
N
O
2.01
2.03
2.03
1.00
1.01
1.01
1.00
1.00
3.05
5.17
5.17
f1 (ppm)

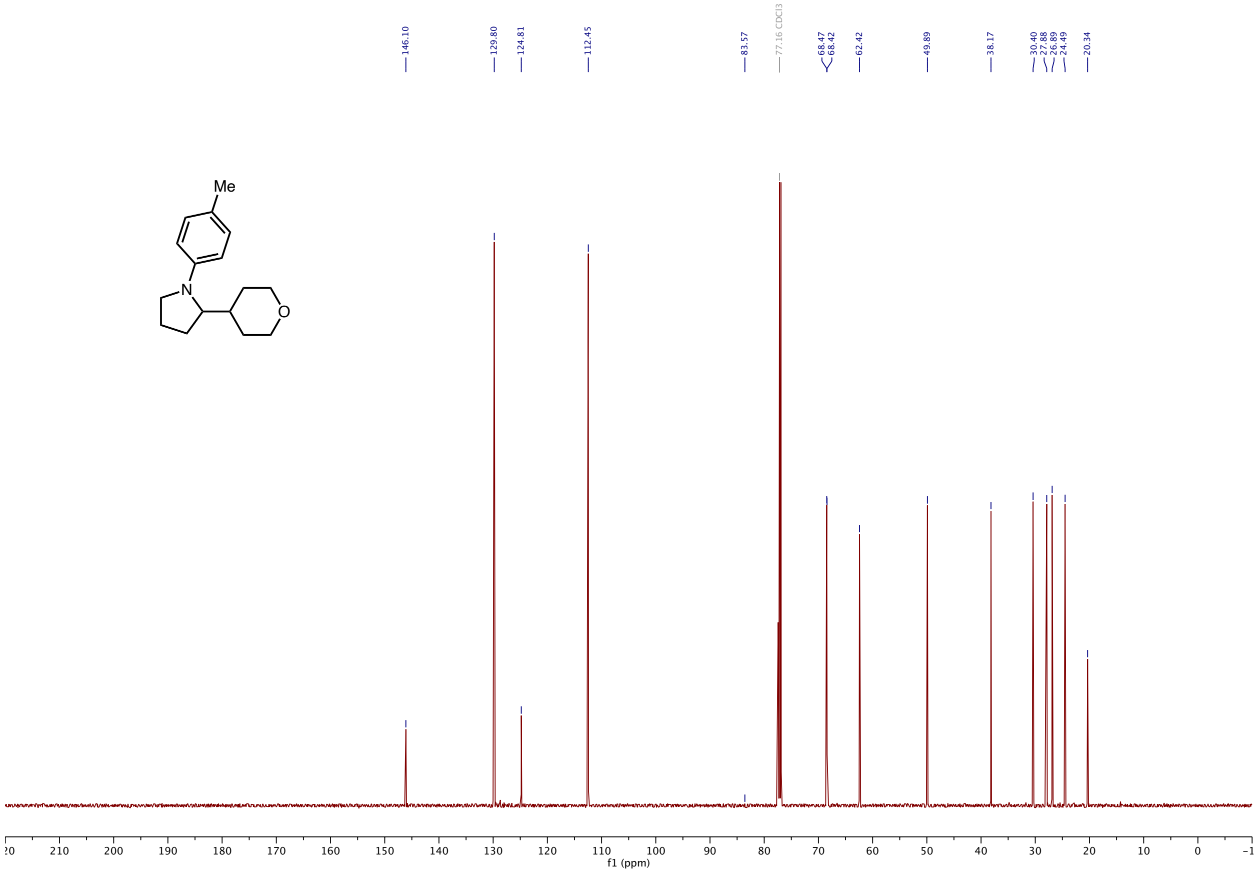
146.10
129.80
124.81
112.45
83.57
77.16 CDCl3
68.47
68.42
62.42
49.89
38.17
30.40
27.88
26.89
24.49
20.34
Me
N
O
f1 (ppm)

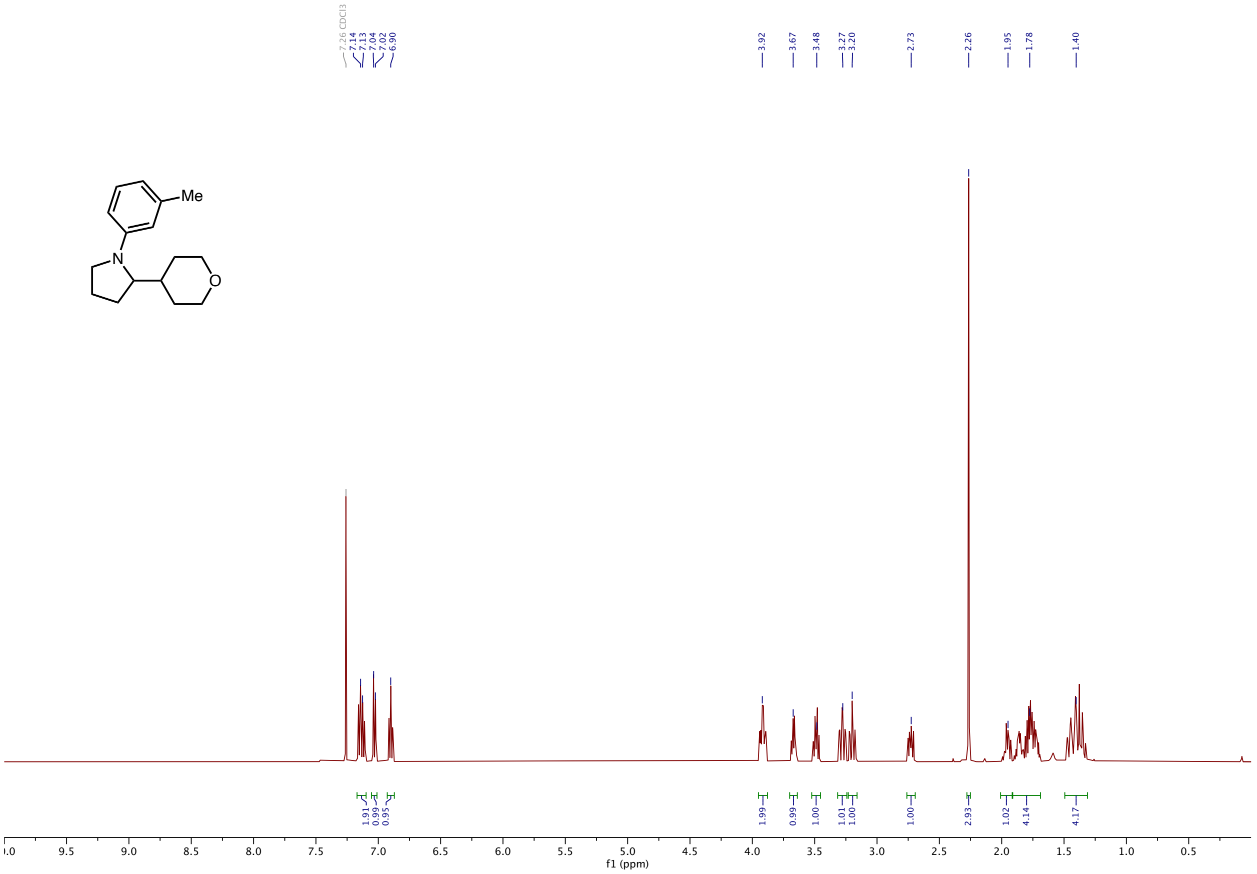
7.26 CDCl3
7.14
7.13
7.04
7.02
6.90
3.92
3.67
3.48
3.27
3.20
2.73
2.26
1.95
1.78
1.40
Me
N
O
1.91
0.99
0.95
1.99
0.99
1.00
1.01
1.00
1.00
2.93
1.02
4.14
4.17
f1 (ppm)

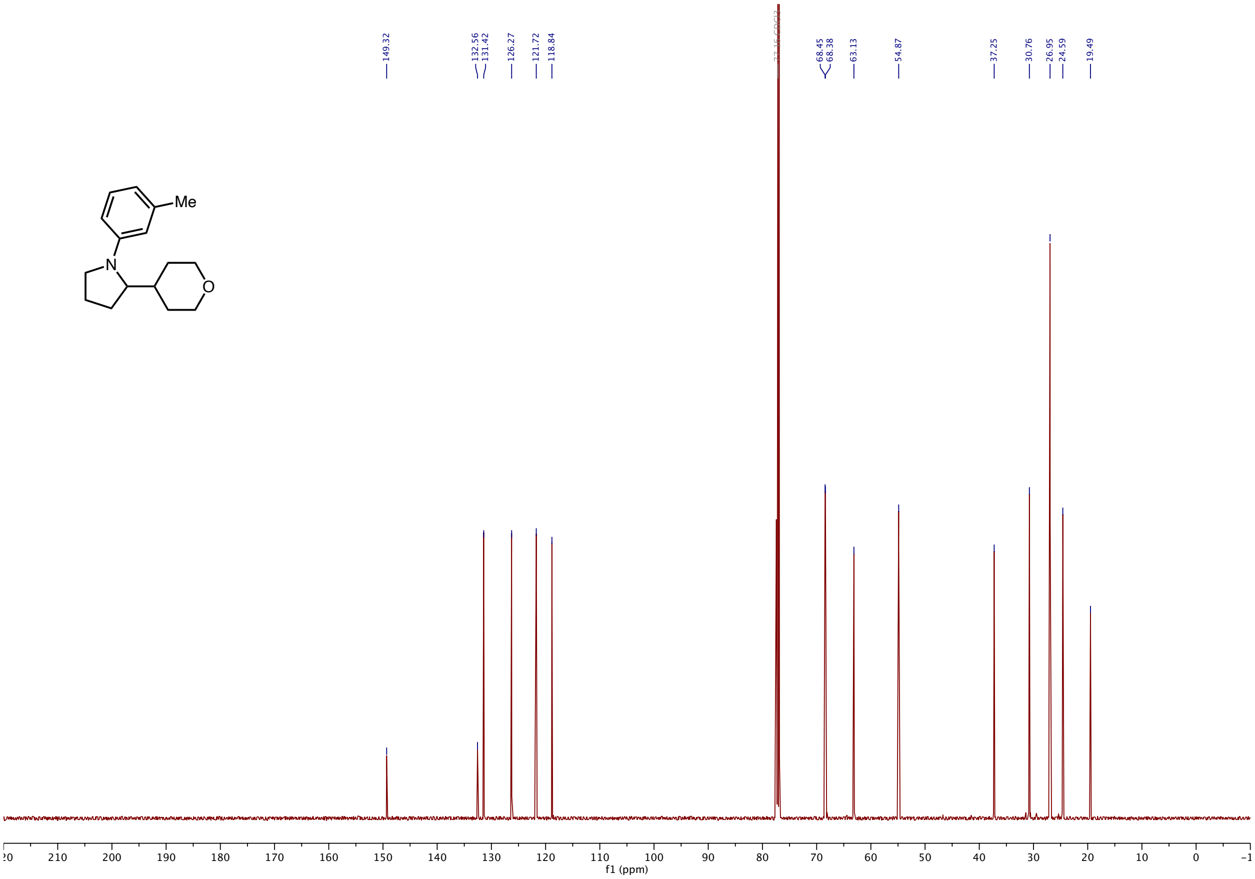
149.32
132.56
131.42
126.27
121.72
118.84
68.45
68.38
63.13
54.87
37.25
30.76
26.95
24.59
19.49
Me
N
O
f1 (ppm)

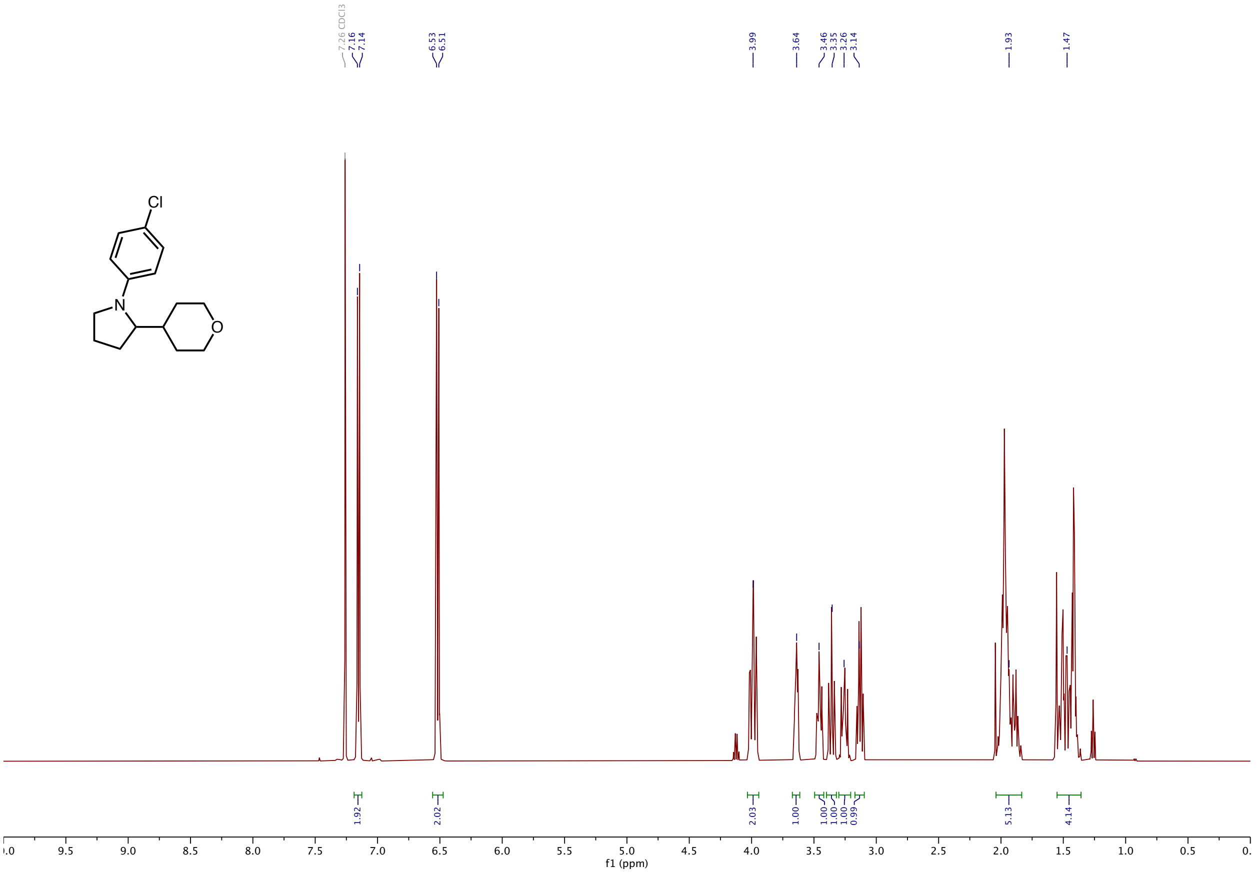
7.26 CDCl3
7.16
7.14
6.53
6.51
3.99
3.64
3.46
3.35
3.26
3.14
1.93
1.47
Cl
N
O
1.92
2.02
2.03
1.00
1.00
1.00
1.00
0.99
5.13
4.14
f1 (ppm)

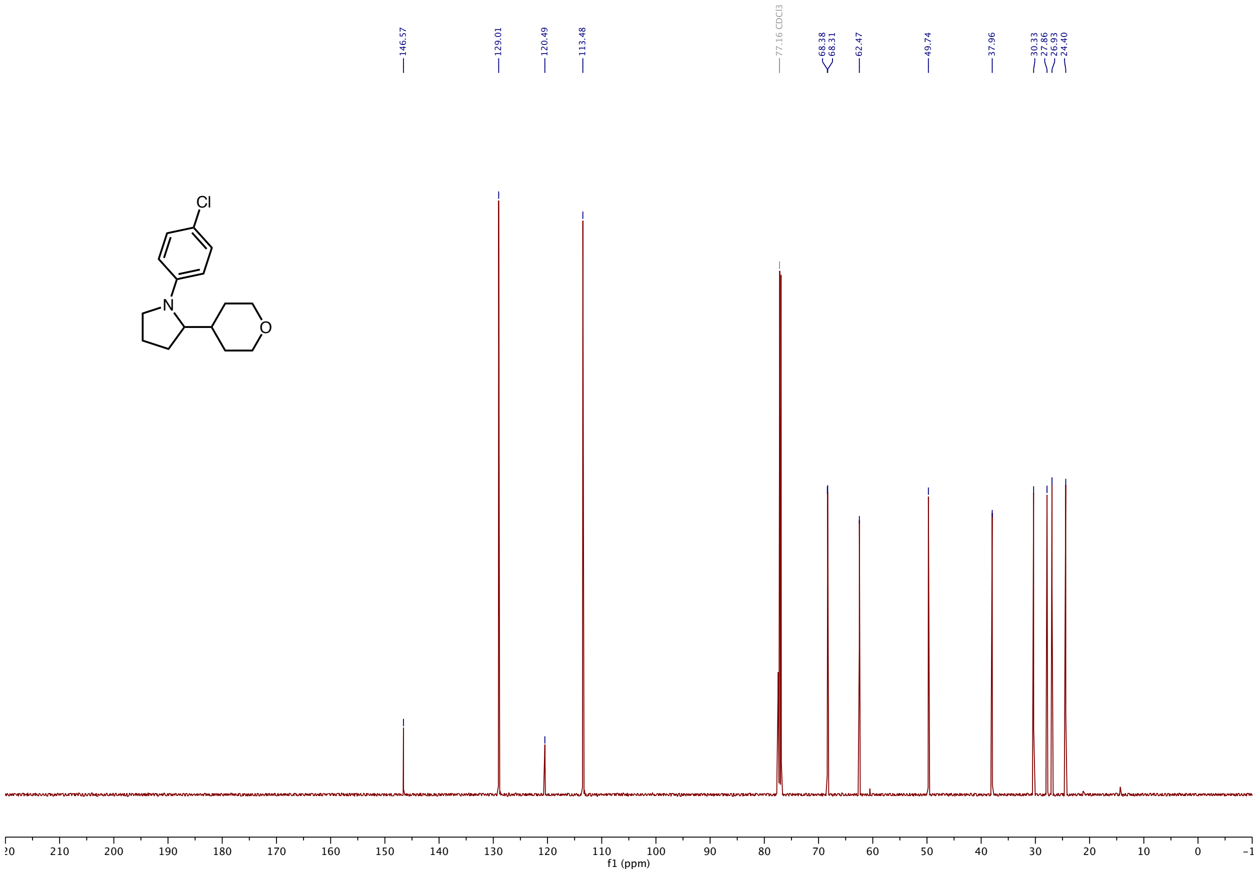
146.57
129.01
120.49
113.48
77.16 CDCl3
68.38
68.31
62.47
49.74
37.96
30.33
27.86
26.93
24.40
Cl
N
O
f1 (ppm)

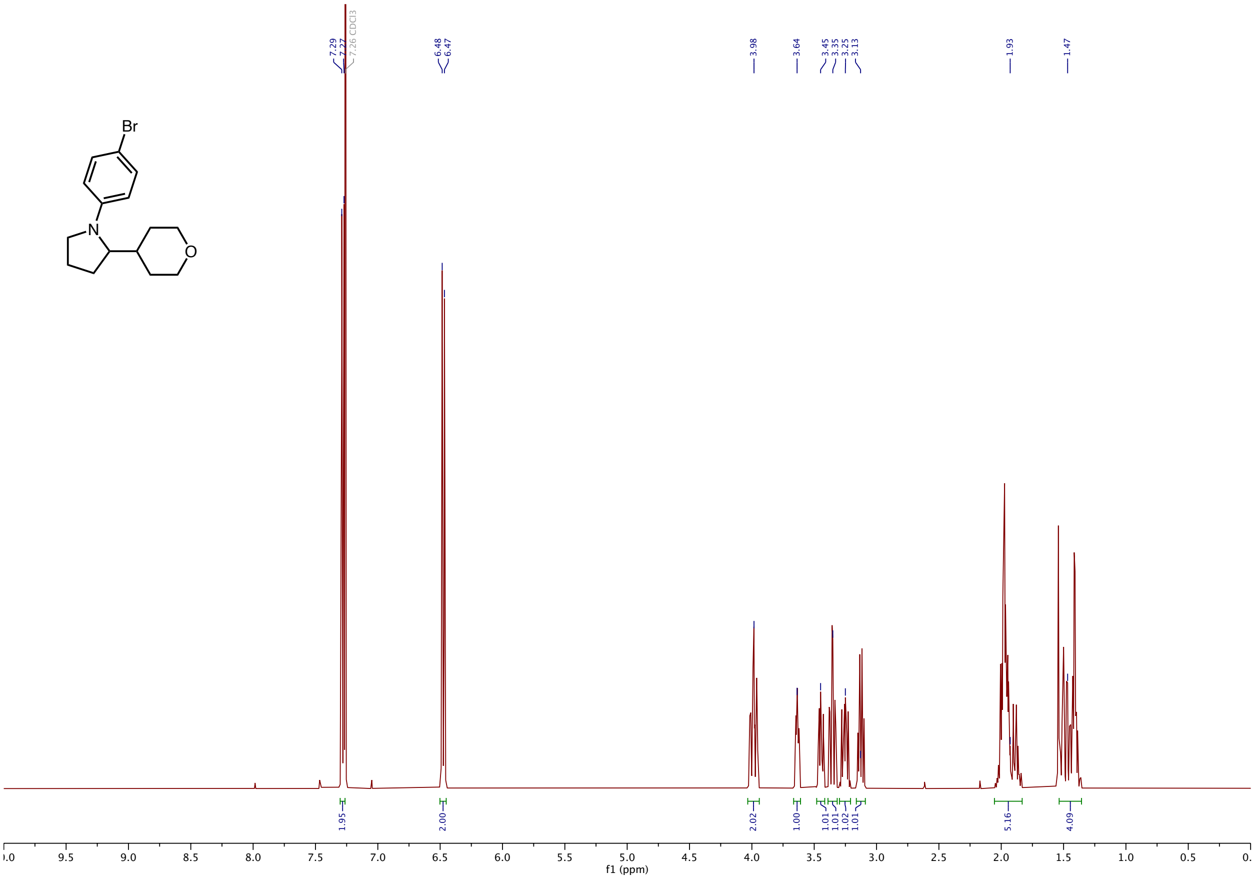

7.29
7.27
7.26 CDCl3
6.48
6.47
3.98
3.64
3.45
3.35
3.25
3.13
1.93
1.47
Br
N
O
1.95
2.00
2.02
1.00
1.01
1.01
1.02
1.01
5.16
4.09
f1 (ppm)

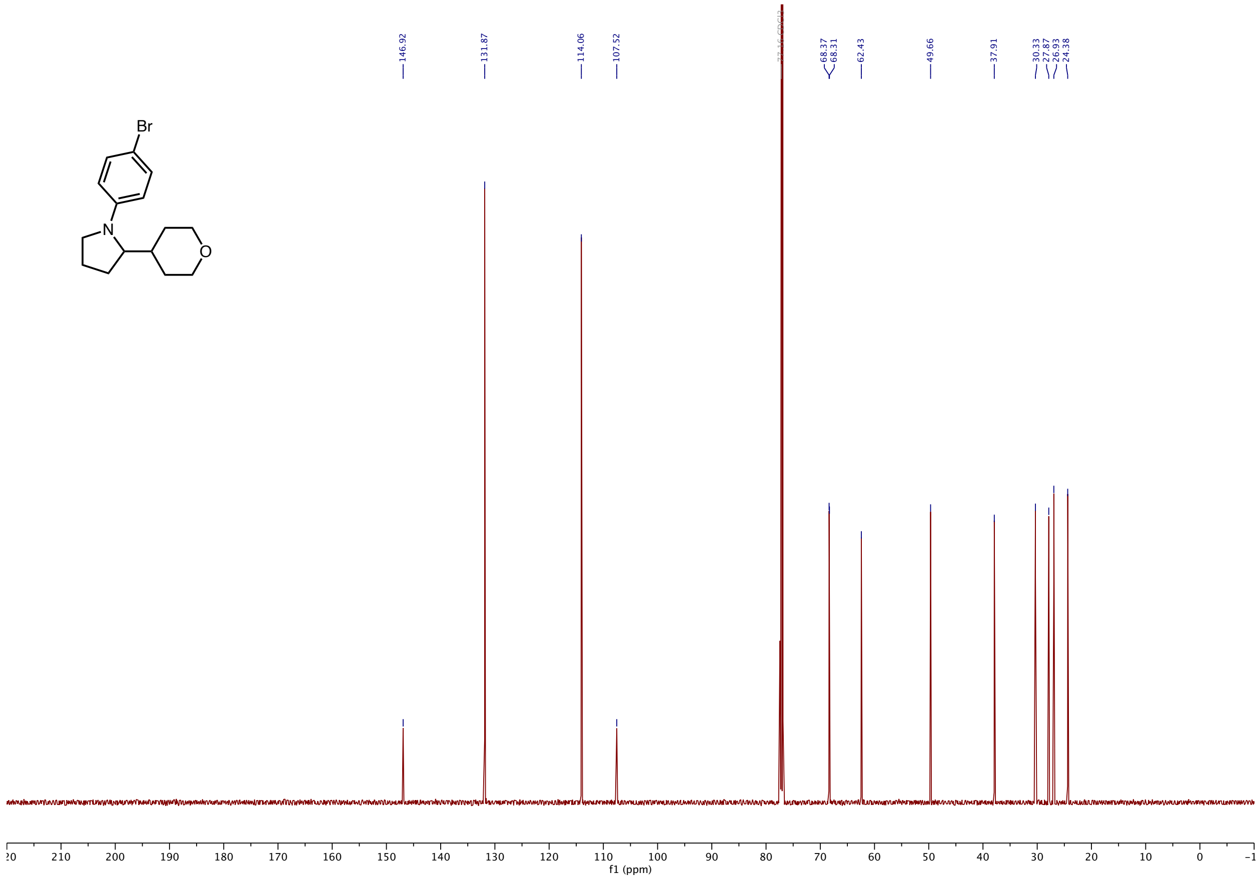

146.92
131.87
114.06
107.52
68.37
68.31
62.43
49.66
37.91
30.33
27.87
26.93
24.38
Br
N
O
f1 (ppm)

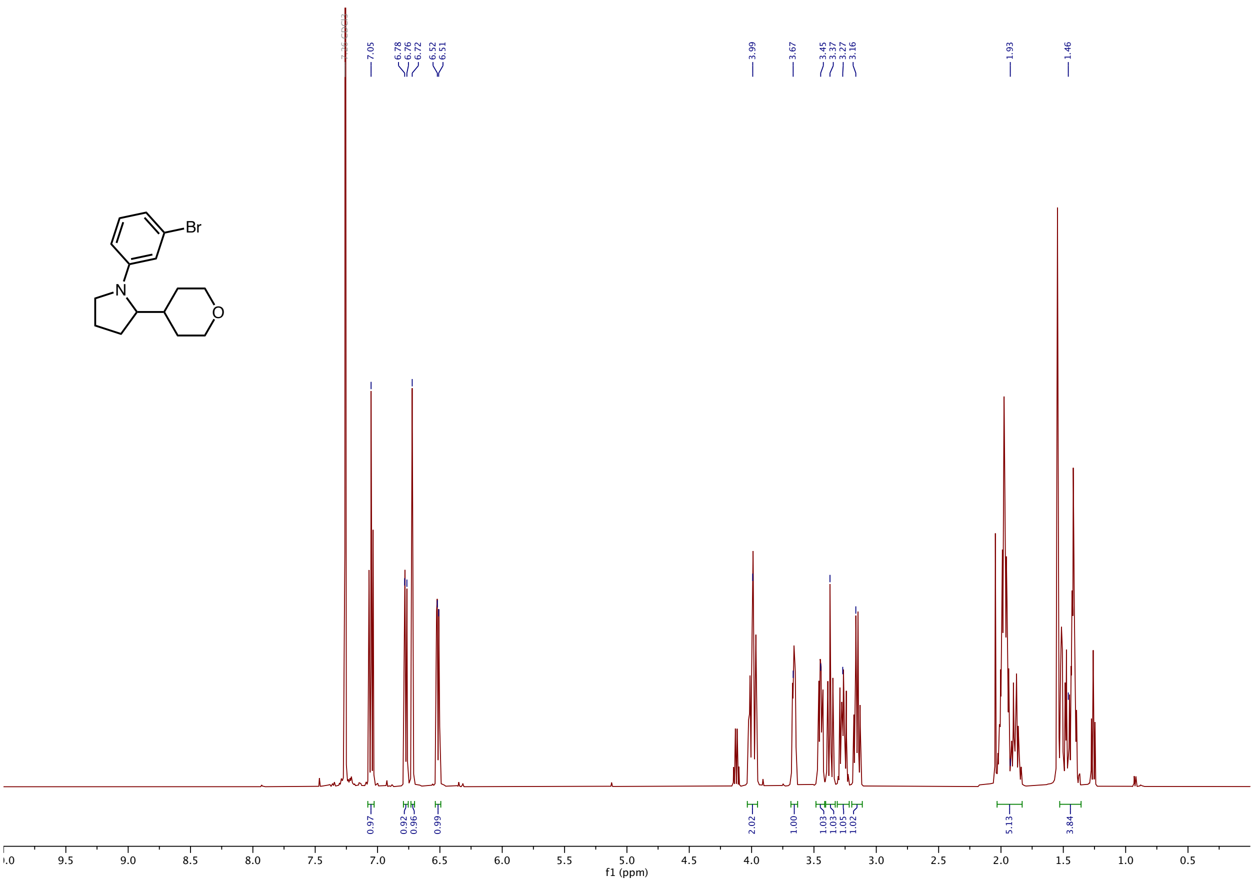
7.05
6.78
6.76
6.72
6.52
6.51
3.99
3.67
3.45
3.37
3.27
3.16
1.93
1.46
Br
N
O
0.97
0.92
0.96
0.99
2.02
1.00
1.03
1.03
1.05
1.02
5.13
3.84
f1 (ppm)

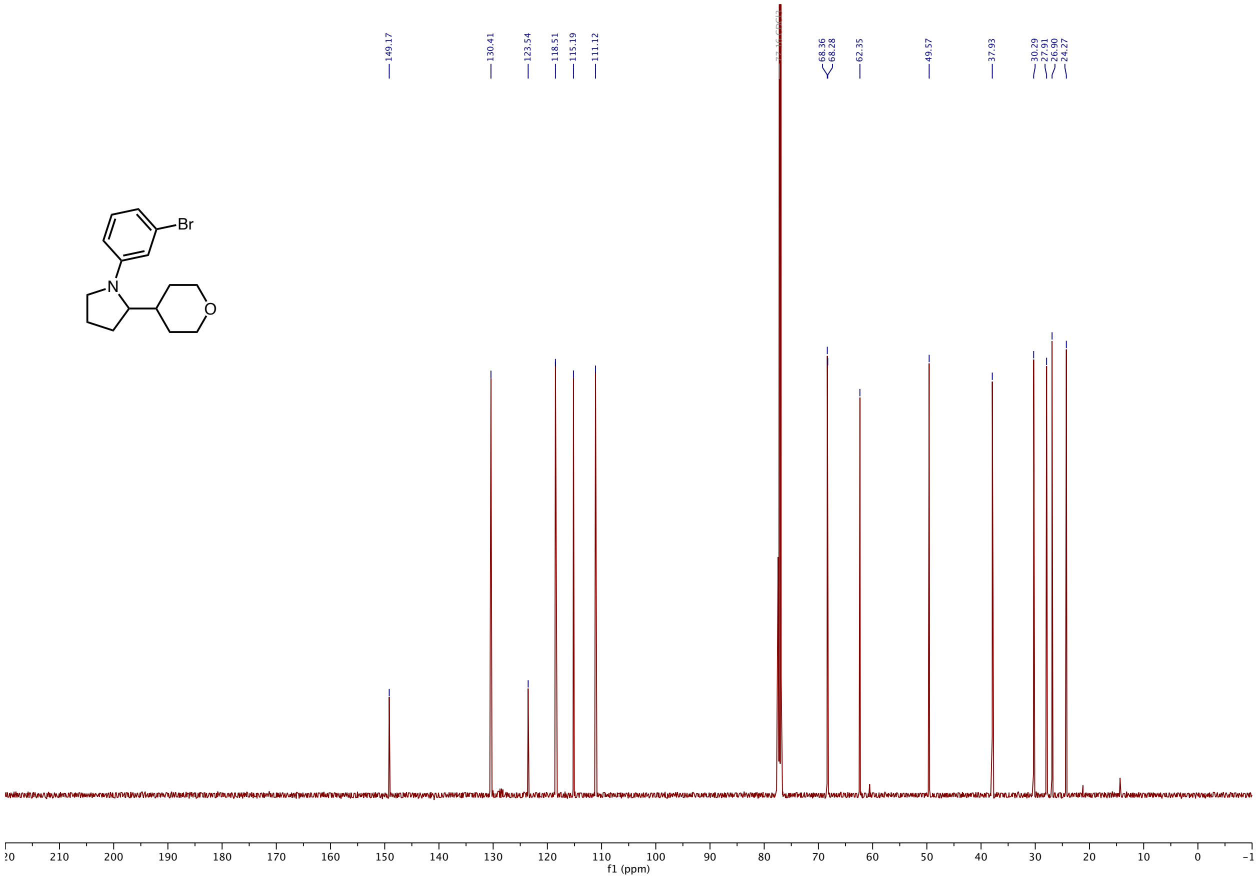
149.17
130.41
123.54
118.51
115.19
111.12
68.36
68.28
62.35
49.57
37.93
30.29
27.91
26.90
24.27
Br
N
O
f1 (ppm)

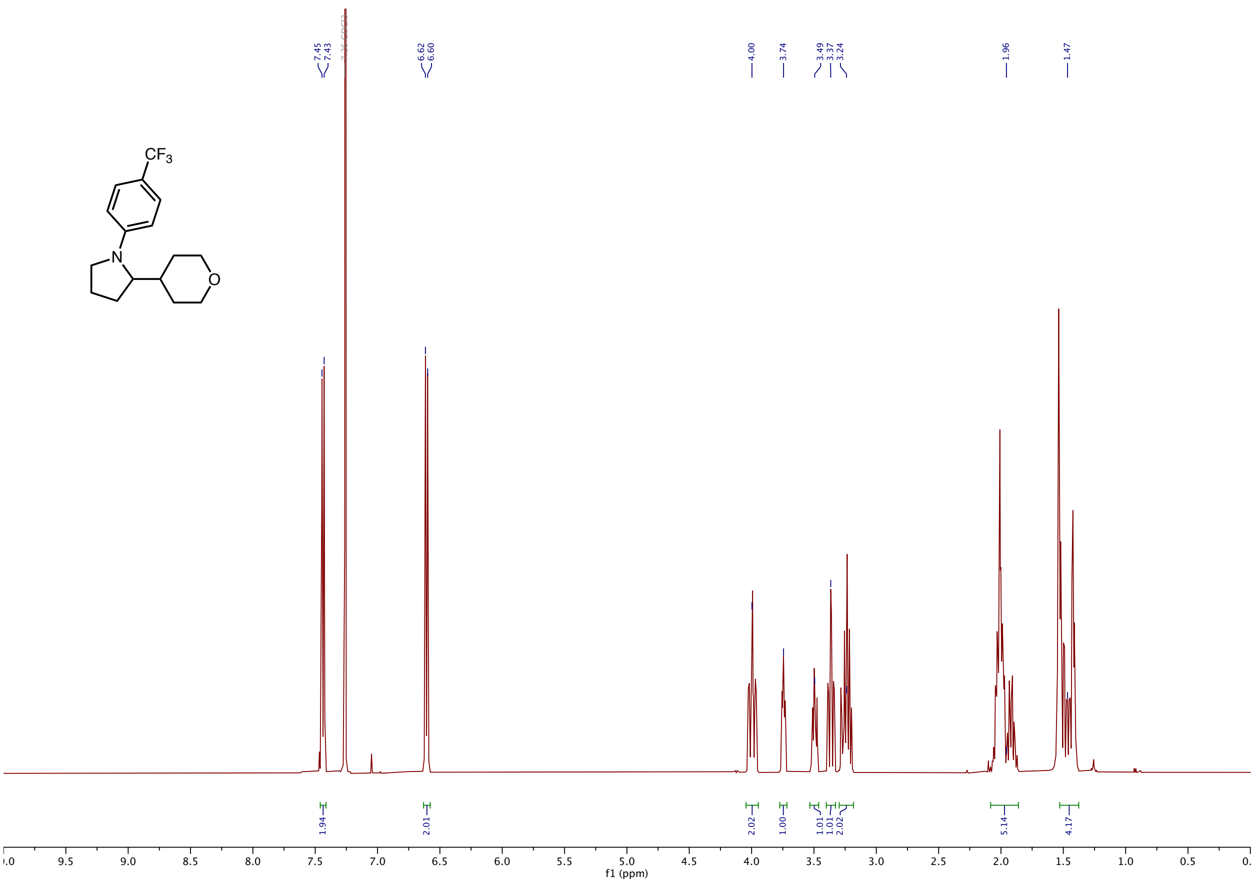

7.45
7.43
6.62
6.60
4.00
3.74
3.49
3.37
3.24
1.96
1.47
CF3
N
O
1.94
2.01
2.02
1.00
1.01
1.01
2.02
5.14
4.17
f1 (ppm)

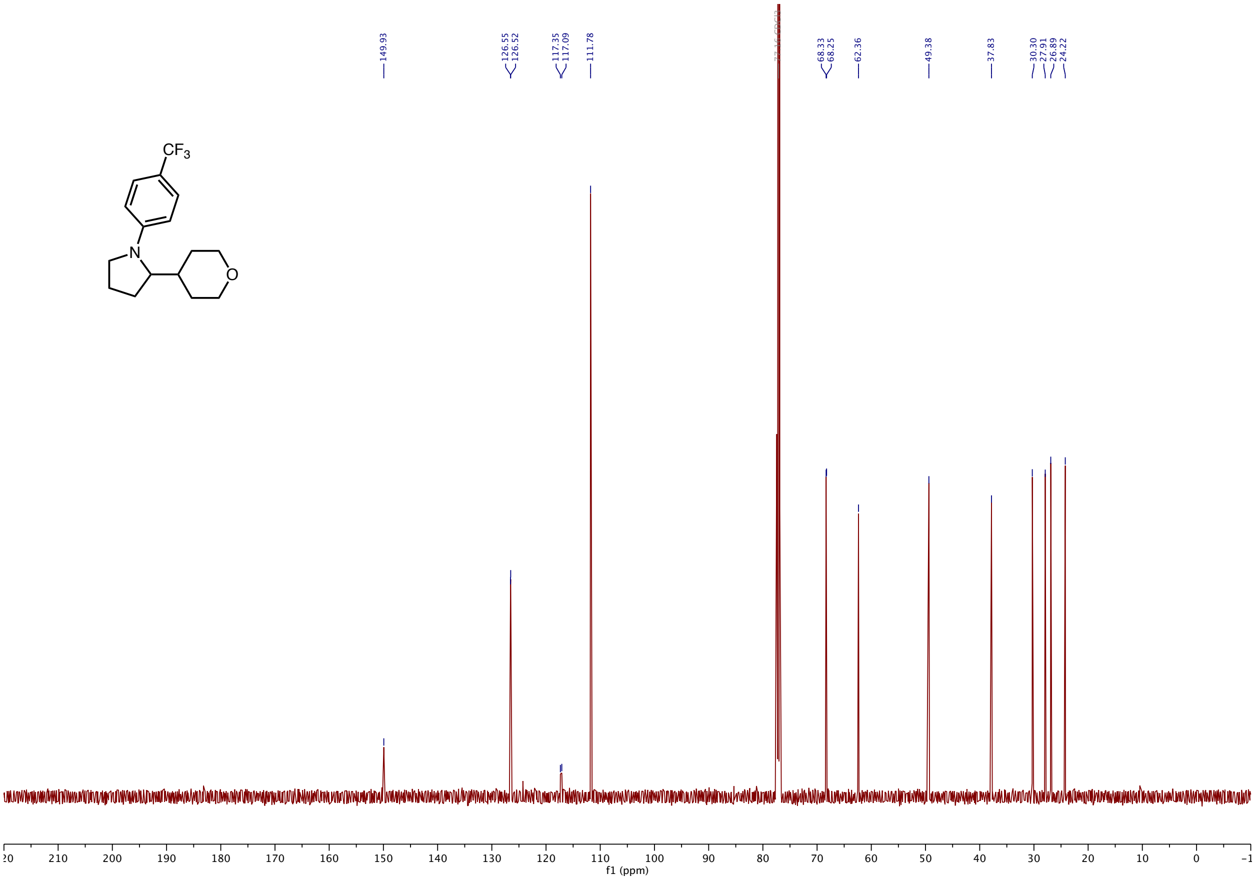

149.93
126.55
126.52
117.35
117.09
111.78
68.33
68.25
62.36
49.38
37.83
30.30
27.91
26.89
24.22
CF3
N
O
f1 (ppm)

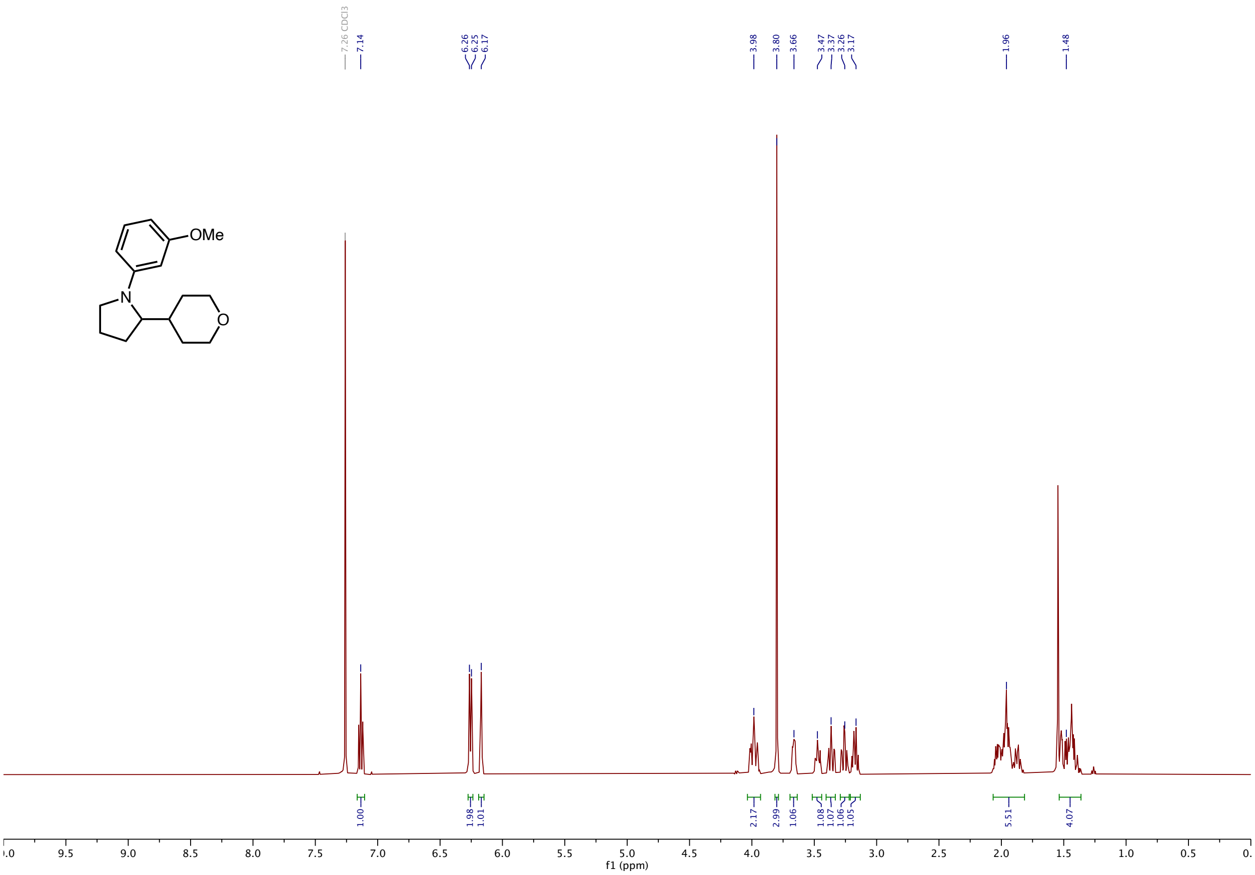
7.26 CDCl3
7.14
6.26
6.25
6.17
3.98
3.80
3.66
3.47
3.37
3.26
3.17
1.96
1.48
OMe
N
O
1.00
1.98
1.01
2.17
2.99
1.06
1.08
1.07
1.06
1.05
5.51
4.07
f1 (ppm)

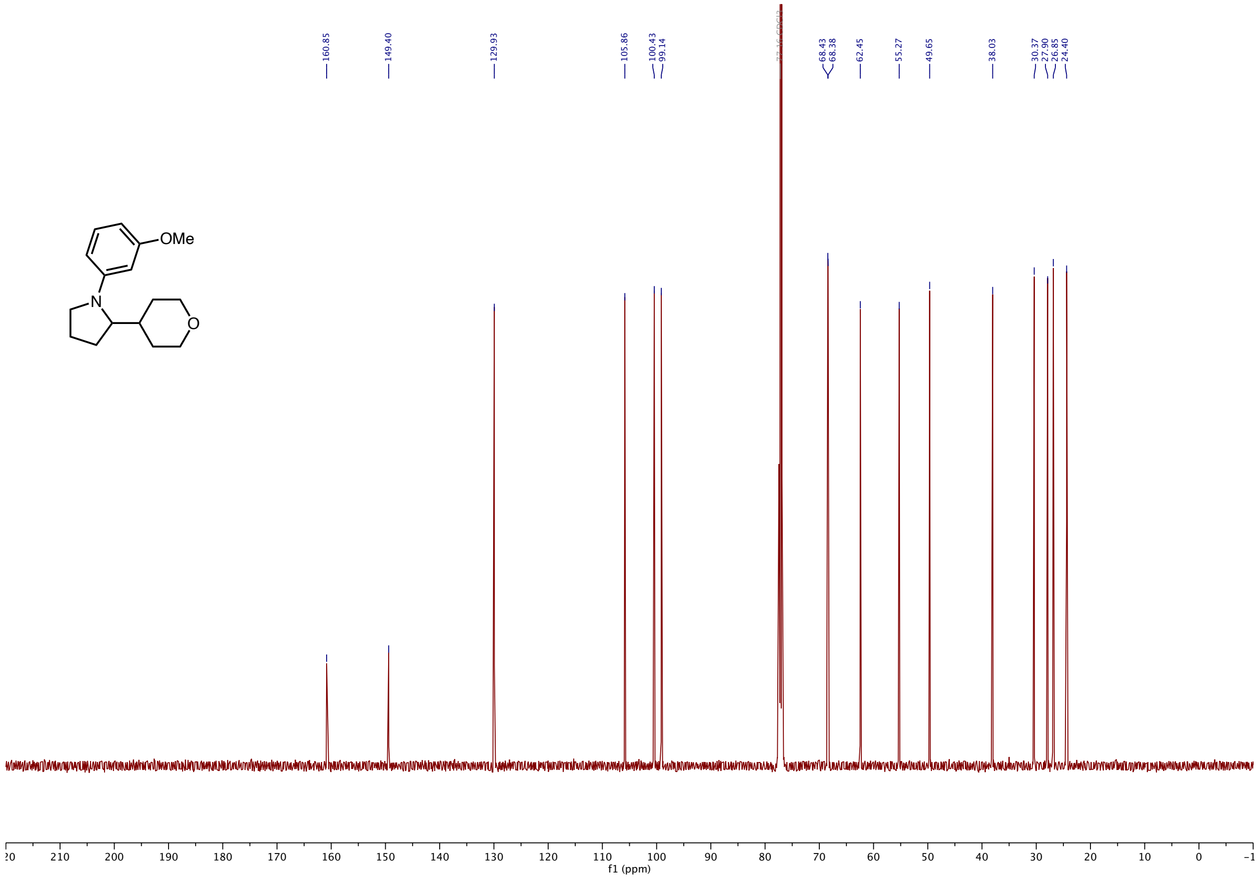
160.85
149.40
129.93
105.86
100.43
99.14
68.43
68.38
62.45
55.27
49.65
38.03
30.37
27.90
26.85
24.40
OMe
N
O
f1 (ppm)

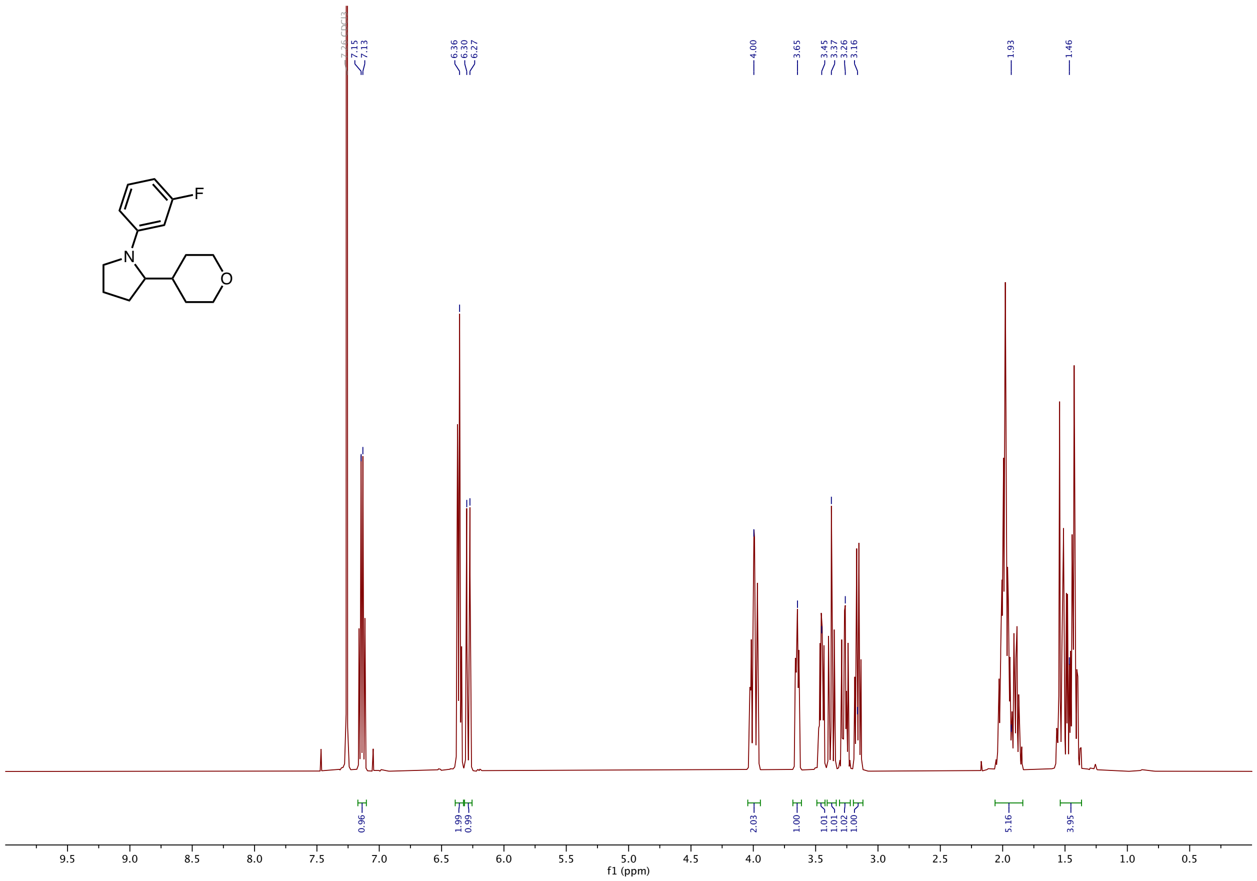

7.15
7.13
6.36
6.30
6.27
4.00
3.65
3.45
3.37
3.26
3.16
1.93
1.46
0.96
1.99
0.99
2.03
1.00
1.01
1.01
1.02
1.00
5.16
3.95
f1 (ppm)
F
N
O

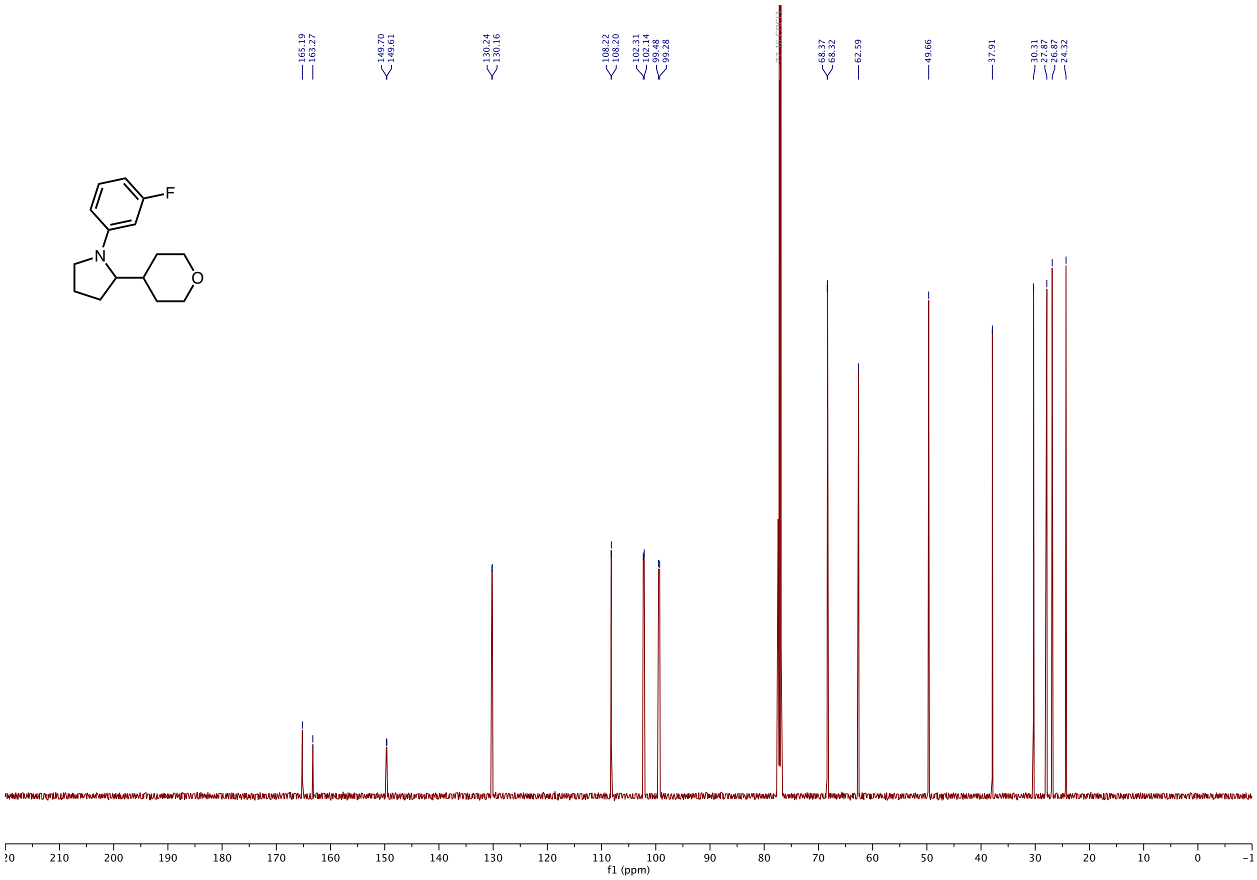

165.19
163.27
149.70
149.61
130.24
130.16
108.22
108.20
102.31
102.14
99.48
99.28
68.37
68.32
62.59
49.66
37.91
30.31
27.87
26.87
24.32
f1 (ppm)
F
N
O

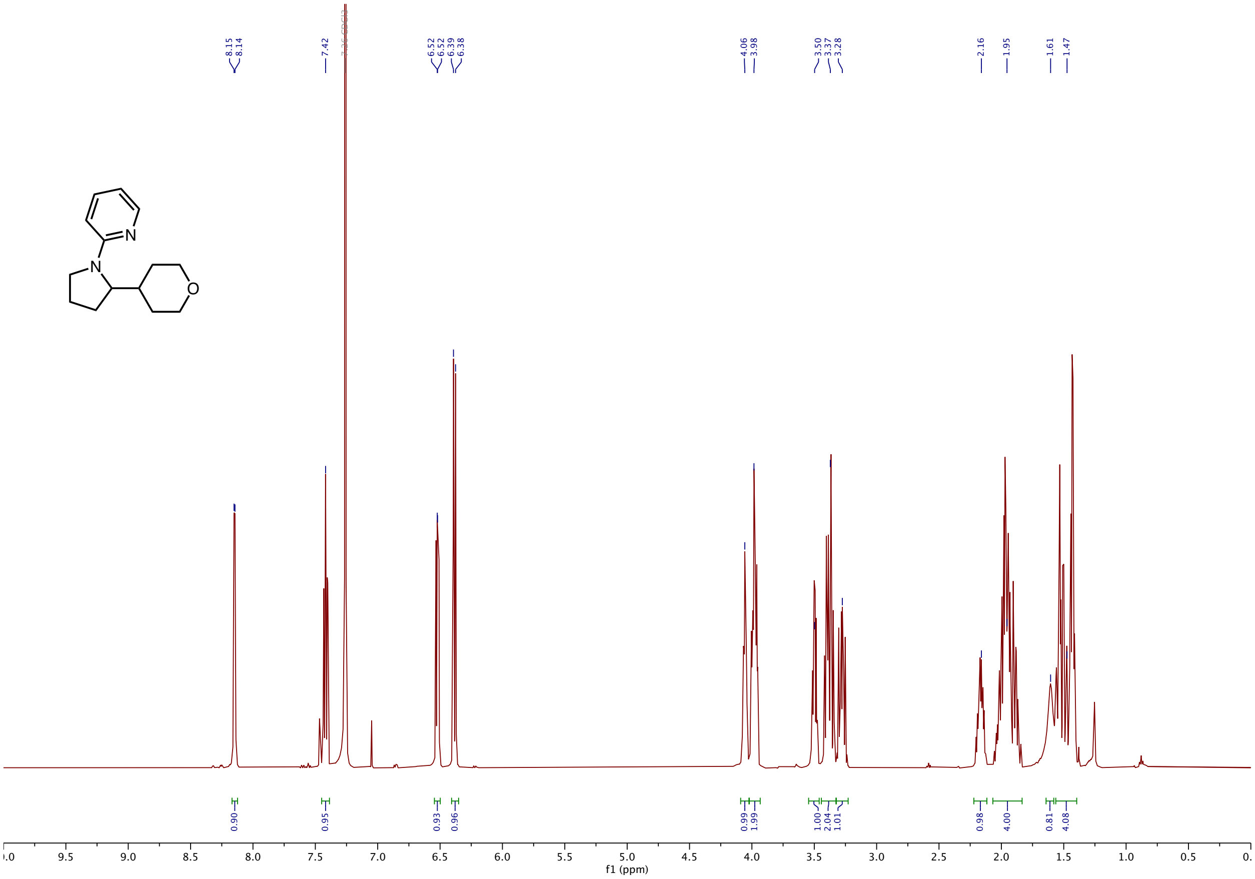

8.15
8.14
7.42
6.52
6.52
6.39
6.38
4.06
3.98
3.50
3.37
3.28
2.16
1.95
1.61
1.47
0.90
0.95
0.93
0.96
0.99
1.99
1.00
2.04
1.01
0.98
4.00
0.81
4.08
N
N
O
f1 (ppm)

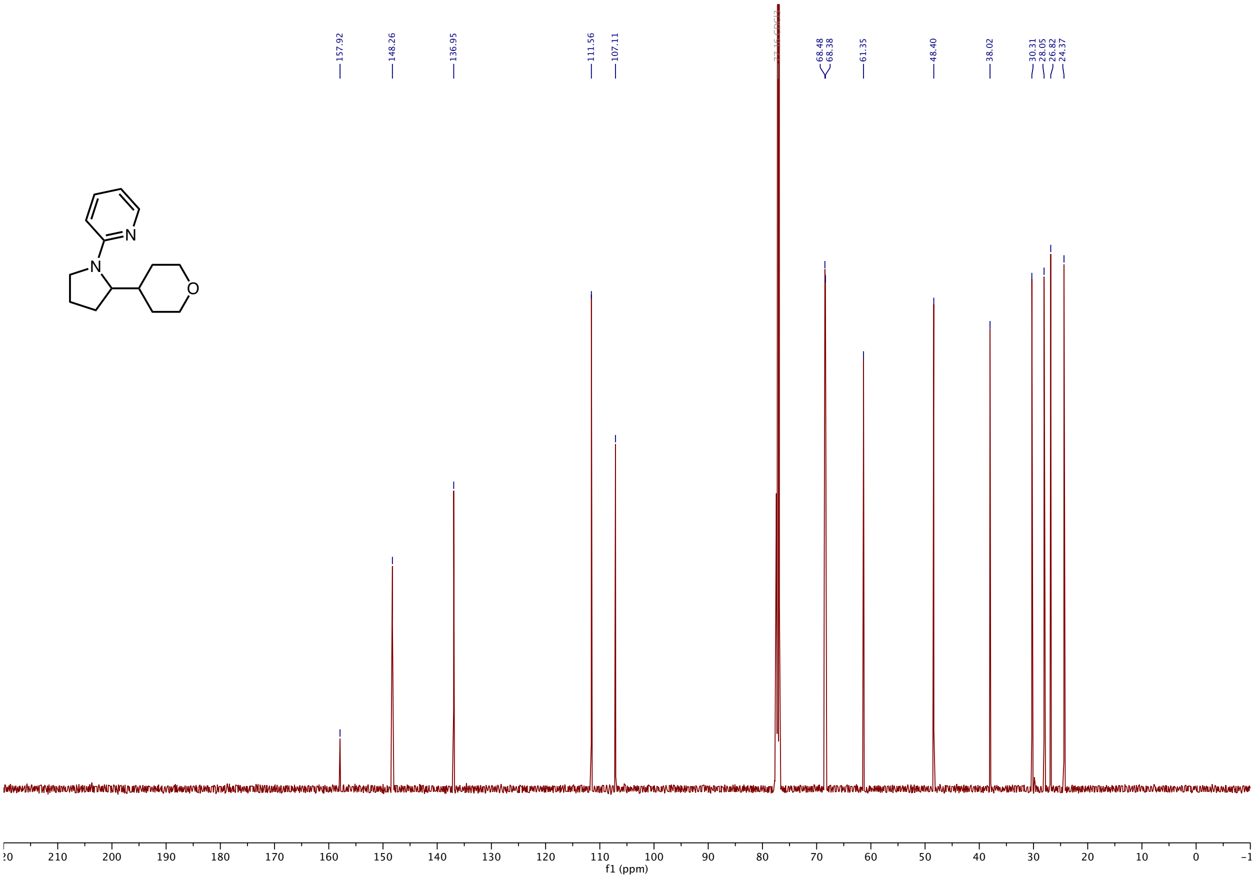

157.92
148.26
136.95
111.56
107.11
68.48
68.38
61.35
48.40
38.02
30.31
28.05
26.82
24.37
N
N
O
f1 (ppm)

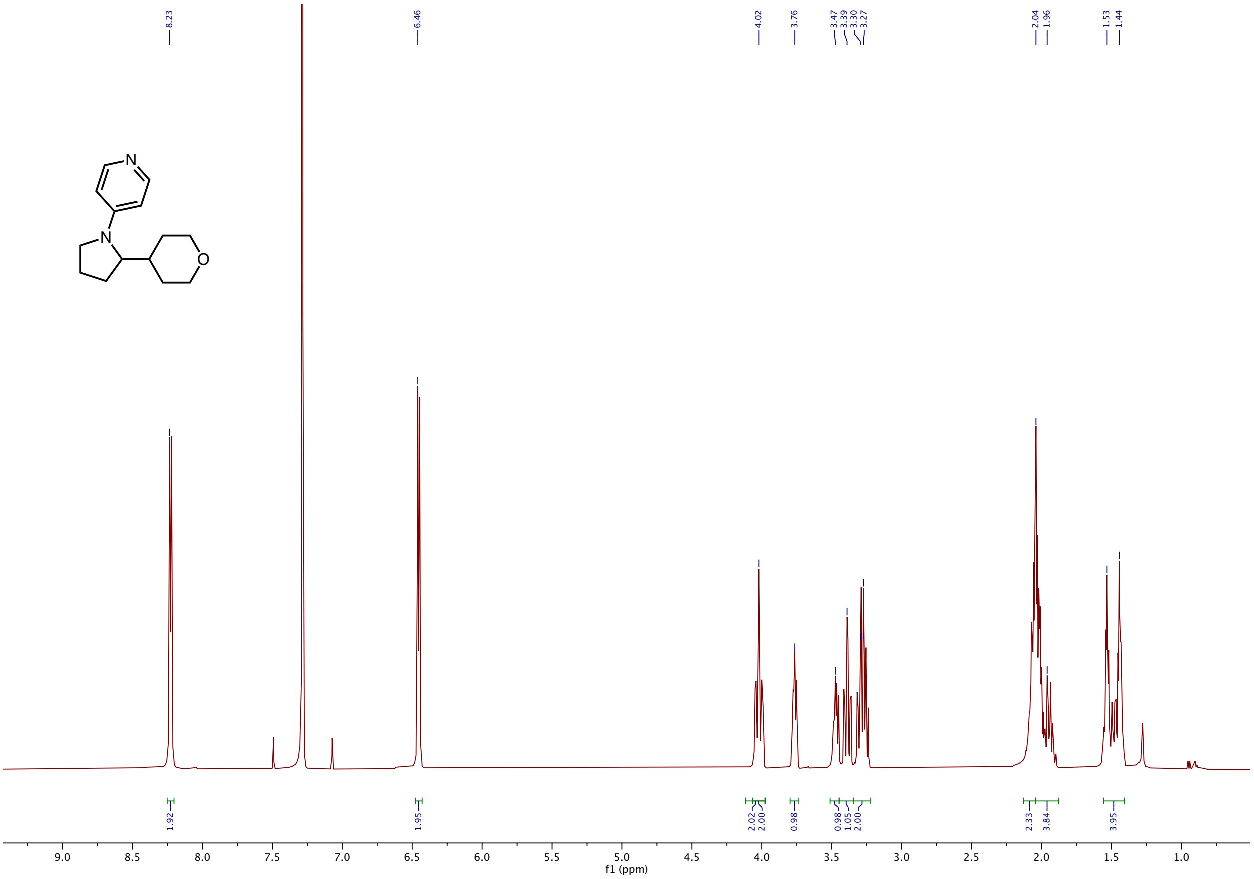
8.23
6.46
4.02
3.76
3.47
3.39
3.30
3.27
2.04
1.96
1.53
1.44
N
O
1.92
1.95
2.02
2.00
0.98
0.98
1.05
2.00
2.33
3.84
3.95
9.0 8.5 8.0 7.5 7.0 6.5 6.0 5.5 5.0 4.5 4.0 3.5 3.0 2.5 2.0 1.5 1.0
f1 (ppm)

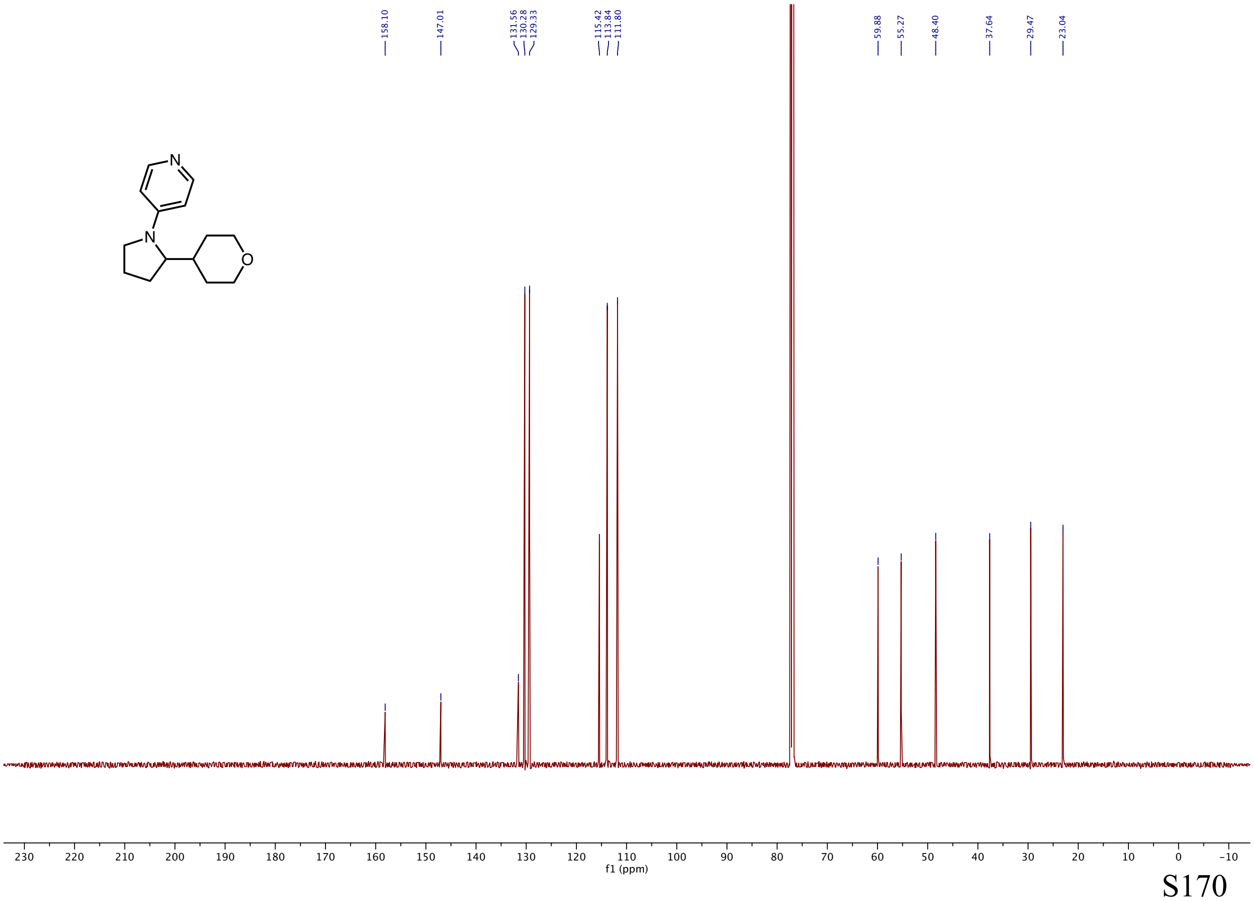
158.10
147.01
131.56
130.28
129.33
115.42
113.84
111.80
59.88
55.27
48.40
37.64
29.47
23.04
N
O
230 220 210 200 190 180 170 160 150 140 130 120 110 100 90 80 70 60 50 40 30 20 10 0 -10
f1 (ppm)

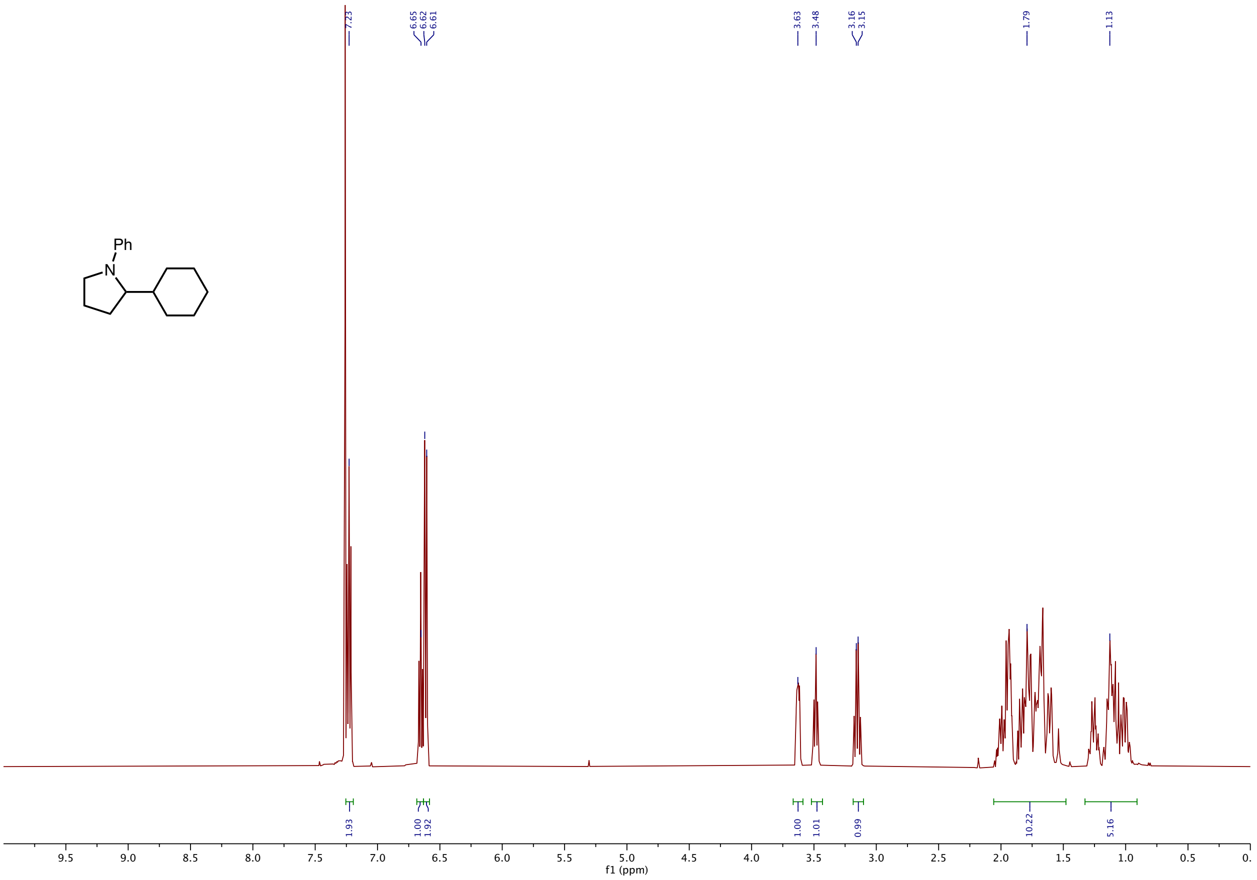

Ph
N
7.23
6.65
6.62
6.61
3.63
3.48
3.16
3.15
1.79
1.13
1.93
1.00
1.92
1.00
1.01
0.99
10.22
5.16
f1 (ppm)

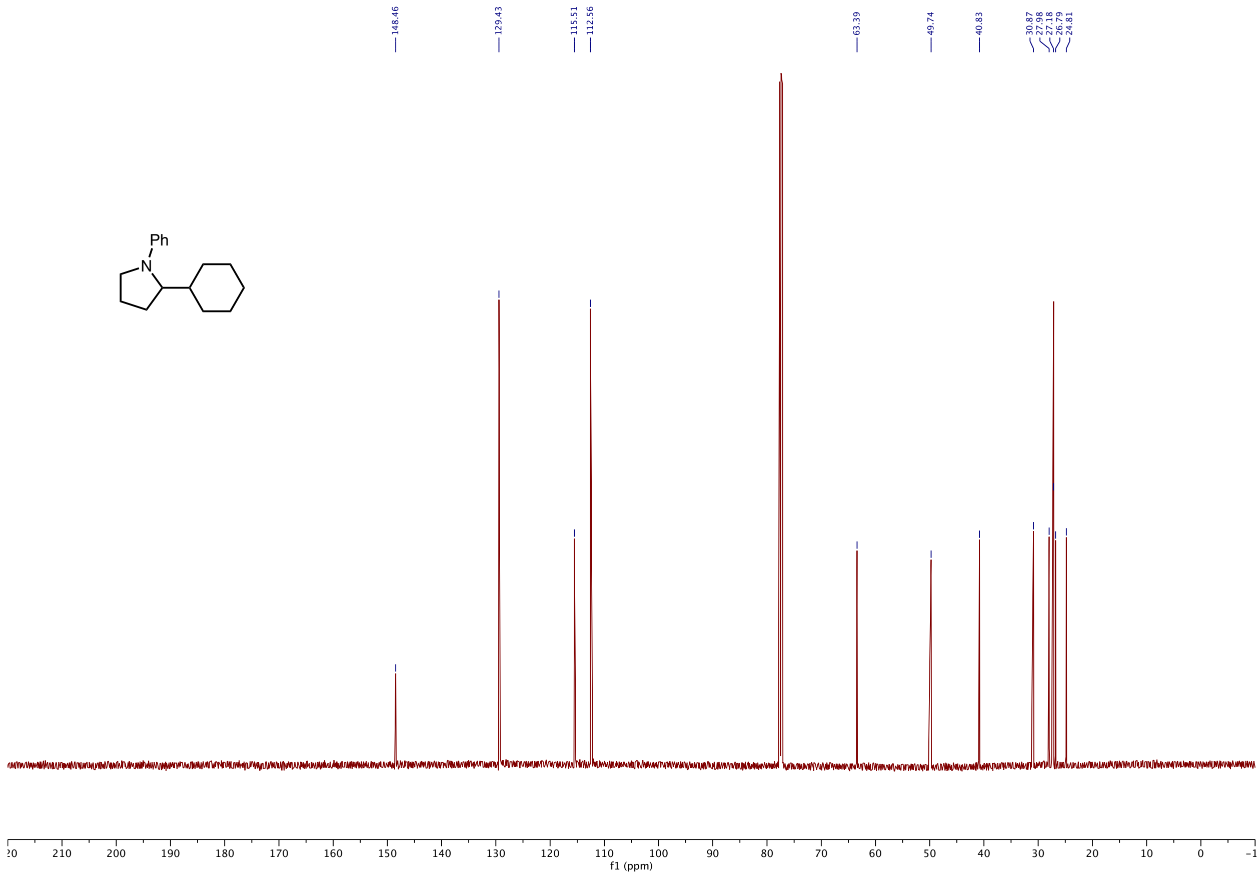

Ph
N
148.46
129.43
115.51
112.56
63.39
49.74
40.83
30.87
27.98
27.18
26.79
24.81
f1 (ppm)

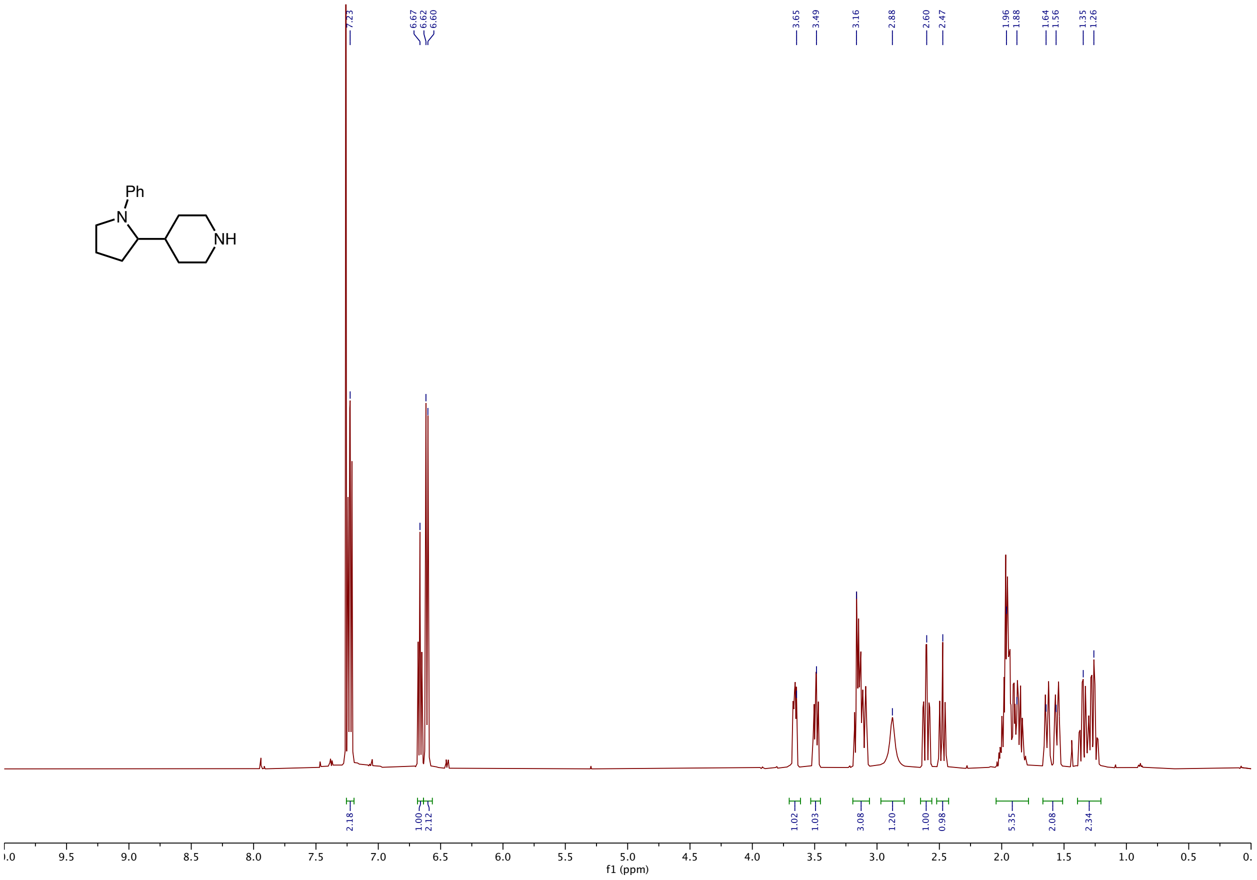

7.23
6.67
6.62
6.60
3.65
3.49
3.16
2.88
2.60
2.47
1.96
1.88
1.64
1.56
1.35
1.26
Ph
N
NH
2.18
1.00
2.12
1.02
1.03
3.08
1.20
1.00
0.98
5.35
2.08
2.34
f1 (ppm)

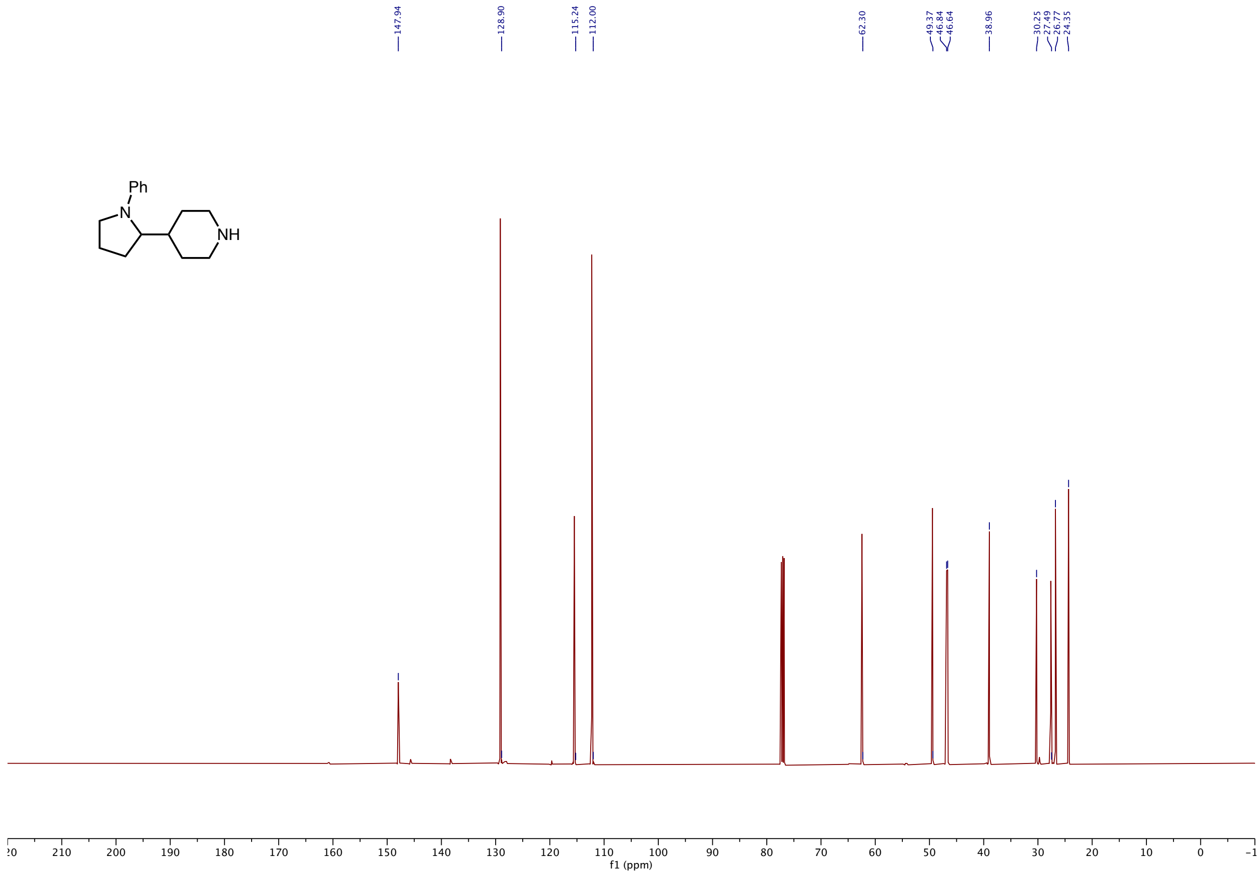

147.94
128.90
115.24
112.00
62.30
49.37
46.84
46.64
38.96
30.25
27.49
26.77
24.35
Ph
N
NH
f1 (ppm)

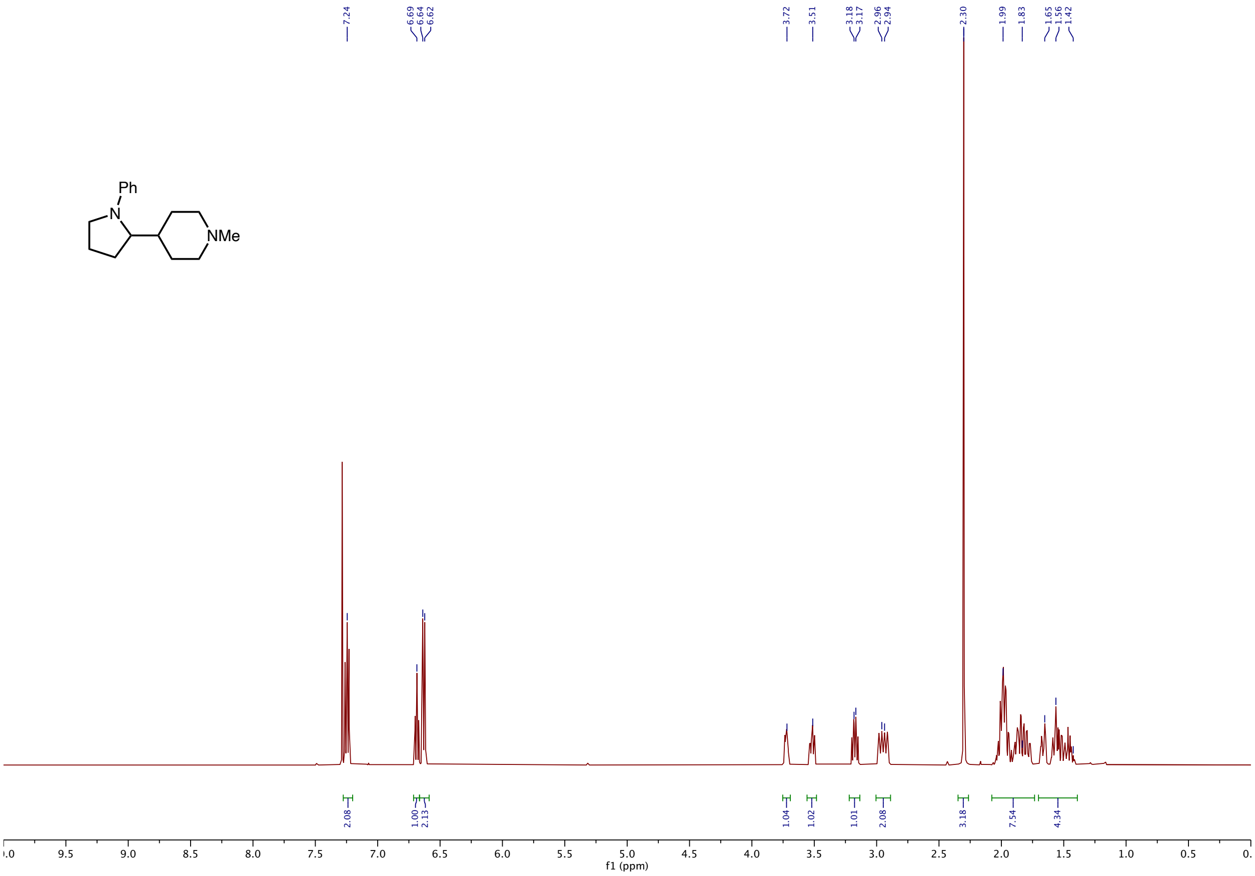
7.24
6.69
6.64
6.62
3.72
3.51
3.18
3.17
2.96
2.94
2.30
1.99
1.83
1.65
1.56
1.42
Ph
N
NMe
2.08
1.00
2.13
1.04
1.02
1.01
2.08
3.18
7.54
4.34
f1 (ppm)

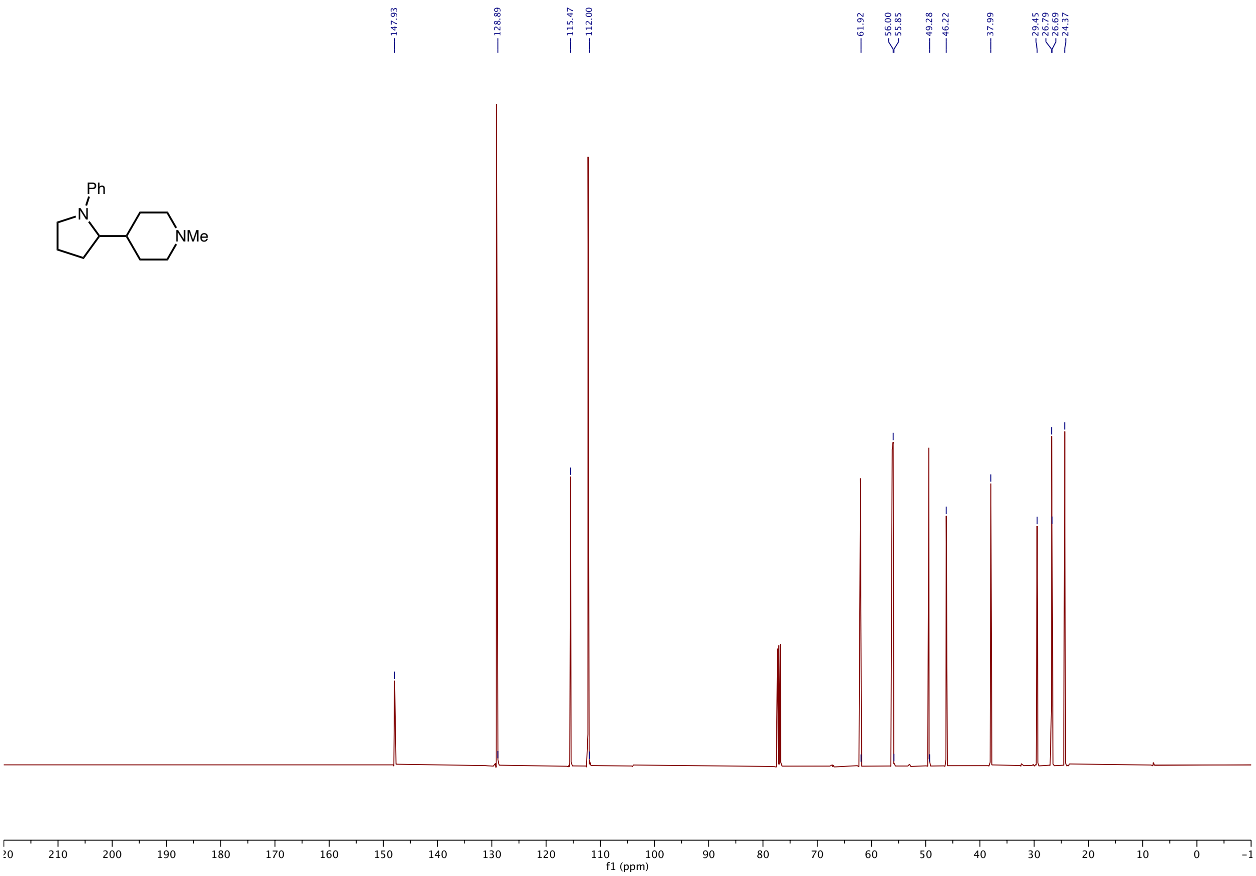
147.93
128.89
115.47
112.00
61.92
56.00
55.85
49.28
46.22
37.99
29.45
26.79
26.69
24.37
Ph
N
NMe
f1 (ppm)

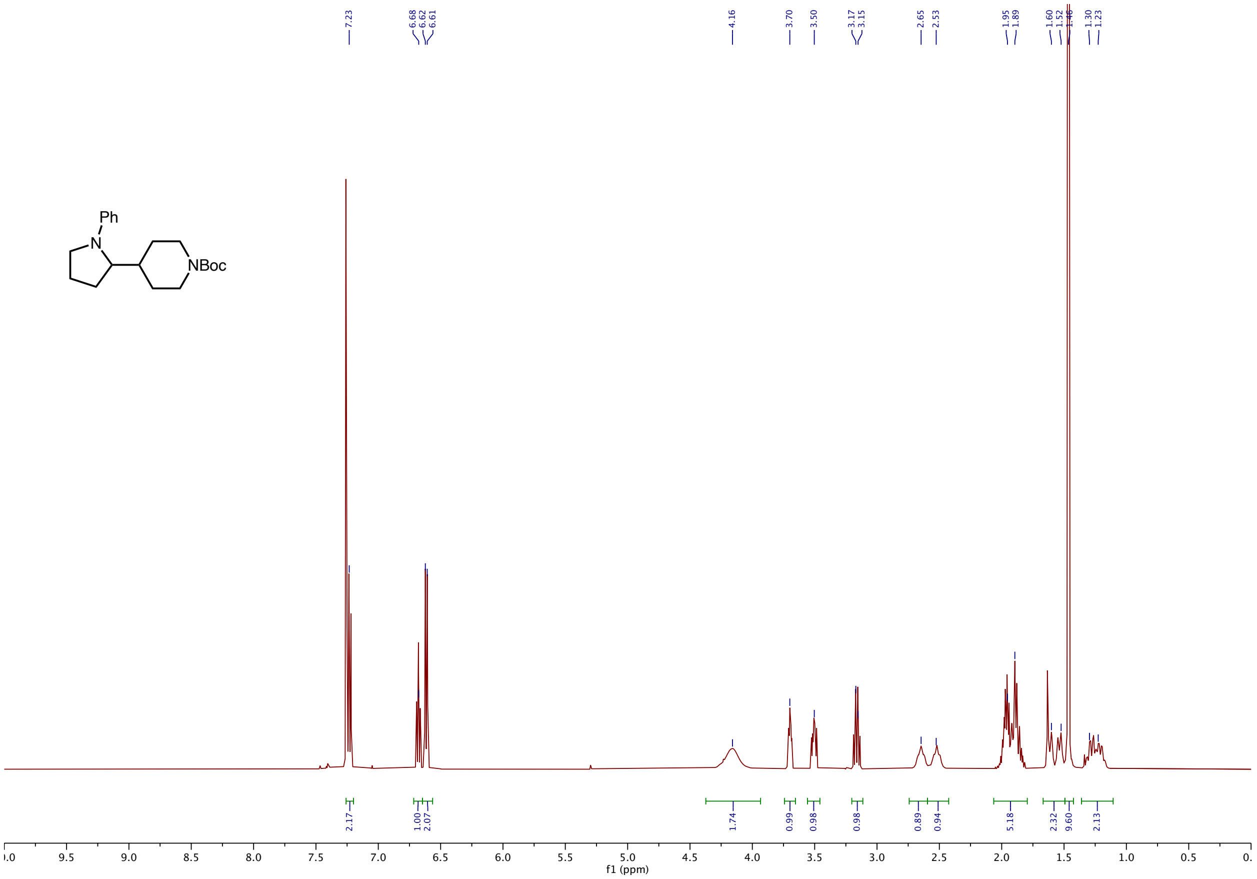
Ph
N
NBoc
f1 (ppm)

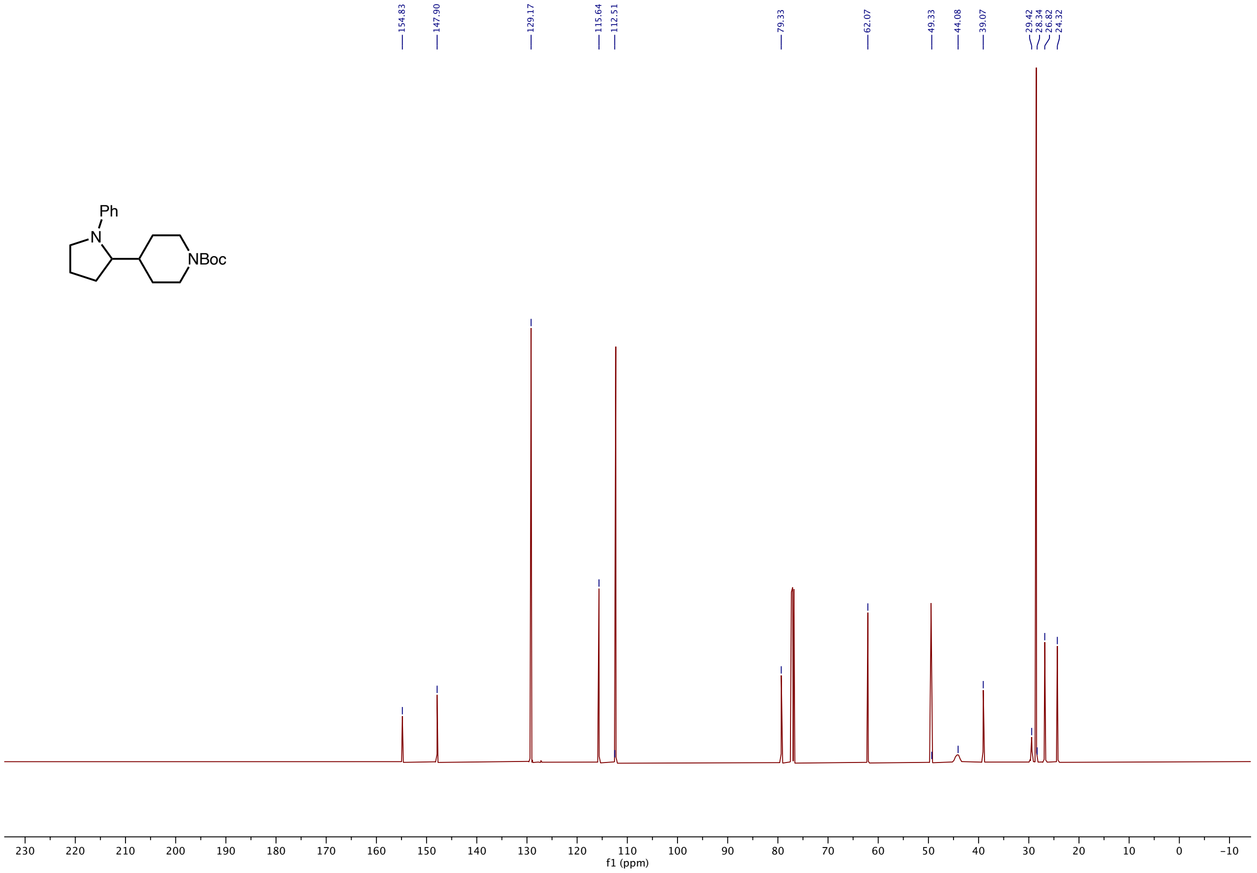
Ph
N
NBoc
f1 (ppm)

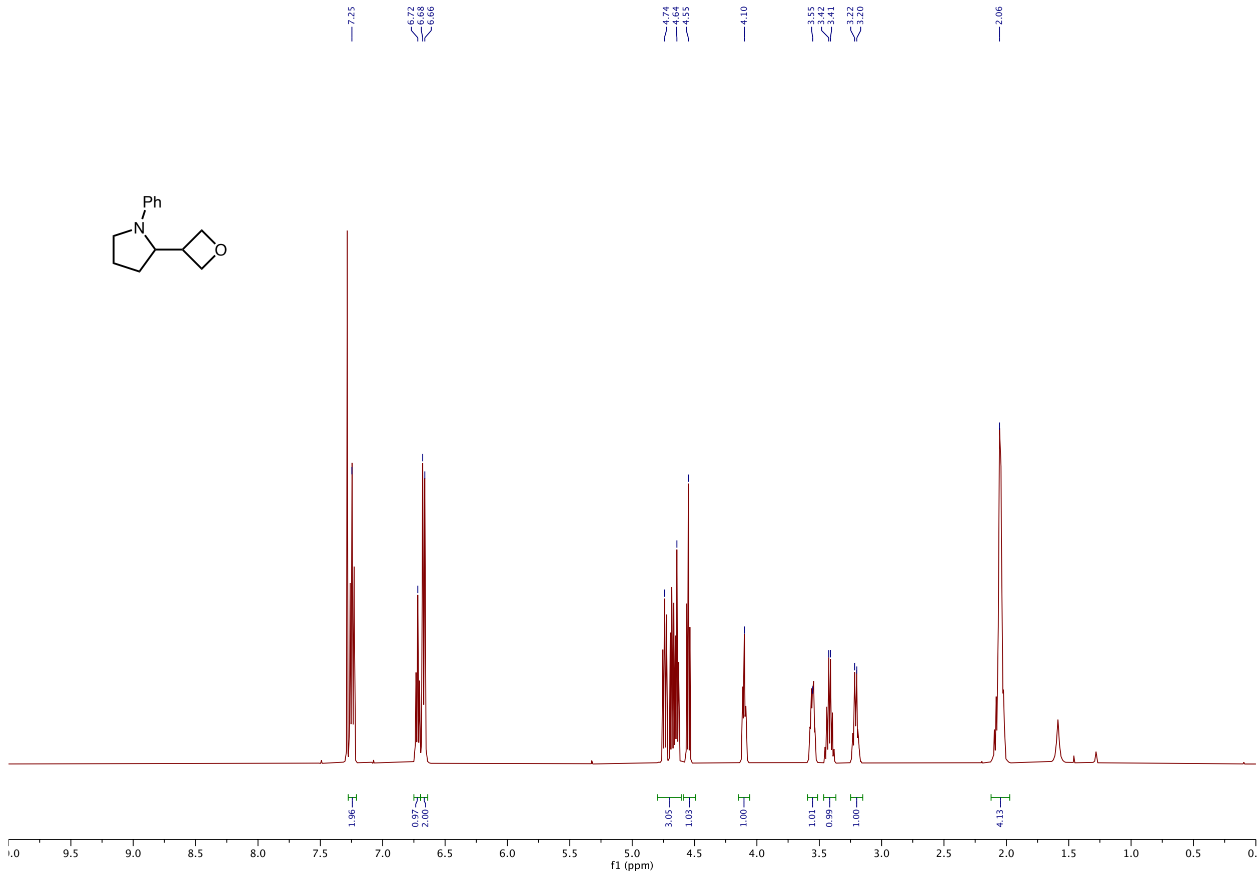

7.25
6.72
6.68
6.66
4.74
4.64
4.55
4.10
3.55
3.42
3.41
3.22
3.20
2.06
Ph
N
O
1.96
0.97
2.00
3.05
1.03
1.00
1.01
0.99
1.00
4.13
f1 (ppm)

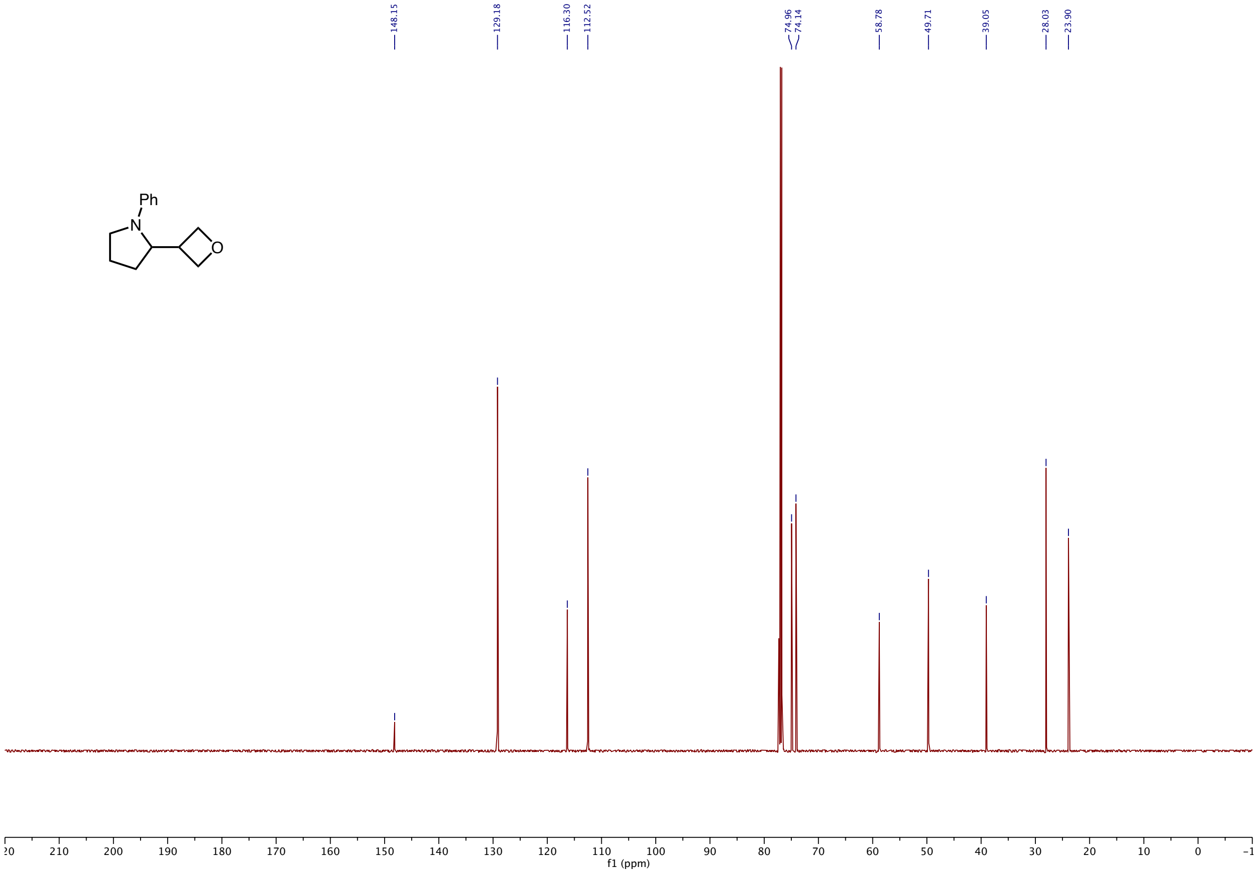

148.15
129.18
116.30
112.52
74.96
74.14
58.78
49.71
39.05
28.03
23.90
Ph
N
O
f1 (ppm)

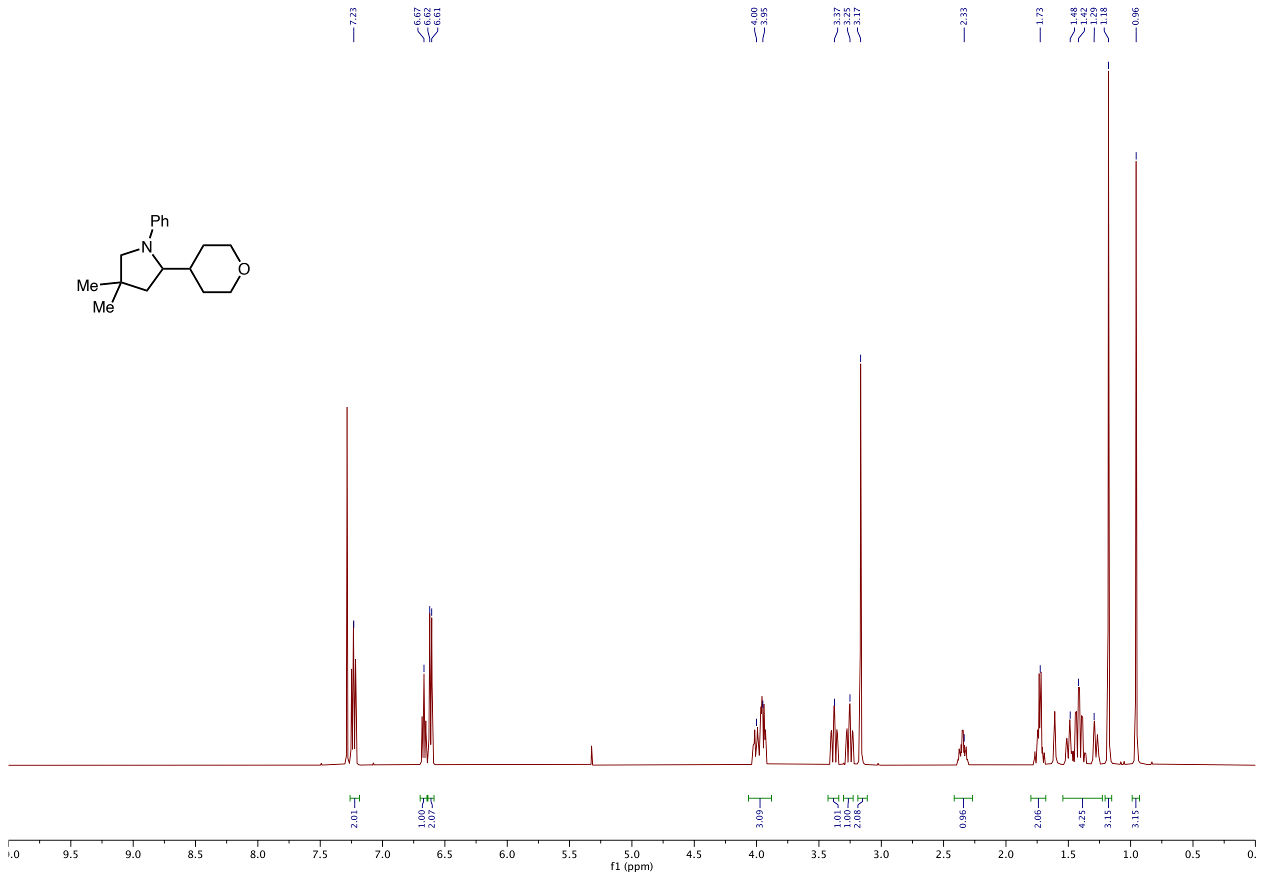
7.23
6.67
6.62
6.61
4.00
3.95
3.37
3.25
3.17
2.33
1.73
1.48
1.42
1.29
1.18
0.96
Ph
N
Me
Me
O
2.01
1.00
2.07
3.09
1.01
1.00
2.08
0.96
2.06
4.25
3.15
3.15
9.5
9.0
8.5
8.0
7.5
7.0
6.5
6.0
5.5
5.0
4.5
4.0
3.5
3.0
2.5
2.0
1.5
1.0
0.5
f1 (ppm)

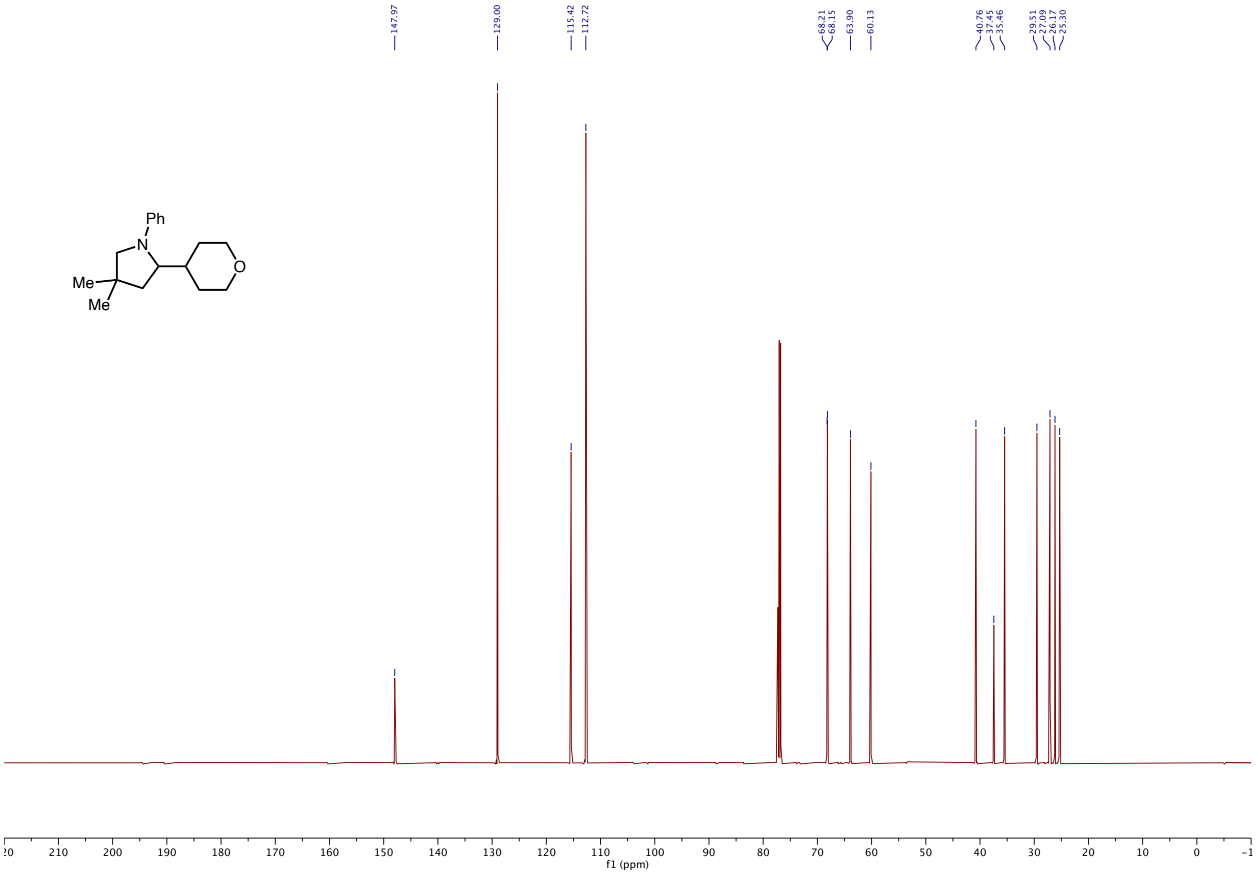
147.97
129.00
115.42
112.72
68.21
68.15
63.90
60.13
40.76
37.45
35.46
29.51
27.09
26.17
25.30
Ph
N
Me
Me
O
210
200
190
180
170
160
150
140
130
120
110
100
90
80
70
60
50
40
30
20
10
0
f1 (ppm)

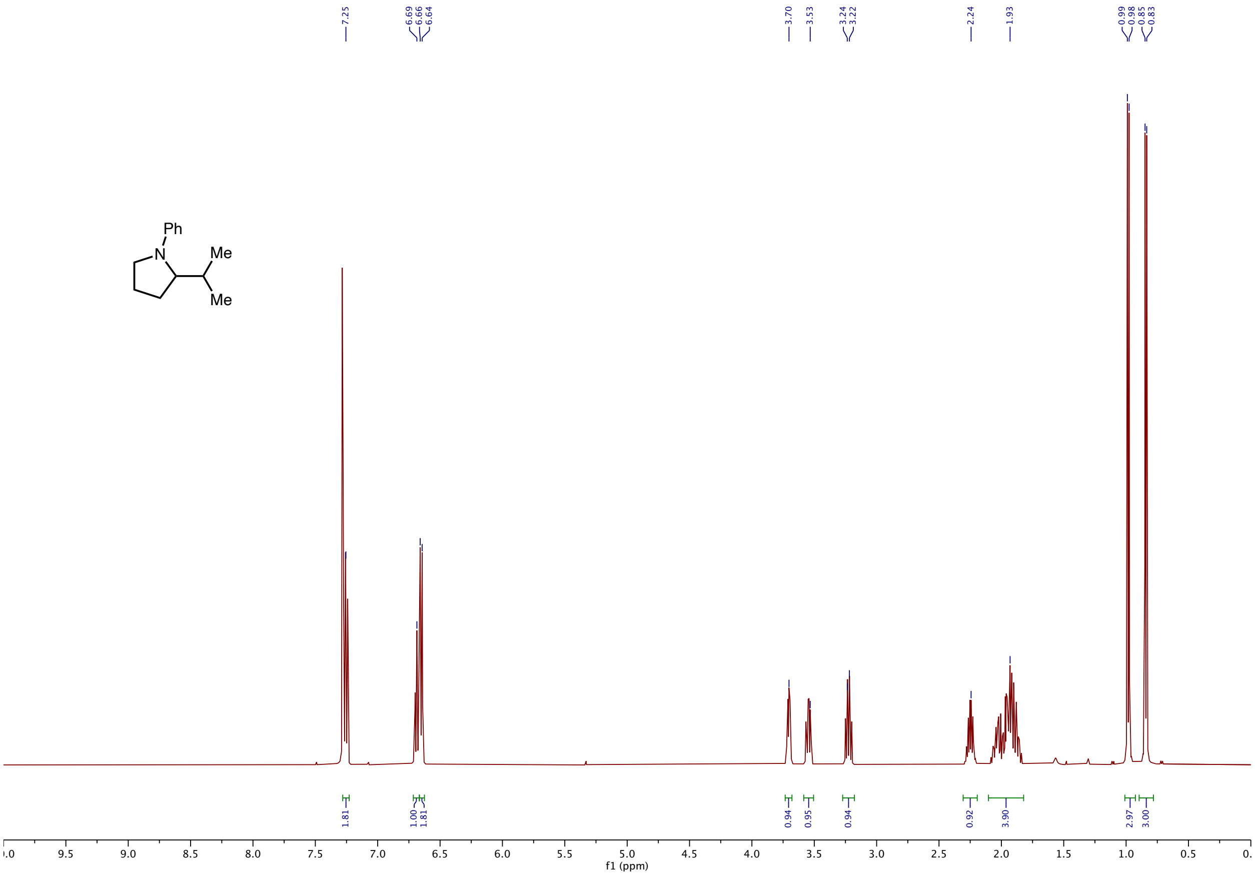
7.25
6.69
6.66
6.64
3.70
3.53
3.24
3.22
2.24
1.93
0.99
0.98
0.85
0.83
Ph
N
Me
Me
1.81
1.00
1.81
0.94
0.95
0.94
0.92
3.90
2.97
3.00
f1 (ppm)

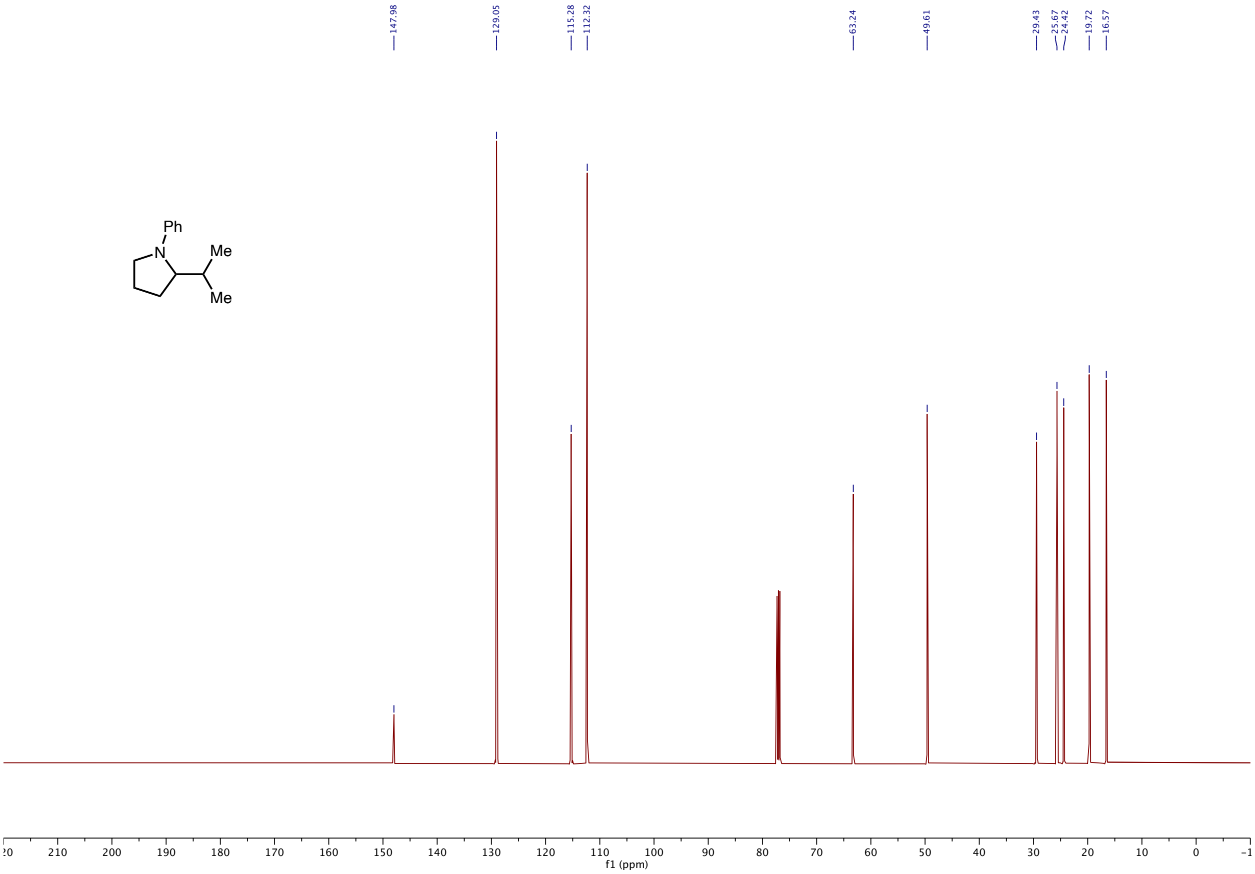
147.98
129.05
115.28
112.32
63.24
49.61
29.43
25.67
24.42
19.72
16.57
Ph
N
Me
Me
f1 (ppm)

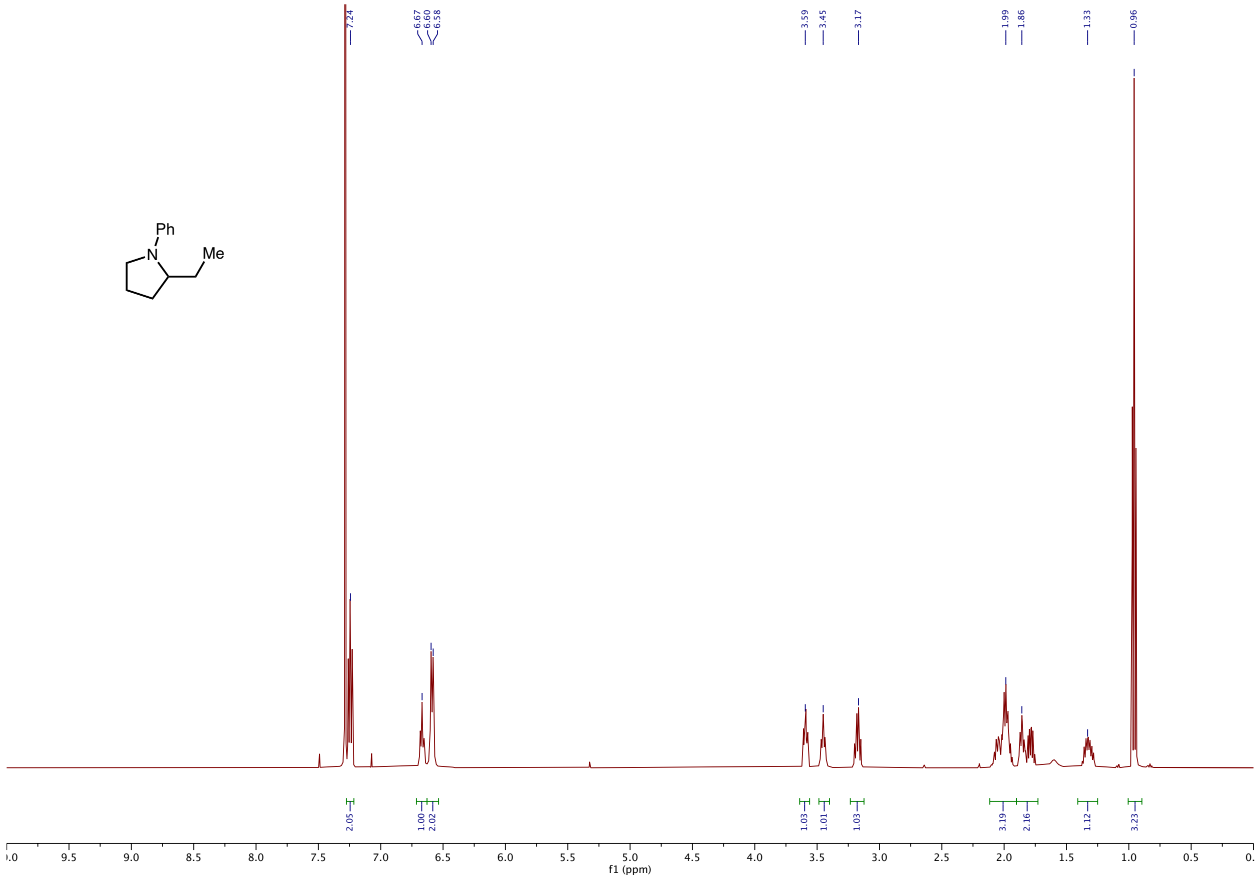
7.24
6.67
6.60
6.58
3.59
3.45
3.17
1.99
1.86
1.33
0.96
Ph
N
Me
2.05
1.00
2.02
1.03
1.01
1.03
3.19
2.16
1.12
3.23
f1 (ppm)

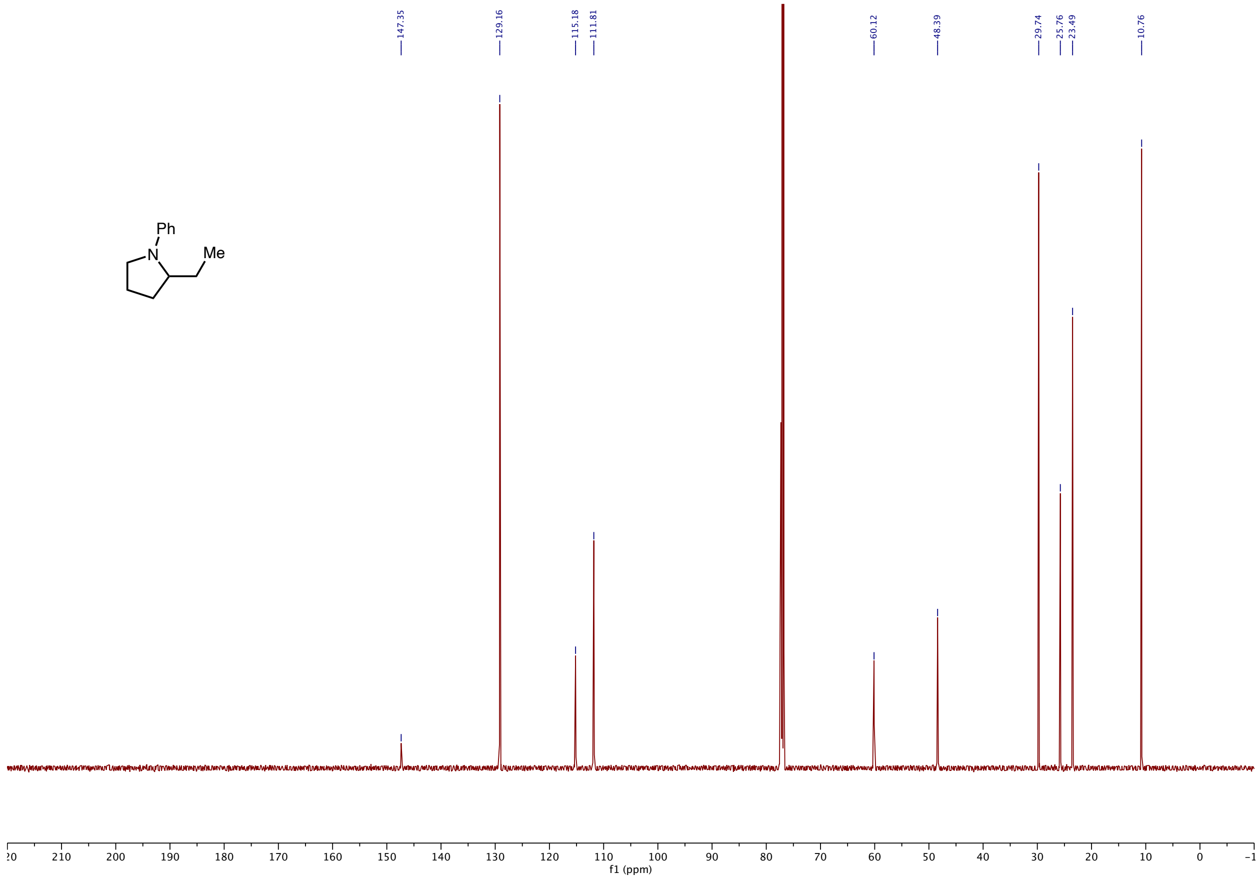
147.35
129.16
115.18
111.81
60.12
48.39
29.74
25.76
23.49
10.76
Ph
N
Me
f1 (ppm)

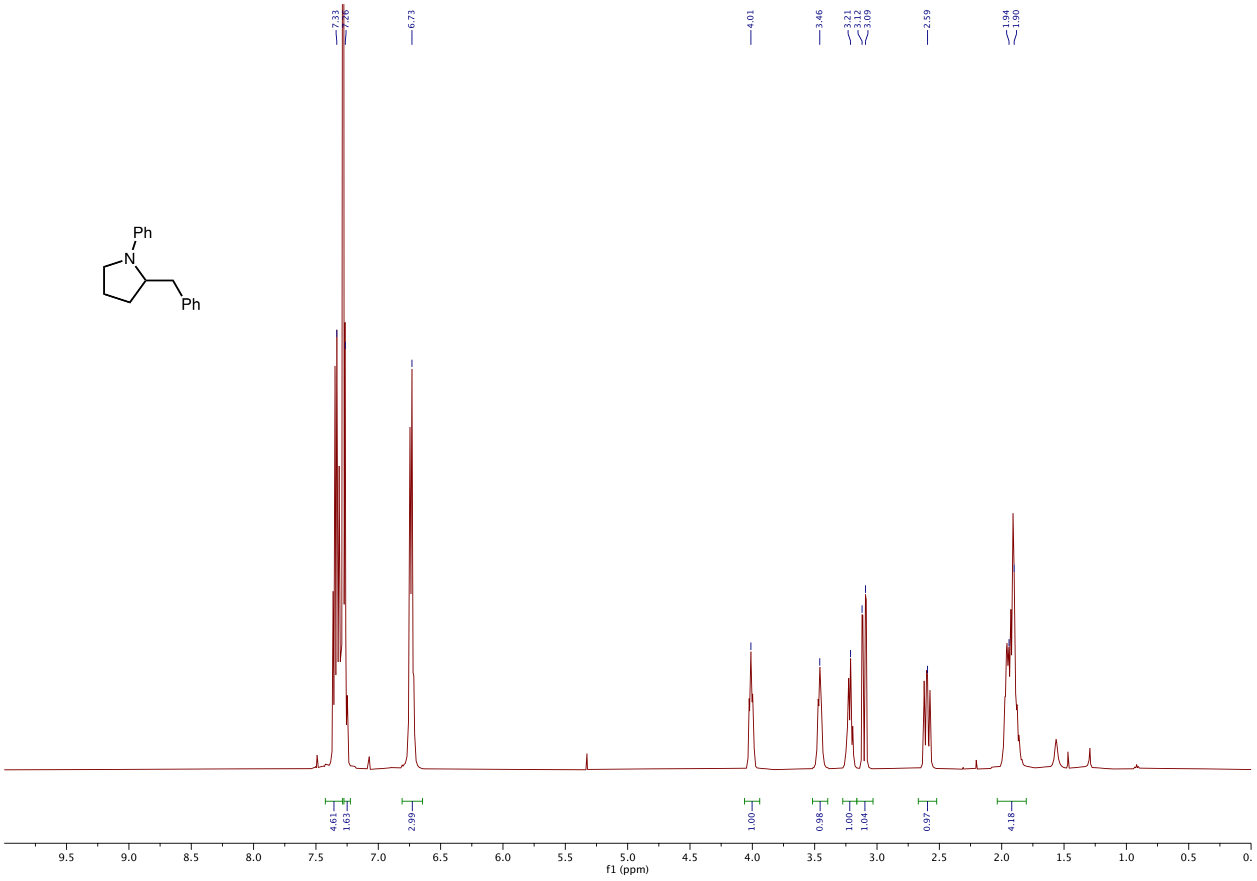
7.33
7.26
6.73
4.01
3.46
3.21
3.12
3.09
2.59
1.94
1.90
Ph
N
Ph
4.61
1.63
2.99
1.00
0.98
1.00
1.04
0.97
4.18
f1 (ppm)

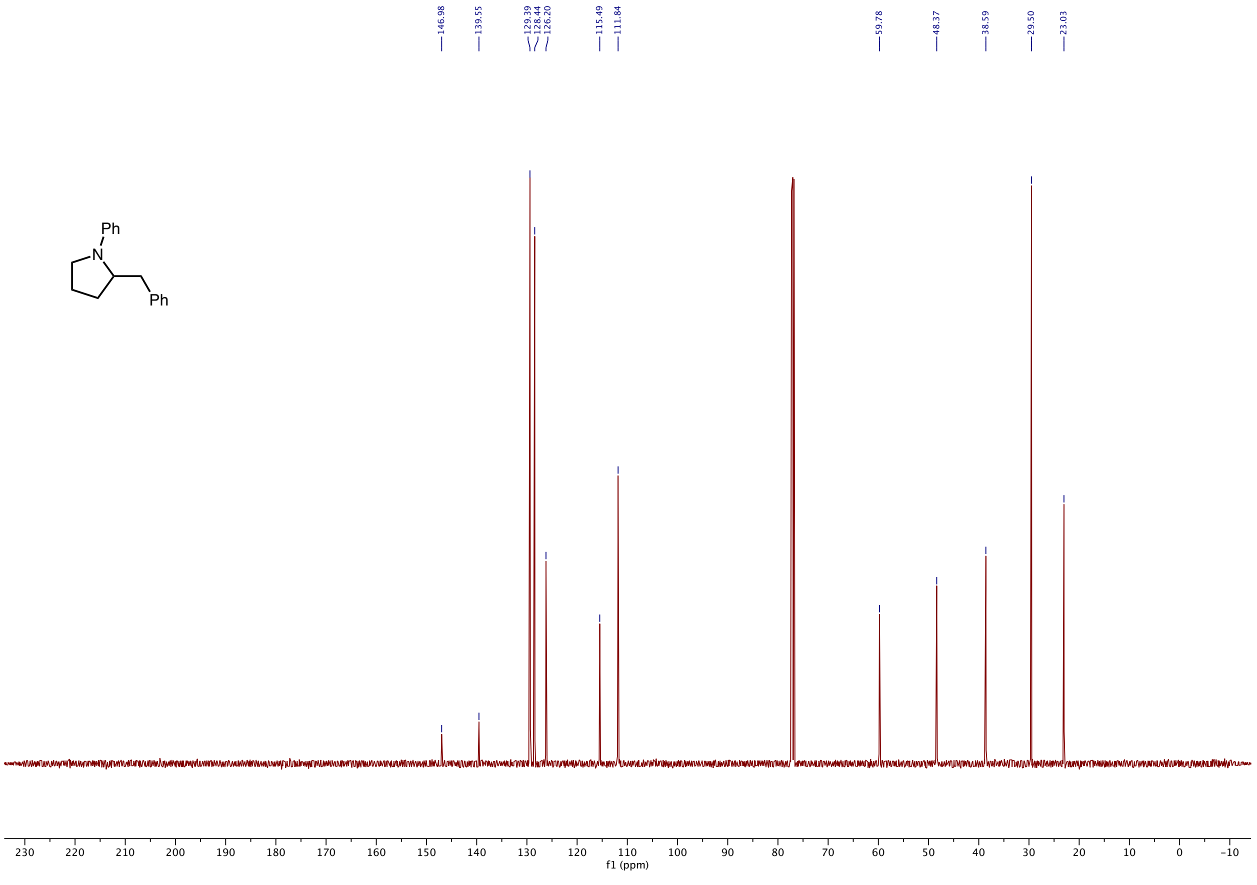
146.98
139.55
129.39
128.44
126.20
115.49
111.84
59.78
48.37
38.59
29.50
23.03
Ph
N
Ph
f1 (ppm)

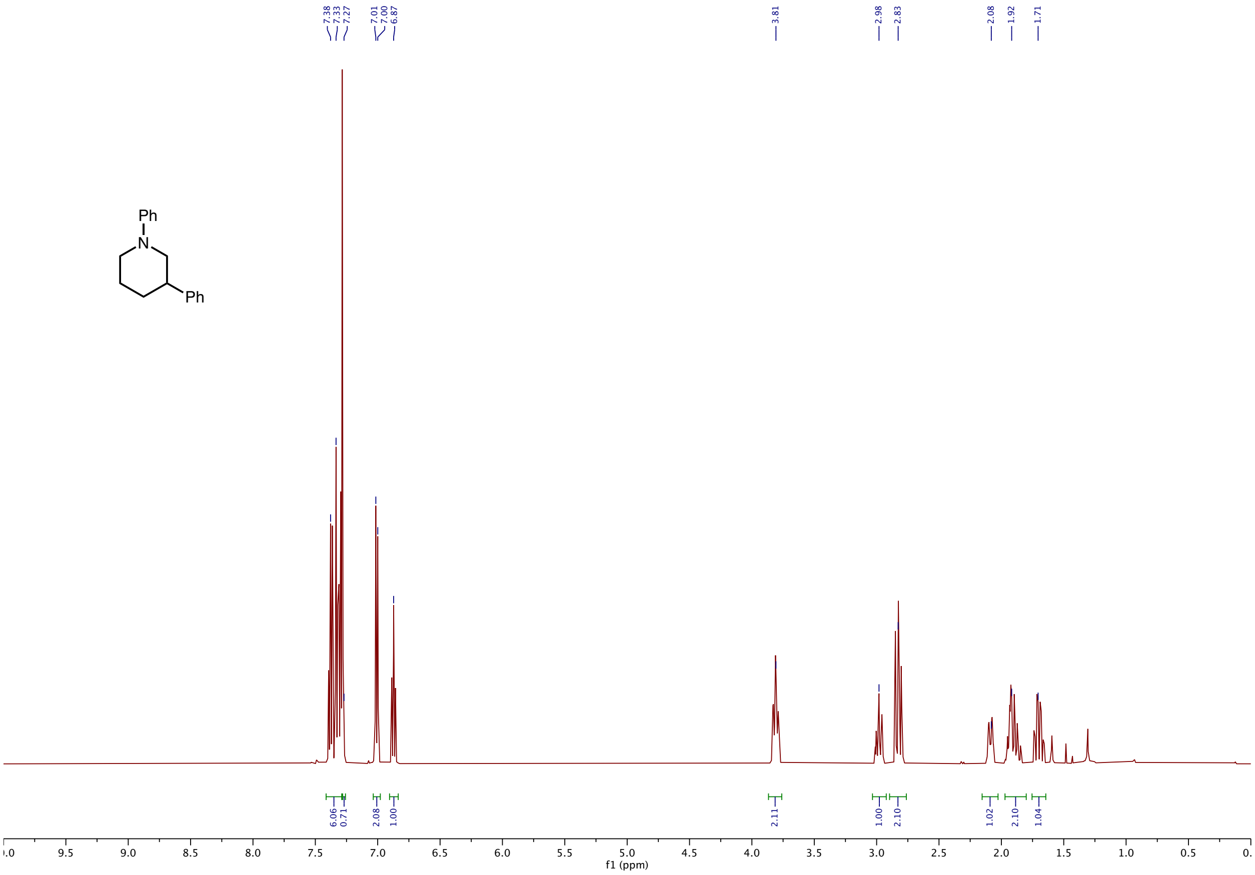
7.38
7.33
7.27
7.01
7.00
6.87
3.81
2.98
2.83
2.08
1.92
1.71
Ph
N
Ph
6.06
0.71
2.08
1.00
2.11
1.00
2.10
1.02
2.10
1.04
f1 (ppm)

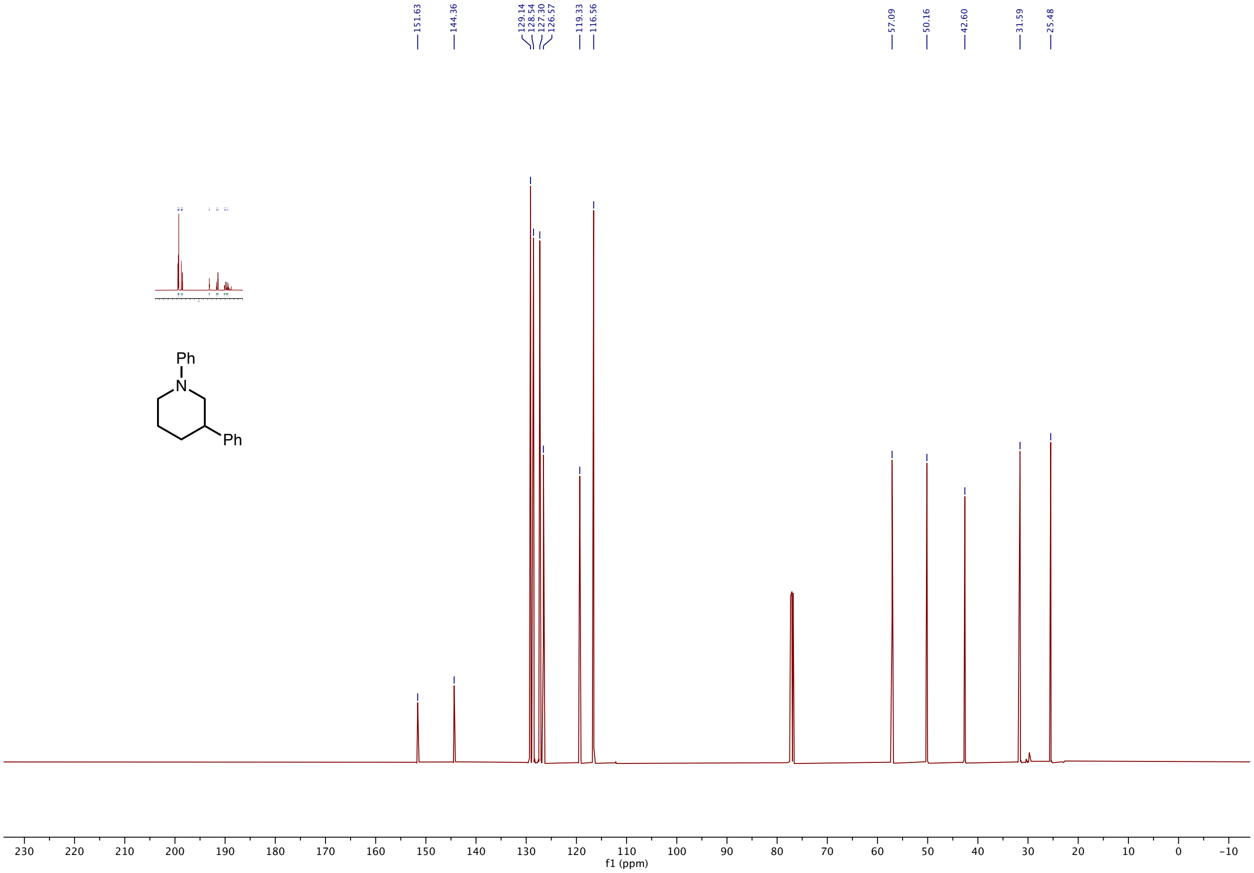
151.63
144.36
129.14
128.54
127.30
126.57
119.33
116.56
57.09
50.16
42.60
31.59
25.48
Ph
N
Ph
f1 (ppm)

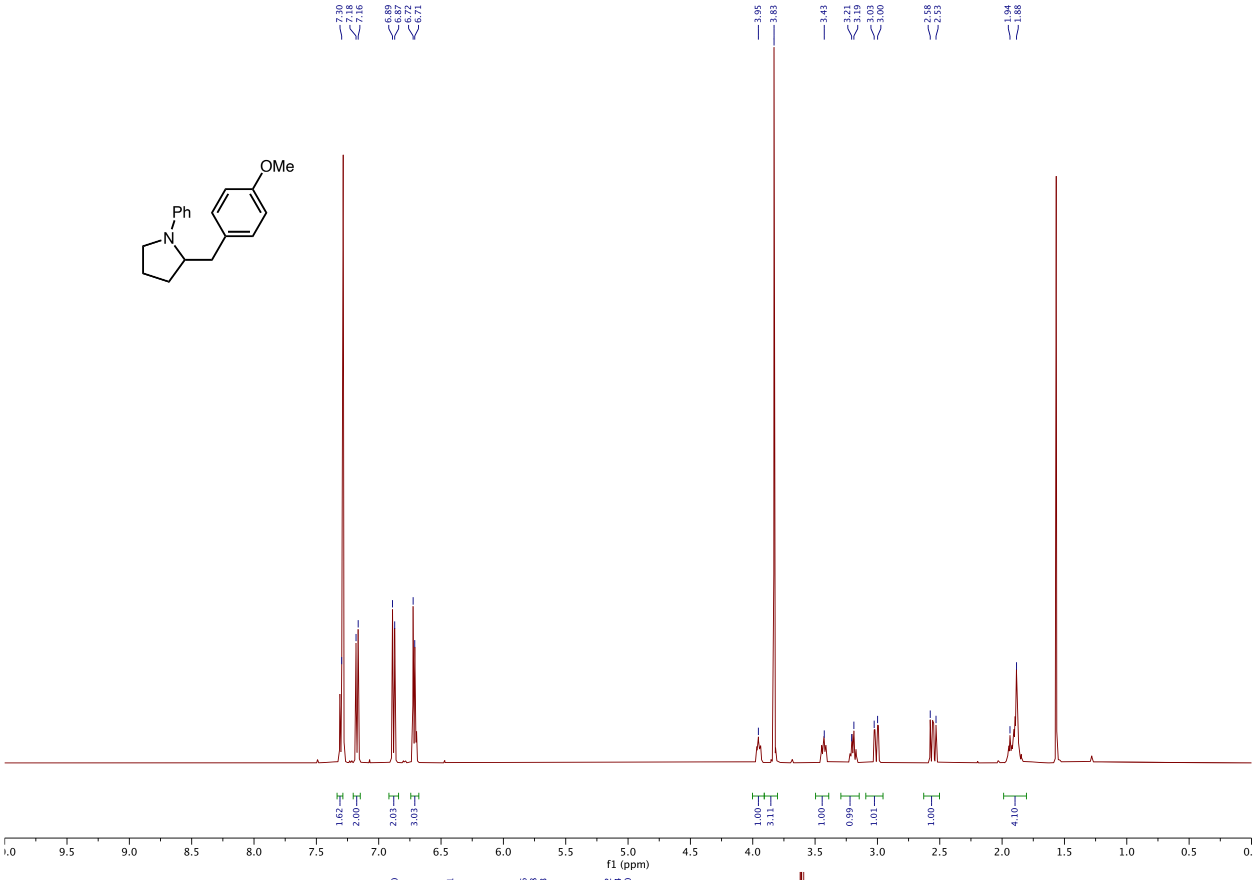

OMe
Ph
N
7.30
7.18
7.16
6.89
6.87
6.72
6.71
3.95
3.83
3.43
3.21
3.19
3.03
3.00
2.58
2.53
1.94
1.88
1.62
2.00
2.03
3.03
1.00
3.11
1.00
0.99
1.01
1.00
4.10
f1 (ppm)

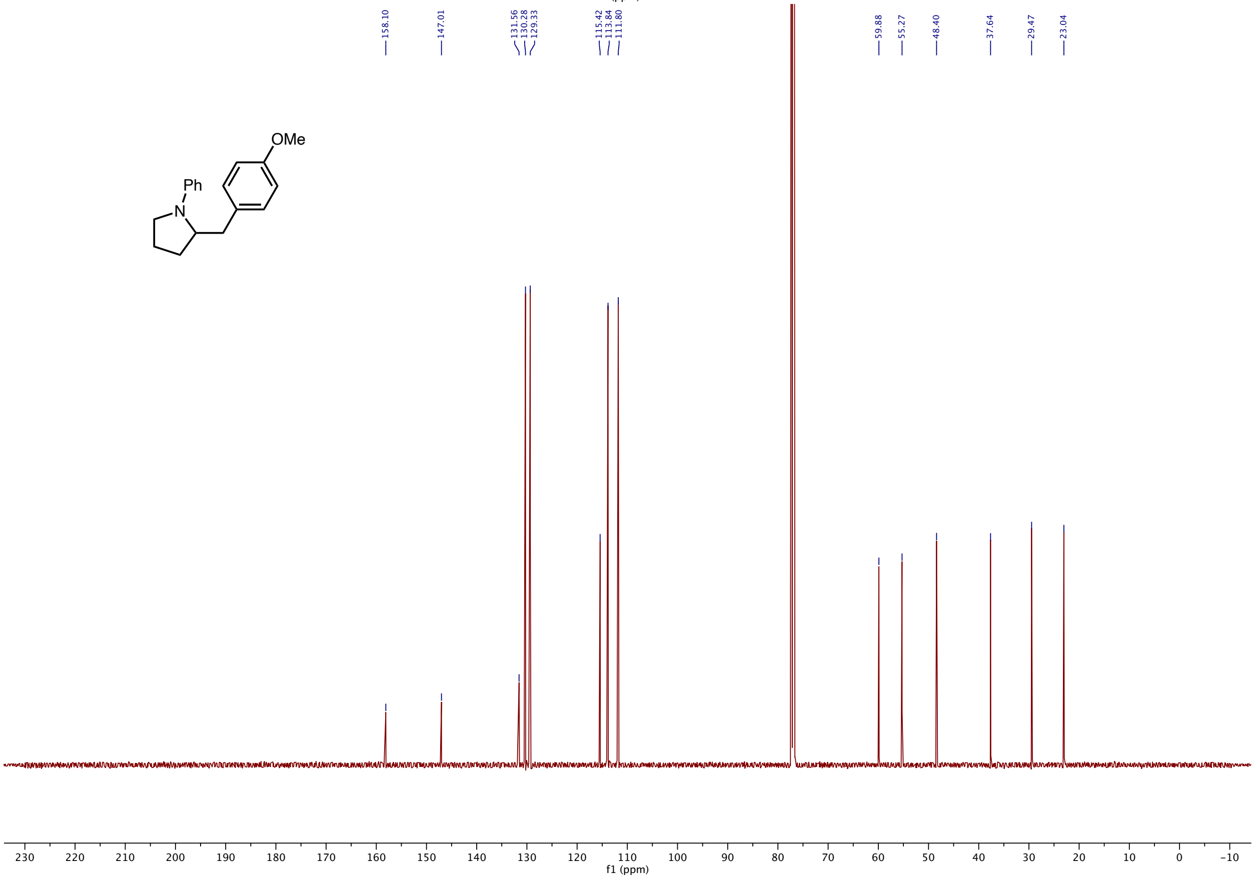

OMe
Ph
N
158.10
147.01
131.56
130.28
129.33
115.42
113.84
111.80
59.88
55.27
48.40
37.64
29.47
23.04
f1 (ppm)

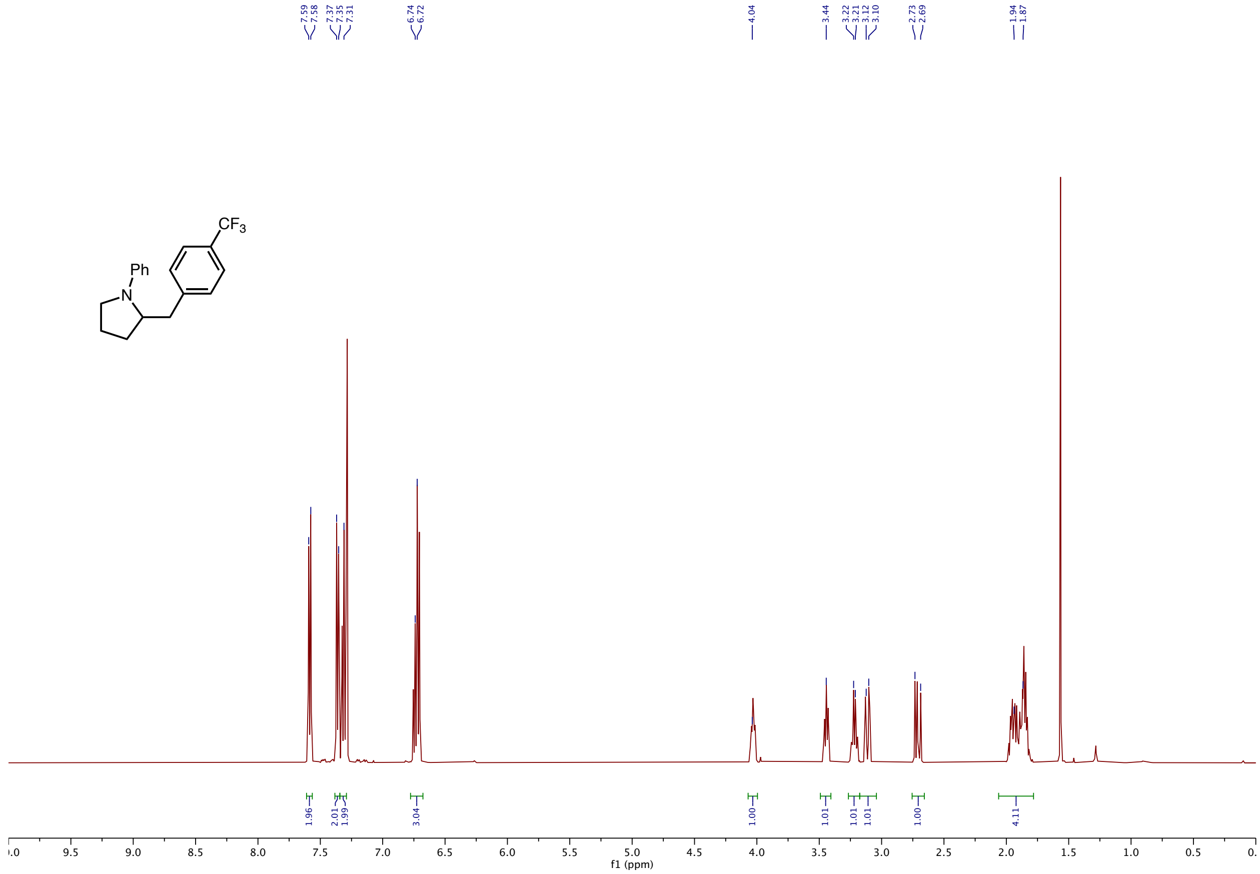
7.59
7.58
7.37
7.35
7.31
6.74
6.72
4.04
3.44
3.22
3.21
3.12
3.10
2.73
2.69
1.94
1.87
CF3
Ph
N
1.96
2.01
1.99
3.04
1.00
1.01
1.01
1.01
1.00
4.11
f1 (ppm)

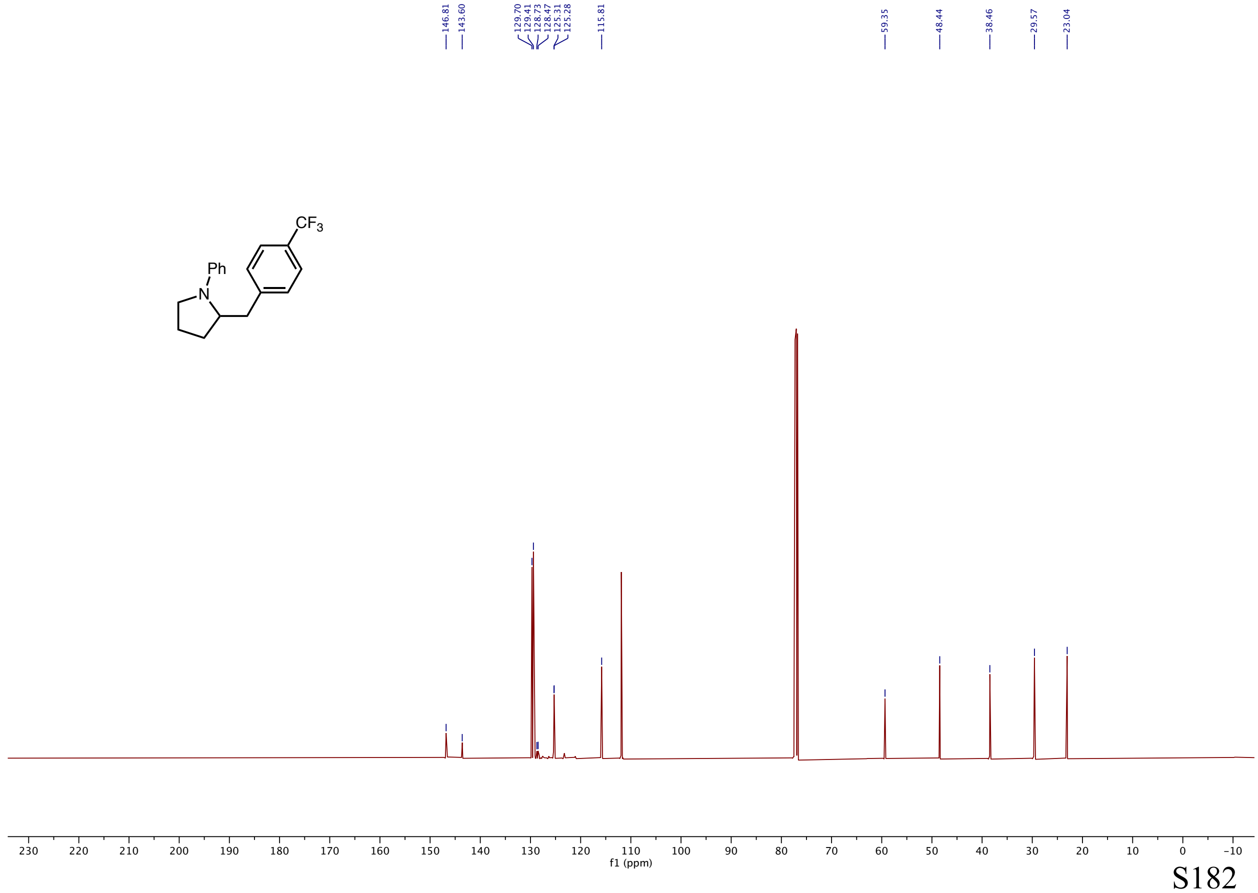
146.81
143.60
129.70
129.41
128.73
128.47
125.31
125.28
115.81
59.35
48.44
38.46
29.57
23.04
CF3
Ph
N
f1 (ppm)

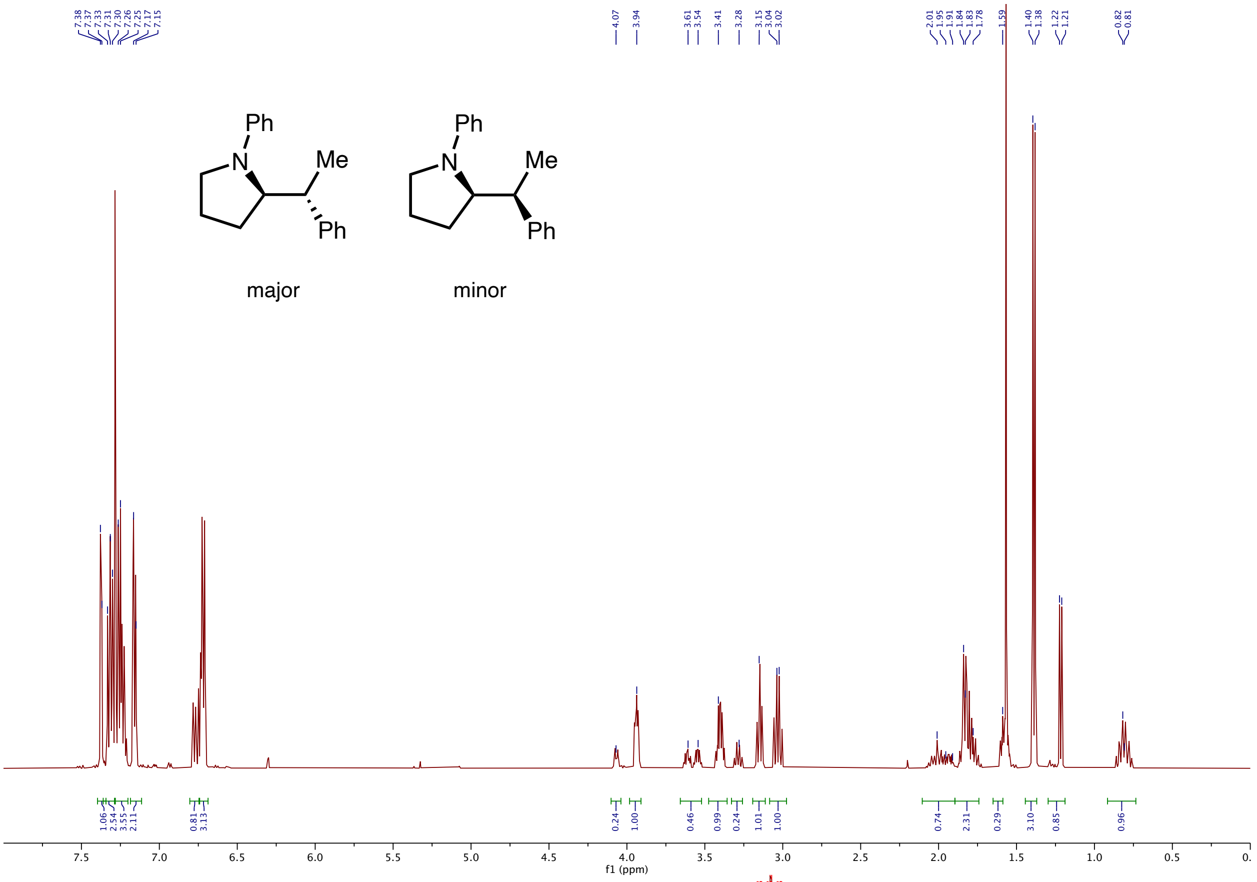

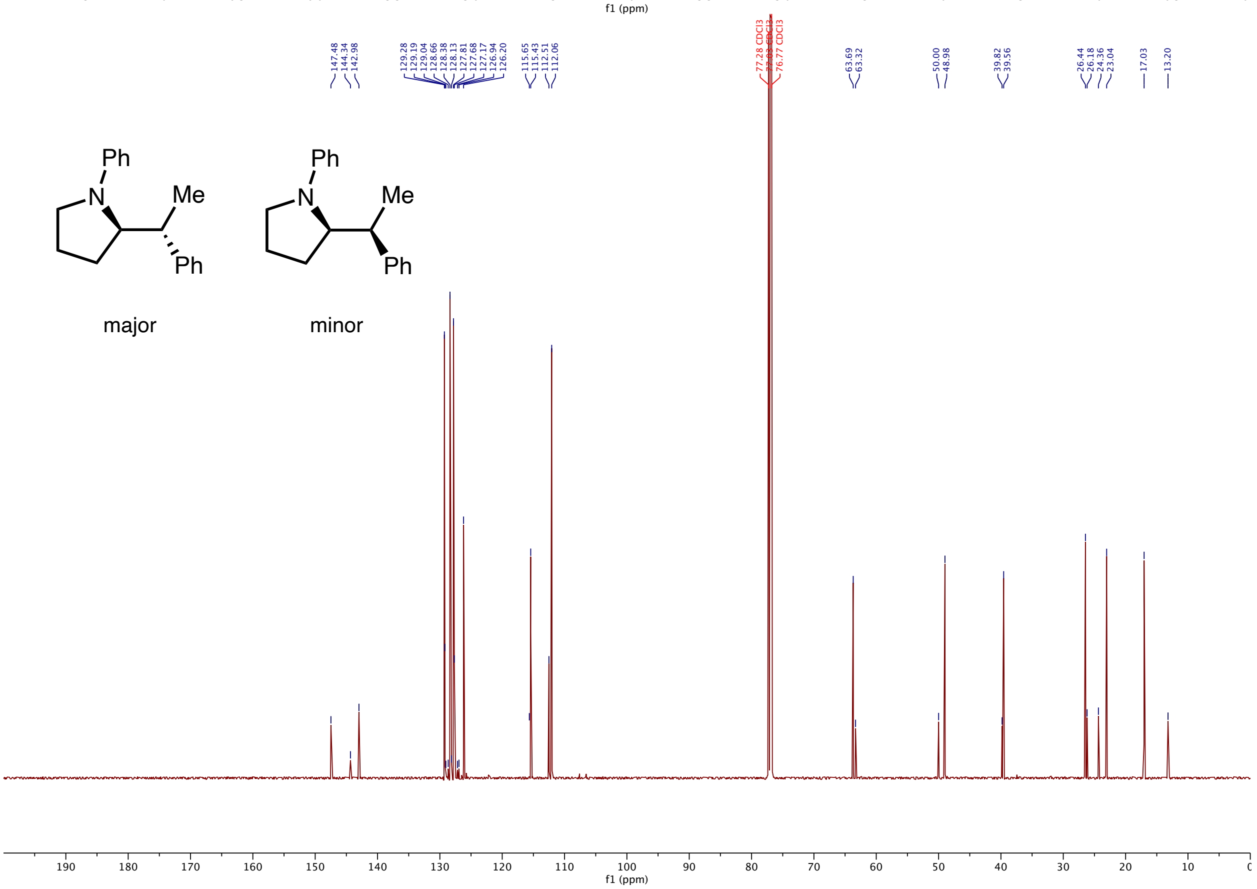

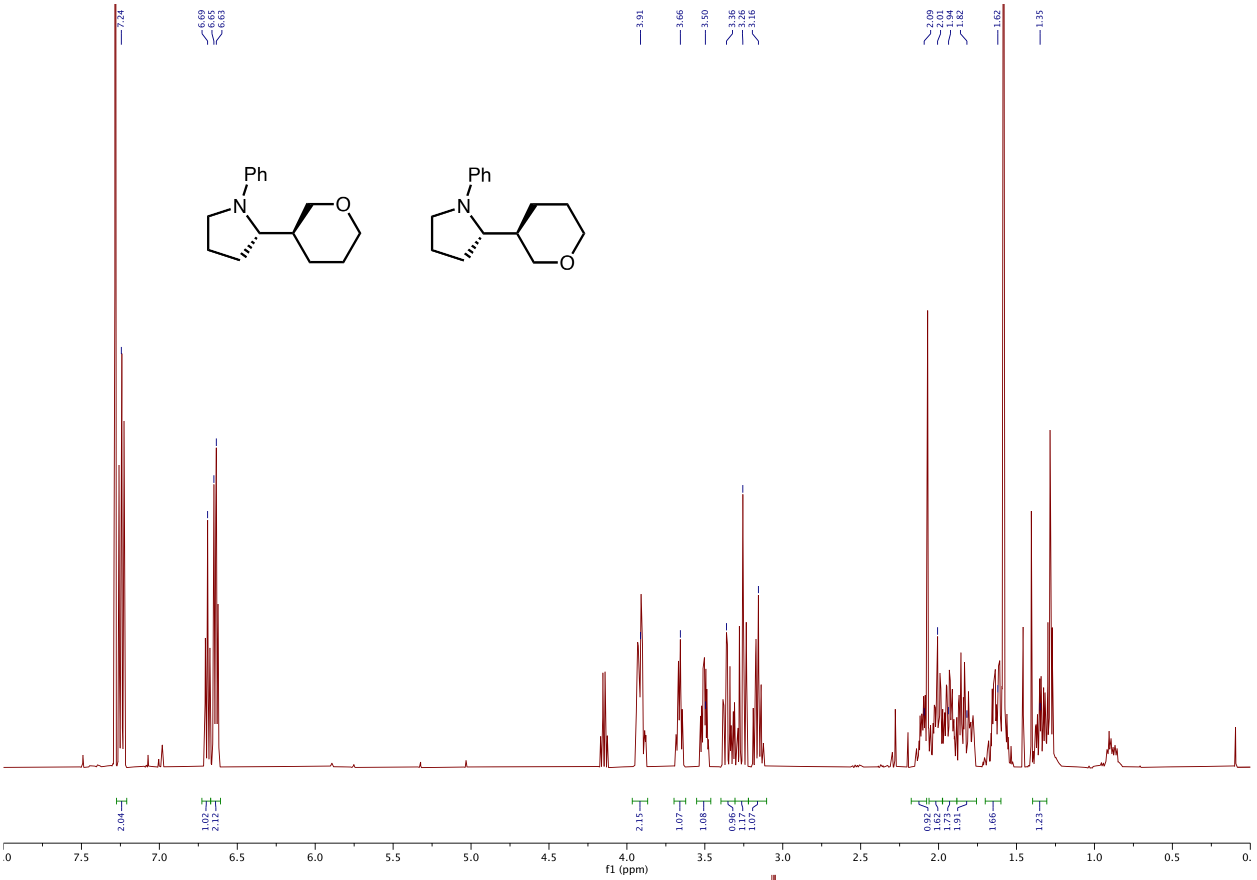

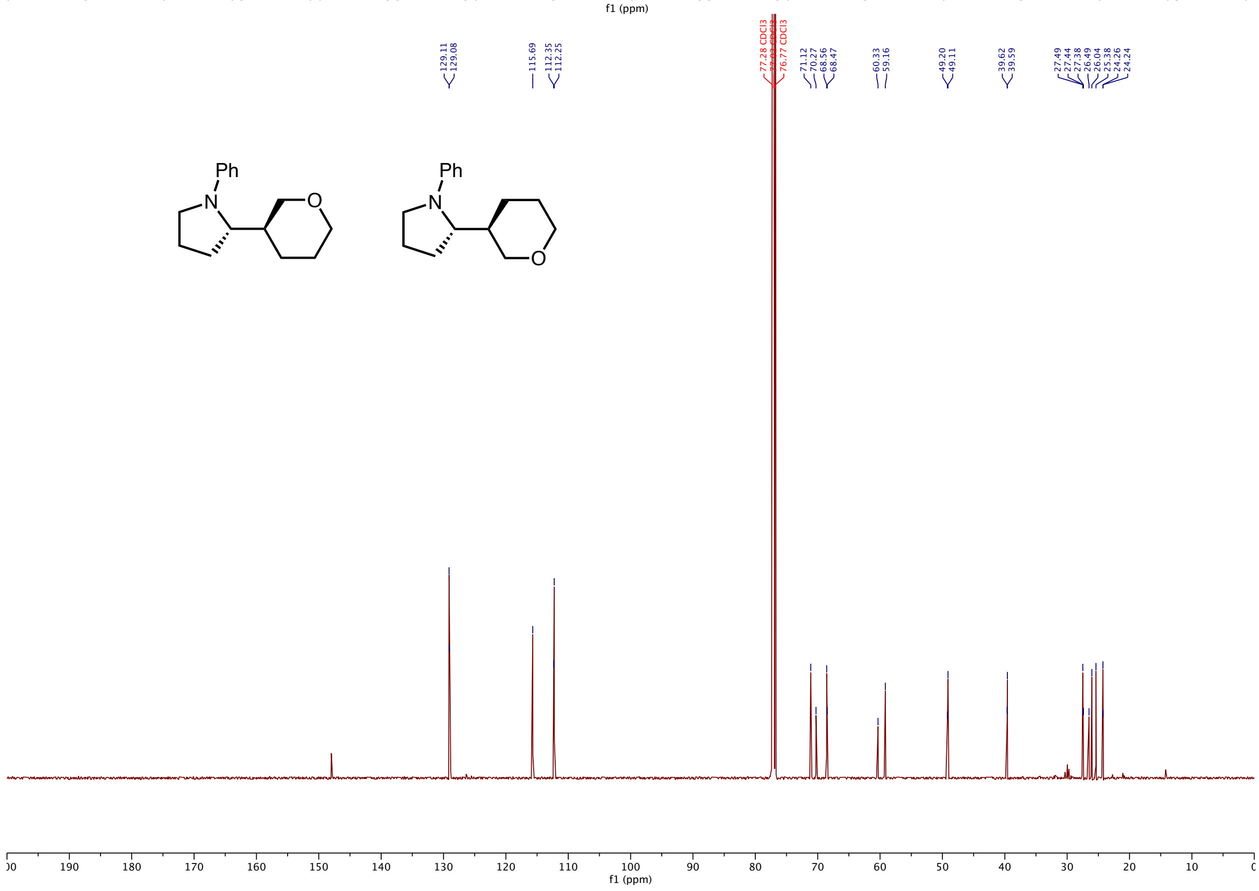

M. References:

(1) Sandoval, B. A.; Meichan, A. J.; Hyster, T. K. Enantioselective Hydrogen Atom Transfer: Discovery of Catalytic Promiscuity in Flavin-Dependent 'Ene'-Reductases. *J. Am. Chem. Soc.* **2017**, *139* (33), 11313–11316. https://doi.org/10.1021/jacs.7b05468.

(2) Kille, S.; Acevedo-Rocha, C. G.; Parra, L. P.; Zhang, Z. G.; Opperman, D. J.; Reetz, M. T.; Acevedo, J. P. Reducing Codon Redundancy and Screening Effort of Combinatorial Protein Libraries Created by Saturation Mutagenesis. *ACS Synth. Biol.* **2013**, *2* (2), 83–92. https://doi.org/10.1021/sb300037w.

(3) Page, C. G.; Cao, J.; Oblinsky, D. G.; Macmillan, S. N.; Dahagam, S.; Lloyd, R. M.; Charnock, S. J.; Scholes, G. D.; Hyster, T. K. Regioselective Radical Alkylation of Arenes Using Evolved Photoenzymes. *J. Am. Chem. Soc.* **2023**, *145* (21), 11866–11874. https://doi.org/10.1021/jacs.3c03607.

(4) Raps, F. C.; Rivas-Souchet, A.; Jones, C. M.; Hyster, T. K. Emergence of a Distinct Mechanism of C–N Bond Formation in Photoenzymes. *Nature* **2025**, *637* (8045), 362–368. https://doi.org/10.1038/s41586-024-08138-w.

(5) Johnston, C. P.; Smith, R. T.; Allmendinger, S.; MacMillant, D. W. C. Metallaphotoredox-Catalysed Sp3-Sp3 Cross-Coupling of Carboxylic Acids with Alkyl Halides. *Nat. Publ. Gr.* **2016**, *536*, 322–325. https://doi.org/10.1038/nature19056.

(6) Cordier, C. J.; Lundgren, R. J.; Fu, G. C. Enantioconvergent Cross-Couplings of Racemic Alkylmetal Reagents with Unactivated Secondary Alkyl Electrophiles: Catalytic Asymmetric Negishi α-Alkylations of N-Boc-Pyrrolidine. *J. Am. Chem. Soc.* **2013**, *135* (30), 10946–10949. https://doi.org/10.1021/ja4054114.

(7) Wang, X.; Long, C. Y.; Su, M. H.; Qu, Y. X.; Li, S. H.; Zhang, X. J.; Huang, S. J.; Wang, X. Q. Rapid Amination of Methoxy Pyridines with Aliphatic Amines. *Org. Process Res. Dev.* **2019**, *23* (8), 1587–1593. https://doi.org/10.1021/acs.oprd.9b00235.

(8) Musacchio, A. J.; Nguyen, L. Q.; Beard, G. H.; Knowles, R. R. Photoredox Method for Direct C − N Bond Formation. *J. Am. Chem. Soc.* **2014**, 15–18.

(9) Musacchio, A. J.; Lainhart, B. C.; Zhang, X.; Naguib, S. G.; Sherwood, T. C.; Knowles, R. R. Catalytic Intermolecular Hydroaminations of Unactivated Olefins with Secondary Alkyl Amines. *Science* **2017**, *355* (6326), 727–730. https://doi.org/10.1126/science.aal3010.
